# Selectivity of tungsten mediated dinitrogen splitting *vs.* proton reduction†
†Electronic supplementary information (ESI) available. CCDC 1943888–1943892. For ESI and crystallographic data in CIF or other electronic format see DOI: 10.1039/c9sc03779a


**DOI:** 10.1039/c9sc03779a

**Published:** 2019-09-24

**Authors:** Bastian Schluschaß, Josh Abbenseth, Serhiy Demeshko, Markus Finger, Alicja Franke, Christian Herwig, Christian Würtele, Ivana Ivanovic-Burmazovic, Christian Limberg, Joshua Telser, Sven Schneider

**Affiliations:** a Georg-August-Universität , Institut für Anorganische Chemie , Tammannstrasse 4 , 37077 Göttingen , Germany . Email: sven.schneider@chemie.uni-goettingen.de; b Lehrstuhl für Bioanorganische Chemie , Department Chemie und Pharmazie , Friedrich-Alexander-Universität Erlangen , Egerlandstrasse 3 , 91058 Erlangen , Germany; c Institut für Chemie , Humboldt Universität zu Berlin , Brook-Taylor-Strasse 2 , 12489 Berlin , Germany; d Department of Biological, Physical and Health Sciences , Roosevelt University , 430 S. Michigan Avenue , Chicago , Illinois 60605 , USA

## Abstract

Mo complexes are currently the most active catalysts for nitrogen fixation under ambient conditions. In comparison, tungsten platforms are scarcely examined. For active catalysts, the control of N_2_*vs.* proton reduction selectivities remains a difficult task. We here present N_2_ splitting using a tungsten pincer platform, which has been proposed as the key reaction for catalytic nitrogen fixation. Starting from [WCl_3_(PNP)] (PNP = N(CH_2_CH_2_P*t*Bu_2_)_2_), the activation of N_2_ enabled the isolation of the dinitrogen bridged redox series [(N_2_){WCl(PNP)}_2_]^0/+/2+^. Protonation of the neutral complex results either in the formation of a nitride [W(N)Cl(*H*PNP)]^+^ or H_2_ evolution and oxidation of the W_2_N_2_ core, respectively, depending on the acid and reaction conditions. Examination of the nitrogen splitting *vs.* proton reduction selectivity emphasizes the role of hydrogen bonding of the conjugate base with the protonated intermediates and provides guidelines for nitrogen fixation.

## Abstract 2

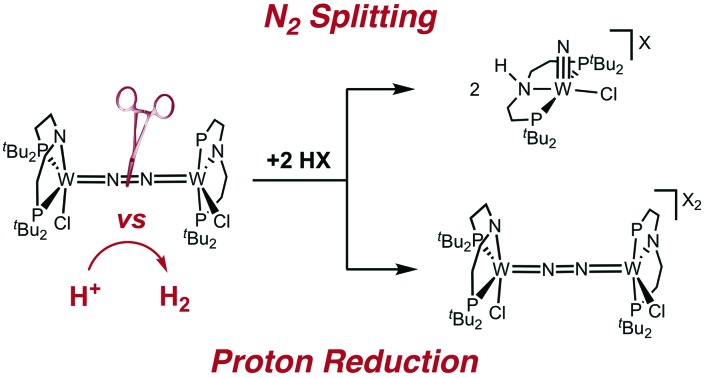
An N_2_-bridged ditungsten complex is presented that undergoes N_2_-splitting or hydrogen evolution upon protonation depending on the acid and reaction conditions. Spectroscopic, kinetic and computational results emphasize the impact of hydrogen bonding on the reaction selectivity.

## Introduction

Homogeneous N_2_ fixation under ambient conditions has made remarkable progress over the past 15 years.^1^ Nishibayashi and co-workers recently obtained over 4000 equiv. of NH_3_ with the proton coupled electron transfer (PCET) reagent H_2_O/SmI_2_ as H^+^/e^–^ sources and a molecular Mo pincer catalyst.^2^ Accordingly, nitrogen reduction (NR) *via* electrochemically or light-driven PCET with related systems has attracted a lot of attention.^3^,^4^ Lindley *et al.* estimated a suitable overpotential window of 1–1.5 V for selective NR (in MeCN) prior to competing hydrogen reduction (HR) at a glassy carbon cathode.^5^ However, besides the thermochemical framework, mechanistic models that account for NR *vs.* HR selectivities of molecular catalysts are generally poorly developed.

**Scheme 1:**
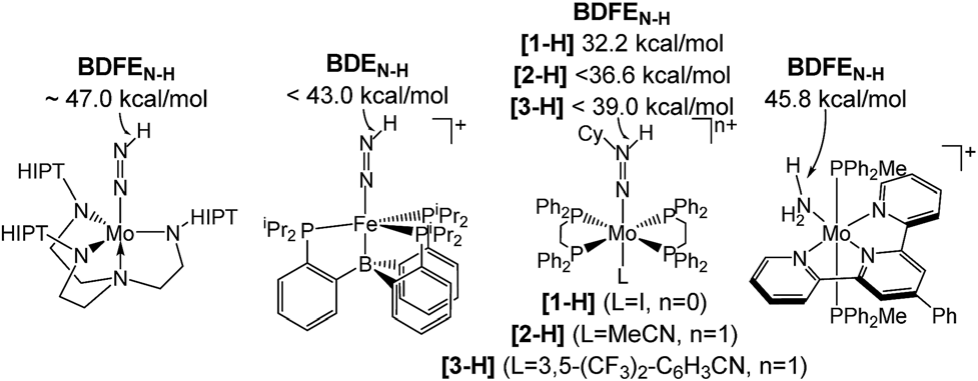
Transition metal species relevant to N_2_ fixation with a low BDFE_N–H_.^7^

Several M(N_*x*_H_*y*_) intermediates relevant to N_2_ fixation (Scheme 1) exhibit low N–H bond dissociation free energies (BDFEs) below that of free H_2_ (BDFE(H_2_, gas) = 97.2 kcal mol^–1^) as possible branching points into HR.^6^,^7^ Computational evaluation of NR *vs.* HR selectivities for a series of Fe catalysts pointed at bimolecular H_2_ loss from species with low N–H BDFEs.^8^,^9^ Attempts to stabilize such Fe(N_*x*_H_*y*_) species by hydrogen bonding with pendant bases so far resulted in shutdown of catalysis.^10^ But, in fact, such secondary interactions might also be relevant for Nishibayashi's catalyst as indicated by selectivities obtained with 2,6-lutidinium acids ([LutH]^+^[X]^–^) as the proton source. These strongly depend on the X^–^ counter anion: NH_3_/H_2_ (X^–^) = 7.0 (Cl^–^), 0.9 (OTf^–^), and 0.14 (BAr_4_^–^).^11^

**Fig. 1:**
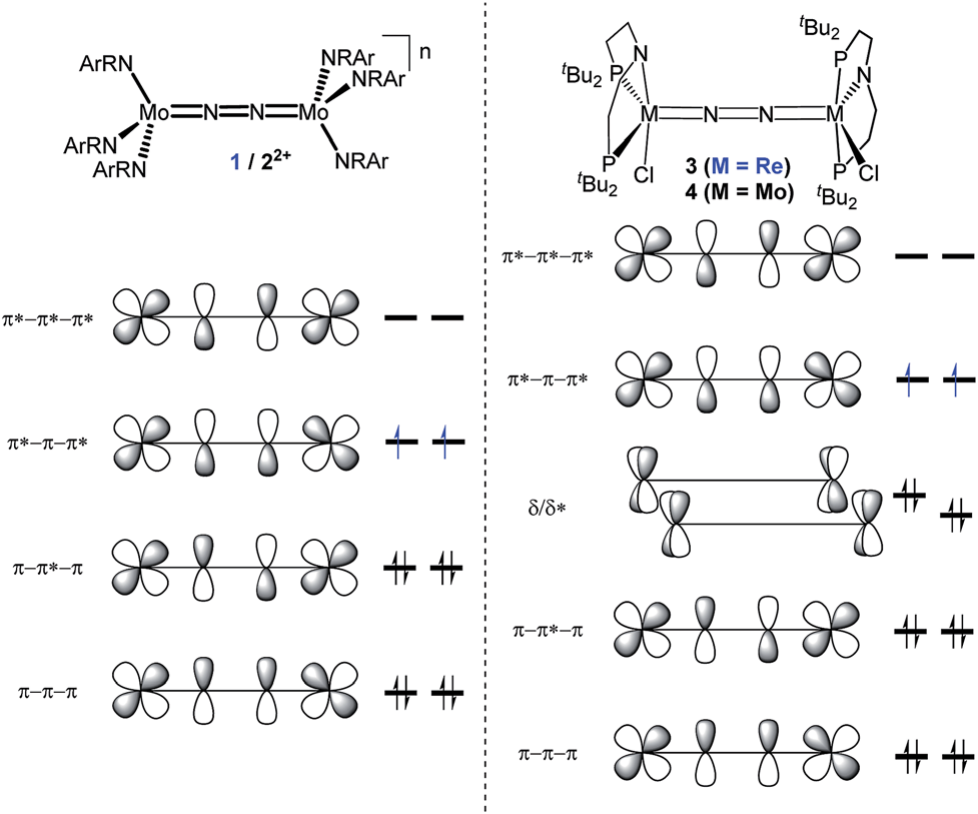
Qualitative molecular orbital schemes for **1**, **2^2+^**, **3** and **4**, illustrating the isolobal relationship of **1** {π^10^}/**3** {π^10^δ^4^} and **2^2+^** {π^8^}/**4** {π^8^δ^4^}, respectively.

In this contribution, we address the role of hydrogen bonding for the selectivity of proton induced N_2_ splitting into molecular nitrides *vs.* proton reduction. N_2_ splitting has evolved as an entry into N_2_ functionalization for a wide variety of metals^12^ and was proposed as the key step in N_2_ fixation with Mo pincer catalysts.^2^,^13^ Cleavage of Cummins' seminal complex **1** (Fig. 1) was attributed to the {π_1_^2^π_2_^2^π_3_^2^π_4_^2^π_5_^1^π_6_^1^} configuration of the Mo_2_N_2_-core, which enables population of a destabilizing σ-antibonding molecular orbital (MO) in the transition state.^14^,^15^ In contrast, the {π^8^} oxidation product **2^2+^** exhibits strong N_2_ activation (Table 1) but lacks two electrons to form stable Mo^VI^(N^3–^) nitrides.^16^

**Table 1:** Comparison of the spectroscopic and structural features of Cummins' Mo–N_2_-dimer redox-series (Ar = C_6_H_3_-3,5-Me_2_)^16^ with square-pyramidal pincer complexes (M = Re,^17^ Mo,^19^ or W; * computed value)

	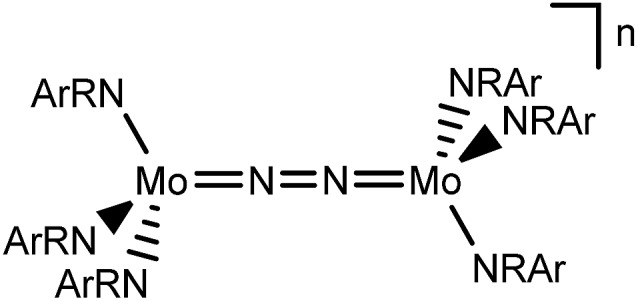			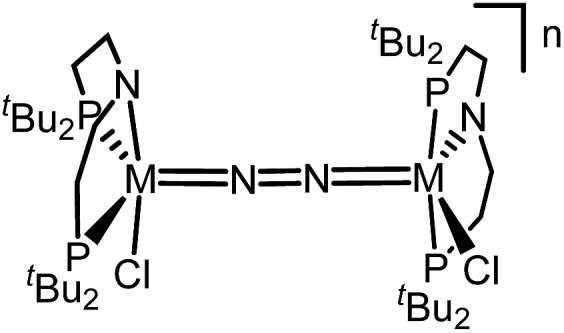				
Compound	**1**	—	**2^2+^**	**3** (M = Re)	**4** (M = Mo)	**6** (M = W)	**7^+^** (M = W)	**8^2+^** (M = W)
*n*	0	+1	+2	0	0	0	+1	+2
Configuration	{π^10^}	{π^9^}	{π^8^}	{π^10^δ^4^}	{π^8^δ^4^}	{π^8^δ^4^}	{π^8^δ^3^}	{π^8^δ^2^}
*d*(NN) [Å]	1.212(2)/1.217(2)	1.239(4)	1.265(5)	1.202(10)	1.258(9)	1.33(4)/1.27(8)	1.277(5)	1.266(12)
*ν*(NN) [cm^–1^]	1630	1503	1349	1771*	1343	1392	1414	1400
*S*	1	1/2	0	1	0	0	1/2	1

**Scheme 2:**
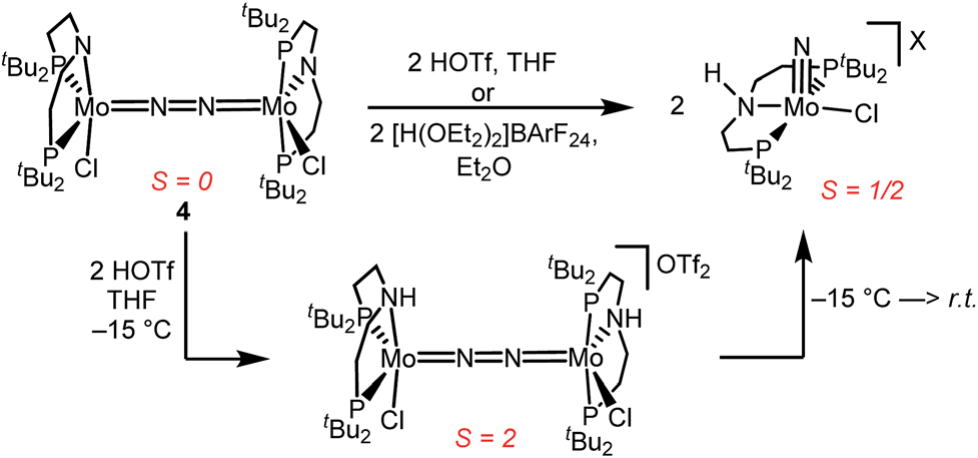
Dinitrogen splitting coupled to pincer protonation.

Similar electronic structure considerations can be applied to metal pincer platforms developed by our group. For example, the square-pyramidally coordinated dinuclear Re complex [(N_2_){ReCl(PNP)}_2_] (**3**, PNP = N(CH_2_CH_2_P*t*Bu_2_)_2_) also splits into nitrides at r.t. and exhibits a {π^10^δ^4^} configuration that is isolobal with **1** (Fig. 1).^17^,^18^ In contrast, the {π^8^δ^4^} complex [(N_2_){MoCl(PNP)}_2_] (**4**) features stronger N_2_-activation comparable to **2^2+^** (Table 1), but is thermally stable.^19^ Unexpectedly, splitting of **4** at r.t. was obtained upon protonation of the pincer backbone (Scheme 2), which was rationalized by a protonation induced low-spin {π^8^δ^4^} to high-spin {π^10^δ^2^} transition that facilitates electron transfer to N_2_.

Given the impressive N_2_ fixation rates with Mo pincer catalysts which possibly proceed *via* N_2_ splitting;^2^,^13^ W nitride formation from N_2_ is surprisingly rare.^20^ We here report tungsten mediated N_2_ splitting that competes with proton reduction upon protonation of a {W_2_N_2_} pincer complex. Our results provide evidence for the significance of hydrogen bonding for the reaction selectivity.

## Results and discussion

### Synthesis of the [(N_2_){WCl(PNP)}_2_]^*n*+^ (*n* = 0–2) redox series

The reaction of WCl_4_ with ^H^PNP in the presence of NEt_3_ gives the pincer complex [WCl_3_(PNP)] (**5**) in yields of up to 60%. In the absence of a signal in the ^31^P{^1^H} NMR spectrum, the paramagnetically shifted ^1^H NMR signals and the solution magnetic moment derived by Evans' method (*μ*_eff_ = 2.8 ± 0.1 *μ*_B_) are in line with a d^2^ high-spin (*S* = 1) configuration. The molecular structure obtained by X-ray diffraction closely resembles previously reported compounds [MCl_3_(PNP)] (M = Re and Mo).18a,^19^

**Scheme 3:**
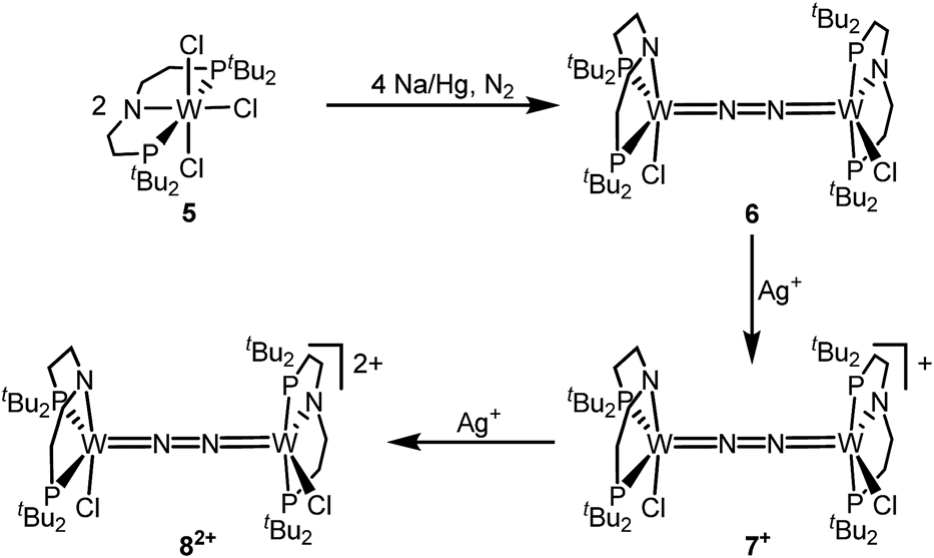
Preparation of the N_2_-bridged ditungsten redox series **6**–**7^+^**–**8^2+^**.

**Fig. 2:**
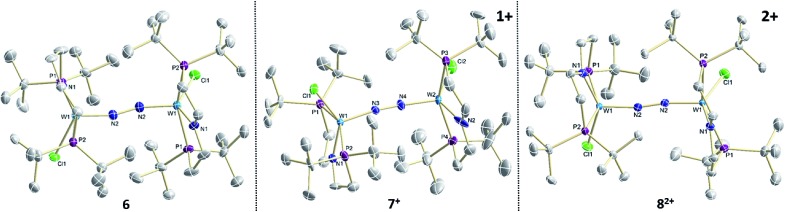
Molecular structures of **6**, **7^+^** and **8^2+^** in the crystal obtained by single crystal X-ray diffraction. Hydrogen atoms and anions are omitted for clarity.

Reduction of **5** with Na/Hg (2 equiv.) under N_2_ (1 atm) in THF gives the green, N_2_-bridged dinuclear complex [(N_2_){WCl(PNP)}_2_] (**6**) in up to 66% isolated yield (Scheme 3). In the solid state (Fig. 2) **6** is isostructural with the molybdenum analogue **4**, regarding the N_2_ binding mode, the approximate *C*_2_ symmetry due to mutually twisted {WCl(PNP)}-fragments (Cl1-W1-W1#-Cl1#: 89.59°/92.27°), and the distorted square-pyramidal metal coordination (*τ* = 0.35).^21^ The short W–N_2_ bond (1.78(2)/1.82(4) Å) indicates multiple bonding character. In turn, the N–N bond (1.33(4)/1.27(8) Å) is at the higher end for N_2_-bridged ditungsten complexes.^22^ The two ^31^P{^1^H} NMR signals (*δ*_P_ = 92.9, 87.8 ppm) with large *trans*-coupling (^2^*J*_PP_ = 147 Hz), the singlet ^15^N NMR resonance (*δ*_N_ = 31.1 ppm), and the ^1^H NMR signature of **6** support a *C*_2_ symmetric structure also in solution on the NMR timescale.

The N_2_ stretching vibration of **6** was assigned to the Raman signal at 1392 cm^–1^ (*λ*_exc_ = 457 nm, THF solution; ^15^N_2_ isotopologue: 1347 cm^–1^) supporting strong N_2_-activation with a formal N–N bond order below the double bonding character (*trans*-diazene: *ν*_NN_ = 1529 cm^–1^).^23^ The closed-shell ground state and degree of N_2_ activation are in line with the covalent bonding picture described in Fig. 1. The {π^8^*δ*^4^} configuration of the W_2_N_2_ core can be rationalized to arise from two low-spin W^II^ ions. The twisted conformation enables strong back bonding of each metal ion with one π*-MO of the N_2_ bridge, respectively, resulting in net transfer of approximately two electrons as judged from the Raman data. This picture is corroborated by DFT computations, which confirm the {π^8^*δ*^4^} configuration of the W_2_N_2_ core, analogous to the Mo analogue **4** and Cummins' **2^2+^**. The blue-shifted N_2_ stretching vibration of **6***vs.***4** (Δ*ν*_NN_ = 49 cm^–1^; Table 1) indicates slightly reduced back-bonding by the 5d metal.

The redox chemistry of **6** was examined to probe the electronic structure model. Cyclic voltammetry (CV) in THF shows no reduction feature down to –2.9 V (*vs.* FeCp_2_^+^/FeCp_2_). In contrast, two reversible oxidation waves (*E*o1 = –1.39 V; *E*o2 = –0.91 V) are observed. Both redox events are cathodically shifted by 250 mV with respect to the Mo analogue **4**, supporting metal centered oxidation. The oxidation products [(N_2_){WCl(PNP)}_2_]^+^ (**7^+^**) and [(N_2_){WCl(PNP)}_2_]^2+^ (**8^2+^**) could be isolated in yields beyond 80% upon chemical oxidation of **6** with one and two equivalents of silver salts, respectively (Scheme 3). Stabilization of **8^2+^** requires a weakly coordinating anion, which was introduced with Ag[Al(OC(CF_3_)_3_)_4_] as the oxidant.

In the solid state, **7^+^** and **8^2+^** resemble the twisted conformation found for **6** (Fig. 2). Distinctly different bond lengths around the two tungsten ions of the mixed-valent complex **7^+^** indicate valence localization, which is further supported in solution by the large comproportionation constant (*K*_c_ ≈ 10^8^)^24^ and the X-band EPR spectrum at r.t. The isotropic signal (*g*_av_ = 1.93) of the low-spin (*S* = 1/2) complex exhibits hyperfine interaction (HFI) with only one tungsten (*A*(^183^W) = 220 MHz) and two phosphorous nuclei (*A*(^31^P) = 56 MHz), respectively. HFIs with the N_2_-bridge are not found and the ^14^N_2_- and ^15^N_2_-isotopologues give identical spectra, further supporting metal centered oxidation. In fact, the degree of N_2_ activation is almost invariant within the redox series **6**/**7^+^**/**8^2+^** as judged from the invariance of the N–N stretching vibrations and the N–N bond lengths of the W_2_N_2_ cores (Table 1). Notably, the ^1^H NMR spectrum of **7^+^** features four signals assignable to *t*Bu groups, in agreement with the averaged *C*_2_ symmetry and therefore valence delocalization on the slow NMR timescale.

The double oxidation product **8^2+^** exhibits paramagnetically shifted, yet relatively sharp ^1^H NMR signals. Magnetic characterization by SQUID magnetometry reveals a *χ*_M_*T* product of about 0.6 cm^3^ mol^–1^ K^–1^ at r.t., which gradually drops to 0 at about 20 K. The data can be fitted to a model with two weakly antiferromagnetically coupled (*J* = –59 cm^–1^) low-spin (*S* = 1/2) ions. The *g*-value (*g*_av_ = 1.90) indicates an orbital contribution in the typical range for W^V^ complexes with multiply bound hard ligands, such as oxo or nitrido complexes.^25^

Characterization of the redox series **6**/**7^+^**/**8^2+^** supports the electronic structure picture with {π^8^δ^4^}/{π^8^δ^3^}/{π^8^δ^2^} configurations for the WIILS/WIILS (**6**), WIILS/WIIILS (**7^+^**) and WIIILS/WIIILS (**8^2+^**) complexes, respectively (Fig. 1 and Table 1). The spin orbitals of **7^+^** and **8^2+^** are orthogonal to the W_2_N_2_ core resulting in weak mutual coupling *via* the N_2_ bridge (*J*(**8^2+^**) = –59 cm^–1^). In consequence, all three complexes of the redox series exhibit strong back bonding to the N_2_ bridge with only weakly affected degrees of N_2_ activation. These interpretations are corroborated by DFT (ESI†). Doublet {π^8^*δ*_1_^2^*δ*_2_^1^} (**7^+^**) and open-shell singlet {π^8^*δ*_1_^1^*δ*_2_^1^} (**8^2+^**) ground states, respectively, were computed with a low lying triplet state for **8^2+^** due to weak antiferromagnetic coupling of the metal centered spins (*J*_DFT_ = –184 cm^–1^).

### Protonation induced N_2_ splitting *vs.* proton reduction

**Scheme 4:**
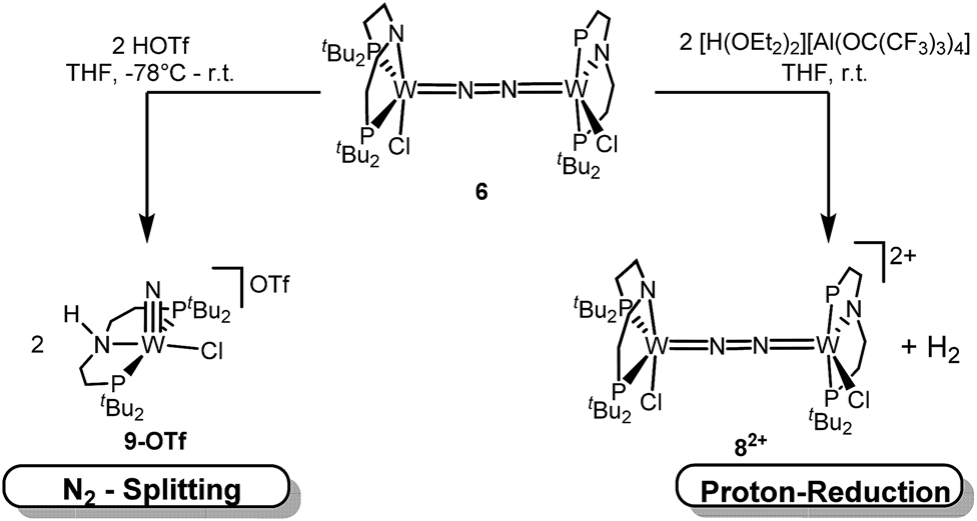
Protonation of **6** with 2 equiv. of different acids.

**Fig. 3:**
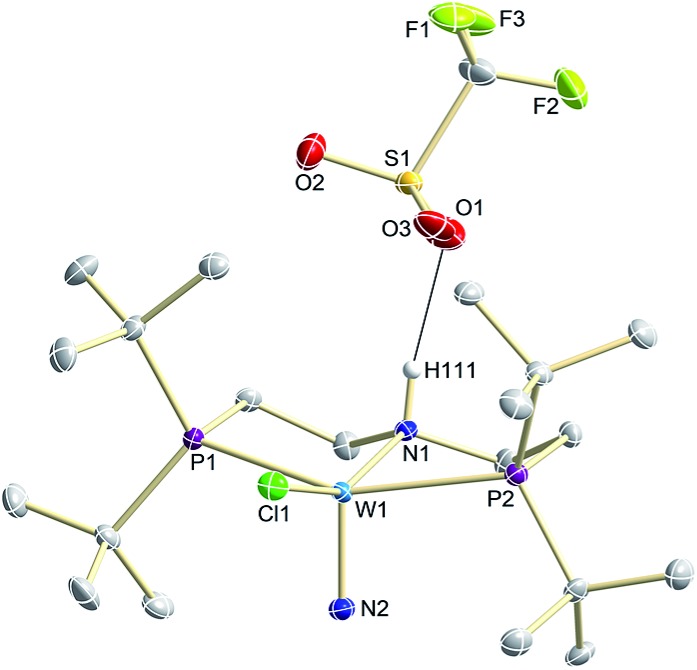
Molecular structure of **9^OTf^** in the crystal obtained by single crystal X-ray diffraction. Hydrogen atoms are omitted for clarity except H111.

N_2_ splitting of **6** into the nitride [W(N)Cl(*H*PNP)]OTf (**9^OTf^**, Scheme 4) as the only detectable tungsten species (NMR/EPR spectroscopy, HR-ESI-MS) was achieved upon adding 2 equiv. of triflic acid at –78 °C and gradual warming to r.t. Complex **9^OTf^** could be isolated in over 60% yield and was fully characterized. The tungsten(v) nitride is NMR silent and features an isotropic signal (*g*_av_ = 1.93) in the X-band EPR spectrum (THF, r.t.) with HFIs with the tungsten and phosphorous nuclei (*A*(^183^W) = 220 MHz; *A*(^31^P) = 56 MHz). The W≡N stretching vibration is found in the IR spectrum at 1058 cm^–1^ (**^15^N-9^OTf^**: Δ*ν* = 29 cm^–1^). In the solid state (Fig. 3), **9^+^** is isostructural with the molybdenum analogue,^19^ featuring square-pyramidally coordinated tungsten with the nitride ligand in the apical site. Hydrogen bonding of the amine proton with the triflate anion is indicated by the short NH^…^O distance (2.03(3) Å). The W≡N bond length (1.679(2) Å) is in the typical range found for the related tungsten nitrides.^1^,17a,^26^

In contrast to the Mo analogue **4** (Scheme 2), the selectivity of protonation induced N_2_ splitting strongly varies with the reaction conditions. The addition of HOTf (2 equiv.) to **6** at r.t. results in low nitride yields and substantial amounts of the oxidation products **7^+^** and **8^2+^**. Furthermore, 2 equiv. of strong acids with weakly coordinating anions, such as [H(OEt_2_)_2_] [Al(OC(CF_3_)_3_)_4_] and [H(OEt_2_)_2_]BAr^F^_24_ (BAr^F^_24_^–^ = B(C_6_H_3_-3,5-(CF_3_)_2_)_4_^–^), exclusively gave dicationic **8^2+^** both at low (–70 °C) and ambient temperatures (Scheme 4). Concomitant H_2_ evolution was confirmed by gas chromatography. Reaction of **6** at r.t. with 1 equiv. of these and other acids (HOTf, (2,6-lutidinium)OTf, [HNEt_3_][BAr^F^_24_], [H(OEt_2_)_2_][BAr^F^_24_], and [H(OEt_2_)_2_] [Al(OC(CF_3_)_3_)_4_]) selectively gives the oxidation product **7^+^** in all cases.

**Scheme 5:**
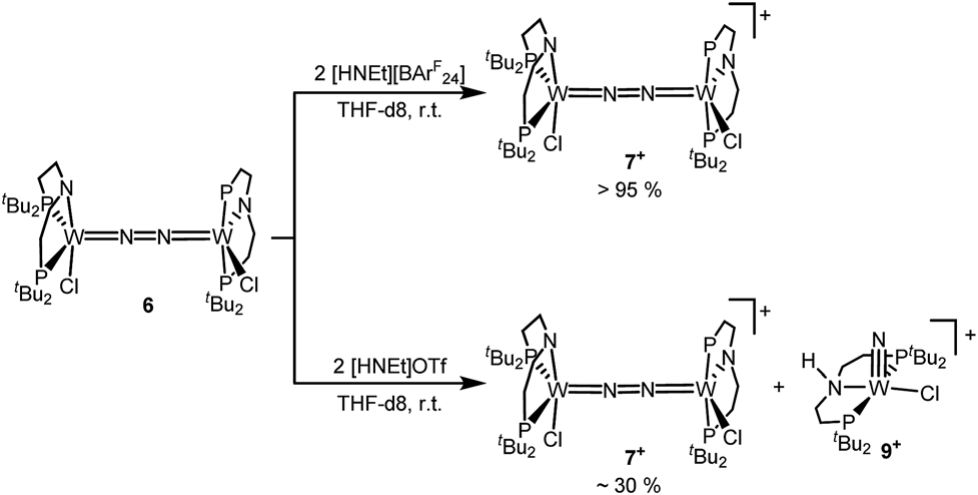
Anion dependent selectivity for the protonation of **6** with [HNEt_3_]X.

Next, the influence of the acid counteranion on the selectivity was probed. Upon protonation with [HNEt_3_][BAr^F^_24_] (2 equiv.), **7^+^** was found exclusively (Scheme 5). The second oxidation is hampered by the higher p*K*_a_ of this acid *vs.* HOTf, which prevents protonation of the monocationic product. Importantly, this selectivity changes with [HNEt_3_]OTf (2 equiv.): in this case, **7^+^** is obtained in spectroscopic yields of up to only 30%. *In situ* HR-ESI-MS examination indicates that nitride **9^+^** is formed as the only other product. This observation is reminiscent of acid dependent selectivities reported by Nishibayashi for catalytic nitrogen fixation (see above).^11^ For this reason, [HNEt_3_]Cl (2 equiv.) was also used. Unfortunately, sluggish mixtures of products were obtained, including substantial amounts of trichloride **5**. In the next sections, experimental and computational mechanistic examinations with only [HNEt_3_]X (X^–^ = BAr^F^_24_^–^, OTf^–^) are therefore reported.

### Mechanistic examinations

**Scheme 6:**
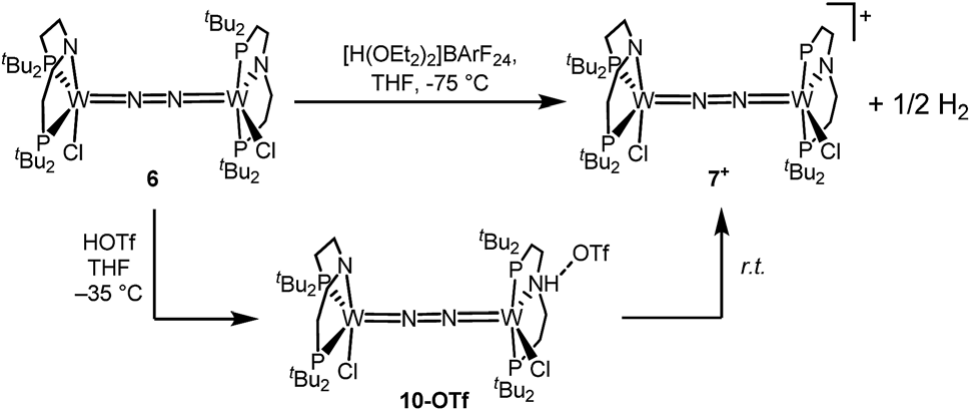
Oxidation of **6** with 1 equiv. of acid at different temperatures.

Stoichiometric protonation at low temperatures was carried out to obtain spectroscopic information about intermediates. With 1 equiv. of HOTf at low *T* (–35 °C), the NMR data are in agreement with pincer protonation to diamagnetic dinuclear *C*_1_-symmetric [(*H*PNP)ClW(μ-N_2_)WCl(PNP)]OTf (**10^OTf^**), analogous to the respective Mo system (Scheme 6).^19^

Notably, immediate formation of the oxidation product **7^+^** was observed with [H(OEt_2_)_2_][BAr^F^_24_], even at temperatures down to –75 °C. The enhanced stability of **10^OTf^** suggests an interaction of the immediate protonation product with the triflate anion. Contact-ion pair formation is confirmed by ^19^F and ^1^H DOSY NMR spectroscopy at –35 °C. The diffusion coefficient of the triflate anion in **10^OTf^** (*D* = 2.29 × 10^–6^ cm^2^ s^–1^) is in the same range as that of the cation (*D* = 2.18–2.14 × 10^–6^ cm^2^ s^–1^) and significantly reduced compared to free triflic acid (*D* = 5.11 × 10^–6^ cm^2^ s^–1^). We tentatively attribute the solution ion-pairing to hydrogen bonding of the triflate with the pincer N–H proton, as found in the solid state for **9^OTf^** (Fig. 3).

Protonation of **6** with 2 equiv. of HOTf at low temperatures in THF is associated with a color change from green to yellow. The absence of a signal in the ^31^P{^1^H} NMR spectrum and the broadened and strongly shifted ^1^H NMR signals indicate the formation of a paramagnetic product. The magnetic moment for the presumable product, [(N_2_){WCl(*H*PNP)}_2_]**^OTf2^** (**11^OTf2^**), was estimated with Evans' method at –60 °C (*μ*_eff_ = 4.7 *μ*_B_), *i.e.* close to the spin-only value for a quintet ground state (4.9 *μ*_B_). Increasing the temperature leads to fading of the color and disappearance of all ^1^H NMR signals, as expected for selective N_2_-splitting into the pale, NMR silent nitride product **9^OTf^**.

**Scheme 7:**
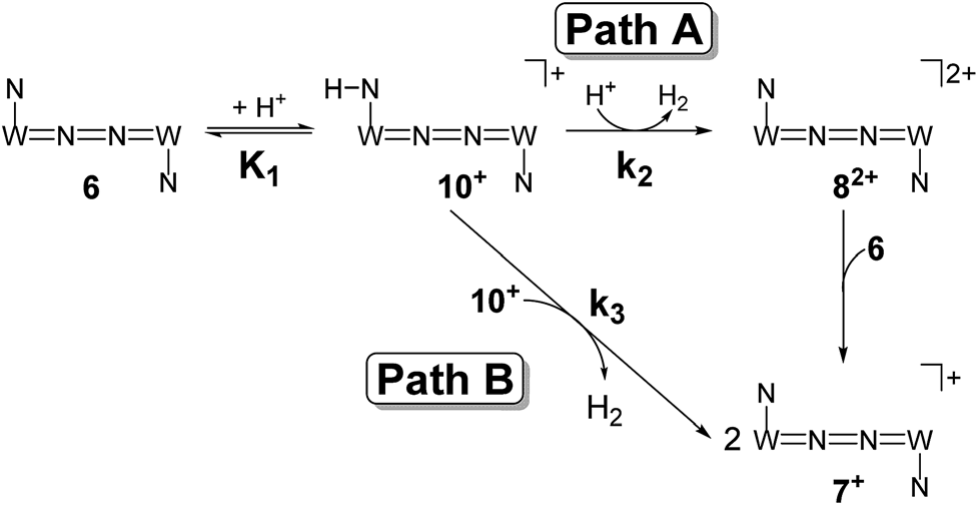
Proposed mechanistic pathways for proton reduction at high (Path A) and low (Path B) acid concentrations.

**Fig. 4:**
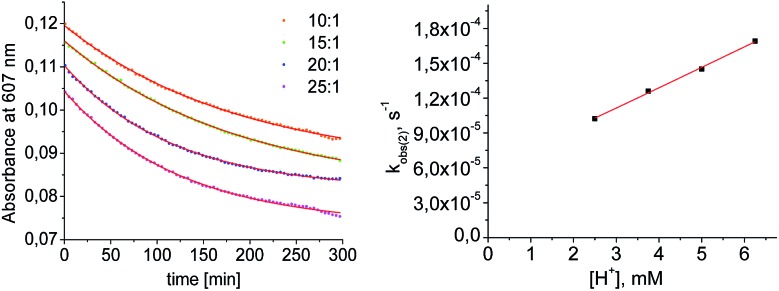
Left: plot of the absorbance at 607 nm *vs.* time for different concentrations of [HNEt_3_^+^]. Results from fitting to the rate law are indicated by red lines. Right: plot of *k*_obs(2)_*vs.* [HNEt_3_^+^].

Mechanistic information about proton reduction was obtained from kinetic studies. For this purpose, [HNEt_3_][BAr^F^_24_] was used as the acid, which selectively gives **7^+^** at r.t. within a convenient timescale even under pseudo first-order conditions. Addition of the acid to **6** in THF leads to an immediate drop of absorbance without significant change of the absorption maxima, suggesting only small changes in the electronic structure. The acid concentration dependence of the absorbance allowed for estimating the equilibrium constant and forward rate of the initial protonation of **6** (*K*_1_ = 1592 ± 578 M^–1^, *k*_1_ = 163 ± 47 M^–1^ s^–1^; Scheme 7 and Fig. S25 and S26 in the ESI†). This step is followed by a much slower, mono-exponential decay, which was monitored over 5 h (Fig. 4, left). Under pseudo first order conditions in acid (*c*(HNEt_3_^+^)_0_/*c*(**6**)_0_ = 10–25), the rate constant (*k*_obs(2)_) linearly depends on the acid concentration (Fig. 4, right), which is in agreement with a slow, irreversible second protonation after the initial, fast pre-equilibrium *K*_1_. However, the non-zero intercept indicates the presence of at least one competitive pathway at a low acid concentration. The rate constant *k*_obs(2)_ for the formation of **7^+^** was therefore expressed as eqn (1) which results from the minimum kinetic model outlined in Scheme 7:
1

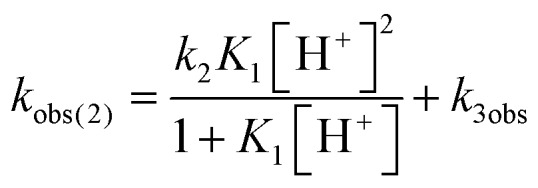




The first term accounts for the initial protonation of **6** to give **10^+^**, followed by irreversible H_2_ release from acid and **10^+^**. Rapid, subsequent comproportionation of the resulting **8^2+^** with **6** to 2 × **7^+^** is in line with the electrochemical results (*K*_c_ ≈ 10^8^, see above). The second term in eqn (1) is ascribed to bimolecular decay of **10^+^** as an alternative path at low acid concentrations. The rate constant *k*_2_ = 0.018 ± 0.001 M^–1^ s^–1^ was derived from fitting the experimental data to eqn (1) (with preserved *K*_1_) under pseudo first order conditions in acid (10–25 equiv.). The rate constant *k*_3_ = 0.4 M^–1^ s^–1^ for the bimolecular path at low acid concentrations was obtained from the initial rate of the reaction of **6** and an equimolar amount of [HNEt_3_][BAr^F^_24_].‡
‡An alternative pathway *via* reduction of **10^+^** with **6** is less likely based on the derived rate constant 
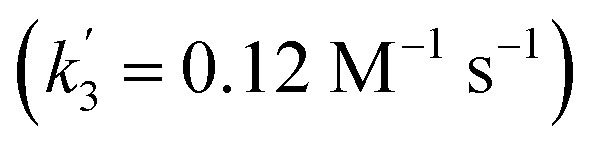
, which is considerably smaller than *k*_3_.


Kinetic analysis suggests two pathways for H_2_ formation which both go through the spectroscopically characterized common intermediate **10^+^** (as **10^OTf^**). Path B (Scheme 7) explains the decay of **10^+^** even in the absence of the acid and reflects a bimolecular H_2_ formation route as proposed by Matson and Peters for an iron diazenide N_2_-fixation intermediate.^8^ However, path A is predominant with excess acid. Besides these routes for hydrogen evolution, splitting of the N_2_ bridge is observed in the presence of triflate as the counteranion and is even selective at lower temperatures. These effects are rationalized computationally in the next section.

### Computational examinations

**Scheme 8:**
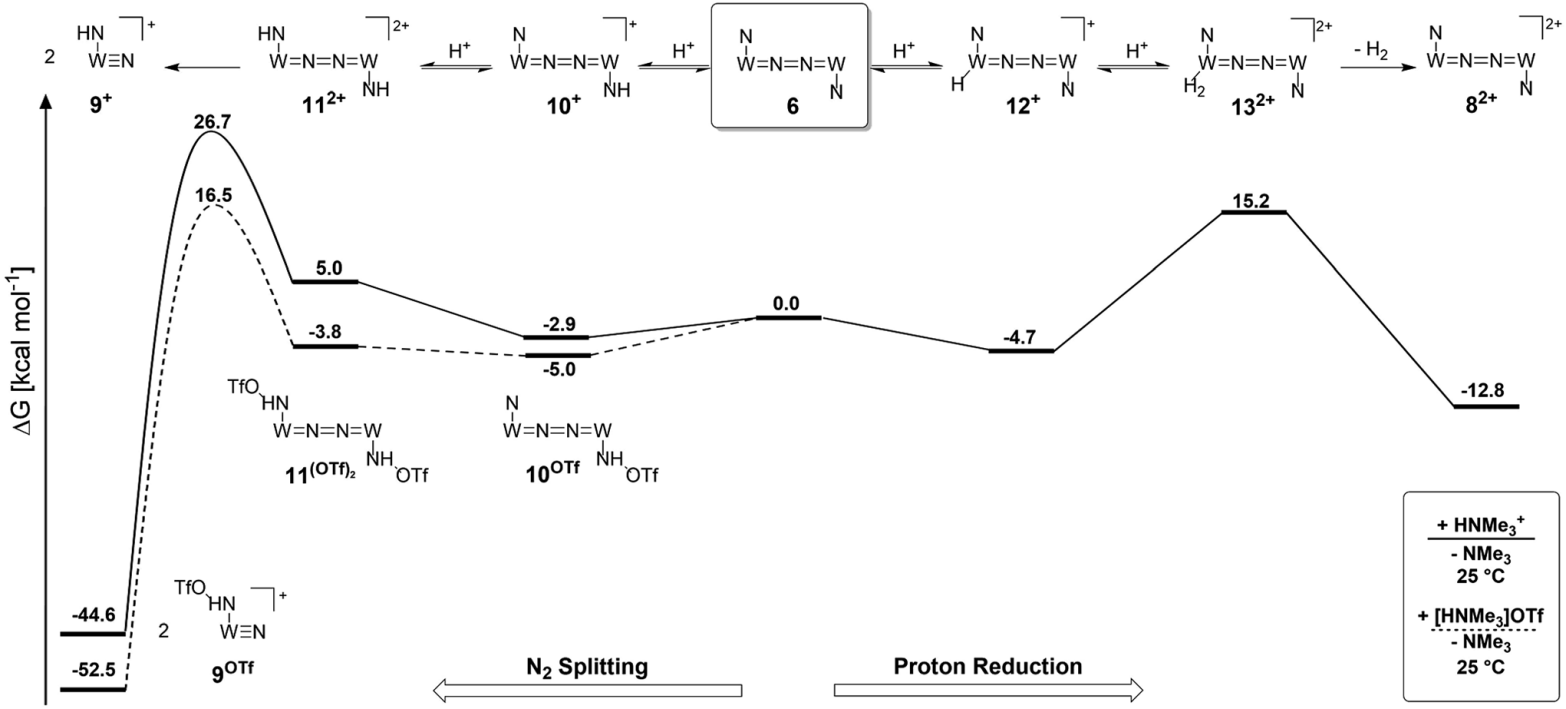
Computed energy profile for protonation induced N_2_ splitting (left branch) and hydrogen evolution *via* Path A (right branch) *via* double protonation of the dinitrogen complex **6** at room temperature in the absence (solid line) and presence (dashed line) of triflate as the counteranion.

Protonation with [NEt_3_H][BAr^F^_24_] was first examined computationally with trimethylammonium as the model acid (Scheme 8). Two different sites, a metal ion and a pincer nitrogen atom, respectively, were considered for the first protonation step. A hydride product [(PNP)W(H)Cl(μ-N_2_)WCl(PNP)]^+^ (**12^+^**) adopts an electronic singlet (*S* = 0) ground state and was found to be the global protonation minimum at 
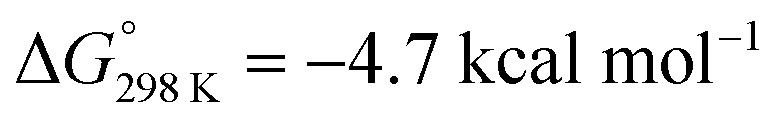
 below **6** and [NMe_3_H]^+^. Hence, the model computation is in excellent agreement with the experimental equilibrium constant *K*_1_. The computed structure of **12^+^** features a bridging hydride between the metal ion and a pincer phosphorous atom. A similar structure was previously found experimentally by Schrock and co-workers for the protonation of a PCP molybdenum(iv) nitride by [NEt_3_H][BAr^F^_24_].^27^ All efforts to experimentally verify hydride intermediates like **12^+^** were unfortunately unsuccessful. However, pincer protonation to **10^+^** is only slightly less exergonic 
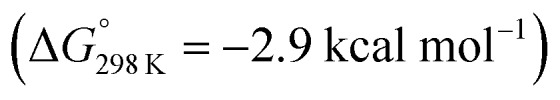
. Importantly, this state is further stabilized upon use of [NEt_3_H]OTf as the acid due to hydrogen bonding of the pincer amine moiety with the triflate anion by 
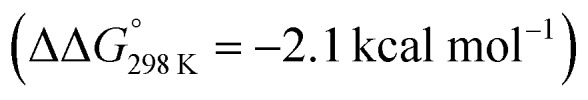
. In contrast, the hydride ligand is not involved in hydrogen bonding, rendering **10^OTf^**
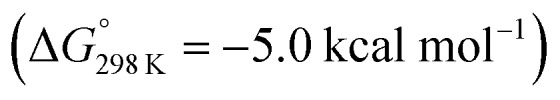
 the global minimum of the first protonation in the presence of triflate. Overall, the metal and pincer protonation products **12^+^** and **10^+^** (and **10^OTf^** in the presence of triflate) should be in rapid equilibrium under these conditions, which is slightly shifted towards pincer protonation by hydrogen bonding with the counteranion. Notably, hydrogen bonding with the conjugate base NMe_3_ was not observed, presumably for steric reasons.

Starting from the amine/hydride equilibrium, the second protonation with [NMe_3_H]^+^ can ultimately lead to hydrogen evolution or N_2_ splitting, respectively. The formation of H_2_ and dicationic **8^2+^**, which represents Path A (Scheme 7), was computed to be exergonic by 
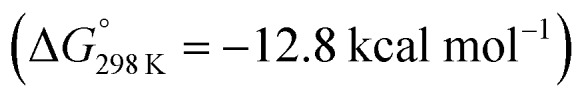
 with respect to **6**. The most reasonable pathway (Scheme 8, right branch) proceeds *via* hydride protonation of **12^+^** leading to the dihydrogen intermediate [(PNP)W(H_2_)Cl(μ-N_2_)WCl(PNP)]^2+^ (**13^2+^**), which is unstable and readily releases H_2_ without barriers. While the transition state that leads from **12^+^** to **13^2+^** could not be reliably located due to the flat potential energy profile of protonation, the free energy of **13^2+^** (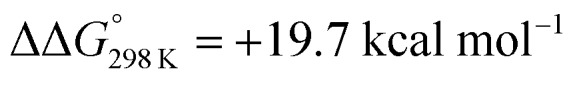 with respect to **12^+^**) was used as an estimate for the kinetic barrier of hydride protonolysis.§
§A transition state for the direct protonation of **12^+^** with HNMe_3_^+^ at the hydride ligand was located at 25.4 kcal mol^–1^ above **6** and two equivalents of [HNMe_3_]^+^. However, this activation free energy is considerably higher than the experimentally determined value (Δ*G*‡eff = 19 kcal mol^–1^). It is not clear if this deviation can be attributed to the computational truncation of the acid or to a lower competing pathway. We therefore prefer to use the experimental value for the discussion of the selectivity. See also the ESI for further discussion.†
 Notably, this value is in excellent agreement with the experimentally derived barrier for Path A (*k*_2_ = 0.018 M^–1^ s^–1^; Δ*G*‡eff = 19 kcal mol^–1^).

Alternatively, splitting of the dinitrogen bridge (Scheme 8, left branch) was computed to proceed *via* protonation of the second pincer nitrogen. In the absence of triflate, [(N_2_){WCl(*H*PNP)}_2_]^2+^ (**11^2+^**) was located at 
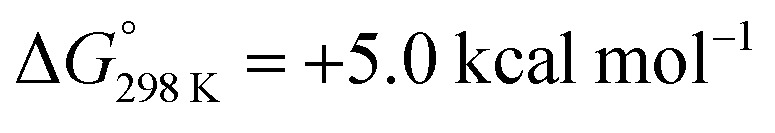
 *vs.***6** (
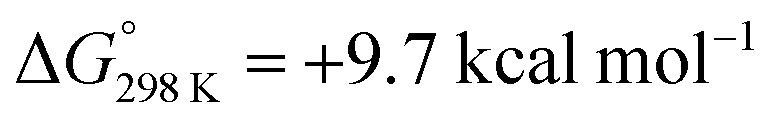
 *vs.* the global first protonation minimum **12^+^**) adopting an electronic quintet (*S* = 2) ground state in accordance with the experimental findings for **11^OTf2^**. From there, N_2_ cleavage into the nitrides **9^+^** was computed to be strongly exergonic 
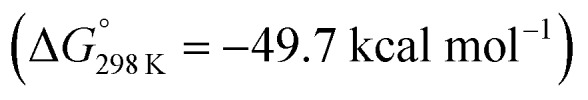
 with a kinetic barrier (Δ*G*‡298 K = 21.7 kcal mol^–1^) that is comparable to the experimentally derived barriers for [(N_2_){MoCl(*H*PNP)}_2_]^2+^ (Δ*G*‡298 K = 19.5 kcal mol^–1^) and [(N_2_){ReCl(PNP)}_2_] (Δ*G*‡298 K = 19.8 kcal mol^–1^), respectively. For the tungsten system, this gives rise to an overall effective barrier for protonation induced N_2_ splitting from the most stable monoprotonation intermediate, hydride **12^+^**, of Δ*G*‡eff = 31.4 kcal mol^–1^. This value is considerably higher than the estimate for the hydrogen evolution pathway (ΔΔ*G*‡eff = +11.5 kcal mol^–1^), which is in line with selective proton reduction with [NEt_3_H][BAr^F^_24_] as the acid.

Importantly, the relative energetics of these two reaction channels are perturbed in the presence of triflate as the counteranion. As was found for the first pincer protonation (see above), triflate hydrogen bonding stabilizes the pincer diprotonation product **11^OTf2^** by –8.8 kcal mol^–1^. Consequently, the estimated effective barrier for hydrogen evolution (Δ*G*‡298 K = 20.2 kcal mol^–1^*vs.* the global first protonation minimum in the presence of triflate **10^OTf^**) is slightly raised. On the other hand, the N_2_ splitting pathway (Δ*G*‡298 K = 21.5 kcal mol^–1^*vs.***10^OTf^**) is almost isoenergetic, in full agreement with the experimental findings. The triflate induced effect on selectivity is therefore attributed to Curtin–Hammett controlled reactivity wherein N–H hydrogen bonding to the counteranion modifies the energetics of the protonation pre-equilibria.

A similar picture evolves for the reaction with triflic acid (see the ESI, Scheme S1†). However, the potential energy of protonation is augmented by the higher driving force with the stronger acid (p*K*THFa(Et_3_NH^+^) – p*K*THFa(HOTf) = 4.7).^28^ This affects the selectivity as the effective barrier for the N_2_ splitting branch *versus* hydrogen evolution is close in energy. Furthermore, all rate determining states are below the starting point **6**. In consequence, under these conditions (HOTf as the acid at r.t.), Curtin–Hammett conditions do not apply resulting in the experimentally observed low selectivity.

Reduction of the temperature to –80 °C further perturbs the relative energetics of the two reaction pathways with HOTf. The computed amine 
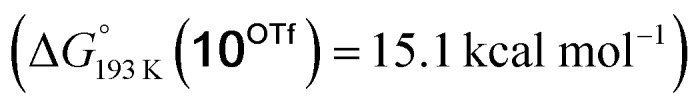
 *vs.* hydride 
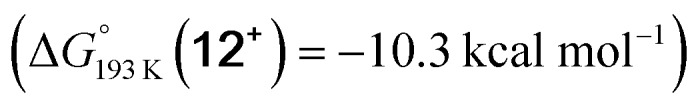
 equilibrium is even more shifted towards the amine due to the lower entropic penalty for hydrogen bonding at low *T*. The negligible population of the hydride tautomer is in agreement with the exclusive experimental observation of **10^OTf^** and **11^OTf2^** upon single and double protonation with HOTf at –80 °C. From **11^OTf2^**, the dihydrogen complex **13^2+^**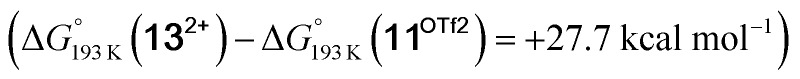 is much higher in free energy than the barrier for N_2_ splitting (Δ*G*‡193 K = 19.9 kcal mol^–1^), in line with selective N_2_ splitting upon double protonation with HOTf at –80 °C and slow warming.

## Concluding remarks

In summary, an anion effect on the selectivity of proton induced dinitrogen splitting (NR) *vs.* hydrogen evolution (HR) at the N_2_ bridged ditungsten complex **6** was demonstrated and rationalized. Our spectroscopic, kinetic and computational studies suggest some guidelines to improve NR over HR yields:

(a) Nitrogen *vs.* metal protonation offers separate reaction channels with a proposed hydride isomer leading to hydrogen evolution analogous to the highly active Mo-oxo polypyridyl HR catalysts.^29^ The tautomerisation equilibrium can be offset by hydrogen bonding with protic N–H hydrogen atoms favoring the use of an acid [BH]^+^X^–^ where the anion X^–^ is prone to form H-bonds for high NR selectivity.

(b) Protonation under Curtin–Hammett control with weak acids can become irreversible with strong acids. Hence, the p*K*_a_ of the acid can have a decisive kinetic effect on the selectivity.

(c) Lower temperatures favour hydrogen bonding interactions due to the reduced entropic penalty as a strategy for increased NR yields.

Besides the immediate application to the current system, these findings might be considered as a model reaction for nitrogen fixation schemes. The studies of Peters and of Nishibayashi have emphasized the importance of proton coupled electron transfer for N_2_ fixation under ambient conditions. Our kinetic model might therefore offer some general strategies regarding the choice of acid to improve NR selectivities with respect to unproductive proton reduction.

## Conflicts of interest

There are no conflicts to declare.

## Supplementary Material

Supplementary information

Crystal structure data
