# Supplementary material for: Selectivity of tungsten mediated dinitrogen splitting vs. proton reduction
Source: Chem Sci. 2019 Sep 24;10(44):10275–82. doi: 10.1039/c9sc03779a (PMC6984443; doi:10.1039/c9sc03779a)
Supplement: Supplementary file 1 [file SC-010-C9SC03779A-s001.pdf]

Electronic Supplementary Material (ESI) for Chemical Science.
This journal is © The Royal Society of Chemistry 2019

Supporting Information

1. Experimental Details **3**

1.1 Materials and synthetic methods 3

1.2 Analytical Methods 3

1.3 Syntheses 4

[WCl_3(PNP)] (**5**) 4

[(N_2){WCl(PNP)}$_2$] (**6**) 4

[(N_2){WCl(PNP)}$_2$]$^+$ (**7**$^+$) 4

[(N_2){WCl(PNP)}$_2$]$^{2+}$ (**8**$^{2+}$) 5

[W(N)Cl(^{H}PNP)]OTf (**9**^{OTf}) 5

Monoprotonation of **6** giving [(^{H}PNP)ClW(N_2)WCl(PNP)]OTf (**10**^{OTf}) 6

Doubleprotonation of **6** giving [{(^{H}PNP)ClW}$_2${μ-N_2}](OTf)$_2$ (**12**^{OTf2}) 6

1.4 Kinetic Analysis 6

K_1 determination 6

k_2 and k_3 determination 6

k_3 determination via initial rate method 6

2. Spectroscopic Results **7**

2.1 Characterization 7

[WCl_3(PNP)] (**5**) 7

[(N_2){WCl(PNP)}$_2$] (**6**) 8

[(N_2){WCl(PNP)}$_2$]$^+$ (**7**$^+$) 11

[(N_2){WCl(PNP)}$_2$]$^{2+}$ (**8**$^{2+}$) 14

[W(N)Cl(^{H}PNP)]OTf (**9**^{OTf}) 16

Monoprotonation of [(N_2){WCl(PNP)}$_2$] (**10**^{OTf}) 17

Double protonation of [(N_2){WCl(PNP)}$_2$] (**11**$^{(OTf)2}$) 20

2.2 Kinetic Analysis 21

Exemplary UVvis spectrum 21

K_1 determination 22

k_2 and k_3 determination 23

3. Crystallographic Details **24**

Crystal Structure of **5** 25

Crystal Structure of **6** 28

Crystal Structure of **7-OTf** 32

Crystal Structure of **8-(BPh_4)$_2$** 37

Crystal Structure of **9-OTf** 42

4. DFT-Calculations **46**

4.1 Computational Details 46
4.2 Thermodynamics of protonation 47
4.3 Computed reaction energies and electronic ground states 48
4.3.1 Computed reaction profile with HOTf 48
4.3.2 Structural, electronic and spectroscopic properties of **6**, **7**$^{+}$ and **8**$^{2+}$ 49
4.3.3 First protonation step 53
4.3.4 Second protonation step, nitrogen splitting and hydrogen formation 54
4.4 Kinetics of protonation 55
4.4.1 Hydrogen evaluation reaction 55
4.4.2 Formation of **11**$^{(OTf)2}$ from **10**^{OTf} and HOTf 56
4.5 Structures 58
References 84

1. Experimental Details

1.1 Materials and synthetic methods

All experiments were carried out under inert conditions using standard Schlenk and glove-box techniques (argon atmosphere). All solvents were purchased in HPLC quality (Sigma Aldrich) and dried using an MBRAUN Solvent Purification System, with exception of chlorobenzene, which was dried over CaH. THF was additionally dried over Na/K-alloy. Deuterated solvents were obtained from Euriso-Top GmbH and dried over Na/K (C_6D_6, d_8-THF), distilled by trap-to-trap transfer *in vacuo*, and degassed by three *freeze-pump-thaw* cycles, respectively. NEt_3 was dried over KOH and distilled under Ar-atmosphere. Silica gel 60 silanized was purchased from Merck KGaA and heated at 120 °C *in vacuo* for 5 days prior to use.

WCl_4, $^{15}N_2$, AgOTf (Sigma Aldrich), $Ag(Al(OC(CF_3)_3)_4)$ (iolitec) and HOTf (ABCR GmbH) were used as purchased. $[H(OEt_2)_2][BArF_{24}]$ ($BArF_{24}^- = B(C_6H_3\text{-}3,5\text{-}(CF_3)_2)_4^-$)[1], $[H(OEt_2)_2][Al(OC(CF_3)_3)_4]$[2], $AgBPh_4$[3] and $HN(CH_2CH_2P^tBu_2)_2$[4] were prepared according to published procedures.

1.2 Analytical Methods

NMR spectra were recorded on Bruker Avance III 300 or Avance III 400 spectrometers or an Avance 500 spectrometer with a Prodigy broadband cryoprobe, respectively, and calibrated to the residual solvent signals (C_6D_6: δ_H = 7.16 ppm, δ_C = 128.4 ppm; d_8-THF: δ_H = 3.58 ppm, δ_C = 67.6 ppm). ^{31}P and ^{15}N NMR chemical shifts are reported relative to external phosphoric acid and nitromethane (δ = 0.0 ppm), respectively. Signal multiplicities are abbreviated as: s (singlet), d (doublet), m (multiplet), br (broad).

Elemental analyses were obtained from the Analytisches Labor, Georg-August-Universität (Göttingen, Germany) using an Elementar Vario EL 3 analyzer.

HR-ESI-MS (Bruker maXis QTOF) and LIFDI-MS (JEOL AccuTOF JMS-T100GCV) spectra were measured by the Zentrale Massenabteilung, Fakultät für Chemie, Georg-August-Universität Göttingen.

All Resonance Raman spectra except for **8**$^{2+}$ were recorded using a HORIBA Scientific LabRAM HR 800 spectrometer with open-electrode CCD detector in combination with a free space optical microscope and a He:Ne-laser (632.8 nm). The Resonance Raman spectra for **8**$^{2+}$ were recorded using a Triple Raman Spectrometer TR 557 from S&I (Spectroscopy & Imaging GmbH).

Cyclic voltammograms were measured with a METROHM Autolab PGSTAT101 in a 0.1 M $[NnBu_4][PF_6]^-$ solution with glassy carbon working electrode, Pt counter electrode and Ag/Ag^+ reference electrode. Original spectra were referenced against the $[Fe(C_5H_5)_2]^{0/+}$ couple.

Experimental X-band EPR spectra were recorded on a Bruker ELEXSYS-II E500 CW-EPR. Simulations were carried out using the program QPOW,[5] as modified by J. Telser.

Magnetic moments were determined by Evans' method as modified by Sur and corrected for diamagnetic contributions.[6] Magnetic susceptibility measurements were performed with a Quantum Design MPMS-XL-5 SQUID magnetometer in the temperature range from 295 to 2 K at 0.5 T applied field. The powdered sample was contained in a Teflon bucket and fixed in a non-magnetic sample holder. Each raw data point for the measured magnetic moment of the sample was corrected for the diamagnetic contribution by subtraction of the experimentally determined magnetic measurement of the Teflon bucket. The molar susceptibility data were corrected for the diamagnetic contribution using the Pascal constants and the increment method according to Haberditzel.[7] Experimental data were modelled with the julX program.[8]

The H_2 was detected by a Shimadzu GC-2014 gas chromatograph equipped with a TCD detector and a ShinCarbon ST 80/100 Silco column. IR spectra were recorded using a Bruker ALPHA FT-IR spectrometer with Platinum ATR module.

UVvis spectra were recorded on an Agilent Cary 60 equipped with an Unisoku Cryostat (CoolSpek) and magnetic stirrer using quartz cuvettes with an attached tube and a screw cap with a septum (K_1-determination) or a J-Young-cap (k_2 and k_3 determination). All UVvis samples were prepared in a glovebox and transferred out of the glovebox prior to the measurement. For the determination of K_1 the amount of acid solution was added with a Hamilton syringe directly while measuring.

1.3 Syntheses

$[WCl_3(PNP)]$ (**5**)

WCl_4 (509 mg, 1.54 mmol, 1.1 eq) is added to a mixture of Et_3N (0.4 mL, 288 mg, 2.85 mmol, 2.1 eq) and $HN(CH_2CH_2P^tBu_2)_2$ (500 mg, 1.38 mmol, 1.0 eq) in benzene (50 mL) and stirred for 5 d at 90 °C. The solvent is removed *in vacuo* and the crude product extracted with benzene (4x 15 mL) and filtered through celite. After evaporation of the solvent, the residue is taken up in THF (~4 mL), layered with pentane (~20 mL) and stored at -40 °C for 2 d. The obtained dark crystals are washed with cold pentane (2x 2mL), taken up in benzene and lyophilizied over night to give **5** as a yellow powder (540 mg, 60 %).
Crystals suitable for X-ray diffraction were obtained by diffusion of pentane into a saturated dichloromethane solution at -40 °C.
^{1}H NMR (C_6D_6. 300 MHz, [ppm]): δ = 9.13 (s_{br}, C*H$_2$*), 0.82 (s_{br}, ^{t}Bu), -139.5 (s_{br}, C*H$_2$*).
Elem. Anal. found (calc) for $C_{20}H_{44}Cl_3NP_2W$: C, 36.85 (36.92); H, 6.93 (6.82); N, 2.16 (2.15).
μ_{eff} = 2.8 ± 0.1 μ_B.

$[(N_2)\{WCl(PNP)\}_2]$ (**6**)

5 (250 mg, 0.384 mmol, 1.0 eq) and NaHg (11.5 g, 0.845 mmol, 2.2 eq) are stirred in THF (10 mL) for 2 h under an N_2 atmosphere at 0 °C to give a color change from yellow to dark green. After extraction with THF (3x 8 mL) the solvent is removed *in vacuo.* The residue is washed with pentane (5x 8 mL) and extracted with benzene (5x 8 mL). **6** is obtained as a green powder after column chromatography over Silica gel 60 silanized with benzene as eluent and lyophilisation out of benzene (150 mg, 66%).
Crystals suitable for X-ray diffraction were obtained from diffusion of pentane into a THF-solution at -40 °C.
$^{15}N_2$ labeled **^{15}N-6** was synthesized analogously under a $^{15}N_2$-atmosphere.
^{1}H NMR (THF-d_8. 500 MHz, [ppm]): δ = 1.13 (d, $^3J_{HP}$ = 12.8 Hz, 36 H, 4 ^{t}Bu), 1.16 (d, $^3J_{HP}$ = 13.9 Hz, 18 H, 2 ^{t}Bu), 1.35 (m, 2 H, PCH*H*), 1.55 (d, $^3J_{HP}$ = 12.8 Hz, 18 H, 2 ^{t}Bu), 1.74 (m, 2 H, PCH*H*), 2.29 (m, 2 H, PCH*H*), 2.39 (m, 2 H, PCH*H*), 3.54 (m, 4 H, NC*H$_2$*), 3.66 (m, 4 H, NC*H$_2$*).
^{13}C{^{1}H} NMR (THF-d_8. 126 MHz, [ppm]): δ = 30.71 (d, $^2J_{CP}$ = 4.3 Hz, 2 PC(*C*H$_3$)$_3$), 30.8 (d, $^2J_{CP}$ = 5.7 Hz, 2 PC(*C*H$_3$)$_3$), 30.8 (d, $^2J_{CP}$ = 3.9 Hz, 2 PC(*C*H$_3$)$_3$), 31.6 (d, $^1J_{CP}$ = 7.35 Hz, 2 P*C*H$_2$), 31.8 (d, $^1J_{CP}$ = 7.62 Hz, 2 P*C*H$_2$), 32.0 (d, $^2J_{CP}$ = 4.9 Hz, 2 PC(*C*H$_3$)$_3$), 38.5-38.9 (m, 8 P*C*(CH$_3$)$_3$), 78.9 (s, 2 N*C*H$_2$), 79.2 (s, 2 N*C*H$_2$). **^{15}N{^{1}H} NMR** (THF-d_8. 50.7 MHz, [ppm]): δ = 31.1 (s) **^{31}P{^{1}H] NMR** (THF-d_8. 162 MHz,[ppm]): δ = 92.9 (d, $^2J_{PP}$ = 147.4 Hz), 87.8 (d, $^2J_{PP}$ = 147.4 Hz).
Elem. Anal. found (calc) for $C_{40}H_{88}Cl_2N_4P_4W_2$: C 40.39 (40.45); H 7.27 (7.47); N 2.69 (4.72). The lower nitrogen content is attributed to N_2-loss of highly sensitive **6** during manipulation and/or measurement. The same observation was made for the analogous Mo compound.[9]
rRaman (λ_{ex} = 457nm, frozen THF-d_8, [cm^{-1}]): **^{14}N-6** 1392 (ν_{NN}); **^{15}N-6** 1347 (ν_{NN}).

$[(N_2)\{WCl(PNP)\}_2]^+$ (**7$^+$**)

Oxidation Route:
Ag[BPh$_4$] (14.4 mg, 33.7 µmol, 1.0 eq) is added to a solution of **6** (40.0 mg, 33.7 µmol, 1.0 eq) in THF (5 mL) leading to a direct color change from green to dark brown. After stirring for 1 h the mixture is filtered over celite, dried *in vacuo*, washed with pentane (3x 4 mL) and extracted with THF (2x 4 mL). After removal of the solvent *in vacuo* $[\{(PNP)ClW\}_2\{N_2\}][BPh_4]$ is obtained as a dark brown solid (43 mg, 85 %).
Crystals suitable for X-ray diffraction were obtained by diffusion of pentane into a saturated benzene solution.
^{1}H NMR (THF-d_8. 300 MHz, [ppm]): δ = 7.26 (m, BPh$_4$), 6.85 (m, BPh$_4$), 6.71 (m, BPh$_4$), 4.37 (s_{br}, CH*H*), 3.13 (s_{br}, ^{t}Bu), 2.99 (s_{br}, ^{t}Bu), 2.51 (s_{br}, ^{t}Bu), 2.38 (s_{br}, ^{t}Bu), -5.60 (s_{br}, 2 CH*H*), -14.14 (s_{br}, CH*H*), -22.06 (s_{br}, CH*H*), -25.1 (s_{br}, CH*H*), -27.8 (s_{br}, CH*H*).
Elem. Anal. found (calc) for $C_{64}H_{108}BCl_2N_4P_4W_2$: C, 51.52 (51.01); H, 7.17 (7.22); N 3.29 (3.72).
rRaman (λ_{ex} = 457nm, frozen THF-d_8, [cm^{-1}]): **^{14}N-7$^+$** 1414 (ν_{NN}); **^{15}N-7$^+$** 1360 (ν_{NN}).

Protonation Route:

$[H(Et_2O)_2][BAr^F_{24}]$ (31 mg, 30.6 µmol, 1.0 eq) is added to a solution of **6** (40.0 mg, 33.7 µmol, 1.1 eq) in Et_2O (8 mL) resulting in a direct color change from green to dark brown. After stirring for 1 h the mixture is filtered, dried *in vacuo* and washed with pentane (3x 10 mL). The crude product is extracted with Et_2O, filtered over celite and dried *in vacuo* to give **7⁺** as a brown solid (47 mg, 75 %).

^{1}H NMR (THF-d_8, 300 MHz, [ppm]): δ = 7.79 (s, BAr^F_{24}), 7.57 (s, BAr^F_{24}), 4.43 (s_{br}, CH*H*), 3.26 (s_{br}, ^{t}Bu), 3.12 (s_{br}, ^{t}Bu), 2.60 (s_{br}, ^{t}Bu), 2.46 (s_{br}, ^{t}Bu), -5.56 (s_{br}, CH*H*), -5.75 (s_{br}, CH*H*), -14.5 (s_{br}, CH*H*), -22.8 (s_{br}, CH*H*), -25.5 (s_{br}, CH*H*), -28.4 (s_{br}, CH*H*).

Elem. Anal. found (calc) for $C_{72}H_{100}BCl_2F_{24}N_4P_4W_2$: C, 41.82 (42.17); H, 4.75 (4.91); N 2.46 (2.73).

HR-ESI-MS found (calc) for $[C_{40}H_{88}Cl_2N_4P_4W_2]^+$: 1186.4347 (1186.4339), 1188.4368 (1188.4380).

$[(N_2)\{WCl(PNP)\}_2]^{2+}$ (**8^{2+}**)

Oxidation Route:

$Ag(Al(C(CF_3)_3)_4)$ (35.3 mg, 32.8 µmol, 2.0 eq) is added to a solution of **6** (20.0 mg, 16.8 µmol, 1.0 eq) in PhCl (8 mL). The solution is stirred under exclusion of light for 12 h. Afterwards the precipitate is filtered off, washed with benzene (6 ml) and extracted with dichloromethane (16 mL). The solution is concentrated *in vacuo*, layered with pentane and stored at -40 °C for 3 d. The resulting brown precipitate is decanted, washed with pentane (2x 2 mL), extracted with THF and dried *in vacuo* to give **8^{2+}** as a brown solid (39.3 mg, 75 %).

The synthesis can also be carried out using $AgBPh_4$ as oxidant in THF. After work-up, crystals suitable for X-ray diffraction could be obtained by diffusion of pentane into a saturated THF solution.

^{1}H NMR (THF-d_8, 400 MHz, [ppm]): δ = 4.32 (s_{br}, ^{t}Bu), 4.19 (s_{br}, ^{t}Bu), 3.78 (s_{br}, ^{t}Bu), 3.49 (s_{br}, ^{t}Bu), -1.56 (s_{br}, CH*H*), -7.03 (s_{br}, CH*H*), -8.81 (s_{br}, CH*H*), -8.99 (s_{br}, CH*H*), -32.1 (s_{br}, CH*H*), -46.9 (s_{br}, CH*H*), -56.6 (s_{br}, CH*H*), -62.0 (s_{br}, CH*H*).

Elem. Anal. found (calc) for $C_{72}H_{88}Al_2Cl_2F_{72}N_4O_8P_4W_2$: C 29.2 (29.4); H 3.30 (3.01); N 1.66 (1.90).

rRaman (λ_{ex} = 514.5 nm, frozen THF-d_8, [cm^{-1}]): **^{14}N-8^{2+}** 1400 (ν_{NN}); **^{15}N-8^{2+}** 1356 (ν_{NN}).

Protonation Route:

A solution of $[H(OEt_2)_2][Al(O(C(CF_3)_3)_4]$ (9.9 mg, 8.84 µmol, 2.1 eq) in Et_2O (2 mL) is added dropwise to a suspension of **6** (5.0 mg, 4.21 µmol, 1.0 eq) in Et_2O (5 mL). After stirring over night all volatiles are removed *in vacuo* and the remaining yellowish brown residue is taken up in THF-d_8 (90 % spectroscopic yield).

^{1}H NMR (THF-d_8, 300 MHz, [ppm]): δ = 4.25 (s_{br}, ^{t}Bu), 4.14 (s_{br}, ^{t}Bu), 3.69 (s_{br}, ^{t}Bu), 3.42 (s_{br}, ^{t}Bu), -1.24 (s_{br}, CH*H*), -6.60 (s_{br}, CH*H*), -8.73 (s_{br}, 2x CH*H*), -31.2 (s_{br}, CH*H*), -45.9 (s_{br}, CH*H*), -55.5 (s_{br}, CH*H*), -60.8 (s_{br}, CH*H*).

HR-ESI-MS found (calc) for $[C_{40}H_{88}Cl_2N_4P_4W_2]^{2+}$: 593.2181 (593.2166), 594.2182 (594.2177).

$[W(N)Cl(^HPNP)]OTf$ (**9^{OTf}**)

A solution of **6** (50 mg, 42.1 µmol, 1.0 eq) in THF (8 mL) is added a solution of HOTf (7.8 µL, 13.3 mg, 88.4 mmol, 2.1 eq) in THF (6 mL) at -78 °C leading to a color change from green to brownish orange. After stirring for 3 h, the solution is warmed to room temperature and dried *in vacuo*. The residue is washed with pentane (4x 5 mL), extracted with benzene (3x 4 mL) and concentrated *in vacuo*. Diffusion of pentane into the saturated benzene solution at RT gives **9^{OTf}** as pale yellow crystals (48 mg, 60 %).

Elem. Anal. found (calc) for $C_{21}H_{45}ClF_3N_2O_3P_2SW\bullet0.8\ C_6H_6$: C 38.23 (38.34); H 6.21 (6.22); N 3.29 (3.46).

HR-ESI-MS found (calc) for $C_{20}H_{45}ClN_2P_2W$: 594.2240 (594.2242), 596.2271 (596.2269), found (calc) for $C_{20}H_{45}ClN^{15}NP_2W$: 595.2212 (595.2226), 597.2242 (597.2260).

IR (ATR-IR): 3079 cm^{-1} (N-H); 1058 cm^{-1} (W≡N).

μ_{eff} = 1.8 ± 0.1 μ_B (Evans, THF-d_8).

Protonation of **6** with HOTf (1 equiv.) to [(^{H}PNP)ClW(N_2)WCl(PNP)]OTf (**10^{OTf}**)

HOTf (0.2 µL, 0.34 mg, 2.27 µmol, 1.0 eq) is added to a solution of **6** (2.7 mg, 2.27 µmol, 1.0 eq) in THF-d_8 (0.6 mL) in a J-Young-Tube at -35 °C. The reaction mixture was characterized *in situ* by NMR-spectroscopy.
^{1}H NMR (THF-d_8, 400 MHz, -35 °C, [ppm]): δ =5.43 (m, 1 H, N-*H*), 1.53 (d, $^3J_{HP}$ = 13.7 Hz, 9 H, ^{t}Bu), 1.40 (d, $^3J_{HP}$ = 13.1 Hz, 9 H, ^{t}Bu), 1.39 (d, $^3J_{HP}$ = 11.8 Hz, 9 H, ^{t}Bu), 1.26 (d, $^3J_{HP}$ = 14.4 Hz, 9 H, ^{t}Bu), 1.17-1.07 (m,36 H, 4 ^{t}Bu).
^{31}P{^{1}H] NMR (THF-d_8, 162 MHz, -35 °C,[ppm]): δ = 86.8 (d, $^2J_{PP}$ = 123.1 Hz), 79.5 (d, $^2J_{PP}$ = 123.2 Hz), 78.1 (d, $^2J_{PP}$ = 126.2 Hz), 74.6 (d, $^2J_{PP}$ = 126.7 Hz). **^{15}N{^{1}H} NMR** (THF-d_8, 50.7 MHz, -35 °C, [ppm]): δ = 27.0 (d, $^1J_{NN}$=10.3 Hz), 23.7 (d, $^1J_{NN}$=10.9 Hz).

Protonation of **6** with HOTf (2 equiv.) to [{(^{H}PNP)ClW}$_2${µ-N_2}]$(OTf)_2$ (**12^{OTf2}**)

HOTf (0.4 µL, 0.68 m, 4.52 µmol, 2.0 eq) is added to a solution of **6** (2.7 mg, 2.27 µmol, 1.0 eq) in THF-d_8 (0.6 mL) in a J-Young-Tube at -60 °C. The reaction mixture was characterized *in situ* by NMR spectroscopy.

1.4 Kinetic Analysis

K_1 determination

A solution of [$HNEt_3$][BAr^F_{24}] in THF (0.25 M; 20, 30, 40 or 50 µL, respectively) was added to a stirred solution of **6** in THF (2 mL, 0.25 M). The reaction was monitored by UVvis spectroscopy, measuring one spectrum every 3 sec.

k_2 and k_3 determination

A solution of [$HNEt_3$][BAr^F_{24}] (5, 7.5, 10 or 12.5 M) in THF (1 mL) was added to a 0.5 M solution of **6** in THF (1 mL) at 25 °C. The reaction was stirred and monitored by UVvis spectroscopy for 5 h, measuring one spectrum every 4 min.

k_3 determination via initial rate method

k_3 was determined by the initial rate method using an equimolar mixture of **6** and [H^+] (0.25 mM). It is assumed that under such conditions reaction proceeds mainly by the reaction pathway k_3. The initial rate for this reaction was calculated to be v = 1.35 x 10^{-9} M s^{-1} and it was determined from the spectral changes ascribed to the decrease in the concentration of **10^+**, (i.e. after time needed for the setting of first equilibrium). Taking into account that K_1 = 1592 M^{-1}, the concentration of **10^+** in the equilibrium can be calculated as [**10^+**] = 5.84 x 10^{-5} M at [**6**] = [H^+] = 0.00025 M. With $v = k_3 \times [\mathbf{10^+}]^2$ and [**10^+**] = 5.84 x 10^{-5} M, k_3 can be calculated as k_3 = 0.4 $M^{-1}s^{-1}$.

2. Spectroscopic Results

2.1 Characterization

$[WCl_3(PNP)]$ (**5**)

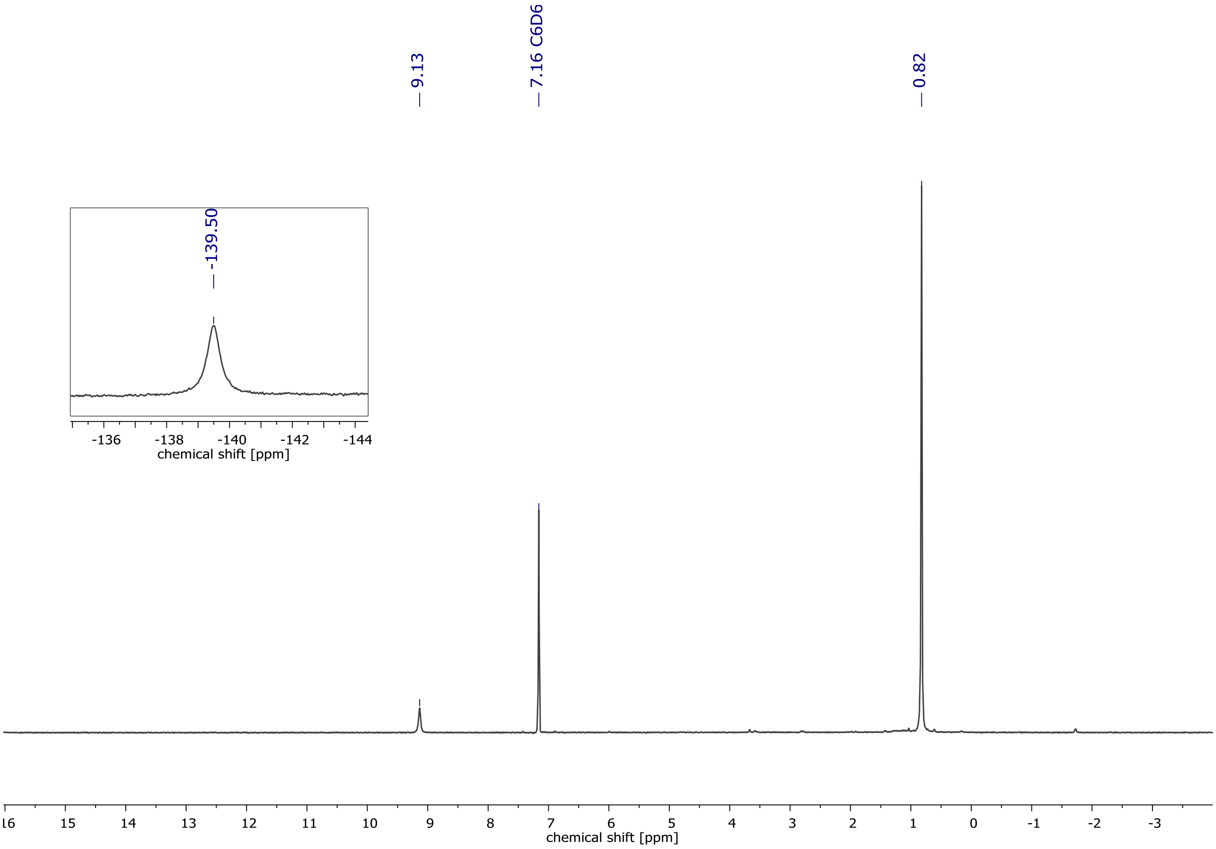

Figure S1 ^{1}H NMR spectrum of **5** in C_6D_6 at RT.

[$(N_2)\{WCl(PNP)\}_2$] (**6**)

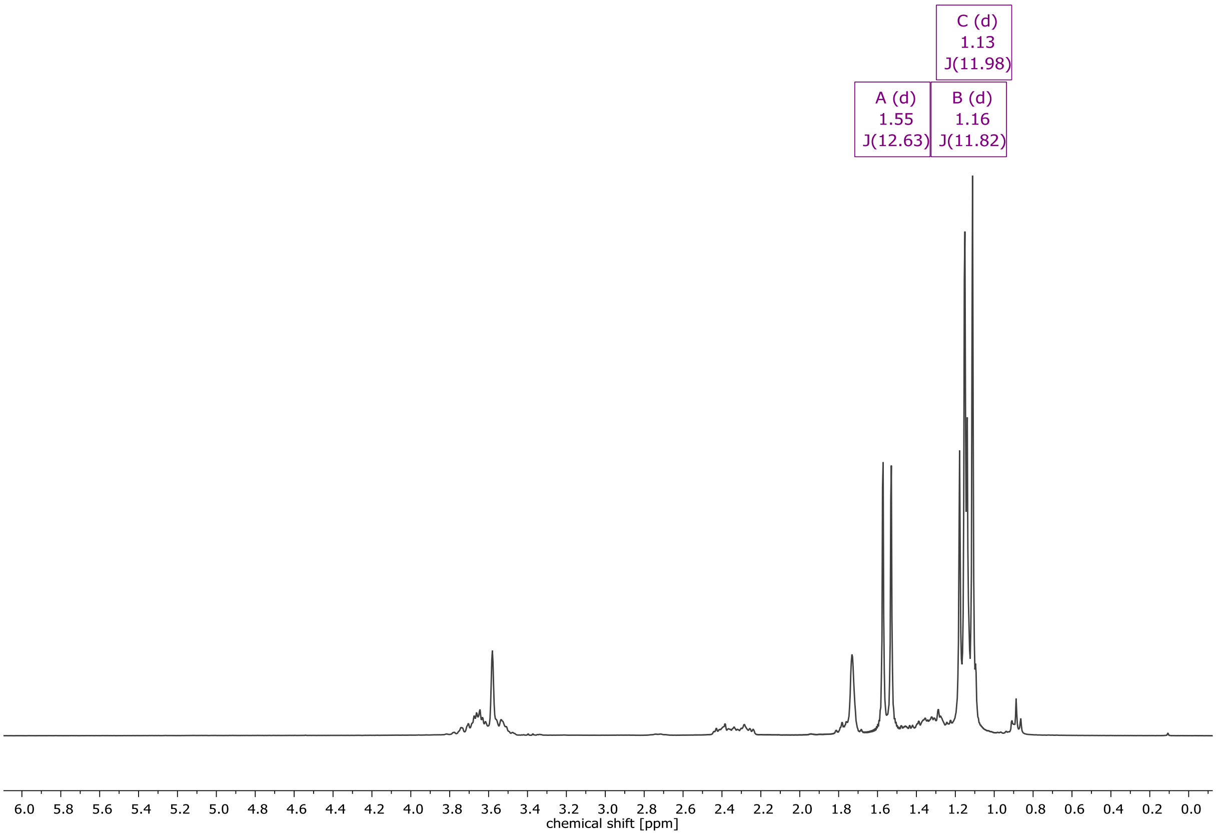

Figure S2 ^{1}H NMR spectrum of **6** in THF-d_8 at RT.

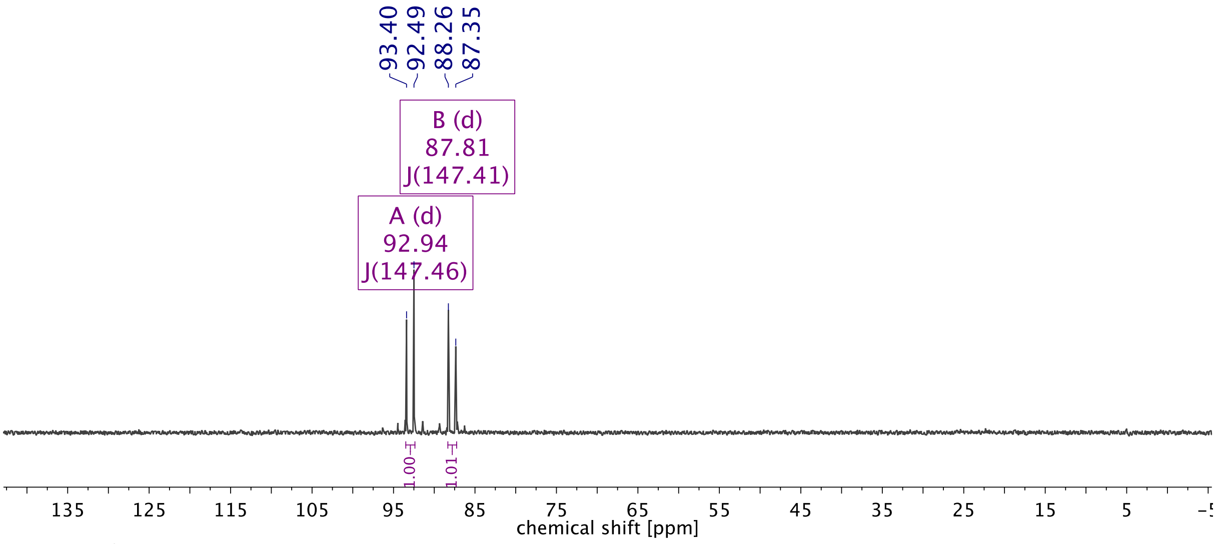

Figure S3 $^{31}P\{^{1}H\}$ NMR spectrum of **6** in THF-d_8 at RT.

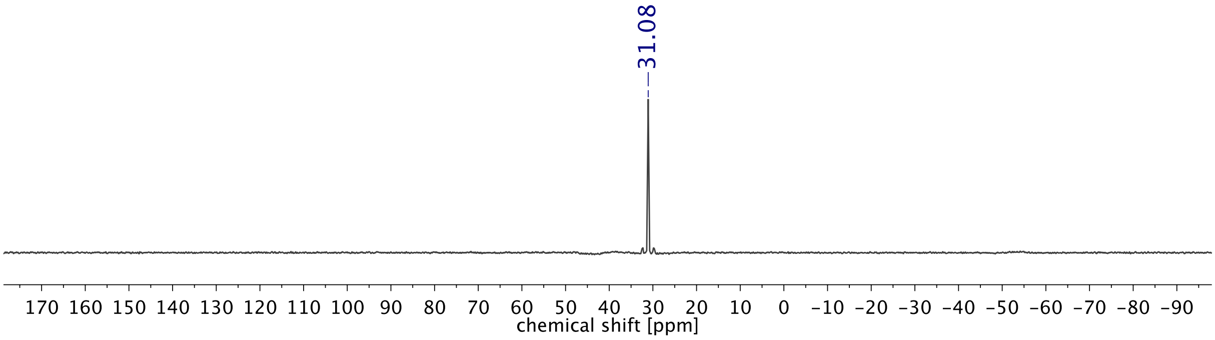

Figure S4 $^{15}N\{^{1}H\}$ NMR spectrum of $\mathbf{^{15}N}$**-6** in THF-d_8 at -35 °C.

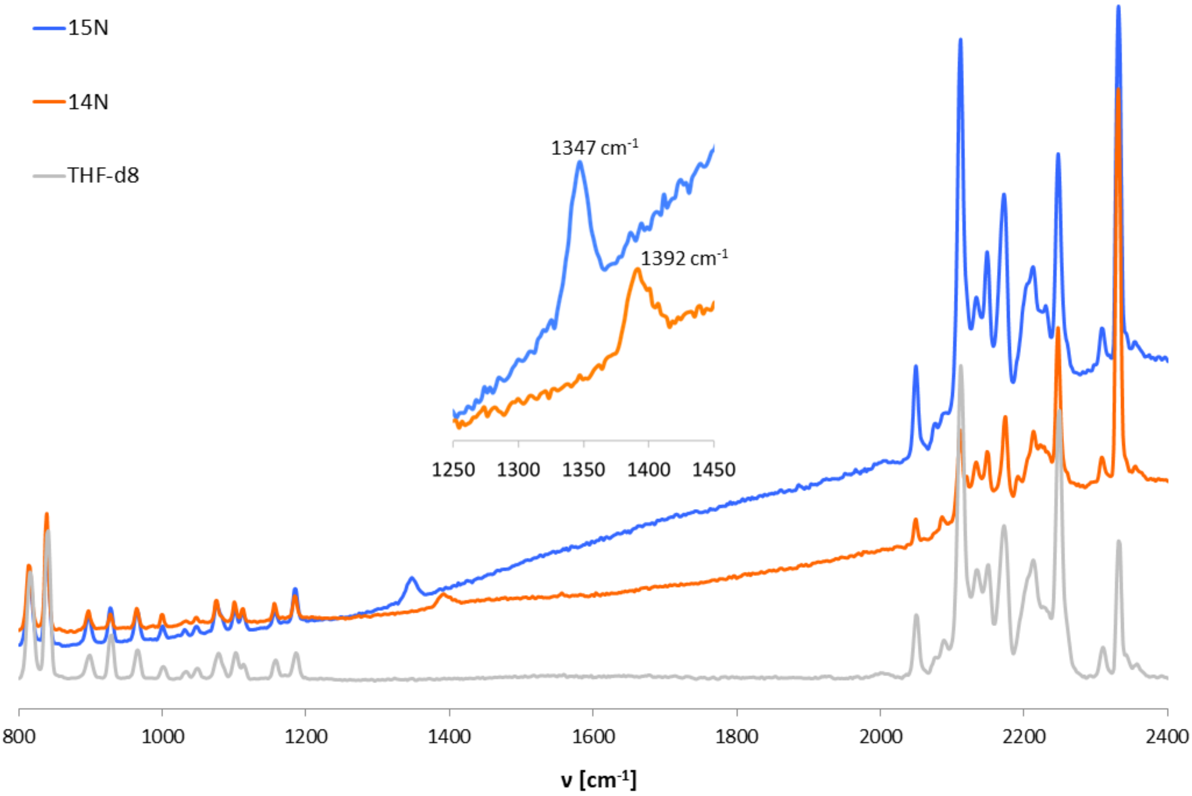

Figure S5 rRaman (457 nm) spectrum of **6/**$\mathbf{^{15}N}$**-6** in frozen THF-d_8.

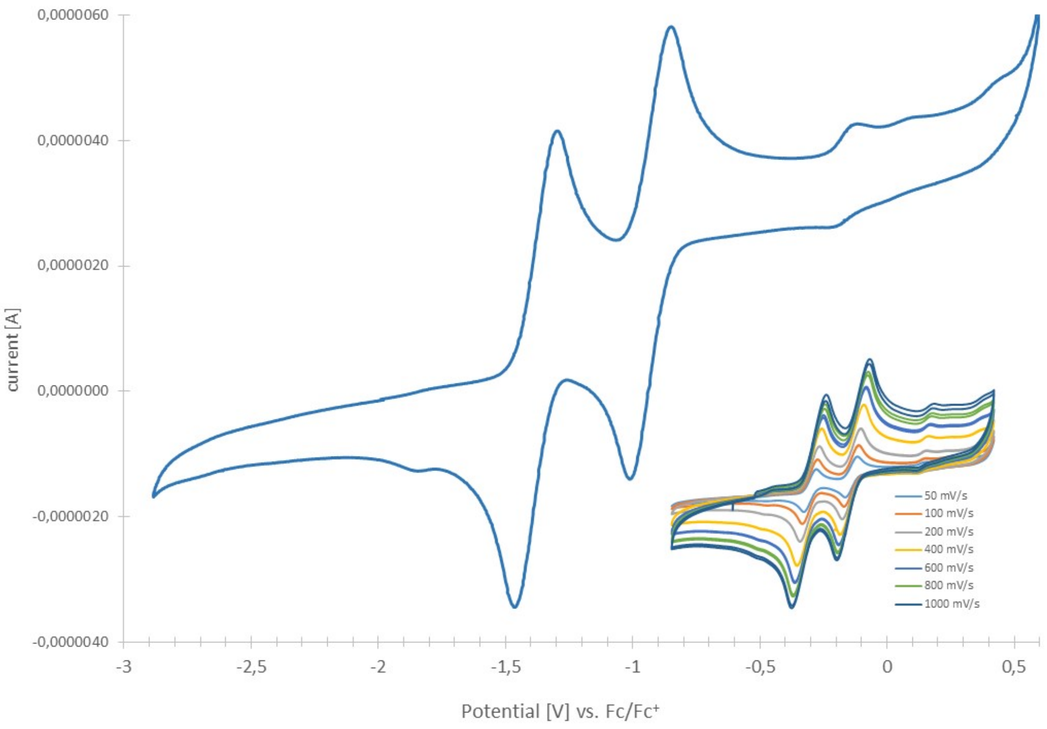

Figure S6 CV (100 mV/s, 2nd cycle) of **6** in 0.1 M solution of [N*n*Bu_4]PF_6 in THF (WE = GC, RE = Ag/Ag^+, CE = Pt).

[(N$_2$){WCl(PNP)}$_2$]$^+$ (**7**$^+$)

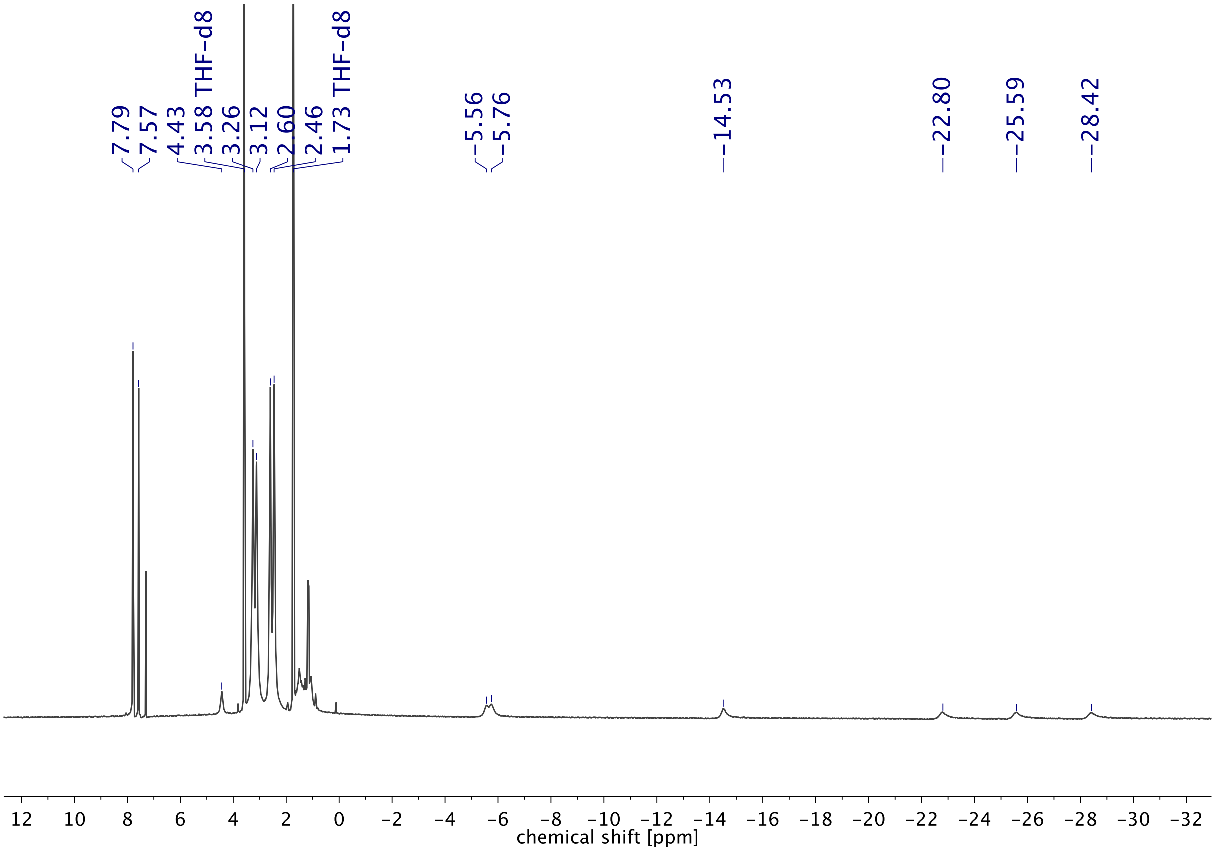

Figure S7 ^{1}H NMR spectrum of **7**$^+$ in THF-d$_8$ at RT.

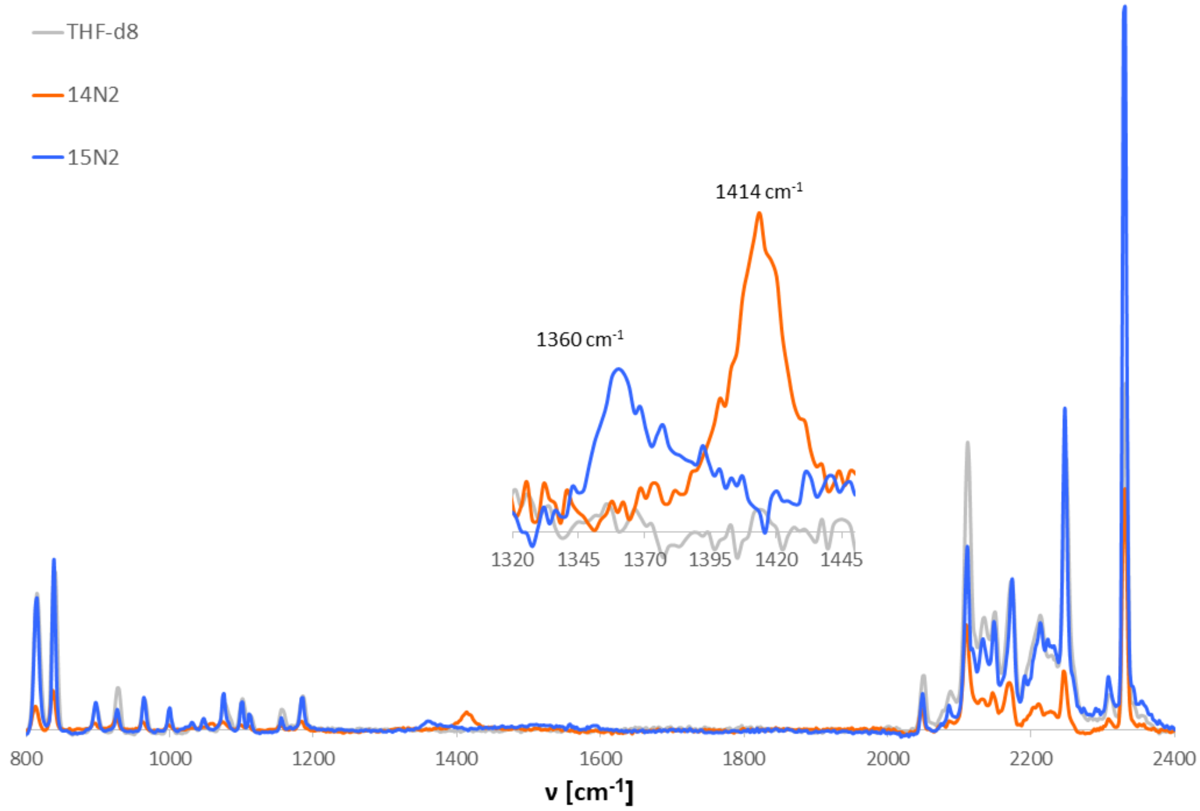

Figure S8 rRaman (457 nm) spectrum of **7**$^+$/^{15}**N-7**$^+$ in frozen THF-d$_8$.

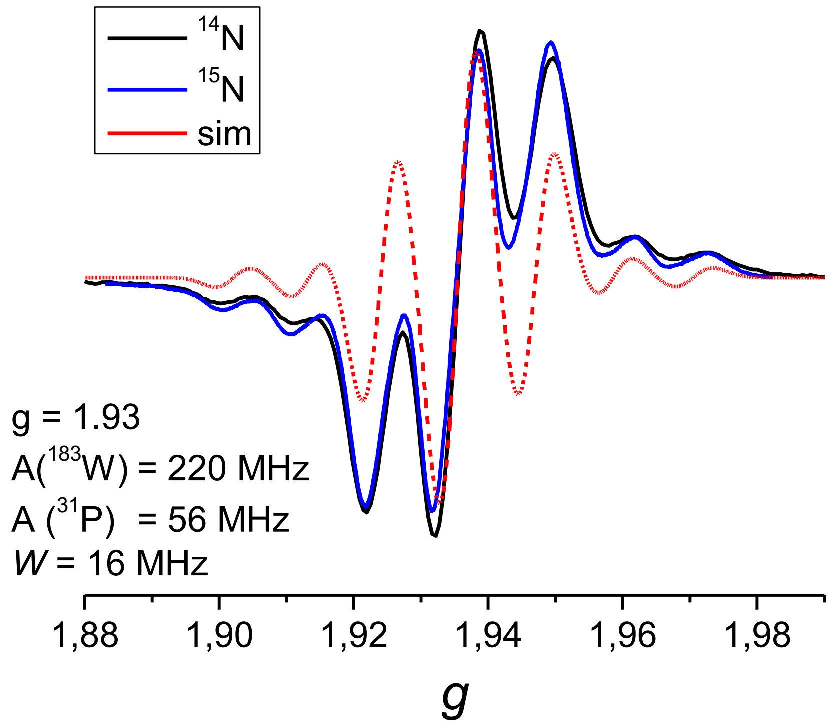

Figure S9 Comparison of the EPR-spectra of **7⁺** (black) and **¹⁵N-7⁺** (blue), both in THF, RT.

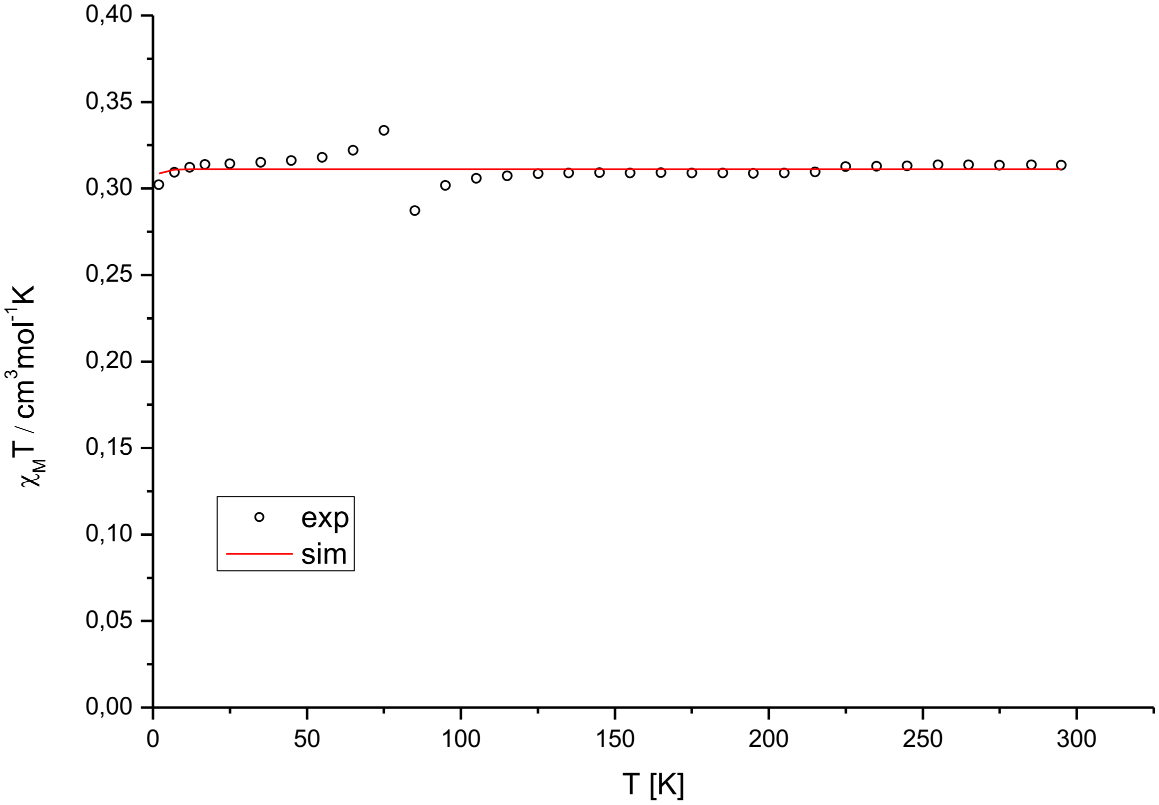

Figure S10. $\chi_M T$ vs. T plot for **7-BAr$^F_{24}$**. The open circles are the observed susceptibility, the red solid line corresponds to the best fit with the parameters g = 1.82 and TIP = $120 \cdot 10^{-6}$ cm^3mol^{-1} (TIP: temperature independent paramagnetism).

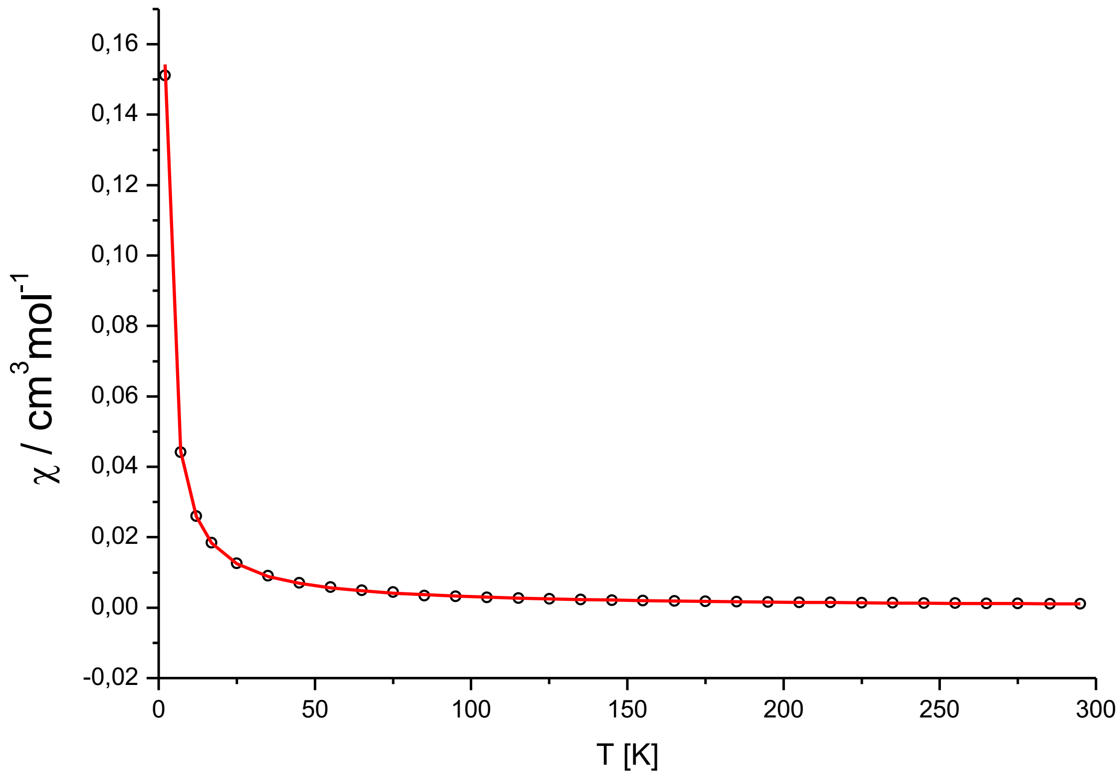

Figure S11 $\chi_M T$ vs. T plot for **7-BAr$^F_{24}$**. The open circles are the observed susceptibility, the red solid line corresponds to the best fit with the parameters g = 1.82 and TIP = $120 \cdot 10^{-6}$ cm^3mol^{-1} (TIP: temperature independent paramagnetism).

$[(N_2)\{WCl(PNP)\}_2]^{2+}$ (**8^{2+}**)

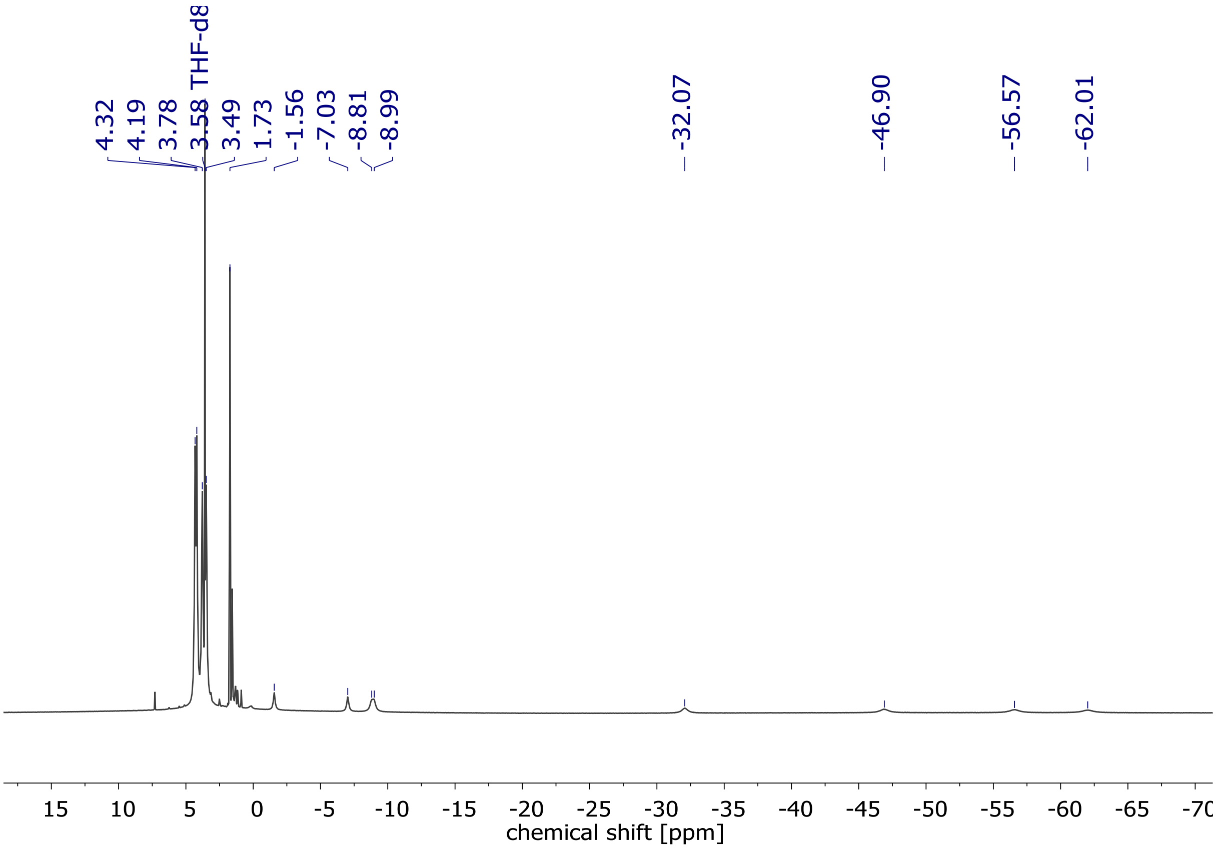

Figure S12 ^{1}H NMR spectrum of **8^{2+}** in THF-d$_8$ at RT.

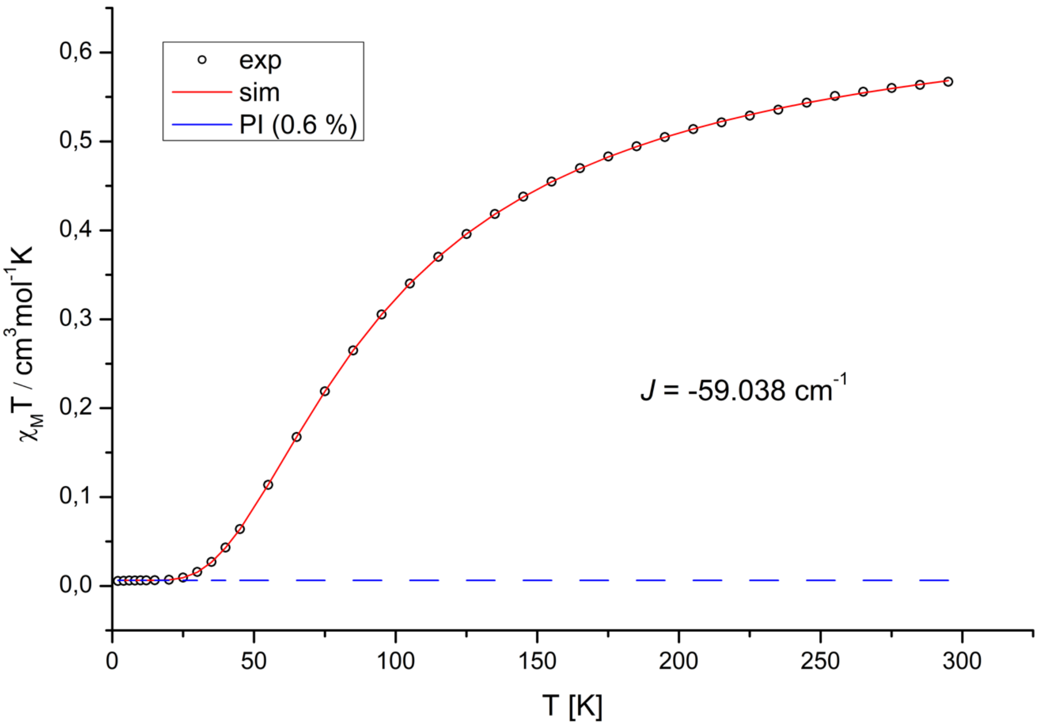

Figure S13 $\chi_M T$ vs. T plot for **8-(Al(OC(CF$_3$)$_3$)$_4$)$_2$** The open circles are the observed susceptibility, the red solid line corresponds to the best fit with the parameters g = 1.90, J = –59 cm^{-1}, TIP = 230·10^{-6} cm^{3}mol^{-1} and PI = 0.6 % (S = 1, the blue broken line, PI: paramagnetic impurity).

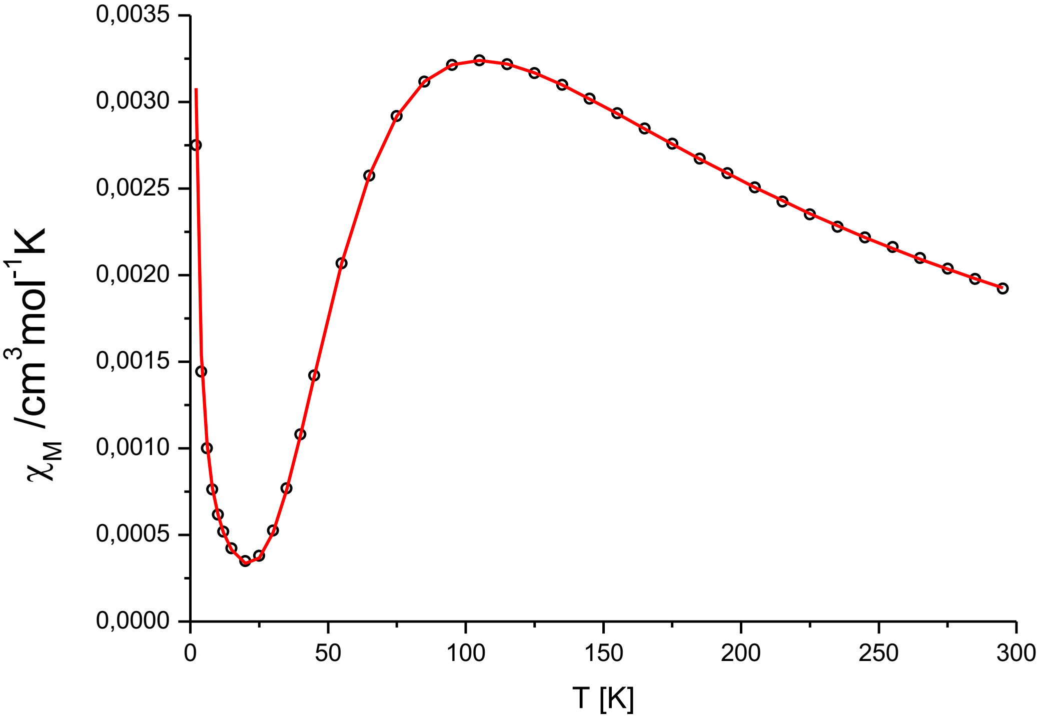

Figure S14 χ_M vs. T plot **for 8-(Al(OC(CF$_3$)$_3$)$_4$)$_2$** The open circles are the observed susceptibility, the red solid line corresponds to the best fit with the parameters g = 1.90, J = –59 cm^{-1}, TIP = 230·10^{-6} cm^{3}mol^{-1}.

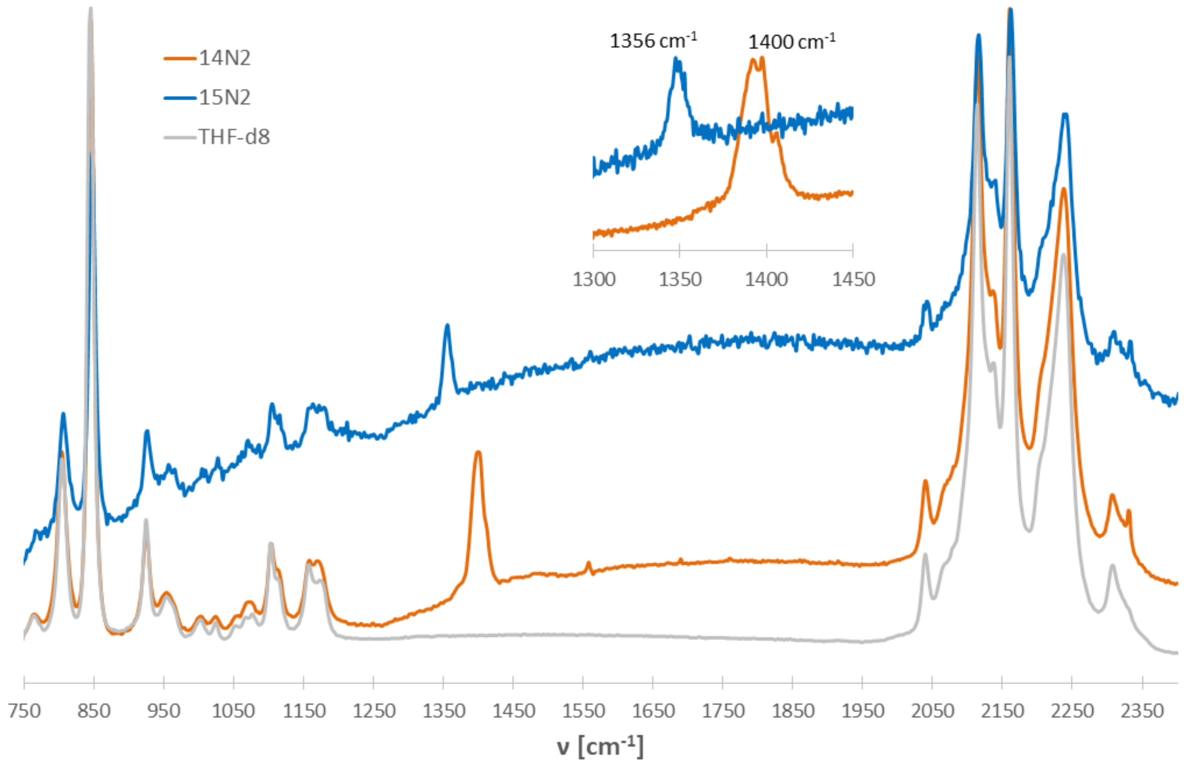

Figure S15 rRaman (514.5nm) spectrum of **8^{2+}/^{15}N-8^{2+}** in THF-d$_8$ at -100 °C.

[W(N)Cl(^{H}PNP)]OTf (**9^{OTf}**)

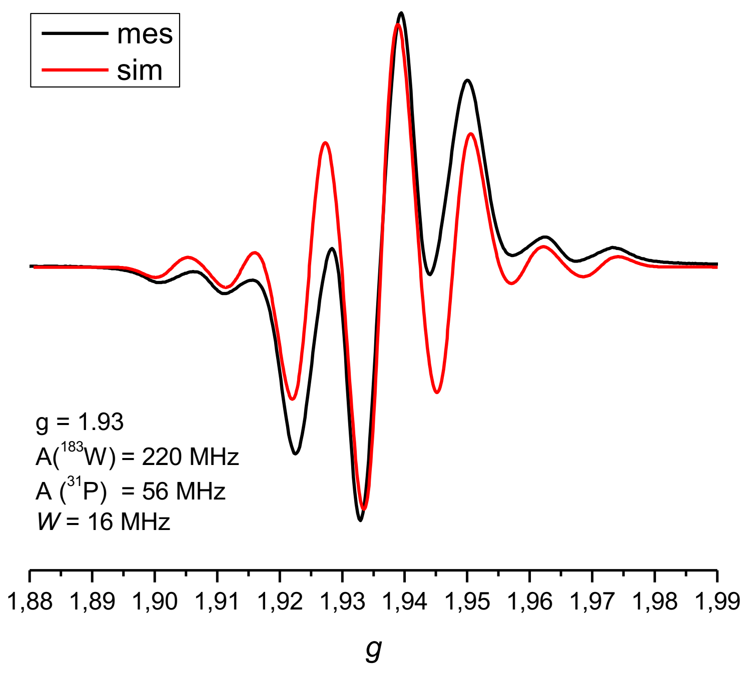

Figure S16 EPR spectrum of **9^{OTf}** in THF, RT.

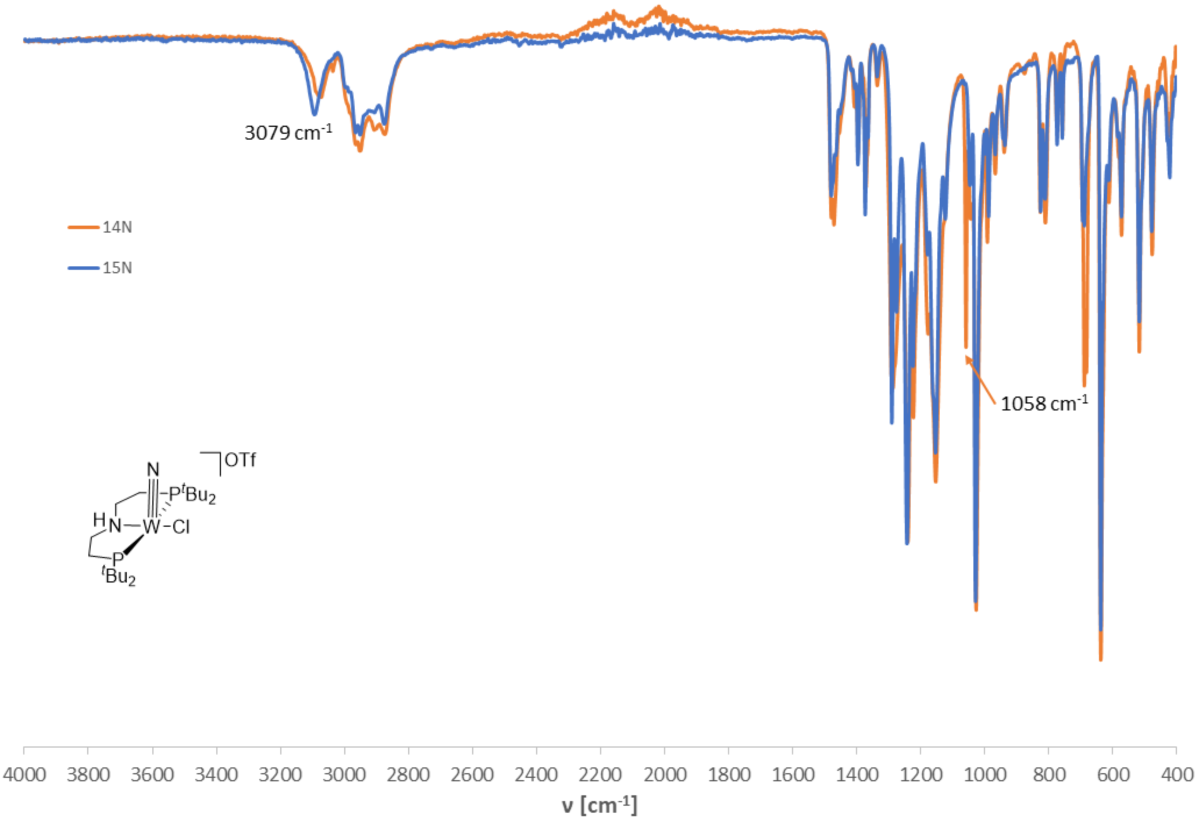

Figure S17 ATR-IR spectrum of **9^{OTf}**/**^{15}N-9^{OTf}**, solid, RT.

Monoprotonation of [(N_2){WCl(PNP)}$_2$] ($\mathbf{10^{OTf}}$)

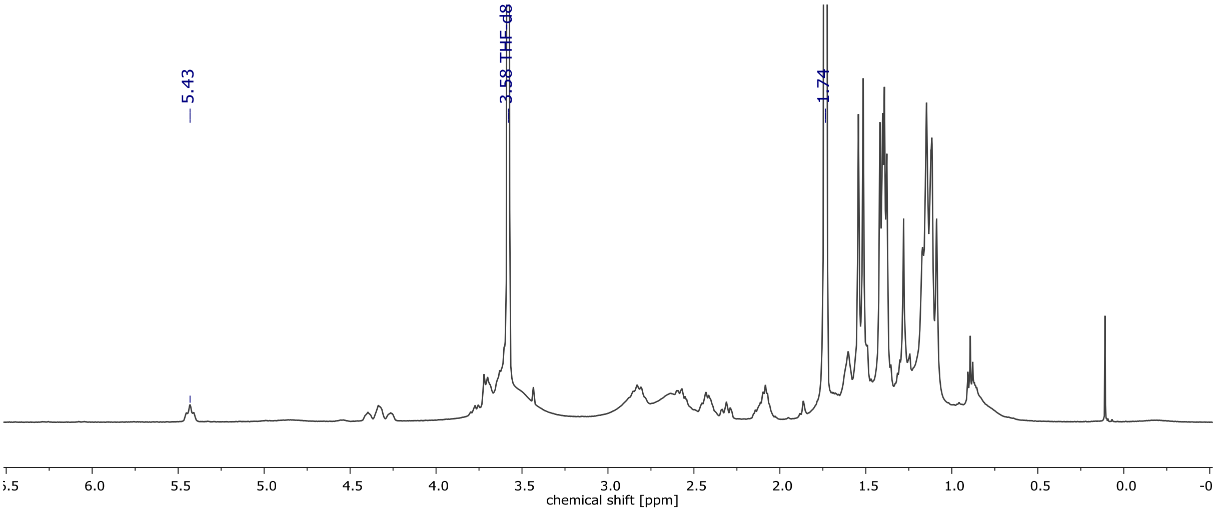

Figure S18 *in situ* ^{1}H NMR spectrum of $\mathbf{10^{OTf}}$ in THF-d$_8$ at -35 °C.

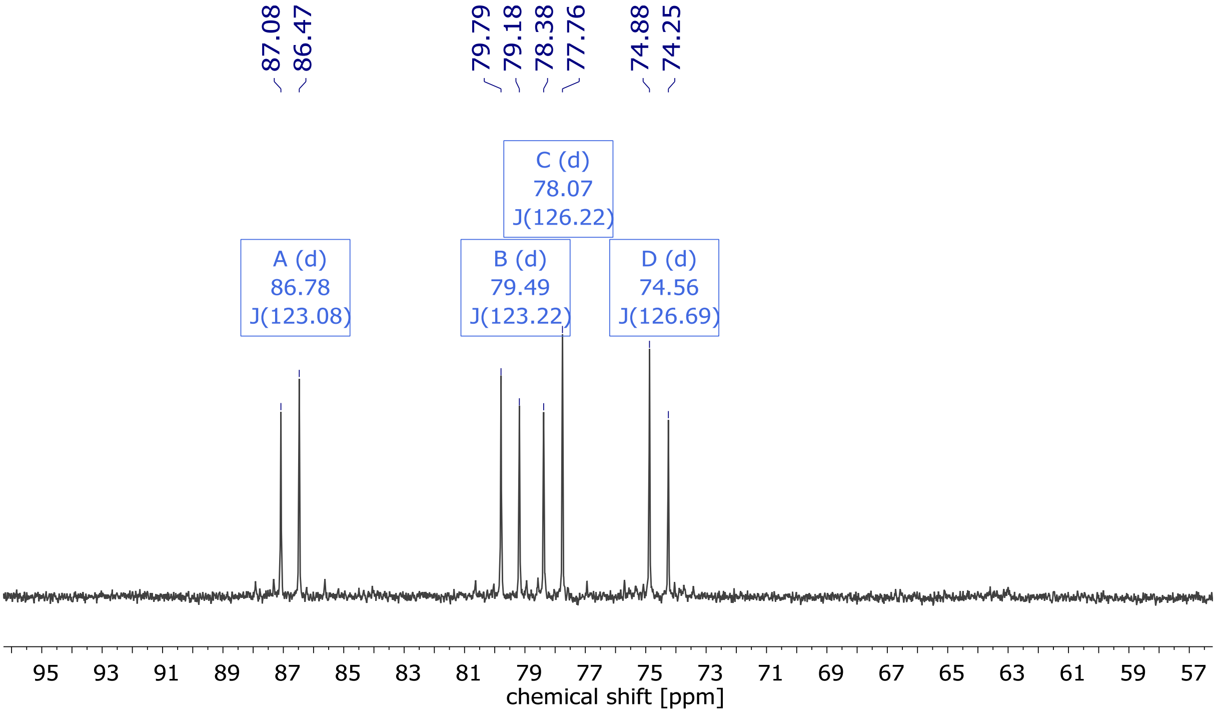

Figure S19 *in situ* ^{31}P{^{1}H} NMR spectrum of $\mathbf{10^{OTf}}$ in THF-d$_8$ at -35 °C.

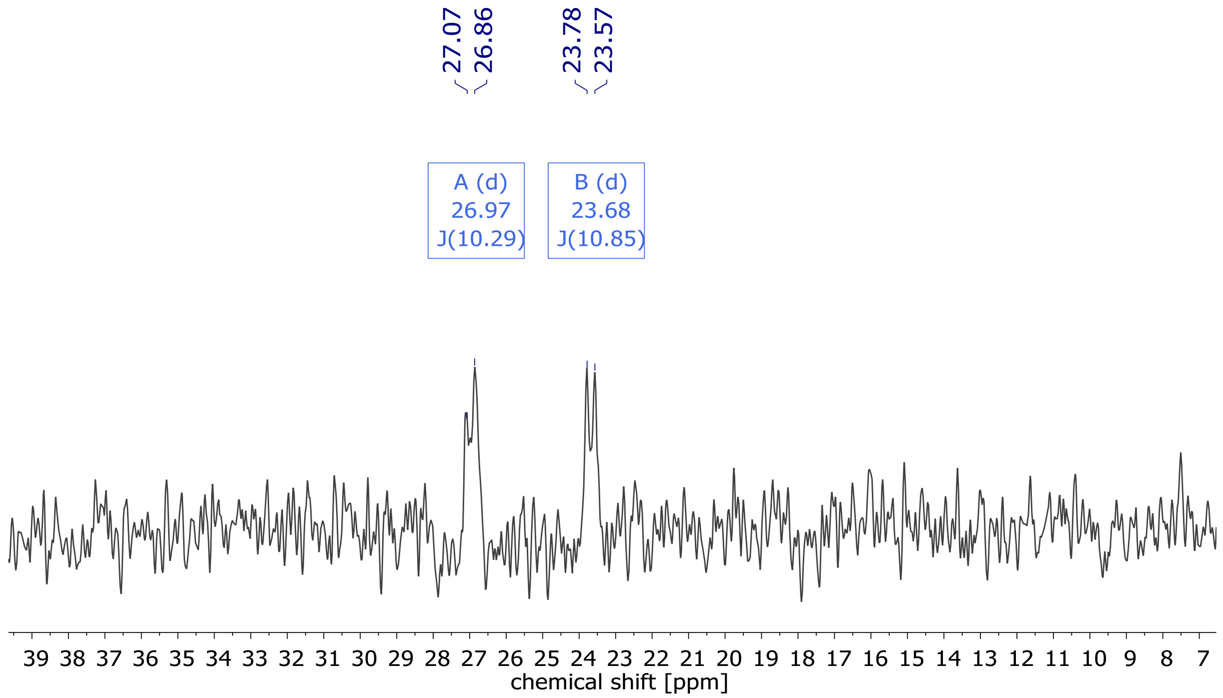

Figure S20 *in situ* ^{15}N{^{1}H} NMR spectrum of **^{15}N-10^{OTf}** in THF-d_8 at -35 °C.

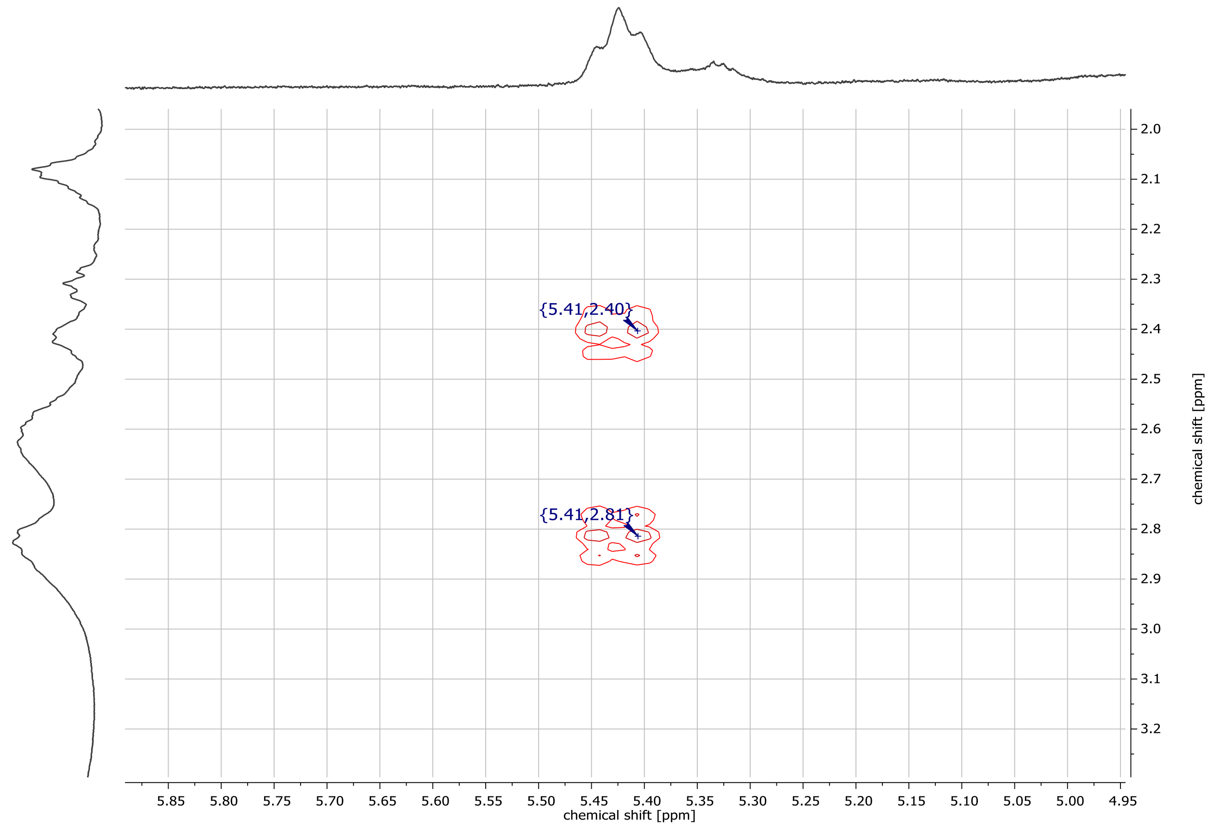

Figure S21 *in situ* ^{1}H{^{31}P} COSY NMR spectrum of **10^{OTf}** in THF-d_8 at -35 °C showing a cross peak between the N*H* proton (5.41 ppm) and the NCH*H* protons (2.40 and 2.81 ppm).

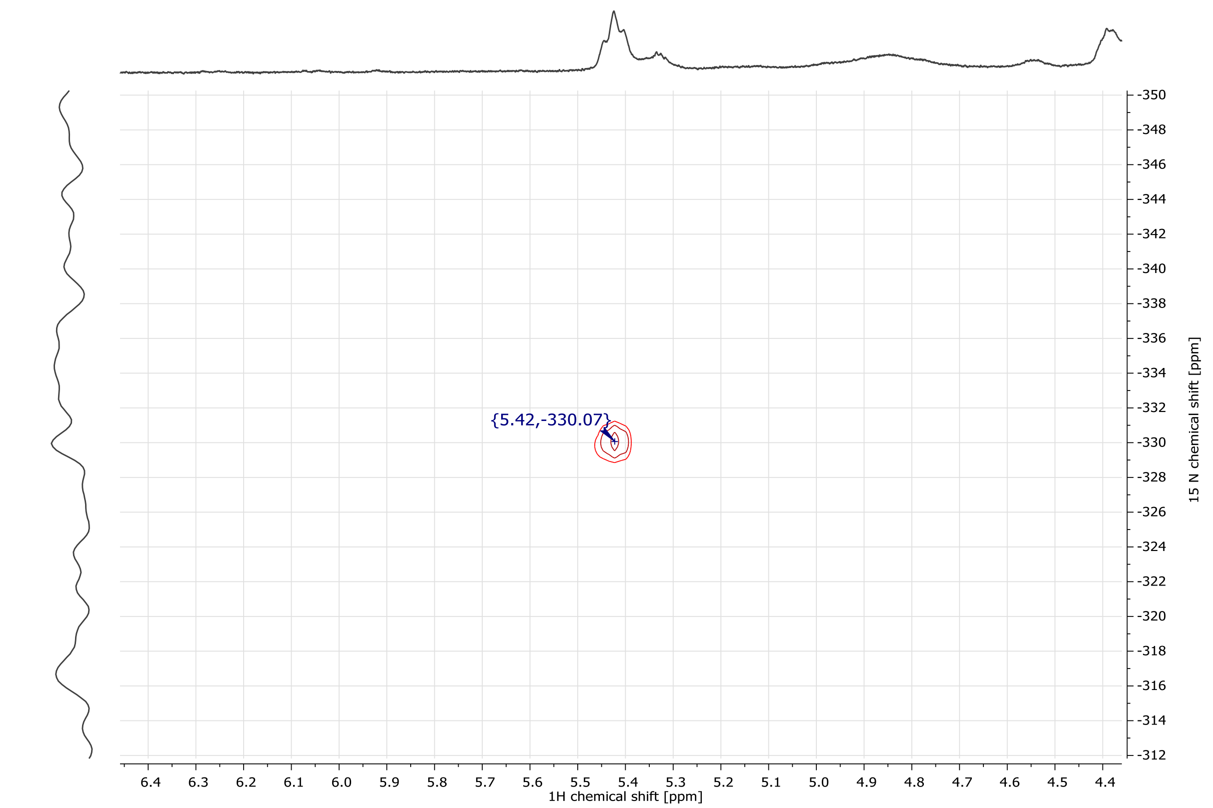

Figure S22 *in situ* ^{1}H-^{15}N HSQC NMR spectrum of **10^{OTf}** in THF-d_8 at -35 °C showing a cross peak between the N*H* proton (5.42 ppm) and amino nitrogen (-330 ppm).

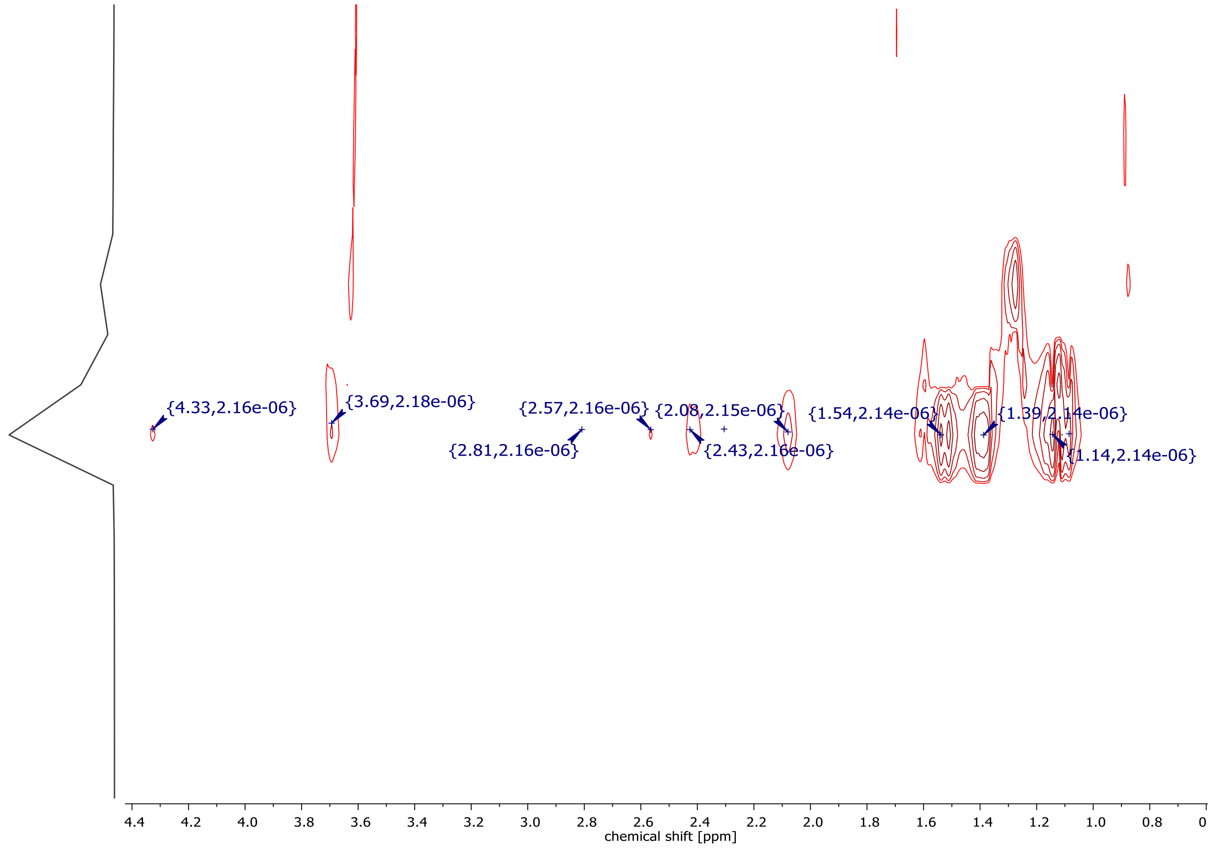

Figure S23 *in situ* ^{1}H DOSY NMR spectrum of **10^{OTf}** in THF-d_8 at -35 °C.

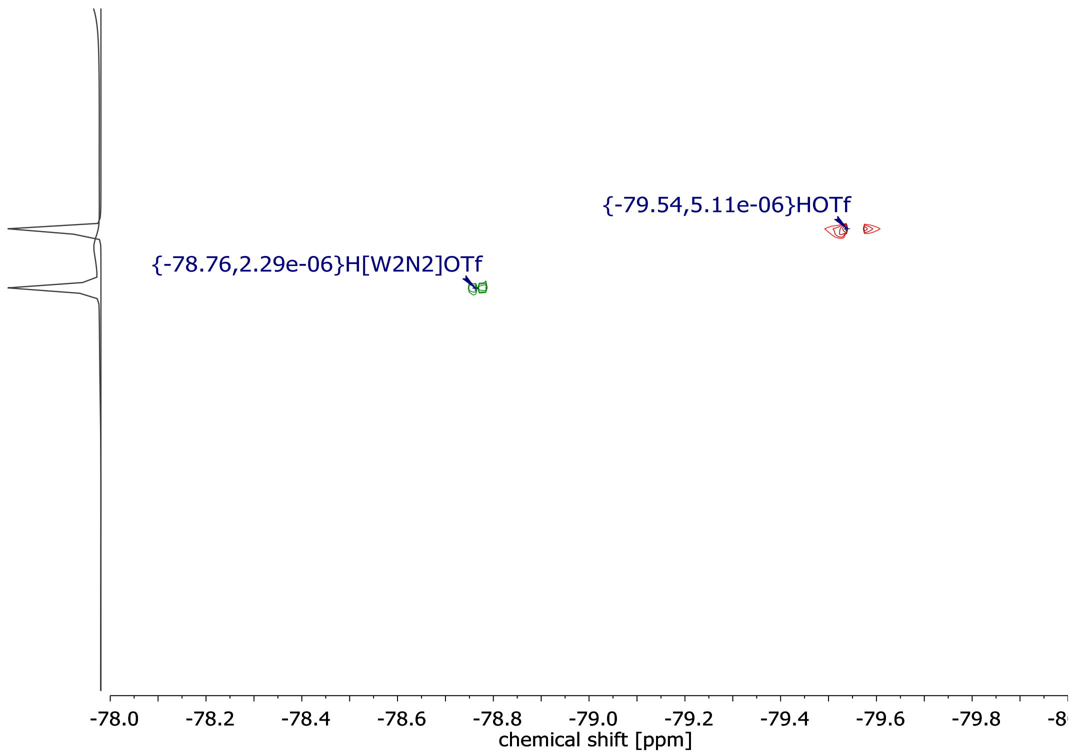

Figure S24 Comparison of the ^{19}F-DOSY NMR spectra $\mathbf{10^{OTf}}$ (*in situ*, green) with free HOTf (red) both in THF-d_8 at -35 °C.

Double protonation of $[(N_2)\{WCl(PNP)\}_2]$ ($\mathbf{11^{(OTf)_2}}$)

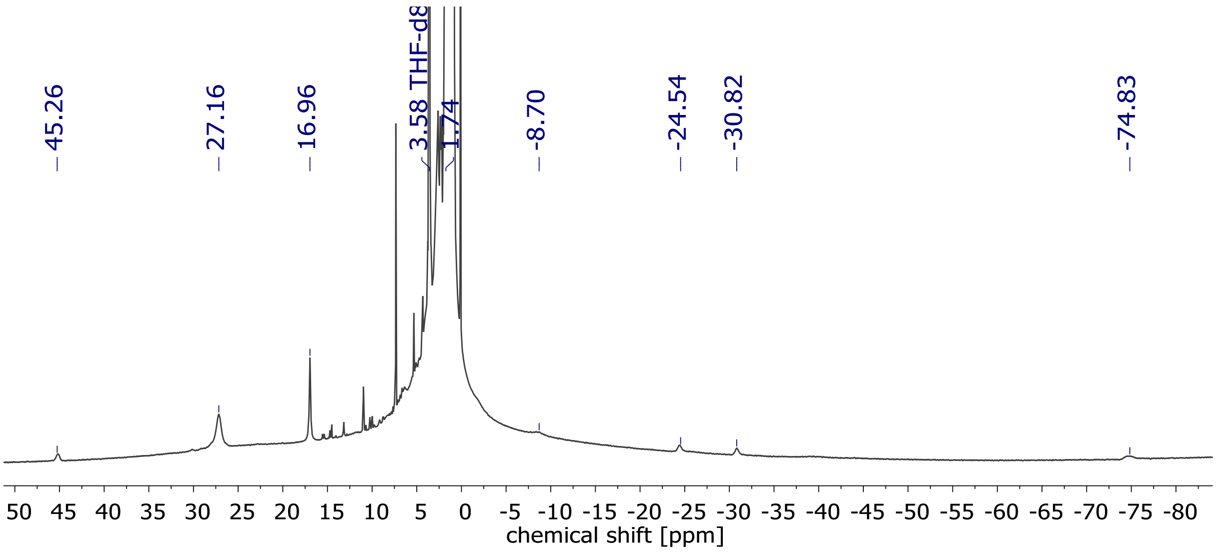

Figure S25 *in situ* ^{1}H NMR spectrum of $\mathbf{11^{(OTf)_2}}$ in THF-d_8 at -60 °C.

2.2 Kinetic Analysis

Exemplary UVvis spectra

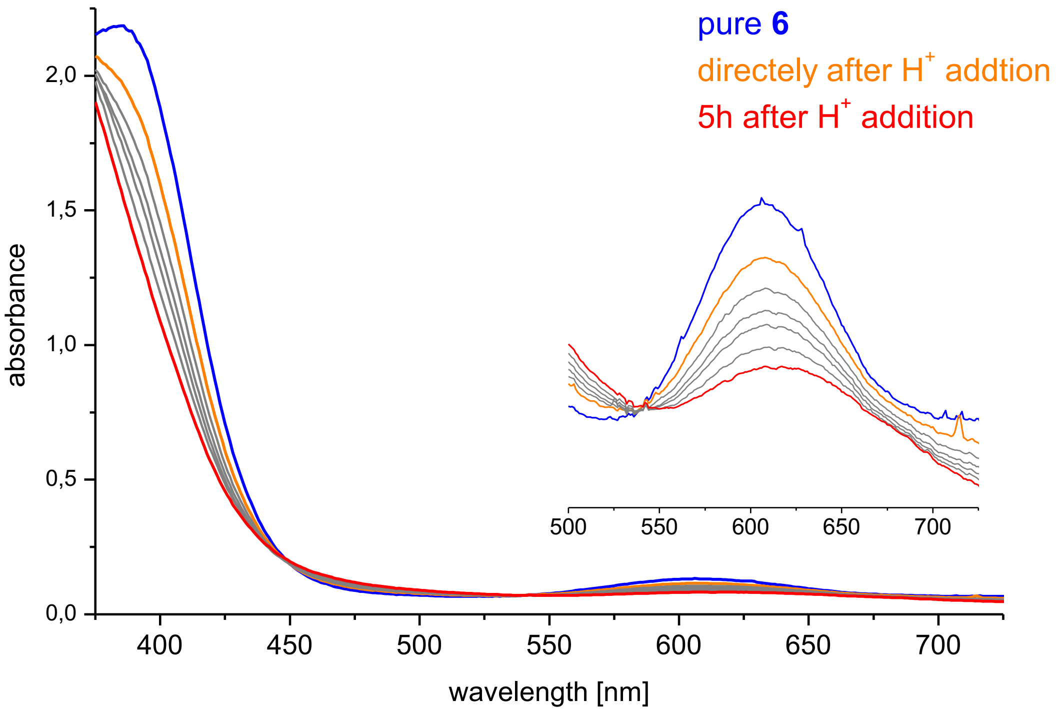

Figure S26 Exemplary UVvis spectrum of the reaction of **6** with 10 equiv. $[HNEt_3][BAr^F{}_{24}]$. The blue trace shows the spectrum of pure **6**. The orange trace shows the spectrum directly after addition of acid. The red trace shows the spectrum after 5h. The grey traces indicate the progress of the reaction within 1 h.

K_1 determination

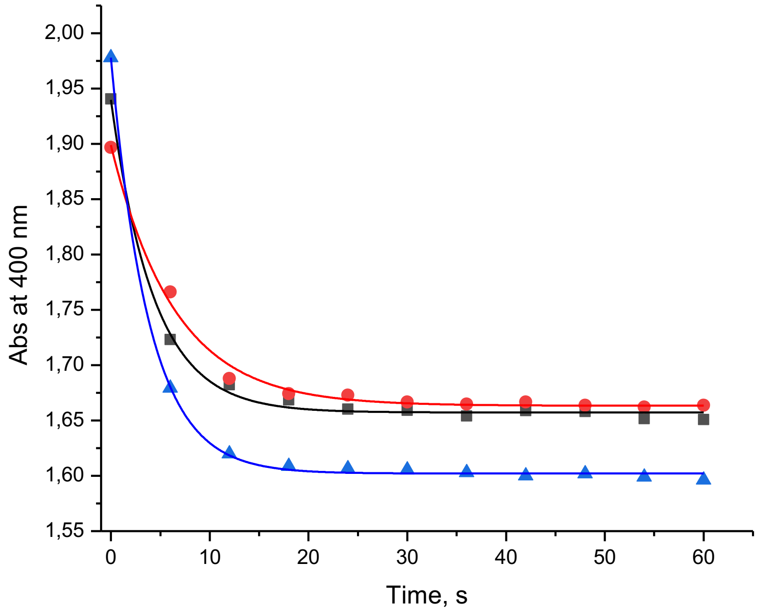

Figure S27 Absorbance (at 400 nm) vs. time plot for the initial protonation of **6** under conditions of 10 equiv. of $[HNEt_3][BAr^F{}_{24}]$.

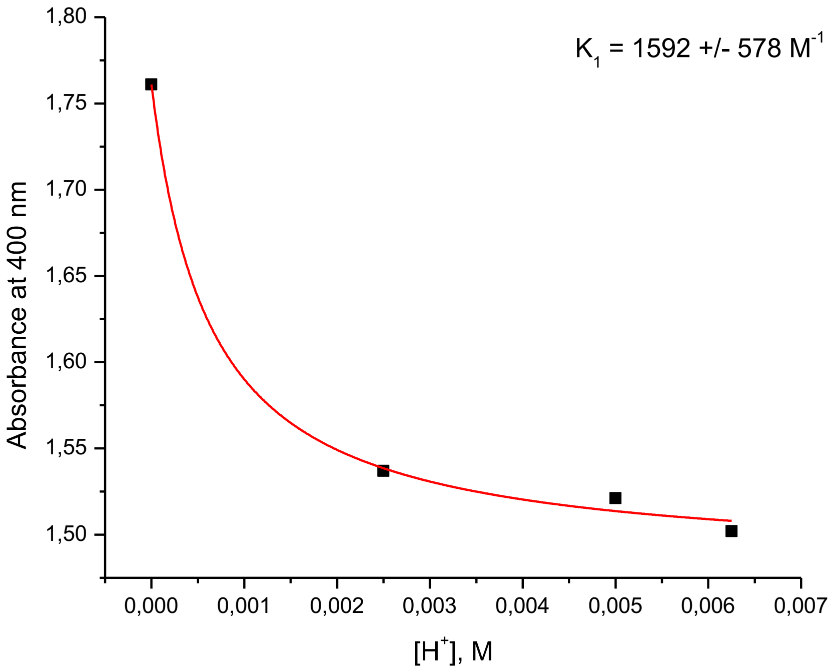

Figure S28 Absorbance (at 400 nm) vs. $[H^+]$ plot for the reaction of **6** with $[HNEt_3][BAr^F{}_{24}]$ to determine the initial, fast pre-equilibrium K_1. The value of K_1 was obtained from the fit of experimental data (R^2 = 0.998) to equation $A^{400} = (A_0{}^{400} + A_1{}^{400} \times K_1 \times [H^+])/(1 + K_1 \times [H^+])$, where $A_0{}^{400}$ and $A_1{}^{400}$ represent absorbance at 400 nm in the absence of acid and for the fully formed protonated species, respectively.[10]

k_2 and k_3 determination

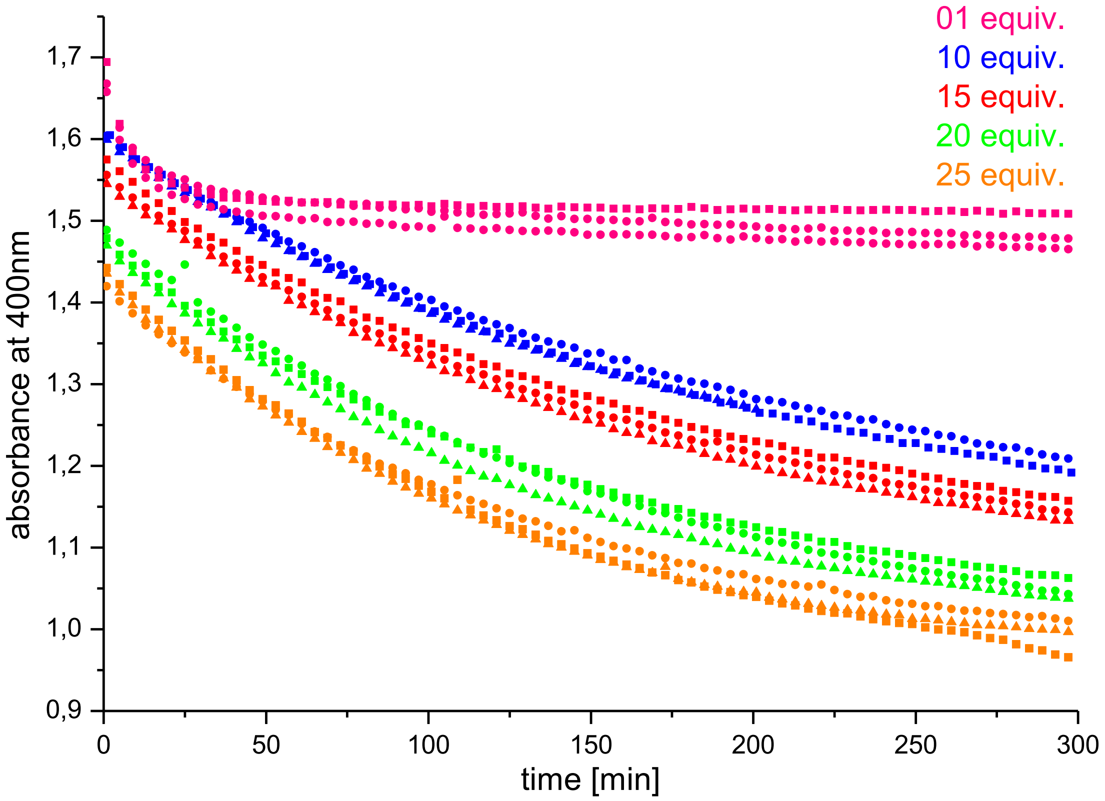

Figure S29 Absorbance at 400 nm vs. time plot for the second, slow reaction of **6** with different amounts of $[HNEt_3][BAr^F_{24}]$.

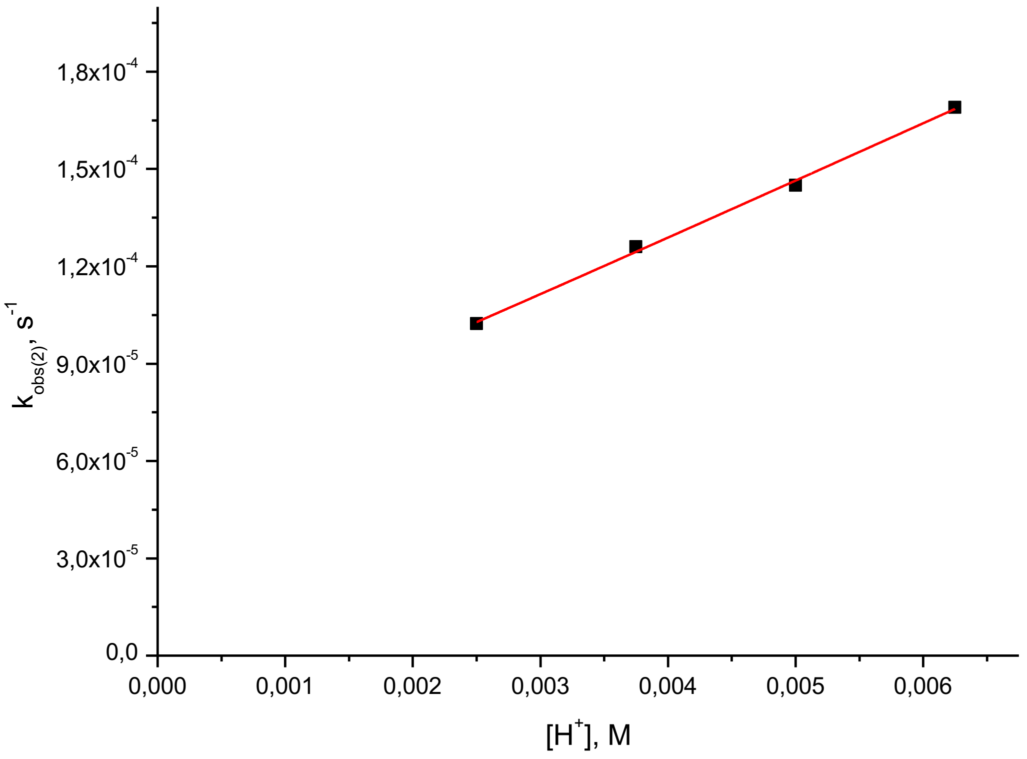

Figure S30 k_{2obs} vs. $[H^+]$ plot for the reaction of **6** with $[HNEt_3][BAr^F_{24}]$. Experimental data were fitted to eq. 1 (R^2 = 0.998) with fixed value of K_1 = 1592 ± 578 M^{-1}.

3. Crystallographic Details

CCDC-1943888 (**5**), CCDC-1943889 (**6**), CCDC-1943890 (**7**$^{+}$), CCDC-1943891(**8**$^{2+}$) and CCDC-1943892 (**9**$^{+}$) contain the supplementary crystallographic data for this paper. This data can be obtained free of charge via http://www.ccdc.cam.ac.uk/ products/csd/request/ (or from Cambridge Crystallographic Data Centre, 12 Union Road, Cambridge, CB2 1EZ, UK. Fax: +44-1223- 336-033; e-mail: deposit@ccdc.cam.ac.uk).

Suitable single crystals for X-ray structure determination were selected from the mother liquor under an inert gas atmosphere and transferred in protective perfluoro polyether oil on a microscope slide. The selected and mounted crystals were transferred to the cold gas stream on the diffractometer. The diffraction data were obtained at 100 K on a Bruker D8 three-circle diffractometer, equipped with a PHOTON 100 CMOS detector and an INCOATEC microfocus source with Quazar mirror optics (Mo-Kα radiation, λ= 0.71073 Å).

The data obtained were integrated with SAINT and a semi-empirical absorption correction from equivalents with SADABS was applied. The structures were solved and refined using the Bruker SHELX 2014 software package.[11] All non-hydrogen atoms were refined with anisotropic displacement parameters. All C-H hydrogen atoms were refined isotropically on calculated positions by using a riding model with their U_{iso} values constrained to 1.5 U_{eq} of their pivot atoms for terminal sp3 carbon atoms and 1.2 times for all other atoms.

Crystal Structure of **5**

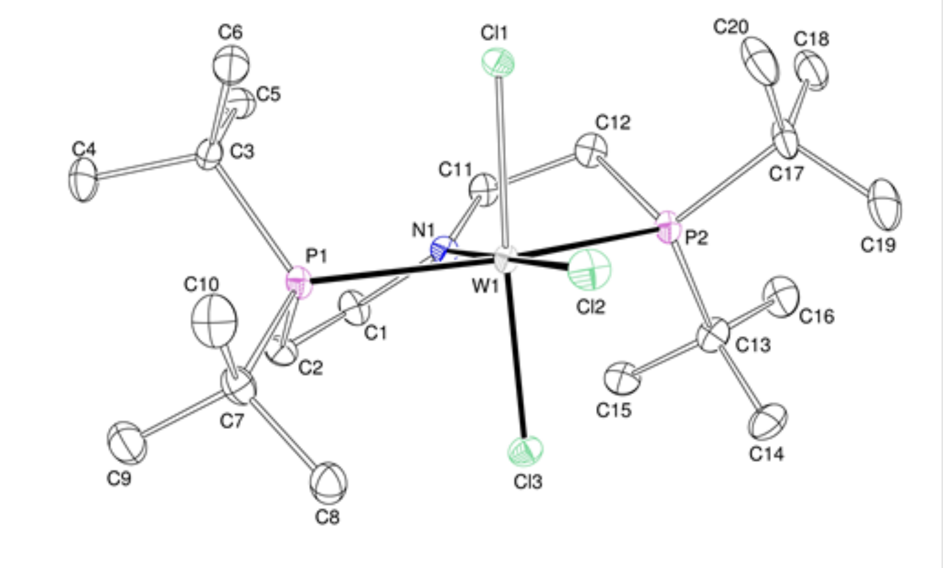

Figure S31 Thermal ellipsoid plot of **5** with the anisotropic displacement parameters drawn at the 50% probability level. The asymmetric unit contains one complex molecule.

Table S1 Crystal data and structure refinement for 5.

Identification code	mo_CV_BS_260516_0m_a (BS-A-122)	
Empirical formula	$C_{20}H_{44}Cl_3NP_2W$	
Formula weight	650.70	
Temperature	100(2) K	
Wavelength	0.71073 Å	
Crystal system	Orthorhombic	
Space group	Pbca	
Unit cell dimensions	a = 13.1751(7) Å	α= 90°
	b = 14.2709(8) Å	β= 90°
	c = 28.3358(16) Å	γ = 90°
Volume	5327.7(5) $Å^3$	
Z	8	
Density (calculated)	1.622 Mg/m^3	
Absorption coefficient	4.765 mm^{-1}	
F(000)	2608	
Crystal size	0.185 x 0.129 x 0.074 mm^3	
Crystal shape and color	Block, clear light orange-yellow	
Theta range for data collection	2.111 to 27.174°	
Index ranges	-16<=h<=16, -18<=k<=18, -36<=l<=36	
Reflections collected	213569	
Independent reflections	5908 [R(int) = 0.1012]	
Completeness to theta = 25.242°	100.0 %	
Max. and min. transmission	0.7455 and 0.6750	
Refinement method	Full-matrix least-squares on F^2	
Data / restraints / parameters	5908 / 0 / 256	
Goodness-of-fit on F2	1.081	
Final R indices [I>2sigma(I)]	R1 = 0.0291,	wR2 = 0.0473
R indices (all data)	R1 = 0.0451,	wR2 = 0.0507
Largest diff. peak and hole	1.018 and -1.379 $eÅ^{-3}$	

Table S2 Bond lengths [Å] and angles [°] for 5.

C(1)-N(1)	1.477(4)	C(11)-C(12)-P(2)	112.0(3)
C(1)-C(2)	1.533(5)	C(16)-C(13)-C(14)	109.9(3)
C(2)-P(1)	1.836(3)	C(16)-C(13)-C(15)	106.8(3)
C(3)-C(4)	1.535(5)	C(14)-C(13)-C(15)	108.3(3)
C(3)-C(6)	1.542(5)	C(16)-C(13)-P(2)	112.3(3)
C(3)-C(5)	1.549(5)	C(14)-C(13)-P(2)	110.9(3)
C(3)-P(1)	1.894(4)	C(15)-C(13)-P(2)	108.5(3)
C(7)-C(10)	1.530(5)	C(19)-C(17)-C(18)	110.0(3)
C(7)-C(8)	1.534(5)	C(19)-C(17)-C(20)	106.5(3)
C(7)-C(9)	1.535(5)	C(18)-C(17)-C(20)	107.3(3)
C(7)-P(1)	1.884(4)	C(19)-C(17)-P(2)	110.7(3)
C(11)-N(1)	1.458(5)	C(18)-C(17)-P(2)	112.5(3)
C(11)-C(12)	1.538(5)	C(20)-C(17)-P(2)	109.6(3)
C(12)-P(2)	1.839(4)	C(11)-N(1)-C(1)	110.7(3)
C(13)-C(16)	1.534(5)	C(11)-N(1)-W(1)	125.1(2)
C(13)-C(14)	1.536(5)	C(1)-N(1)-W(1)	124.2(2)
C(13)-C(15)	1.542(5)	C(2)-P(1)-C(7)	105.17(17)
C(13)-P(2)	1.897(4)	C(2)-P(1)-C(3)	105.08(17)
C(17)-C(19)	1.536(6)	C(7)-P(1)-C(3)	110.02(17)
C(17)-C(18)	1.538(6)	C(2)-P(1)-W(1)	93.55(12)
C(17)-C(20)	1.541(5)	C(7)-P(1)-W(1)	121.45(12)
C(17)-P(2)	1.904(4)	C(3)-P(1)-W(1)	117.69(12)
N(1)-W(1)	1.938(3)	C(12)-P(2)-C(13)	104.14(17)
P(1)-W(1)	2.5683(9)	C(12)-P(2)-C(17)	104.23(18)
P(2)-W(1)	2.5873(9)	C(13)-P(2)-C(17)	109.06(18)
Cl(1)-W(1)	2.4020(9)	C(12)-P(2)-W(1)	92.20(12)
Cl(2)-W(1)	2.4137(9)	C(13)-P(2)-W(1)	119.31(13)
Cl(3)-W(1)	2.3811(9)	C(17)-P(2)-W(1)	122.84(13)
		N(1)-W(1)-Cl(3)	91.91(9)
N(1)-C(1)-C(2)	114.4(3)	N(1)-W(1)-Cl(1)	91.76(9)
C(1)-C(2)-P(1)	112.0(2)	Cl(3)-W(1)-Cl(1)	175.71(3)
C(4)-C(3)-C(6)	108.5(3)	N(1)-W(1)-Cl(2)	178.56(9)
C(4)-C(3)-C(5)	106.9(3)	Cl(3)-W(1)-Cl(2)	88.81(3)
C(6)-C(3)-C(5)	109.0(3)	Cl(1)-W(1)-Cl(2)	87.58(3)
C(4)-C(3)-P(1)	113.1(3)	N(1)-W(1)-P(1)	78.50(9)
C(6)-C(3)-P(1)	111.1(3)	Cl(3)-W(1)-P(1)	90.29(3)
C(5)-C(3)-P(1)	108.0(2)	Cl(1)-W(1)-P(1)	92.62(3)
C(10)-C(7)-C(8)	107.6(3)	Cl(2)-W(1)-P(1)	100.25(3)
C(10)-C(7)-C(9)	109.9(3)	N(1)-W(1)-P(2)	78.83(9)
C(8)-C(7)-C(9)	107.4(3)	Cl(3)-W(1)-P(2)	91.92(3)
C(10)-C(7)-P(1)	109.6(3)	Cl(1)-W(1)-P(2)	86.61(3)
C(8)-C(7)-P(1)	109.3(3)	Cl(2)-W(1)-P(2)	102.40(3)
C(9)-C(7)-P(1)	112.9(3)	P(1)-W(1)-P(2)	157.28(3)
N(1)-C(11)-C(12)	114.3(3)		

Table S3 Torsion angles [°] for 5.

N(1)-C(1)-C(2)-P(1)	18.2(4)	C(5)-C(3)-P(1)-C(2)	-49.6(3)
N(1)-C(11)-C(12)-P(2)	23.4(4)	C(4)-C(3)-P(1)-C(7)	-44.2(3)
C(12)-C(11)-N(1)-C(1)	-164.5(3)	C(6)-C(3)-P(1)-C(7)	78.2(3)
C(12)-C(11)-N(1)-W(1)	14.4(4)	C(5)-C(3)-P(1)-C(7)	-162.4(2)
C(2)-C(1)-N(1)-C(11)	-161.3(3)	C(4)-C(3)-P(1)-W(1)	170.9(2)
C(2)-C(1)-N(1)-W(1)	19.7(4)	C(6)-C(3)-P(1)-W(1)	-66.7(3)
C(1)-C(2)-P(1)-C(7)	-158.1(3)	C(5)-C(3)-P(1)-W(1)	52.7(3)
C(1)-C(2)-P(1)-C(3)	85.7(3)	C(11)-C(12)-P(2)-C(13)	84.3(3)
C(1)-C(2)-P(1)-W(1)	-34.2(3)	C(11)-C(12)-P(2)-C(17)	-161.4(3)
C(10)-C(7)-P(1)-C(2)	-163.8(3)	C(11)-C(12)-P(2)-W(1)	-36.7(3)
C(8)-C(7)-P(1)-C(2)	78.5(3)	C(16)-C(13)-P(2)-C(12)	66.1(3)
C(9)-C(7)-P(1)-C(2)	-41.0(3)	C(14)-C(13)-P(2)-C(12)	-170.6(3)
C(10)-C(7)-P(1)-C(3)	-51.2(3)	C(15)-C(13)-P(2)-C(12)	-51.7(3)
C(8)-C(7)-P(1)-C(3)	-168.8(3)	C(16)-C(13)-P(2)-C(17)	-44.7(3)
C(9)-C(7)-P(1)-C(3)	71.7(3)	C(14)-C(13)-P(2)-C(17)	78.6(3)
C(10)-C(7)-P(1)-W(1)	92.2(3)	C(15)-C(13)-P(2)-C(17)	-162.5(3)
C(8)-C(7)-P(1)-W(1)	-25.4(3)	C(16)-C(13)-P(2)-W(1)	166.9(2)
C(9)-C(7)-P(1)-W(1)	-144.9(2)	C(14)-C(13)-P(2)-W(1)	-69.8(3)
C(4)-C(3)-P(1)-C(2)	68.6(3)	C(15)-C(13)-P(2)-W(1)	49.0(3)
C(6)-C(3)-P(1)-C(2)	-169.1(3)		

Crystal Structure of **6**

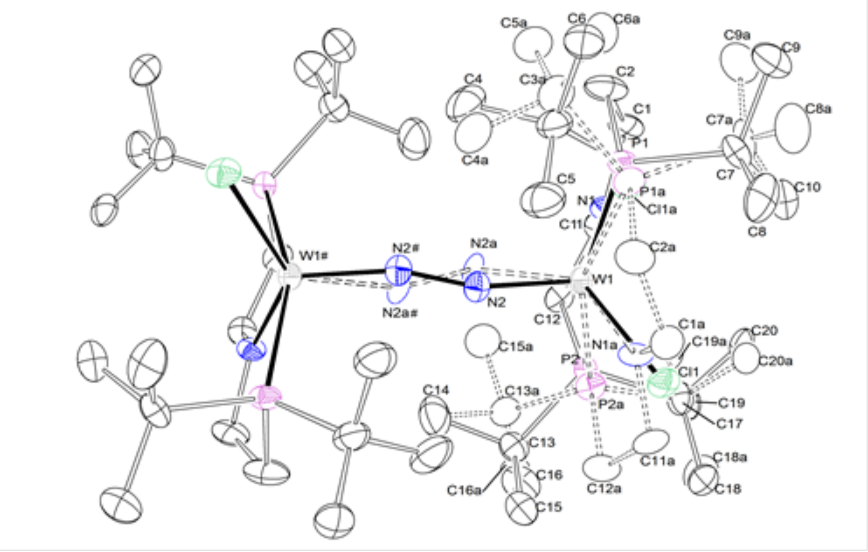

Figure S32 Thermal ellipsoid plot of **6** with the anisotropic displacement parameters drawn at the 50% probability level. The asymmetric unit contains a half disordered complex molecule. The disordered complex molecule was refined with population of 0.670(3) on the main domain using some restraints (SADI, RIGU).

Table S4 Crystal data and structure refinement for 6.

Identification code	mo_CV_BS_150816_0m_a (BS-A-165)	
Empirical formula	$C_{40}H_{88}Cl_2N_4P_4W_2$	
Formula weight	1187.62	
Temperature	100(2) K	
Wavelength	0.71073 Å	
Crystal system	Monoclinic	
Space group	C2/c	
Unit cell dimensions	a = 23.0363(8) Å	α= 90°
	b = 9.8057(3) Å	β= 111.350(2)°
	c = 23.3255(8) Å	γ = 90°
Volume	4907.3(3) Å3	
Z	4	
Density (calculated)	1.607 Mg/m^3	
Absorption coefficient	4.955 mm^{-1}	
F(000)	2392	
Crystal size	0.168 x 0.128 x 0.088 mm^3	
Crystal shape and color	Block, clear intense green	
Theta range for data collection	2.128 to 28.358°	
Index ranges	-30<=h<=30, -13<=k<=13, -30<=l<=31	
Reflections collected	53392	
Independent reflections	6110 [R(int) = 0.0747]	
Completeness to theta = 25.242°	100.0 %	
Max. and min. transmission	0.7457 and 0.5543	
Refinement method	Full-matrix least-squares on F^2	
Data / restraints / parameters	6110 / 217 / 456	
Goodness-of-fit on F2	1.089	
Final R indices [I>2sigma(I)]	R1 = 0.0303,	wR2 = 0.0482
R indices (all data)	R1 = 0.0473,	wR2 = 0.0518
Largest diff. peak and hole	1.434 and -1.253 $eÅ^{-3}$	

Table S5 Bond lengths [Å] and angles [°] for 6.

C(17)-C(18A)	1.487(10)	C(9A)-C(7A)	1.53(3)
C(17)-C(19)	1.505(6)	C(8A)-C(7A)	1.46(4)
C(17)-C(20A)	1.519(12)	C(6A)-C(3A)	1.49(3)
C(17)-C(20)	1.543(8)	C(5A)-C(3A)	1.538(19)
C(17)-C(18)	1.587(6)	C(4A)-C(3A)	1.51(2)
C(17)-C(19A)	1.629(10)	C(15A)-C(13A)	1.56(2)
C(17)-P(2)	1.871(5)	C(16A)-C(13A)	1.49(4)
C(17)-P(2A)	1.888(9)		
C(14)-C(13A)	1.381(14)	C(18A)-C(17)-C(20A)	112.4(10)
C(14)-C(13)	1.504(8)	C(19)-C(17)-C(20)	108.8(7)
C(10)-C(7A)	1.45(4)	C(19)-C(17)-C(18)	108.3(4)
C(10)-C(7)	1.563(15)	C(20)-C(17)-C(18)	104.7(5)
C(1)-N(1)	1.467(7)	C(18A)-C(17)-C(19A)	106.1(7)
C(1)-C(2)	1.528(9)	C(20A)-C(17)-C(19A)	104.5(9)
C(2)-P(1)	1.833(6)	C(19)-C(17)-P(2)	117.1(3)
C(3)-C(5)	1.517(10)	C(20)-C(17)-P(2)	108.1(7)
C(3)-C(6)	1.536(14)	C(18)-C(17)-P(2)	109.2(3)
C(3)-C(4)	1.538(10)	C(18A)-C(17)-P(2A)	119.0(7)
C(3)-P(1)	1.880(9)	C(20A)-C(17)-P(2A)	108.1(14)
C(7)-C(9)	1.544(16)	C(19A)-C(17)-P(2A)	105.4(6)
C(7)-C(8)	1.556(14)	N(1)-C(1)-C(2)	112.2(5)
C(7)-P(1)	1.857(19)	C(1)-C(2)-P(1)	109.7(4)
C(11)-N(1)	1.487(8)	C(5)-C(3)-C(6)	108.5(8)
C(11)-C(12)	1.517(8)	C(5)-C(3)-C(4)	107.2(7)
C(12)-P(2)	1.837(6)	C(6)-C(3)-C(4)	106.4(8)
C(13)-C(15)	1.520(9)	C(5)-C(3)-P(1)	107.0(5)
C(13)-C(16)	1.536(19)	C(6)-C(3)-P(1)	118.6(6)
C(13)-P(2)	1.880(7)	C(4)-C(3)-P(1)	108.7(6)
N(1)-W(1)	1.938(6)	C(9)-C(7)-C(8)	108.6(11)
N(2)-N(2)#1	1.33(4)	C(9)-C(7)-C(10)	108.0(9)
N(2)-W(1)	1.78(2)	C(8)-C(7)-C(10)	105.5(10)
P(1)-W(1)	2.444(4)	C(9)-C(7)-P(1)	115.8(10)
P(2)-W(1)	2.483(4)	C(8)-C(7)-P(1)	110.3(8)
Cl(1)-W(1)	2.5082(16)	C(10)-C(7)-P(1)	108.2(9)
Cl(1A)-W(1)	2.576(4)	N(1)-C(11)-C(12)	112.6(4)
P(1A)-C(2A)	1.837(13)	C(11)-C(12)-P(2)	108.8(4)
P(1A)-C(3A)	1.902(18)	C(14)-C(13)-C(15)	103.2(5)
P(1A)-C(7A)	1.95(3)	C(14)-C(13)-C(16)	110.8(10)
P(1A)-W(1)	2.468(9)	C(15)-C(13)-C(16)	109.7(9)
N(1A)-C(1A)	1.442(16)	C(14)-C(13)-P(2)	109.6(4)
N(1A)-C(11A)	1.492(15)	C(15)-C(13)-P(2)	109.2(4)
N(1A)-W(1)	1.893(11)	C(16)-C(13)-P(2)	113.9(10)
C(1A)-C(2A)	1.536(16)	C(1)-N(1)-C(11)	106.8(5)
N(2A)-N(2A)#1	1.27(8)	C(1)-N(1)-W(1)	126.5(4)
N(2A)-W(1)	1.82(4)	C(11)-N(1)-W(1)	126.7(4)
P(2A)-C(12A)	1.834(12)	N(2)#1-N(2)-W(1)	163.8(18)
P(2A)-C(13A)	1.880(17)	C(2)-P(1)-C(7)	104.1(5)
P(2A)-W(1)	2.417(8)	C(2)-P(1)-C(3)	104.6(4)
C(11A)-C(12A)	1.504(16)	C(7)-P(1)-C(3)	110.3(5)

C(2)-P(1)-W(1)	97.3(2)	C(6A)-C(3A)-C(4A)	114.5(14)
C(7)-P(1)-W(1)	117.4(5)	C(6A)-C(3A)-C(5A)	107.4(17)
C(3)-P(1)-W(1)	119.9(3)	C(4A)-C(3A)-C(5A)	107.0(12)
C(12)-P(2)-C(17)	101.7(3)	C(6A)-C(3A)-P(1A)	111.0(17)
C(12)-P(2)-C(13)	105.4(3)	C(4A)-C(3A)-P(1A)	109.5(12)
C(17)-P(2)-C(13)	110.9(3)	C(5A)-C(3A)-P(1A)	107.0(10)
C(12)-P(2)-W(1)	96.2(2)	C(11A)-C(12A)-P(2A)	109.1(8)
C(17)-P(2)-W(1)	117.5(2)	C(14)-C(13A)-C(16A)	111(2)
C(13)-P(2)-W(1)	120.8(2)	C(14)-C(13A)-C(15A)	92.2(10)
C(2A)-P(1A)-C(3A)	102.8(8)	C(16A)-C(13A)-C(15A)	107(2)
C(2A)-P(1A)-C(7A)	104.2(11)	C(14)-C(13A)-P(2A)	118.2(10)
C(3A)-P(1A)-C(7A)	110.8(12)	C(16A)-C(13A)-P(2A)	117(2)
C(2A)-P(1A)-W(1)	95.8(5)	C(15A)-C(13A)-P(2A)	107.3(10)
C(3A)-P(1A)-W(1)	121.0(6)	N(2A)-W(1)-N(1A)	118.0(8)
C(7A)-P(1A)-W(1)	117.9(11)	N(2)-W(1)-N(1)	114.9(4)
C(1A)-N(1A)-C(11A)	107.2(9)	N(2A)-W(1)-P(2A)	101.8(13)
C(1A)-N(1A)-W(1)	128.4(9)	N(1A)-W(1)-P(2A)	83.4(4)
C(11A)-N(1A)-W(1)	124.2(8)	N(2)-W(1)-P(1)	99.2(7)
N(1A)-C(1A)-C(2A)	112.7(10)	N(1)-W(1)-P(1)	81.70(17)
C(1A)-C(2A)-P(1A)	107.8(8)	N(2A)-W(1)-P(1A)	100.3(13)
N(2A)#1-N(2A)-W(1)	160(4)	N(1A)-W(1)-P(1A)	80.3(4)
C(12A)-P(2A)-C(13A)	104.3(7)	P(2A)-W(1)-P(1A)	156.8(3)
C(12A)-P(2A)-C(17)	99.9(5)	N(2)-W(1)-P(2)	101.2(7)
C(13A)-P(2A)-C(17)	111.8(6)	N(1)-W(1)-P(2)	80.08(17)
C(12A)-P(2A)-W(1)	96.7(4)	P(1)-W(1)-P(2)	156.91(12)
C(13A)-P(2A)-W(1)	119.1(6)	N(2)-W(1)-Cl(1)	109.4(3)
C(17)-P(2A)-W(1)	119.9(4)	N(1)-W(1)-Cl(1)	135.67(16)
N(1A)-C(11A)-C(12A)	114.1(9)	P(1)-W(1)-Cl(1)	93.53(8)
C(10)-C(7A)-C(8A)	112(3)	P(2)-W(1)-Cl(1)	89.68(8)
C(10)-C(7A)-C(9A)	103.7(18)	N(2A)-W(1)-Cl(1A)	106.2(7)
C(8A)-C(7A)-C(9A)	115(2)	N(1A)-W(1)-Cl(1A)	135.7(4)
C(10)-C(7A)-P(1A)	106(2)	P(2A)-W(1)-Cl(1A)	91.08(17)
C(8A)-C(7A)-P(1A)	112.4(17)	P(1A)-W(1)-Cl(1A)	89.26(18)
C(9A)-C(7A)-P(1A)	108(2)		

Symmetry transformations used to generate equivalent atoms:
#1 -x+1,y,-z+3/2

Table S6 Torsion angles [°] for 6.

N(1)-C(1)-C(2)-P(1)	36.6(6)	C(8)-C(7)-P(1)-C(2)	165.2(8)
N(1)-C(11)-C(12)-P(2)	-36.6(6)	C(10)-C(7)-P(1)-C(2)	-79.9(7)
C(2)-C(1)-N(1)-C(11)	158.9(5)	C(9)-C(7)-P(1)-C(3)	-70.2(9)
C(2)-C(1)-N(1)-W(1)	-18.5(7)	C(8)-C(7)-P(1)-C(3)	53.5(10)
C(12)-C(11)-N(1)-C(1)	-166.6(5)	C(10)-C(7)-P(1)-C(3)	168.4(5)
C(12)-C(11)-N(1)-W(1)	10.8(7)	C(9)-C(7)-P(1)-W(1)	147.6(7)
C(1)-C(2)-P(1)-C(7)	85.9(7)	C(8)-C(7)-P(1)-W(1)	-88.7(9)
C(1)-C(2)-P(1)-C(3)	-158.3(5)	C(10)-C(7)-P(1)-W(1)	26.2(8)
C(1)-C(2)-P(1)-W(1)	-34.8(5)	C(5)-C(3)-P(1)-C(2)	163.7(5)
C(9)-C(7)-P(1)-C(2)	41.5(9)	C(6)-C(3)-P(1)-C(2)	-73.3(9)

C(4)-C(3)-P(1)-C(2)	48.3(6)	C(18A)-C(17)-P(2A)-C(13A)	-64.5(9)
C(5)-C(3)-P(1)-C(7)	-84.8(7)	C(20A)-C(17)-P(2A)-C(13A)	165.7(10)
C(6)-C(3)-P(1)-C(7)	38.1(10)	C(19A)-C(17)-P(2A)-C(13A)	54.4(8)
C(4)-C(3)-P(1)-C(7)	159.7(6)	C(18A)-C(17)-P(2A)-W(1)	149.0(7)
C(5)-C(3)-P(1)-W(1)	56.3(6)	C(20A)-C(17)-P(2A)-W(1)	19.2(9)
C(6)-C(3)-P(1)-W(1)	179.2(8)	C(19A)-C(17)-P(2A)-W(1)	-92.1(6)
C(4)-C(3)-P(1)-W(1)	-59.2(6)	C(1A)-N(1A)-C(11A)-C(12A)	161.6(10)
C(11)-C(12)-P(2)-C(17)	-80.3(4)	W(1)-N(1A)-C(11A)-C(12A)	-14.7(15)
C(11)-C(12)-P(2)-C(13)	163.9(4)	N(1A)-C(11A)-C(12A)-P(2A)	35.2(13)
C(11)-C(12)-P(2)-W(1)	39.6(4)	C(13A)-P(2A)-C(12A)-C(11A)	-157.3(9)
C(19)-C(17)-P(2)-C(12)	-38.2(5)	C(17)-P(2A)-C(12A)-C(11A)	87.1(9)
C(20)-C(17)-P(2)-C(12)	85.0(5)	W(1)-P(2A)-C(12A)-C(11A)	-35.0(9)
C(18)-C(17)-P(2)-C(12)	-161.7(4)	C(12A)-P(2A)-C(13A)-C(14)	63.5(12)
C(19)-C(17)-P(2)-C(13)	73.4(5)	C(17)-P(2A)-C(13A)-C(14)	170.5(9)
C(20)-C(17)-P(2)-C(13)	-163.4(5)	W(1)-P(2A)-C(13A)-C(14)	-42.7(13)
C(18)-C(17)-P(2)-C(13)	-50.0(4)	C(12A)-P(2A)-C(13A)-C(16A)	-74(2)
C(19)-C(17)-P(2)-W(1)	-141.7(4)	C(17)-P(2A)-C(13A)-C(16A)	33(2)
C(20)-C(17)-P(2)-W(1)	-18.4(5)	W(1)-P(2A)-C(13A)-C(16A)	-180(2)
C(18)-C(17)-P(2)-W(1)	94.9(3)	C(12A)-P(2A)-C(13A)-C(15A)	165.8(9)
C(14)-C(13)-P(2)-C(12)	-58.6(5)	C(17)-P(2A)-C(13A)-C(15A)	-87.2(10)
C(15)-C(13)-P(2)-C(12)	-170.8(4)	W(1)-P(2A)-C(13A)-C(15A)	59.6(11)
C(16)-C(13)-P(2)-C(12)	66.2(9)	N(2)#1-N(2)-W(1)-N(1)	-25(4)
C(14)-C(13)-P(2)-C(17)	-167.9(4)	N(2)#1-N(2)-W(1)-P(1)	60(4)
C(15)-C(13)-P(2)-C(17)	79.8(5)	N(2)#1-N(2)-W(1)-P(2)	-110(4)
C(16)-C(13)-P(2)-C(17)	-43.2(9)	N(2)#1-N(2)-W(1)-Cl(1)	157(4)
C(14)-C(13)-P(2)-W(1)	48.5(5)	N(2A)#1-N(2A)-W(1)-N(1A)	-37(5)
C(15)-C(13)-P(2)-W(1)	-63.8(5)	N(2A)#1-N(2A)-W(1)-P(2A)	51(4)
C(16)-C(13)-P(2)-W(1)	173.2(9)	N(2A)#1-N(2A)-W(1)-P(1A)	-122(4)
C(11A)-N(1A)-C(1A)-C(2A)	-165.7(10)	N(2A)#1-N(2A)-W(1)-Cl(1A)	146(4)
W(1)-N(1A)-C(1A)-C(2A)	10.5(16)	C(1A)-N(1A)-W(1)-N(2A)	-83.6(19)
N(1A)-C(1A)-C(2A)-P(1A)	-35.6(13)	C(11A)-N(1A)-W(1)-N(2A)	92.0(18)
C(3A)-P(1A)-C(2A)-C(1A)	161.7(9)	C(1A)-N(1A)-W(1)-P(2A)	176.5(12)
C(7A)-P(1A)-C(2A)-C(1A)	-82.7(14)	C(11A)-N(1A)-W(1)-P(2A)	-7.9(10)
W(1)-P(1A)-C(2A)-C(1A)	38.0(9)	C(1A)-N(1A)-W(1)-P(1A)	13.0(11)
C(18A)-C(17)-P(2A)-C(12A)	45.4(9)	C(11A)-N(1A)-W(1)-P(1A)	-171.4(11)
C(20A)-C(17)-P(2A)-C(12A)	-84.5(10)	C(1A)-N(1A)-W(1)-Cl(1A)	91.8(12)
C(19A)-C(17)-P(2A)-C(12A)	164.3(7)	C(11A)-N(1A)-W(1)-Cl(1A)	-92.6(11)

Symmetry transformations used to generate equivalent atoms:
#1 -x+1,y,-z+3/2

Crystal Structure of **7-OTf**

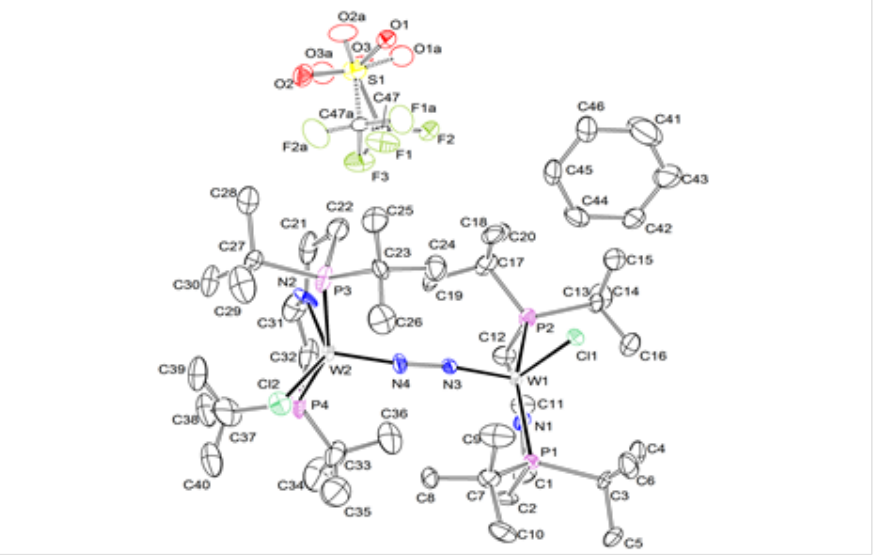

Figure S33 Thermal ellipsoid plot of **7-OTf** with the anisotropic displacement parameters drawn at the 50% probability level. The asymmetric unit contains one complex molecule, one benzene solvent molecule and one disordered $CF_3SO_3^-$ anion with a population of 0.67(1) on the main domain. The structure was refined as twin using the twin law -100 010 00-1 (BASF: 0.47(1)) and some restraints and constrains (SADI, RIGU, EADP).

Table S7 Crystal data and structure refinement for 7-OTf.

Identification code	CW_BS_170118_2_a (BS-C-116)	
Empirical formula	$C_{47}H_{94}Cl_2F_3N_4O_3P_4SW_2$	
Formula weight	1414.80	
Temperature	100(2) K	
Wavelength	0.71073 Å	
Crystal system	Orthorhombic	
Space group	$Pna2_1$	
Unit cell dimensions	a = 19.7515(9) Å	α = 90°
	b = 13.2485(6) Å	β = 90°
	c = 22.2689(10) Å	γ = 90°
Volume	5827.3(5) $Å^3$	
Z	4	
Density (calculated)	1.613 Mg/m^3	
Absorption coefficient	4.232 mm^{-1}	
F(000)	2852	
Crystal size	0.370 x 0.096 x 0.033 mm^3	
Crystal shape and color	Needle, clear intense brown	
Theta range for data collection	2.256 to 28.397°	
Index ranges	-26<=h<=26, -17<=k<=17, -29<=l<=29	
Reflections collected	175705	
Independent reflections	14544 [R(int) = 0.1450]	
Completeness to theta = 25.242°	99.9 %	
Refinement method	Full-matrix least-squares on F^2	
Data / restraints / parameters	14544 / 232 / 651	
Goodness-of-fit on F2	1.081	
Final R indices [I>2sigma(I)]	R1 = 0.0513,	wR2 = 0.0968
R indices (all data)	R1 = 0.0683,	wR2 = 0.1029
Largest diff. peak and hole	2.428 and -1.729 $eÅ^{-3}$	

Table S8 Bond lengths [Å] and angles [°] 7-OTf.

C(1)-N(1)	1.463(13)	N(2)-W(2)	2.093(13)
C(1)-C(2)	1.553(19)	N(3)-N(4)	1.266(12)
C(2)-P(1)	1.835(13)	N(3)-W(1)	1.781(9)
C(3)-C(6)	1.517(19)	N(4)-W(2)	1.813(10)
C(3)-C(4)	1.524(18)	P(1)-W(1)	2.524(3)
C(3)-C(5)	1.537(18)	P(2)-W(1)	2.522(3)
C(3)-P(1)	1.886(12)	P(3)-W(2)	2.504(4)
C(7)-C(8)	1.507(18)	P(4)-W(2)	2.471(3)
C(7)-C(10)	1.532(18)	S(1)-O(3A)	1.31(3)
C(7)-C(9)	1.542(19)	S(1)-O(2A)	1.35(3)
C(7)-P(1)	1.869(12)	S(1)-O(3)	1.397(16)
C(11)-N(1)	1.456(13)	S(1)-O(1)	1.415(15)
C(11)-C(12)	1.533(18)	S(1)-O(2)	1.443(15)
C(12)-P(2)	1.827(13)	S(1)-O(1A)	1.45(3)
C(13)-C(15)	1.52(2)	S(1)-C(47A)	1.74(4)
C(13)-C(16)	1.532(18)	S(1)-C(47)	1.820(18)
C(13)-C(14)	1.535(19)	C(47)-F(1)	1.27(3)
C(13)-P(2)	1.903(13)	C(47)-F(2)	1.36(2)
C(17)-C(20)	1.516(18)	C(47A)-F(2A)	1.29(5)
C(17)-C(19)	1.52(2)	C(47A)-F(1A)	1.41(5)
C(17)-C(18)	1.55(2)		
C(17)-P(2)	1.867(14)	N(1)-C(1)-C(2)	113.1(10)
C(21)-N(2)	1.416(16)	C(1)-C(2)-P(1)	108.1(9)
C(21)-C(22)	1.56(2)	C(6)-C(3)-C(4)	106.7(11)
C(22)-P(3)	1.871(15)	C(6)-C(3)-C(5)	107.1(12)
C(23)-C(24)	1.506(18)	C(4)-C(3)-C(5)	110.1(12)
C(23)-C(25)	1.517(18)	C(6)-C(3)-P(1)	111.5(10)
C(23)-C(26)	1.57(2)	C(4)-C(3)-P(1)	109.7(9)
C(23)-P(3)	1.848(13)	C(5)-C(3)-P(1)	111.7(9)
C(27)-C(30)	1.51(2)	C(8)-C(7)-C(10)	108.4(11)
C(27)-C(29)	1.52(2)	C(8)-C(7)-C(9)	107.7(13)
C(27)-C(28)	1.58(2)	C(10)-C(7)-C(9)	110.4(11)
C(27)-P(3)	1.886(13)	C(8)-C(7)-P(1)	108.8(8)
C(31)-N(2)	1.399(15)	C(10)-C(7)-P(1)	113.6(10)
C(31)-C(32)	1.50(2)	C(9)-C(7)-P(1)	107.8(9)
C(32)-P(4)	1.891(18)	N(1)-C(11)-C(12)	114.4(10)
C(33)-C(36)	1.523(16)	C(11)-C(12)-P(2)	108.2(9)
C(33)-C(34)	1.531(16)	C(15)-C(13)-C(16)	106.9(12)
C(33)-C(35)	1.545(17)	C(15)-C(13)-C(14)	111.3(12)
C(33)-P(4)	1.843(15)	C(16)-C(13)-C(14)	108.8(11)
C(37)-C(39)	1.519(18)	C(15)-C(13)-P(2)	110.4(9)
C(37)-C(38)	1.527(17)	C(16)-C(13)-P(2)	107.6(9)
C(37)-C(40)	1.541(18)	C(14)-C(13)-P(2)	111.6(10)
C(37)-P(4)	1.854(18)	C(20)-C(17)-C(19)	108.4(12)
Cl(1)-W(1)	2.366(3)	C(20)-C(17)-C(18)	109.7(13)
Cl(2)-W(2)	2.446(3)	C(19)-C(17)-C(18)	106.8(12)
F(3)-C(47)	1.29(2)	C(20)-C(17)-P(2)	114.4(10)
F(3)-C(47A)	1.65(5)	C(19)-C(17)-P(2)	109.0(10)
N(1)-W(1)	1.993(10)	C(18)-C(17)-P(2)	108.2(10)

N(2)-C(21)-C(22)	120.5(12)	C(22)-P(3)-C(27)	104.0(7)
C(21)-C(22)-P(3)	105.7(11)	C(23)-P(3)-W(2)	118.4(5)
C(24)-C(23)-C(25)	109.0(11)	C(22)-P(3)-W(2)	97.7(6)
C(24)-C(23)-C(26)	105.0(12)	C(27)-P(3)-W(2)	117.6(5)
C(25)-C(23)-C(26)	109.8(12)	C(33)-P(4)-C(37)	113.2(7)
C(24)-C(23)-P(3)	111.4(10)	C(33)-P(4)-C(32)	105.8(7)
C(25)-C(23)-P(3)	114.8(10)	C(37)-P(4)-C(32)	101.0(7)
C(26)-C(23)-P(3)	106.3(9)	C(33)-P(4)-W(2)	120.5(4)
C(30)-C(27)-C(29)	106.9(12)	C(37)-P(4)-W(2)	116.6(6)
C(30)-C(27)-C(28)	110.8(13)	C(32)-P(4)-W(2)	94.7(5)
C(29)-C(27)-C(28)	106.6(13)	O(3A)-S(1)-O(2A)	111(2)
C(30)-C(27)-P(3)	109.7(10)	O(3)-S(1)-O(1)	117.6(11)
C(29)-C(27)-P(3)	111.2(10)	O(3)-S(1)-O(2)	113.5(11)
C(28)-C(27)-P(3)	111.4(10)	O(1)-S(1)-O(2)	114.2(9)
N(2)-C(31)-C(32)	114.6(13)	O(3A)-S(1)-O(1A)	103(2)
C(31)-C(32)-P(4)	111.6(10)	O(2A)-S(1)-O(1A)	104.6(19)
C(36)-C(33)-C(34)	104.1(13)	O(3A)-S(1)-C(47A)	112(2)
C(36)-C(33)-C(35)	109.2(14)	O(2A)-S(1)-C(47A)	112(2)
C(34)-C(33)-C(35)	113.8(15)	O(1A)-S(1)-C(47A)	113(2)
C(36)-C(33)-P(4)	108.9(11)	O(3)-S(1)-C(47)	103.5(10)
C(34)-C(33)-P(4)	114.9(11)	O(1)-S(1)-C(47)	103.9(9)
C(35)-C(33)-P(4)	105.7(11)	O(2)-S(1)-C(47)	101.5(9)
C(39)-C(37)-C(38)	106.8(15)	N(3)-W(1)-N(1)	114.2(4)
C(39)-C(37)-C(40)	103.2(16)	N(3)-W(1)-Cl(1)	108.9(3)
C(38)-C(37)-C(40)	111.6(14)	N(1)-W(1)-Cl(1)	136.8(3)
C(39)-C(37)-P(4)	109.4(11)	N(3)-W(1)-P(2)	101.1(3)
C(38)-C(37)-P(4)	116.9(13)	N(1)-W(1)-P(2)	79.8(3)
C(40)-C(37)-P(4)	108.2(11)	Cl(1)-W(1)-P(2)	93.38(11)
C(11)-N(1)-C(1)	108.5(10)	N(3)-W(1)-P(1)	100.4(3)
C(11)-N(1)-W(1)	125.2(8)	N(1)-W(1)-P(1)	79.7(3)
C(1)-N(1)-W(1)	126.3(8)	Cl(1)-W(1)-P(1)	91.69(10)
C(31)-N(2)-C(21)	114.4(13)	P(2)-W(1)-P(1)	154.94(10)
C(31)-N(2)-W(2)	124.1(10)	N(4)-W(2)-N(2)	113.0(5)
C(21)-N(2)-W(2)	120.8(9)	N(4)-W(2)-Cl(2)	113.2(4)
N(4)-N(3)-W(1)	169.7(8)	N(2)-W(2)-Cl(2)	133.7(3)
N(3)-N(4)-W(2)	172.3(10)	N(4)-W(2)-P(4)	102.2(3)
C(2)-P(1)-C(7)	106.6(6)	N(2)-W(2)-P(4)	80.7(3)
C(2)-P(1)-C(3)	106.0(6)	Cl(2)-W(2)-P(4)	92.29(12)
C(7)-P(1)-C(3)	112.2(6)	N(4)-W(2)-P(3)	100.8(3)
C(2)-P(1)-W(1)	96.6(4)	N(2)-W(2)-P(3)	80.4(3)
C(7)-P(1)-W(1)	117.6(4)	Cl(2)-W(2)-P(3)	88.55(13)
C(3)-P(1)-W(1)	115.4(4)	P(4)-W(2)-P(3)	154.55(12)
C(12)-P(2)-C(17)	104.8(7)	F(1)-C(47)-F(3)	110.4(17)
C(12)-P(2)-C(13)	106.9(7)	F(1)-C(47)-F(2)	107.6(17)
C(17)-P(2)-C(13)	111.3(6)	F(3)-C(47)-F(2)	101.7(17)
C(12)-P(2)-W(1)	96.5(4)	F(1)-C(47)-S(1)	113.6(16)
C(17)-P(2)-W(1)	119.2(4)	F(3)-C(47)-S(1)	113.3(13)
C(13)-P(2)-W(1)	115.6(5)	F(2)-C(47)-S(1)	109.3(13)
C(23)-P(3)-C(22)	103.2(7)	F(2A)-C(47A)-F(1A)	113(4)
C(23)-P(3)-C(27)	112.2(6)	F(2A)-C(47A)-F(3)	101(3)

F(1A)-C(47A)-F(3)	126(3)	F(1A)-C(47A)-S(1)	107(3)
F(2A)-C(47A)-S(1)	108(3)	F(3)-C(47A)-S(1)	101(2)

Table S9 Torsion angles [°] for 7-OTf.

N(1)-C(1)-C(2)-P(1)	-40.1(13)	C(25)-C(23)-P(3)-C(22)	72.8(12)
N(1)-C(11)-C(12)-P(2)	40.2(14)	C(26)-C(23)-P(3)-C(22)	-165.5(10)
N(2)-C(21)-C(22)-P(3)	-35.6(16)	C(24)-C(23)-P(3)-C(27)	-163.0(10)
N(2)-C(31)-C(32)-P(4)	35.7(18)	C(25)-C(23)-P(3)-C(27)	-38.5(13)
C(12)-C(11)-N(1)-C(1)	161.5(11)	C(26)-C(23)-P(3)-C(27)	83.2(11)
C(12)-C(11)-N(1)-W(1)	-17.1(16)	C(24)-C(23)-P(3)-W(2)	54.8(11)
C(2)-C(1)-N(1)-C(11)	-161.7(11)	C(25)-C(23)-P(3)-W(2)	179.4(9)
C(2)-C(1)-N(1)-W(1)	17.0(16)	C(26)-C(23)-P(3)-W(2)	-59.0(10)
C(32)-C(31)-N(2)-C(21)	160.8(13)	C(21)-C(22)-P(3)-C(23)	159.2(10)
C(32)-C(31)-N(2)-W(2)	-10.4(19)	C(21)-C(22)-P(3)-C(27)	-83.5(11)
C(22)-C(21)-N(2)-C(31)	-161.2(13)	C(21)-C(22)-P(3)-W(2)	37.6(10)
C(22)-C(21)-N(2)-W(2)	10.2(17)	C(30)-C(27)-P(3)-C(23)	-166.5(10)
C(1)-C(2)-P(1)-C(7)	160.9(9)	C(29)-C(27)-P(3)-C(23)	-48.4(12)
C(1)-C(2)-P(1)-C(3)	-79.4(10)	C(28)-C(27)-P(3)-C(23)	70.4(12)
C(1)-C(2)-P(1)-W(1)	39.5(9)	C(30)-C(27)-P(3)-C(22)	82.6(12)
C(8)-C(7)-P(1)-C(2)	-46.5(10)	C(29)-C(27)-P(3)-C(22)	-159.2(11)
C(10)-C(7)-P(1)-C(2)	74.4(11)	C(28)-C(27)-P(3)-C(22)	-40.4(13)
C(9)-C(7)-P(1)-C(2)	-163.0(11)	C(30)-C(27)-P(3)-W(2)	-24.1(12)
C(8)-C(7)-P(1)-C(3)	-162.1(9)	C(29)-C(27)-P(3)-W(2)	94.1(11)
C(10)-C(7)-P(1)-C(3)	-41.2(12)	C(28)-C(27)-P(3)-W(2)	-147.1(10)
C(9)-C(7)-P(1)-C(3)	81.4(11)	C(36)-C(33)-P(4)-C(37)	161.7(11)
C(8)-C(7)-P(1)-W(1)	60.5(10)	C(34)-C(33)-P(4)-C(37)	45.4(16)
C(10)-C(7)-P(1)-W(1)	-178.7(8)	C(35)-C(33)-P(4)-C(37)	-81.0(12)
C(9)-C(7)-P(1)-W(1)	-56.1(11)	C(36)-C(33)-P(4)-C(32)	52.0(12)
C(6)-C(3)-P(1)-C(2)	-161.1(9)	C(34)-C(33)-P(4)-C(32)	-64.3(15)
C(4)-C(3)-P(1)-C(2)	81.0(10)	C(35)-C(33)-P(4)-C(32)	169.3(10)
C(5)-C(3)-P(1)-C(2)	-41.3(12)	C(36)-C(33)-P(4)-W(2)	-53.5(12)
C(6)-C(3)-P(1)-C(7)	-45.2(11)	C(34)-C(33)-P(4)-W(2)	-169.8(12)
C(4)-C(3)-P(1)-C(7)	-163.1(9)	C(35)-C(33)-P(4)-W(2)	63.8(11)
C(5)-C(3)-P(1)-C(7)	74.6(12)	C(39)-C(37)-P(4)-C(33)	165.2(11)
C(6)-C(3)-P(1)-W(1)	93.3(10)	C(38)-C(37)-P(4)-C(33)	-73.4(16)
C(4)-C(3)-P(1)-W(1)	-24.7(11)	C(40)-C(37)-P(4)-C(33)	53.4(14)
C(5)-C(3)-P(1)-W(1)	-146.9(9)	C(39)-C(37)-P(4)-C(32)	-82.2(13)
C(11)-C(12)-P(2)-C(17)	-161.6(9)	C(38)-C(37)-P(4)-C(32)	39.2(16)
C(11)-C(12)-P(2)-C(13)	80.1(10)	C(40)-C(37)-P(4)-C(32)	166.1(12)
C(11)-C(12)-P(2)-W(1)	-39.1(9)	C(39)-C(37)-P(4)-W(2)	18.9(14)
C(20)-C(17)-P(2)-C(12)	-72.6(13)	C(38)-C(37)-P(4)-W(2)	140.3(13)
C(19)-C(17)-P(2)-C(12)	49.0(12)	C(40)-C(37)-P(4)-W(2)	-92.9(12)
C(18)-C(17)-P(2)-C(12)	164.8(9)	C(31)-C(32)-P(4)-C(33)	-161.8(11)
C(20)-C(17)-P(2)-C(13)	42.6(14)	C(31)-C(32)-P(4)-C(37)	80.0(12)
C(19)-C(17)-P(2)-C(13)	164.2(10)	C(31)-C(32)-P(4)-W(2)	-38.2(11)
C(18)-C(17)-P(2)-C(13)	-80.0(10)	N(4)-N(3)-W(1)-N(1)	11(5)
C(20)-C(17)-P(2)-W(1)	-178.9(10)	N(4)-N(3)-W(1)-Cl(1)	-168(5)
C(19)-C(17)-P(2)-W(1)	-57.3(12)	N(4)-N(3)-W(1)-P(2)	95(5)
C(18)-C(17)-P(2)-W(1)	58.5(11)	N(4)-N(3)-W(1)-P(1)	-72(5)
C(24)-C(23)-P(3)-C(22)	-51.7(11)	O(3)-S(1)-C(47)-F(1)	-179.5(17)

O(1)-S(1)-C(47)-F(1)	57.2(18)	O(2A)-S(1)-C(47A)-F(2A)	61(4)
O(2)-S(1)-C(47)-F(1)	-61.6(18)	O(1A)-S(1)-C(47A)-F(2A)	179(3)
O(3)-S(1)-C(47)-F(3)	-52.4(19)	O(3A)-S(1)-C(47A)-F(1A)	174(3)
O(1)-S(1)-C(47)-F(3)	-175.8(15)	O(2A)-S(1)-C(47A)-F(1A)	-61(4)
O(2)-S(1)-C(47)-F(3)	65.4(17)	O(1A)-S(1)-C(47A)-F(1A)	57(4)
O(3)-S(1)-C(47)-F(2)	60.3(17)	O(3A)-S(1)-C(47A)-F(3)	40(3)
O(1)-S(1)-C(47)-F(2)	-63.1(16)	O(2A)-S(1)-C(47A)-F(3)	166(2)
O(2)-S(1)-C(47)-F(2)	178.1(14)	O(1A)-S(1)-C(47A)-F(3)	-76(2)
O(3A)-S(1)-C(47A)-F(2A)	-65(4)		

Crystal Structure of **8-(BPh$_4$)$_2$**

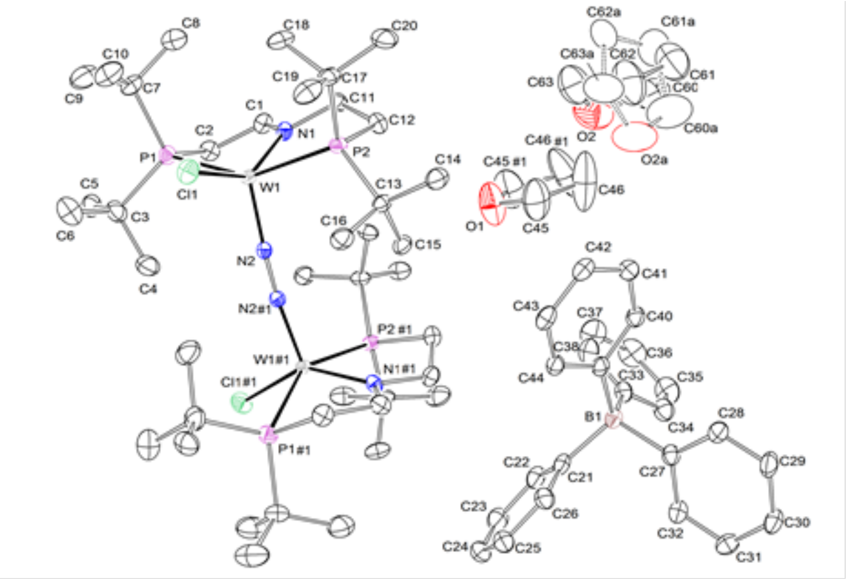

Figure S34 Thermal ellipsoid plot of **8-(BPh$_4$)$_2$** with the anisotropic displacement parameters drawn at the 50% probability level. The asymmetric unit contains a half complex molecule, a half THF solvent molecule, one BPh$_4$ anion and one disordered THF solvent molecule. The disordered THF molecule was refined with population of 0.506(6) on the main domain using some restraints (SADI, RIGU).

Table S10 Crystal data and structure refinement for 8-(BPh$_4$)$_2$.

Identification code	mo_CW_BS_181217_0m_a (BS-C-103)	
Empirical formula	$C_{100}H_{152}B_2Cl_2N_4O_3P_4W_2$	
Formula weight	2042.35	
Temperature	101(2) K	
Wavelength	0.71073 Å	
Crystal system	Monoclinic	
Space group	C2/c	
Unit cell dimensions	a = 35.4758(14) Å	α = 90°
	b = 14.6906(6) Å	β = 131.4330(10)°
	c = 24.6143(17) Å	γ = 90°
Volume	9617.5(9) Å^3	
Z	4	
Density (calculated)	1.411 Mg/m^3	
Absorption coefficient	2.563 mm^{-1}	
F(000)	4224	
Crystal size	0.200 x 0.101 x 0.076 mm^3	
Crystal shape and color	Plate, clear intense yellow-brown	
Theta range for data collection	2.201 to 30.605°	
Index ranges	-50<=h<=50, -20<=k<=20, -34<=l<=35	
Reflections collected	139562	
Independent reflections	14725 [R(int) = 0.1073]	
Completeness to theta = 25.242°	100.0 %	
Refinement method	Full-matrix least-squares on F^2	
Data / restraints / parameters	14725 / 90 / 586	
Goodness-of-fit on F2	1.018	
Final R indices [I>2sigma(I)]	R1 = 0.0373,	wR2 = 0.0618
R indices (all data)	R1 = 0.0714,	wR2 = 0.0700
Largest diff. peak and hole	1.601 and -1.376 eÅ^{-3}	

Table S11 Bond lengths [Å] and angles [°] for 8-$(BPh_4)_2$.

W(1)-N(2)	1.785(2)	C(37)-C(38)	1.391(5)
W(1)-N(1)	1.983(3)	C(39)-C(44)	1.400(4)
W(1)-Cl(1)	2.3507(8)	C(39)-C(40)	1.406(4)
W(1)-P(2)	2.5269(8)	C(40)-C(41)	1.385(4)
W(1)-P(1)	2.5535(8)	C(41)-C(42)	1.380(5)
C(1)-N(1)	1.481(4)	C(42)-C(43)	1.378(5)
C(1)-C(2)	1.524(4)	C(43)-C(44)	1.391(4)
B(1)-C(39)	1.642(5)	O(1)-C(45)#1	1.397(5)
B(1)-C(33)	1.641(5)	O(1)-C(45)	1.397(5)
B(1)-C(21)	1.646(5)	C(45)-C(46)	1.471(7)
B(1)-C(27)	1.654(5)	C(46)-C(46)#1	1.391(11)
P(1)-C(2)	1.826(3)	O(2)-C(63)	1.405(10)
P(1)-C(3)	1.872(4)	O(2)-C(60)	1.426(10)
P(1)-C(7)	1.890(3)	C(60)-C(61)	1.495(11)
N(1)-C(11)	1.475(4)	C(61)-C(62)	1.517(12)
P(2)-C(12)	1.833(3)	C(62)-C(63)	1.490(12)
P(2)-C(17)	1.877(3)	O(2A)-C(60A)	1.398(11)
P(2)-C(13)	1.878(3)	O(2A)-C(63A)	1.418(12)
N(2)-N(2)#1	1.277(5)	C(60A)-C(61A)	1.501(11)
C(7)-C(9)	1.528(5)	C(61A)-C(62A)	1.499(11)
C(7)-C(8)	1.537(5)	C(62A)-C(63A)	1.521(11)
C(7)-C(10)	1.539(5)		
C(6)-C(3)	1.552(5)	N(2)-W(1)-N(1)	111.71(10)
C(5)-C(3)	1.540(5)	N(2)-W(1)-Cl(1)	105.53(8)
C(4)-C(3)	1.520(5)	N(1)-W(1)-Cl(1)	142.77(7)
C(12)-C(11)	1.533(4)	N(2)-W(1)-P(2)	100.52(8)
C(13)-C(15)	1.527(4)	N(1)-W(1)-P(2)	80.15(7)
C(13)-C(14)	1.532(4)	Cl(1)-W(1)-P(2)	93.40(3)
C(13)-C(16)	1.534(4)	N(2)-W(1)-P(1)	106.42(8)
C(18)-C(17)	1.536(4)	N(1)-W(1)-P(1)	78.30(7)
C(17)-C(20)	1.521(5)	Cl(1)-W(1)-P(1)	91.45(3)
C(17)-C(19)	1.544(5)	P(2)-W(1)-P(1)	150.21(3)
C(21)-C(22)	1.400(4)	N(1)-C(1)-C(2)	114.4(3)
C(21)-C(26)	1.405(5)	C(39)-B(1)-C(33)	108.7(3)
C(22)-C(23)	1.385(5)	C(39)-B(1)-C(21)	112.9(3)
C(23)-C(24)	1.379(5)	C(33)-B(1)-C(21)	109.0(3)
C(24)-C(25)	1.388(5)	C(39)-B(1)-C(27)	110.3(3)
C(25)-C(26)	1.389(5)	C(33)-B(1)-C(27)	109.5(3)
C(27)-C(32)	1.400(4)	C(21)-B(1)-C(27)	106.4(3)
C(27)-C(28)	1.402(5)	C(2)-P(1)-C(3)	106.68(16)
C(28)-C(29)	1.390(5)	C(2)-P(1)-C(7)	106.55(15)
C(29)-C(30)	1.382(5)	C(3)-P(1)-C(7)	111.63(16)
C(30)-C(31)	1.375(5)	C(2)-P(1)-W(1)	95.61(10)
C(31)-C(32)	1.385(4)	C(3)-P(1)-W(1)	116.98(11)
C(33)-C(38)	1.401(5)	C(7)-P(1)-W(1)	116.95(11)
C(33)-C(34)	1.401(5)	C(11)-N(1)-C(1)	107.3(2)
C(34)-C(35)	1.393(5)	C(11)-N(1)-W(1)	126.76(19)
C(35)-C(36)	1.380(6)	C(1)-N(1)-W(1)	125.9(2)
C(36)-C(37)	1.392(6)	C(12)-P(2)-C(17)	106.20(15)

C(12)-P(2)-C(13)	107.45(14)	C(24)-C(25)-C(26)	119.8(3)
C(17)-P(2)-C(13)	112.12(14)	C(25)-C(26)-C(21)	123.3(3)
C(12)-P(2)-W(1)	98.28(10)	C(32)-C(27)-C(28)	114.9(3)
C(17)-P(2)-W(1)	114.53(11)	C(32)-C(27)-B(1)	120.8(3)
C(13)-P(2)-W(1)	116.49(10)	C(28)-C(27)-B(1)	124.2(3)
C(1)-C(2)-P(1)	110.2(2)	C(29)-C(28)-C(27)	122.6(3)
N(2)#1-N(2)-W(1)	169.7(3)	C(30)-C(29)-C(28)	120.2(3)
C(9)-C(7)-C(8)	107.4(3)	C(31)-C(30)-C(29)	118.9(3)
C(9)-C(7)-C(10)	109.1(3)	C(30)-C(31)-C(32)	120.3(3)
C(8)-C(7)-C(10)	105.9(3)	C(31)-C(32)-C(27)	123.0(3)
C(9)-C(7)-P(1)	113.9(3)	C(38)-C(33)-C(34)	115.3(3)
C(8)-C(7)-P(1)	109.0(2)	C(38)-C(33)-B(1)	120.8(3)
C(10)-C(7)-P(1)	111.2(2)	C(34)-C(33)-B(1)	123.7(3)
C(4)-C(3)-C(5)	108.6(3)	C(35)-C(34)-C(33)	122.7(3)
C(4)-C(3)-C(6)	109.1(3)	C(36)-C(35)-C(34)	120.0(4)
C(5)-C(3)-C(6)	110.3(3)	C(35)-C(36)-C(37)	119.2(3)
C(4)-C(3)-P(1)	107.5(2)	C(36)-C(37)-C(38)	119.8(4)
C(5)-C(3)-P(1)	112.3(2)	C(37)-C(38)-C(33)	122.9(3)
C(6)-C(3)-P(1)	109.0(2)	C(44)-C(39)-C(40)	114.3(3)
C(11)-C(12)-P(2)	110.0(2)	C(44)-C(39)-B(1)	125.3(3)
N(1)-C(11)-C(12)	113.9(2)	C(40)-C(39)-B(1)	120.4(3)
C(15)-C(13)-C(14)	107.8(3)	C(41)-C(40)-C(39)	123.4(3)
C(15)-C(13)-C(16)	107.9(3)	C(42)-C(41)-C(40)	120.2(3)
C(14)-C(13)-C(16)	111.4(3)	C(43)-C(42)-C(41)	118.6(3)
C(15)-C(13)-P(2)	108.1(2)	C(42)-C(43)-C(44)	120.7(3)
C(14)-C(13)-P(2)	112.2(2)	C(43)-C(44)-C(39)	122.9(3)
C(16)-C(13)-P(2)	109.3(2)	C(45)#1-O(1)-C(45)	110.3(5)
C(20)-C(17)-C(18)	107.6(3)	O(1)-C(45)-C(46)	107.0(4)
C(20)-C(17)-C(19)	110.2(3)	C(46)#1-C(46)-C(45)	107.8(3)
C(18)-C(17)-C(19)	107.9(3)	C(63)-O(2)-C(60)	108.0(7)
C(20)-C(17)-P(2)	113.3(2)	O(2)-C(60)-C(61)	104.4(8)
C(18)-C(17)-P(2)	107.1(2)	C(60)-C(61)-C(62)	105.3(9)
C(19)-C(17)-P(2)	110.5(2)	C(63)-C(62)-C(61)	96.7(10)
C(22)-C(21)-C(26)	114.6(3)	O(2)-C(63)-C(62)	105.3(9)
C(22)-C(21)-B(1)	123.9(3)	C(60A)-O(2A)-C(63A)	107.5(8)
C(26)-C(21)-B(1)	121.1(3)	O(2A)-C(60A)-C(61A)	111.3(9)
C(23)-C(22)-C(21)	122.8(3)	C(62A)-C(61A)-C(60A)	104.4(8)
C(24)-C(23)-C(22)	120.8(3)	C(61A)-C(62A)-C(63A)	104.3(8)
C(23)-C(24)-C(25)	118.6(3)	O(2A)-C(63A)-C(62A)	108.5(9)

Symmetry transformations used to generate equivalent atoms:
#1 -x+1,y,-z+3/2

Table S12 Torsion angles [°] for 8-(BPh_4)$_2$.

C(2)-C(1)-N(1)-C(11)	179.5(3)	W(1)-P(1)-C(2)-C(1)	-38.4(2)
C(2)-C(1)-N(1)-W(1)	2.0(4)	N(1)-W(1)-N(2)-N(2)#1	-18.3(8)
N(1)-C(1)-C(2)-P(1)	29.9(3)	Cl(1)-W(1)-N(2)-N(2)#1	161.7(8)
C(3)-P(1)-C(2)-C(1)	-158.7(2)	P(2)-W(1)-N(2)-N(2)#1	-101.8(8)
C(7)-P(1)-C(2)-C(1)	81.9(2)	P(1)-W(1)-N(2)-N(2)#1	65.4(8)

C(2)-P(1)-C(7)-C(9)	76.4(3)	C(21)-C(22)-C(23)-C(24)	1.4(5)
C(3)-P(1)-C(7)-C(9)	-39.7(3)	C(22)-C(23)-C(24)-C(25)	-0.6(5)
W(1)-P(1)-C(7)-C(9)	-178.3(2)	C(23)-C(24)-C(25)-C(26)	-0.9(5)
C(2)-P(1)-C(7)-C(8)	-43.5(3)	C(24)-C(25)-C(26)-C(21)	1.7(5)
C(3)-P(1)-C(7)-C(8)	-159.6(2)	C(22)-C(21)-C(26)-C(25)	-0.9(5)
W(1)-P(1)-C(7)-C(8)	61.9(3)	B(1)-C(21)-C(26)-C(25)	-174.5(3)
C(2)-P(1)-C(7)-C(10)	-159.9(3)	C(39)-B(1)-C(27)-C(32)	154.8(3)
C(3)-P(1)-C(7)-C(10)	84.0(3)	C(33)-B(1)-C(27)-C(32)	-85.6(4)
W(1)-P(1)-C(7)-C(10)	-54.5(3)	C(21)-B(1)-C(27)-C(32)	32.1(4)
C(2)-P(1)-C(3)-C(4)	72.5(3)	C(39)-B(1)-C(27)-C(28)	-28.5(4)
C(7)-P(1)-C(3)-C(4)	-171.5(3)	C(33)-B(1)-C(27)-C(28)	91.1(4)
W(1)-P(1)-C(3)-C(4)	-33.0(3)	C(21)-B(1)-C(27)-C(28)	-151.2(3)
C(2)-P(1)-C(3)-C(5)	-46.9(3)	C(32)-C(27)-C(28)-C(29)	-0.3(5)
C(7)-P(1)-C(3)-C(5)	69.1(3)	B(1)-C(27)-C(28)-C(29)	-177.2(3)
W(1)-P(1)-C(3)-C(5)	-152.4(2)	C(27)-C(28)-C(29)-C(30)	0.3(6)
C(2)-P(1)-C(3)-C(6)	-169.4(2)	C(28)-C(29)-C(30)-C(31)	-0.1(6)
C(7)-P(1)-C(3)-C(6)	-53.4(3)	C(29)-C(30)-C(31)-C(32)	-0.1(5)
W(1)-P(1)-C(3)-C(6)	85.1(3)	C(30)-C(31)-C(32)-C(27)	0.1(5)
C(17)-P(2)-C(12)-C(11)	-86.9(2)	C(28)-C(27)-C(32)-C(31)	0.1(5)
C(13)-P(2)-C(12)-C(11)	153.0(2)	B(1)-C(27)-C(32)-C(31)	177.1(3)
W(1)-P(2)-C(12)-C(11)	31.7(2)	C(39)-B(1)-C(33)-C(38)	-45.6(4)
C(1)-N(1)-C(11)-C(12)	-157.5(3)	C(21)-B(1)-C(33)-C(38)	77.8(4)
W(1)-N(1)-C(11)-C(12)	20.0(4)	C(27)-B(1)-C(33)-C(38)	-166.1(3)
P(2)-C(12)-C(11)-N(1)	-35.4(3)	C(39)-B(1)-C(33)-C(34)	138.7(3)
C(12)-P(2)-C(13)-C(15)	-45.4(2)	C(21)-B(1)-C(33)-C(34)	-97.8(4)
C(17)-P(2)-C(13)-C(15)	-161.7(2)	C(27)-B(1)-C(33)-C(34)	18.2(4)
W(1)-P(2)-C(13)-C(15)	63.6(2)	C(38)-C(33)-C(34)-C(35)	-1.8(5)
C(12)-P(2)-C(13)-C(14)	73.4(2)	B(1)-C(33)-C(34)-C(35)	174.1(3)
C(17)-P(2)-C(13)-C(14)	-43.0(3)	C(33)-C(34)-C(35)-C(36)	0.9(6)
W(1)-P(2)-C(13)-C(14)	-177.65(19)	C(34)-C(35)-C(36)-C(37)	0.1(6)
C(12)-P(2)-C(13)-C(16)	-162.6(2)	C(35)-C(36)-C(37)-C(38)	-0.1(6)
C(17)-P(2)-C(13)-C(16)	81.1(2)	C(36)-C(37)-C(38)-C(33)	-0.9(6)
W(1)-P(2)-C(13)-C(16)	-53.6(2)	C(34)-C(33)-C(38)-C(37)	1.8(5)
C(12)-P(2)-C(17)-C(20)	-41.4(3)	B(1)-C(33)-C(38)-C(37)	-174.2(3)
C(13)-P(2)-C(17)-C(20)	75.7(3)	C(33)-B(1)-C(39)-C(44)	134.0(3)
W(1)-P(2)-C(17)-C(20)	-148.7(2)	C(21)-B(1)-C(39)-C(44)	12.9(4)
C(12)-P(2)-C(17)-C(18)	77.2(3)	C(27)-B(1)-C(39)-C(44)	-105.9(3)
C(13)-P(2)-C(17)-C(18)	-165.7(2)	C(33)-B(1)-C(39)-C(40)	-46.6(4)
W(1)-P(2)-C(17)-C(18)	-30.1(3)	C(21)-B(1)-C(39)-C(40)	-167.7(3)
C(12)-P(2)-C(17)-C(19)	-165.6(2)	C(27)-B(1)-C(39)-C(40)	73.4(4)
C(13)-P(2)-C(17)-C(19)	-48.5(3)	C(44)-C(39)-C(40)-C(41)	2.1(4)
W(1)-P(2)-C(17)-C(19)	87.1(2)	B(1)-C(39)-C(40)-C(41)	-177.4(3)
C(39)-B(1)-C(21)-C(22)	124.2(3)	C(39)-C(40)-C(41)-C(42)	-1.5(5)
C(33)-B(1)-C(21)-C(22)	3.3(4)	C(40)-C(41)-C(42)-C(43)	0.1(5)
C(27)-B(1)-C(21)-C(22)	-114.7(3)	C(41)-C(42)-C(43)-C(44)	0.5(5)
C(39)-B(1)-C(21)-C(26)	-62.8(4)	C(42)-C(43)-C(44)-C(39)	0.2(5)
C(33)-B(1)-C(21)-C(26)	176.2(3)	C(40)-C(39)-C(44)-C(43)	-1.4(4)
C(27)-B(1)-C(21)-C(26)	58.2(4)	B(1)-C(39)-C(44)-C(43)	178.0(3)
C(26)-C(21)-C(22)-C(23)	-0.7(5)	C(45)#1-O(1)-C(45)-C(46)	-1.1(4)
B(1)-C(21)-C(22)-C(23)	172.7(3)	O(1)-C(45)-C(46)-C(46)#1	2.9(11)

C(63)-O(2)-C(60)-C(61)	9.0(13)	C(63A)-O(2A)-C(60A)-C(61A)	-11.3(19)
O(2)-C(60)-C(61)-C(62)	19.5(15)	O(2A)-C(60A)-C(61A)-C(62A)	-1.4(19)
C(60)-C(61)-C(62)-C(63)	-37.9(15)	C(60A)-C(61A)-C(62A)-C(63A)	12.4(15)
C(60)-O(2)-C(63)-C(62)	-35.0(12)	C(60A)-O(2A)-C(63A)-C(62A)	19.4(16)
C(61)-C(62)-C(63)-O(2)	44.1(13)	C(61A)-C(62A)-C(63A)-O(2A)	-19.9(14)

Symmetry transformations used to generate equivalent atoms:

#1 -x+1,y,-z+3/2

Crystal Structure of **9-OTf**

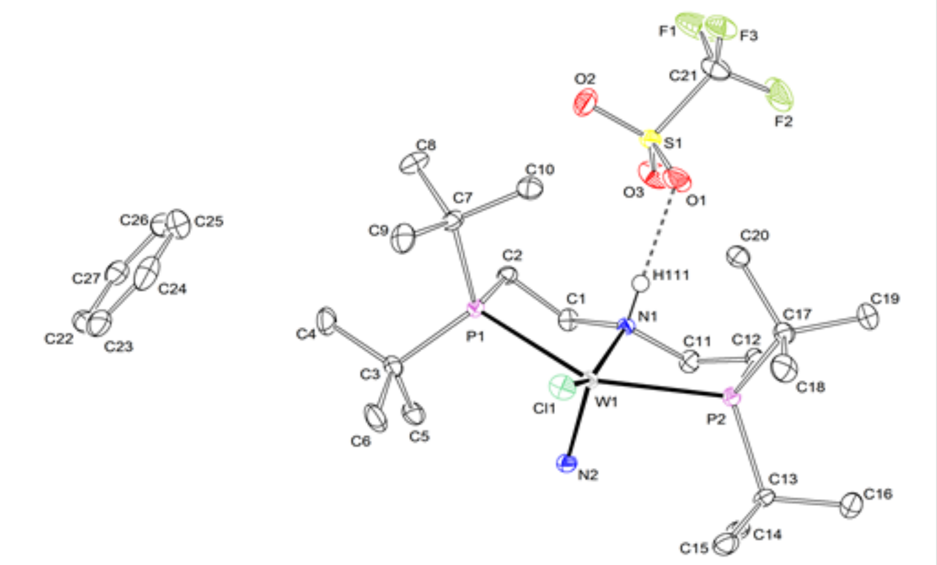

Figure S35 Thermal ellipsoid plot of **9-OTf** with the anisotropic displacement parameters drawn at the 50% probability level. The asymmetric unit contains one cationic complex molecule, one CF_3COO^- anion and one benzene solvent molecule. The N-H hydrogen atom was found from the residual density map and isotropically refined.

Table S13 Crystal data and structure refinement for 9-OTf.

Identification code	CW_BS_090517_a (BS-B-135)	
Empirical formula	$C_{27}H_{51}ClF_3N_2O_3P_2SW$	
Formula weight	821.99	
Temperature	100(2) K	
Wavelength	0.71073 Å	
Crystal system	Monoclinic	
Space group	$P2_1/c$	
Unit cell dimensions	a = 19.1996(8) Å	α = 90°
	b = 10.9562(4) Å	β= 99.585(2)°
	c = 16.6152(7) Å	γ = 90°
Volume	3446.3(2) $Å^3$	
Z	4	
Density (calculated)	1.584 Mg/m^3	
Absorption coefficient	3.629 mm^{-1}	
F(000)	1660	
Crystal size	0.336 x 0.327 x 0.178 mm^3	
Crystal shape and color	Plate, clear intense green	
Theta range for data collection	2.236 to 28.437°	
Index ranges	-25<=h<=25, -14<=k<=14, -22<=l<=22	
Reflections collected	130600	
Independent reflections	8680 [R(int) = 0.0796]	
Completeness to theta = 25.242°	100.0 %	
Refinement method	Full-matrix least-squares on F^2	
Data / restraints / parameters	8680 / 0 / 377	
Goodness-of-fit on F2	1.058	
Final R indices [I>2sigma(I)]	R1 = 0.0261,	wR2 = 0.0470
R indices (all data)	R1 = 0.0371,	wR2 = 0.0495
Largest diff. peak and hole	1.456 and -1.350 $eÅ^{-3}$	

Table S14 Bond lengths [Å] and angles [°] for 9-OTf.

W(1)-N(2)	1.679(2)	N(1)-W(1)-P(2)	78.45(6)
W(1)-N(1)	2.202(2)	Cl(1)-W(1)-P(2)	97.58(2)
W(1)-Cl(1)	2.3657(7)	P(1)-W(1)-P(2)	152.54(2)
W(1)-P(1)	2.5308(7)	O(3)-S(1)-O(2)	115.73(14)
W(1)-P(2)	2.5352(7)	O(3)-S(1)-O(1)	114.27(14)
S(1)-O(3)	1.429(2)	O(2)-S(1)-O(1)	114.16(14)
S(1)-O(2)	1.431(2)	O(3)-S(1)-C(21)	104.15(13)
S(1)-O(1)	1.445(2)	O(2)-S(1)-C(21)	104.15(14)
S(1)-C(21)	1.823(3)	O(1)-S(1)-C(21)	102.19(13)
P(1)-C(2)	1.840(3)	C(2)-P(1)-C(7)	104.70(12)
P(1)-C(7)	1.871(3)	C(2)-P(1)-C(3)	106.43(12)
P(1)-C(3)	1.871(3)	C(7)-P(1)-C(3)	113.18(12)
P(2)-C(12)	1.837(3)	C(2)-P(1)-W(1)	100.43(8)
P(2)-C(17)	1.870(3)	C(7)-P(1)-W(1)	115.51(9)
P(2)-C(13)	1.872(3)	C(3)-P(1)-W(1)	114.71(9)
F(1)-C(21)	1.327(4)	C(12)-P(2)-C(17)	104.42(12)
F(2)-C(21)	1.320(4)	C(12)-P(2)-C(13)	106.68(12)
F(3)-C(21)	1.335(3)	C(17)-P(2)-C(13)	114.22(12)
N(1)-C(1)	1.493(3)	C(12)-P(2)-W(1)	100.79(8)
N(1)-C(11)	1.498(3)	C(17)-P(2)-W(1)	114.39(9)
N(1)-H(111)	0.85(3)	C(13)-P(2)-W(1)	114.49(8)
C(1)-C(2)	1.519(4)	C(1)-N(1)-C(11)	110.2(2)
C(3)-C(4)	1.527(4)	C(1)-N(1)-W(1)	113.30(15)
C(3)-C(5)	1.533(4)	C(11)-N(1)-W(1)	113.20(16)
C(3)-C(6)	1.538(4)	C(1)-N(1)-H(111)	106(2)
C(7)-C(8)	1.532(4)	C(11)-N(1)-H(111)	102.9(19)
C(7)-C(9)	1.534(4)	W(1)-N(1)-H(111)	111(2)
C(7)-C(10)	1.542(4)	N(1)-C(1)-C(2)	109.7(2)
C(11)-C(12)	1.524(4)	C(1)-C(2)-P(1)	110.90(18)
C(13)-C(16)	1.532(4)	C(4)-C(3)-C(5)	108.2(2)
C(13)-C(15)	1.536(4)	C(4)-C(3)-C(6)	110.6(2)
C(13)-C(14)	1.538(4)	C(5)-C(3)-C(6)	108.7(2)
C(17)-C(18)	1.533(4)	C(4)-C(3)-P(1)	112.89(19)
C(17)-C(20)	1.537(4)	C(5)-C(3)-P(1)	107.74(18)
C(17)-C(19)	1.537(4)	C(6)-C(3)-P(1)	108.57(18)
C(22)-C(23)	1.380(5)	C(8)-C(7)-C(9)	110.4(2)
C(22)-C(27)	1.384(4)	C(8)-C(7)-C(10)	108.9(2)
C(23)-C(24)	1.378(4)	C(9)-C(7)-C(10)	108.5(2)
C(24)-C(25)	1.380(5)	C(8)-C(7)-P(1)	112.99(19)
C(25)-C(26)	1.377(5)	C(9)-C(7)-P(1)	110.32(18)
C(26)-C(27)	1.376(4)	C(10)-C(7)-P(1)	105.44(18)
		N(1)-C(11)-C(12)	109.7(2)
N(2)-W(1)-N(1)	99.26(10)	C(11)-C(12)-P(2)	110.18(17)
N(2)-W(1)-Cl(1)	105.91(8)	C(16)-C(13)-C(15)	110.6(2)
N(1)-W(1)-Cl(1)	154.83(6)	C(16)-C(13)-C(14)	108.9(2)
N(2)-W(1)-P(1)	98.88(7)	C(15)-C(13)-C(14)	108.0(2)
N(1)-W(1)-P(1)	78.60(6)	C(16)-C(13)-P(2)	112.10(18)
Cl(1)-W(1)-P(1)	96.77(2)	C(15)-C(13)-P(2)	109.15(18)
N(2)-W(1)-P(2)	99.51(7)	C(14)-C(13)-P(2)	107.95(18)

C(18)-C(17)-C(20)	108.4(2)	F(2)-C(21)-S(1)	111.3(2)
C(18)-C(17)-C(19)	109.7(2)	F(1)-C(21)-S(1)	111.2(2)
C(20)-C(17)-C(19)	108.9(2)	F(3)-C(21)-S(1)	111.2(2)
C(18)-C(17)-P(2)	110.24(18)	C(23)-C(22)-C(27)	120.2(3)
C(20)-C(17)-P(2)	105.56(17)	C(24)-C(23)-C(22)	119.7(3)
C(19)-C(17)-P(2)	113.8(2)	C(23)-C(24)-C(25)	119.9(3)
F(2)-C(21)-F(1)	107.8(3)	C(26)-C(25)-C(24)	120.6(3)
F(2)-C(21)-F(3)	108.0(3)	C(27)-C(26)-C(25)	119.6(3)
F(1)-C(21)-F(3)	107.2(2)	C(26)-C(27)-C(22)	120.1(3)

Table S15 Torsion angles [°] for 9-OTf.

C(11)-N(1)-C(1)-C(2)	-172.5(2)	W(1)-P(2)-C(13)-C(16)	-175.93(17)
W(1)-N(1)-C(1)-C(2)	59.6(2)	C(12)-P(2)-C(13)-C(15)	-163.62(18)
N(1)-C(1)-C(2)-P(1)	-50.8(2)	C(17)-P(2)-C(13)-C(15)	81.6(2)
C(7)-P(1)-C(2)-C(1)	140.95(18)	W(1)-P(2)-C(13)-C(15)	-53.1(2)
C(3)-P(1)-C(2)-C(1)	-98.94(19)	C(12)-P(2)-C(13)-C(14)	-46.4(2)
W(1)-P(1)-C(2)-C(1)	20.88(18)	C(17)-P(2)-C(13)-C(14)	-161.23(17)
C(2)-P(1)-C(3)-C(4)	-74.6(2)	W(1)-P(2)-C(13)-C(14)	64.15(19)
C(7)-P(1)-C(3)-C(4)	39.8(2)	C(12)-P(2)-C(17)-C(18)	-168.78(19)
W(1)-P(1)-C(3)-C(4)	175.30(17)	C(13)-P(2)-C(17)-C(18)	-52.6(2)
C(2)-P(1)-C(3)-C(5)	44.8(2)	W(1)-P(2)-C(17)-C(18)	82.03(19)
C(7)-P(1)-C(3)-C(5)	159.26(18)	C(12)-P(2)-C(17)-C(20)	74.4(2)
W(1)-P(1)-C(3)-C(5)	-65.3(2)	C(13)-P(2)-C(17)-C(20)	-169.48(17)
C(2)-P(1)-C(3)-C(6)	162.4(2)	W(1)-P(2)-C(17)-C(20)	-34.8(2)
C(7)-P(1)-C(3)-C(6)	-83.2(2)	C(12)-P(2)-C(17)-C(19)	-45.0(2)
W(1)-P(1)-C(3)-C(6)	52.3(2)	C(13)-P(2)-C(17)-C(19)	71.1(2)
C(2)-P(1)-C(7)-C(8)	41.2(2)	W(1)-P(2)-C(17)-C(19)	-154.24(17)
C(3)-P(1)-C(7)-C(8)	-74.3(2)	O(3)-S(1)-C(21)-F(2)	-58.1(3)
W(1)-P(1)-C(7)-C(8)	150.64(17)	O(2)-S(1)-C(21)-F(2)	-179.8(2)
C(2)-P(1)-C(7)-C(9)	165.37(19)	O(1)-S(1)-C(21)-F(2)	61.2(2)
C(3)-P(1)-C(7)-C(9)	49.9(2)	O(3)-S(1)-C(21)-F(1)	62.1(3)
W(1)-P(1)-C(7)-C(9)	-85.20(19)	O(2)-S(1)-C(21)-F(1)	-59.6(2)
C(2)-P(1)-C(7)-C(10)	-77.64(19)	O(1)-S(1)-C(21)-F(1)	-178.7(2)
C(3)-P(1)-C(7)-C(10)	166.87(17)	O(3)-S(1)-C(21)-F(3)	-178.5(2)
W(1)-P(1)-C(7)-C(10)	31.8(2)	O(2)-S(1)-C(21)-F(3)	59.8(2)
C(1)-N(1)-C(11)-C(12)	171.9(2)	O(1)-S(1)-C(21)-F(3)	-59.3(2)
W(1)-N(1)-C(11)-C(12)	-60.1(2)	C(27)-C(22)-C(23)-C(24)	-0.1(5)
N(1)-C(11)-C(12)-P(2)	51.8(2)	C(22)-C(23)-C(24)-C(25)	-0.2(5)
C(17)-P(2)-C(12)-C(11)	-140.82(19)	C(23)-C(24)-C(25)-C(26)	0.4(5)
C(13)-P(2)-C(12)-C(11)	97.9(2)	C(24)-C(25)-C(26)-C(27)	-0.1(5)
W(1)-P(2)-C(12)-C(11)	-21.93(19)	C(25)-C(26)-C(27)-C(22)	-0.2(5)
C(12)-P(2)-C(13)-C(16)	73.5(2)	C(23)-C(22)-C(27)-C(26)	0.3(5)
C(17)-P(2)-C(13)-C(16)	-41.3(2)		

Table S16 Hydrogen bonds for 9-OTf [Å and °].

D-H...A	d(D-H)	d(H...A)	d(D...A)	<(DHA)
N(1)-H(111)...O(1)	0.85(3)	2.03(3)	2.870(3)	171(3)

4. DFT-Calculations

4.1 Computational Details

All calculations were performed within the ORCA program suite.[12] Optimization of the molecular structures was carried out using the PBE0[13] functional, Grimme's dispersion correction with Becke-Johnson damping (D3(BJ))[14] and the RIJCOSX[15] approach to minimize computational costs. The hybrid DFT functional was used as the SCF steps with the pure GGA functional PBE fail to converge in case of quasi-degenerate orbitals such as for the oxidized dimer **7**$^+$. In contrast, PBE0 reproduces the valence localized structure of **7**$^+$ in which the degeneracy is lifted by distortion along the W-N=N-W unit.[16] Ahlrichs' revised def2-SVP basis set and the corresponding auxiliary basis set were used with an all electron basis for all elements but W for which a Stuttgart-Dresden 60 electron core potential replaced the inner shell 1s-4f orbitals.[17] Tight convergence criteria in the SCF procedure and optimization and a fine integration grid (Grid 5 and GRIDX5) were applied in all calculations. The full structures were evaluated except for NEt_3 and $HNEt_3^+$ that were truncated to NMe_3 and $HNMe_3^+$, respectively, to limit the conformational space. No symmetry restrains were imposed and the optimized (gas phase) structures were defined as minima (no negative eigenvalue) or transition states (one negative eigenvalue) by analytical vibrational analyses at the same level of theory as the geometry optimization. Transition states were verified by distortion of the structures along the reaction mode followed by full optimizations.

The electronic structures of dinuclear W complexes with high spin electronic configuration were re-evaluated by the broken symmetry protocol and their geometries re-optimized within this approach. The energies of the open-shell singlet (OSS) BS(1,1) or BS(2,2) states, which denotes the number of coupling electrons, were estimated from the energies ε_{LS} of the optimized single-determinant broken symmetry solutions and the energies ε_{HS} from separate unrestricted triplet (m_s = 1) or quintet (m_s = 2) high spin calculations at the same geometry with the same functional and basis set, using the approximate spin correction formula proposed by Yamaguchi:[18]

$$\varepsilon_S \approx \frac{S_{\mathrm{HS}}^2\varepsilon_{\mathrm{LS}} - S_{\mathrm{LS}}^2\varepsilon_{\mathrm{HS}}}{S_{\mathrm{HS}}^2 - S_{\mathrm{LS}}^2}$$

The antiferromagnetic coupling constant can be estimated accordingly by:

$$J_A \approx 2.1947 \cdot 10^5 cm^{-1} \cdot \frac{\varepsilon_{\mathrm{LS}} - \varepsilon_{\mathrm{HS}}}{S_{\mathrm{HS}}^2 - S_{\mathrm{LS}}^2}$$

In fact, the ground state of **8**$^{2+}$ is correctly predicted applying Yamaguchi's correction while without, the OSS and triplet states are computed nearly equal in free energy (0.1 kcal·mol^{-1} in favour of the triplet state).

Similarly, the transition states of nitrogen splitting were computed on the OSS BS(2,2) energy surface. However, applying Yamaguchi's correction leads to rather unrealistic coupling constants of over 1000 cm^{-1}. Therefore, we refrained from its application and used the total energies directly without spin projection.

Thermodynamic data were computed by applying Grimme's quasi-RRHO approach which treats low energy frequencies below 35 cm^{-1} as free rotors instead of harmonic vibrations for the vibrational partition function.[19] The thus obtained free energies were further corrected for the difference between ideal gas standard conditions (1 atm, 298.15 K) and standard solution conditions (1 mol/L, 298.15 K):

$$G_{sol} = G_{gas} + RTln\frac{RT}{p}$$

$$G_{sol} = G_{gas} + RTln(24.47)$$

$$G_{sol} = G_{gas} + 1.89 \text{ kcal/mol}$$

In steps involving reaction of the THF molecule (see next section), the free energy was further corrected by applying the actual concentration of the pure solvent, i.e (ρ = 0.889 g/ml (25°C), c = 12.3 mol/l)[1]:

$$G'_{sol} = G_{sol} + RTlnc$$

[1] The influence of temperature on the actual density and thus concentration of pure THF was neglected.

$$G'_{sol} = G_{sol} + 1.49 \text{ kcal/mol}$$

Single point calculations were conducted with the M06 functional[20] and Ahlrichs' def2-TZVPP basis set for all atoms, again replacing the 60 core electrons of W with the SD(60,MWB) effective core potential. The influence of the solvent (THF) was accounted for with Truhlar's SMD solvation model.[21]

The redox potentials of the **6/7$^+$** and **7$^+$/8^{2+}** redox couples in THF have been computed from the free energy differences of the redox couples according to:

$$E_{abs} = \frac{G(Ox) - G(Red)}{F}$$

giving the absolute potential which is not known from experiment. To circumvent any possible problems that could arise by comparison with a reference such as ferrocene,[22] the potentials were calibrated to the related $[Re^{VI}(N)Cl(PNP)]^+/[Re^{V}(N)Cl(PNP)]$ redox couple that exhibits a fully reversible wave at -0.086 V:[23]

$$E(Red/Ox) = E_{abs}^{DFT}(Red/Ox) - E_{abs}^{DFT}(Re^{V}/Re^{VI}) + (-0.086\ V)$$

4.2 Thermodynamics of protonation

In order to describe the strength of the applied acids HOTf and $HNMe_3^+$ (for $HNEt_3^+$) realistically, the acid/base equilibria of both acids with the solvent (THF) were investigated (see Table S17). According to these calculations, H^+ is stabilized in solution by binding to two THF molecules. Optimization of structures with more than two THF molecules bound to the proton always lead to linear structures where the additional molecules separated from the $H(THF)_2^+$ core. Complete dissociation of HOTf and $HNMe_3^+$ are both endothermic and endergonic, excluding $H(THF)_2^+$ as actual acid. Furthermore, stabilization of the free acid by binding to a solvent molecule is both exothermic and exergonic for HOTf while for $HNMe_3^+$, it is endergonic reflecting the lower acidity of the latter. Thus, $HNMe_3^+$ and the THF-adduct of HOTf (THF–H–OTf) were applied as actual acids in the protonation reactions, respectively.

In consequence, the driving force of the protonation steps with HOTf (ΔG) is lowered by 3.3 kcal·mol^{-1} in comparison to pure HOTf. Additionally, the influence of entropy changes with the number of molecules involved.

Table S17. Calculated energies (ΔE) and free energies (ΔG) of several protonation equilibria in kcal·mol^{-1}

**A =**	thf		OTf$^-$		NMe$_3$	
**Reaction**	**ΔE**	**ΔG**	**ΔE**	**ΔG**	**ΔE**	**ΔG**
HA^+ + thf ⇄ (thf)H^+ + A	-	-	16.8	15.8	29.8	28.0
HA^+ + thf ⇄ (thf)–H–A^+	-17.2	-9.6	-12.3	-3.3	-5.0	2.0
HA^+ + 2 thf ⇄ $H(thf)_2^+$ + A	-	-	-0.4	6.2	12.5	18.4
(thf)–HA^+ + thf ⇄ $H(thf)_2^+$ + A	-	-	11.9	9.5	17.5	16.4

Taking into account that the acid constant K_a neglects the solvent concentration and assuming the following equilibria:

(thf)–H–OTf + thf ⇄ $H(thf)_2^+$ + OTf^- (ΔG = 9.5 kcal·mol^{-1})
$HNMe_3^+$ + 2 thf ⇄ $H(thf)_2^+$ + NMe_3 (ΔG = 18.4 kcal·mol^{-1})

the pK_a difference in THF between $HNMe_3^+$ (N) and HOTf (O) can be estimated according to:

$$pK_a^N - pK_a^O = -\log(K^N \cdot [\text{thf}]^2) + \log(K^O \cdot [\text{thf}])$$
$$= \log \frac{K^O \cdot [\text{thf}]}{K^N \cdot [\text{thf}]^2}$$

$$= \frac{1}{2.303}(\ln K^O - \ln K^N - \ln[\text{thf}])$$
$$= \frac{1}{2.303}\left(\frac{\Delta G^N - \Delta G^O}{RT} - \ln[\text{thf}]\right)$$
$$= \frac{1}{2.303}\left(\frac{4184 \cdot (18.4 - 9.5)}{RT} - \ln 12.3\right)$$
$$= 5.4$$

The computed value is in good agreement with the experimental pK_a difference of $HNEt_3^+$ and HOTf ($\Delta pK_a = 4.7$) corresponding to an error of 1 kcal·mol^{-1} in ΔΔG. Notably, the difference between the computed and experimental pK_a values should be slightly larger due to the use of the truncated acid (NEt_3 is slightly more basic than NMe_3), indicating some error cancellation effect. However, assuming the following equilibrium for HOTf:

$$\text{HOTf} + 2\ \text{thf} \rightleftarrows \text{H(thf)}_2^+ + \text{OTf}^- \qquad (\Delta G = 6.2\ \text{kcal·mol}^{-1})$$

the computed difference of the pK_a values would be 8.9, thus severely overestimating the acidity of HOTf in comparison to $HNMe_3^+$. For simplification, we write in all schemes HOTf, although the acid that was actually considered is the THF adduct liberating an additional molecule of THF in the proton transfer step.

4.3 Computed reaction energies and electronic ground states

4.3.1 Computed reaction profile with HOTf

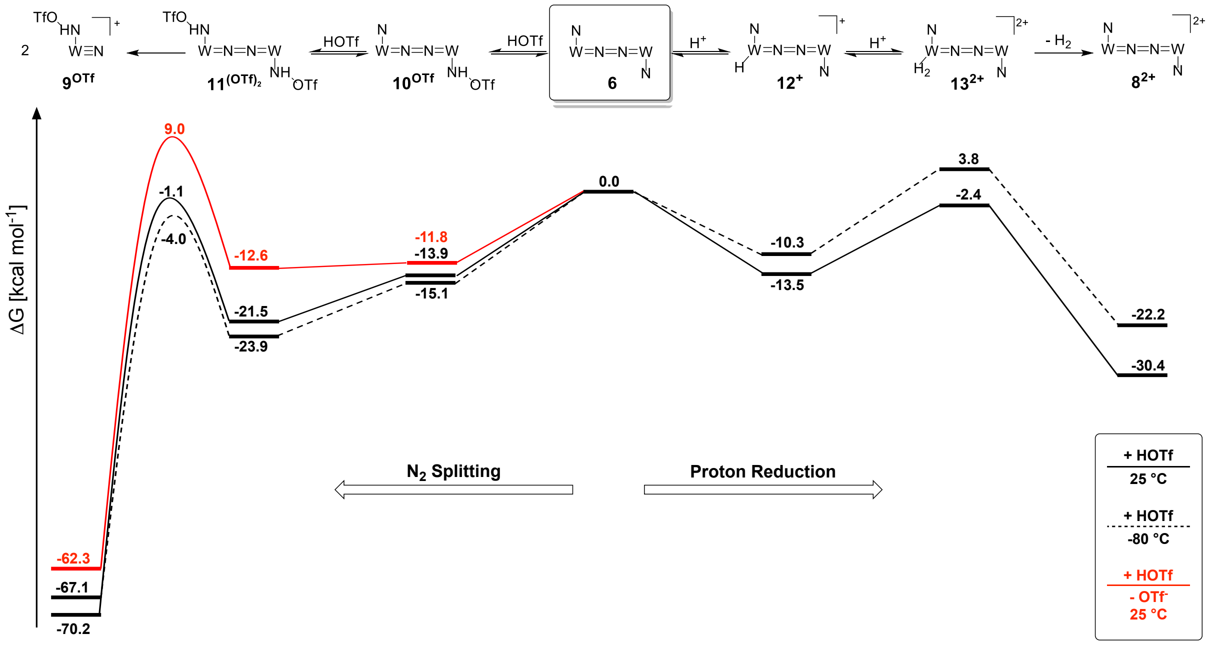

Scheme S1 Energy profile (ΔG in kcal·mol^{-1}) of the possible reaction pathways for the double protonation of **6** either leading to N_2 splitting (left pathway) or to proton reduction (right pathway). The energies for protonation with triflic acid at 25 °C (solid) and -80 °C (dashed) are depicted in black (counter ion assisted) or red (without counter ion). The evaluated pathway of proton reduction is in all cases the same.

4.3.2 Structural, electronic and spectroscopic properties of **6**, **7⁺** and **8²⁺**

The structural and electronic properties of the redox series of the W dimers are well reproduced by density functional theory including Hartree Fock exchange. Structurally, the N—N distances of the bridging dinitrogen ligand are slightly shorter (around 0.03 Å) and the W—N bind distances slightly longer than determined in the crystal hinting towards an underestimation of the strength of the W-N bonding interactions by DFT. Consequently, the computed N-N stretching vibrations are blueshifted by around 180 – 200 cm^{-1}. However, the trend within the redox series is reproduced. For **8²⁺**, a diamagnetic ground state is correctly predicted that is arising from antiferromagnetic coupling of two W based S = ½ centers. The lowest triplet structure is only 0.5 kcal·mol^{-1} higher in energy. More elaborate theoretical methods, e.g. multireference approaches, are required to describe the electronic properties of this compound more exactly. However, the computed antiferromagnetic coupling within the Broken Symmetry approach is close to the experimental value (see table below). More importantly, the redox potentials of the **6**/**7⁺** and **7⁺**/**8²⁺** redox couples are very close to experiment showing that the energy differences within this series are very accurate. Oxidation of **6** and **7⁺** is purely metal centered and occurs by partial depletion of the nonbonding δ-orbitals (see MO schemes below).

Table S18 Computed and experimental structural, electronic and spectroscopic properties of **6**, **7⁺** and **8²⁺.**

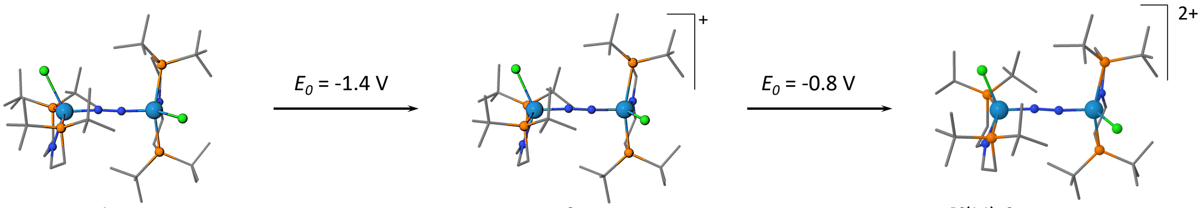

^{1}**6** ^{2}**7⁺** $^{BS(1,1)}$**8²⁺**

	**6**		**7⁺**		**8²⁺**	
	**Exp.**	**DFT**	**Exp.**	**DFT**	**Exp.**	**DFT**
Ground state	Diamagnetic	S = 0	S = ½	S = ½<br>$\langle S^2\rangle$ = 0.778	Diamagnetic ground state<br>*J* =-59 cm^{-1}	BS(1,1)<br>*J* = -184 cm^{-1}<br>$\langle S^2\rangle$ = 1.065
E^0 (V) in THF	-1.39 V (**6/7⁺**)	-1.40 (**6/7⁺**)	-0.91 (**7⁺/8²⁺**)	-0.80 (**7⁺/8²⁺**)	-	-
$\upsilon_{N\text{-}N}$ (cm^{-1})	1392	1567	1414	1610	1400	1579
W—N (Å)	1.78 (2)/<br>1.82 (4)	1.798	1.813<br>1.781	1.820<br>1.783	1.785	1.795
N—N (Å)	1.33 (4) /<br>1.27 (8)	1.249	1.266	1.239	1.277	1.240

Table S19 Computed SCF and free energy corrections of **8²⁺** (in kcal·mol^{-1}).

			**ΔE | ΔG with correction**	**ΔE | ΔG without correction**
$^{BS(1,1)}$**8²⁺**	ε_{LS} = -4215.588516 H<br>ε_{HS} = -4215.587703 H<br>E_{ZPE} = 1.245362 H	$\langle S^2\rangle_{LS}$ = 1.065<br>$\langle S^2\rangle_{HS}$ = 2.034<br>ΔG = 727.25 kcal·mol^{-1}	0.0 | 0.0	0.0 | 0.1
^{3}**8²⁺**	E_{SCF} = -4215.587627 H<br>E_{ZPE} = 1.245377 H	ΔG = 726.62 kcal·mol^{-1}	1.1 | 0.5	0.6 | 0.0

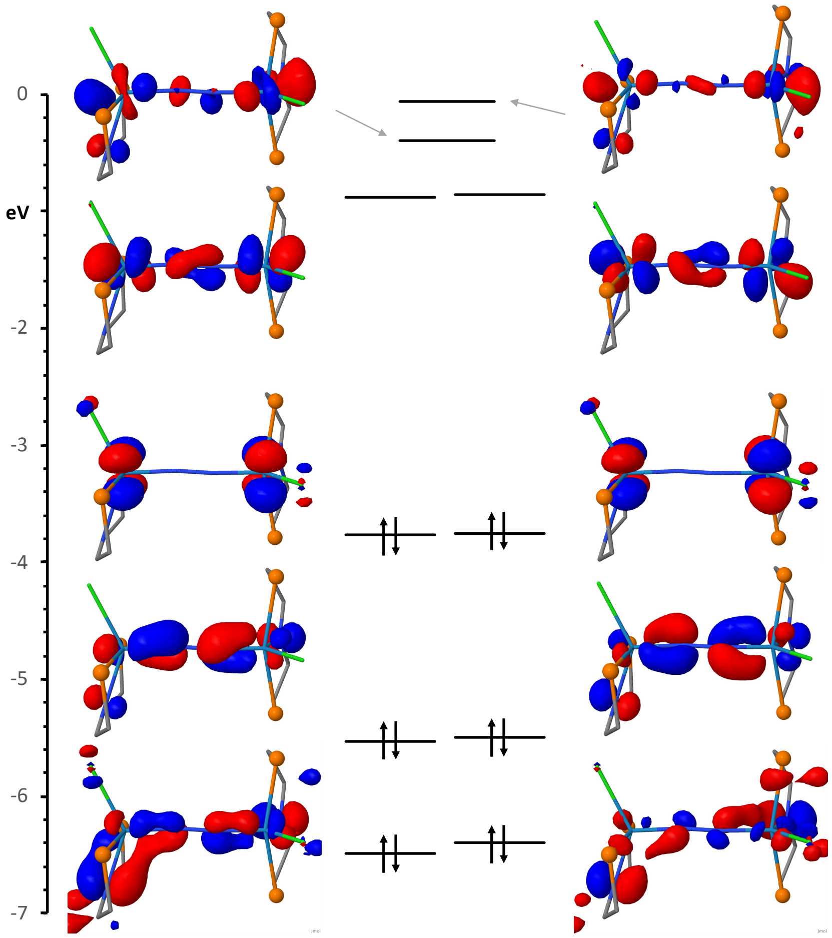

Figure S36 MO Scheme of **6**: Nearly degenerate orbitals are arranged side by side in two columns. The HOMOs are the non-bonding metal centred δ-orbitals.

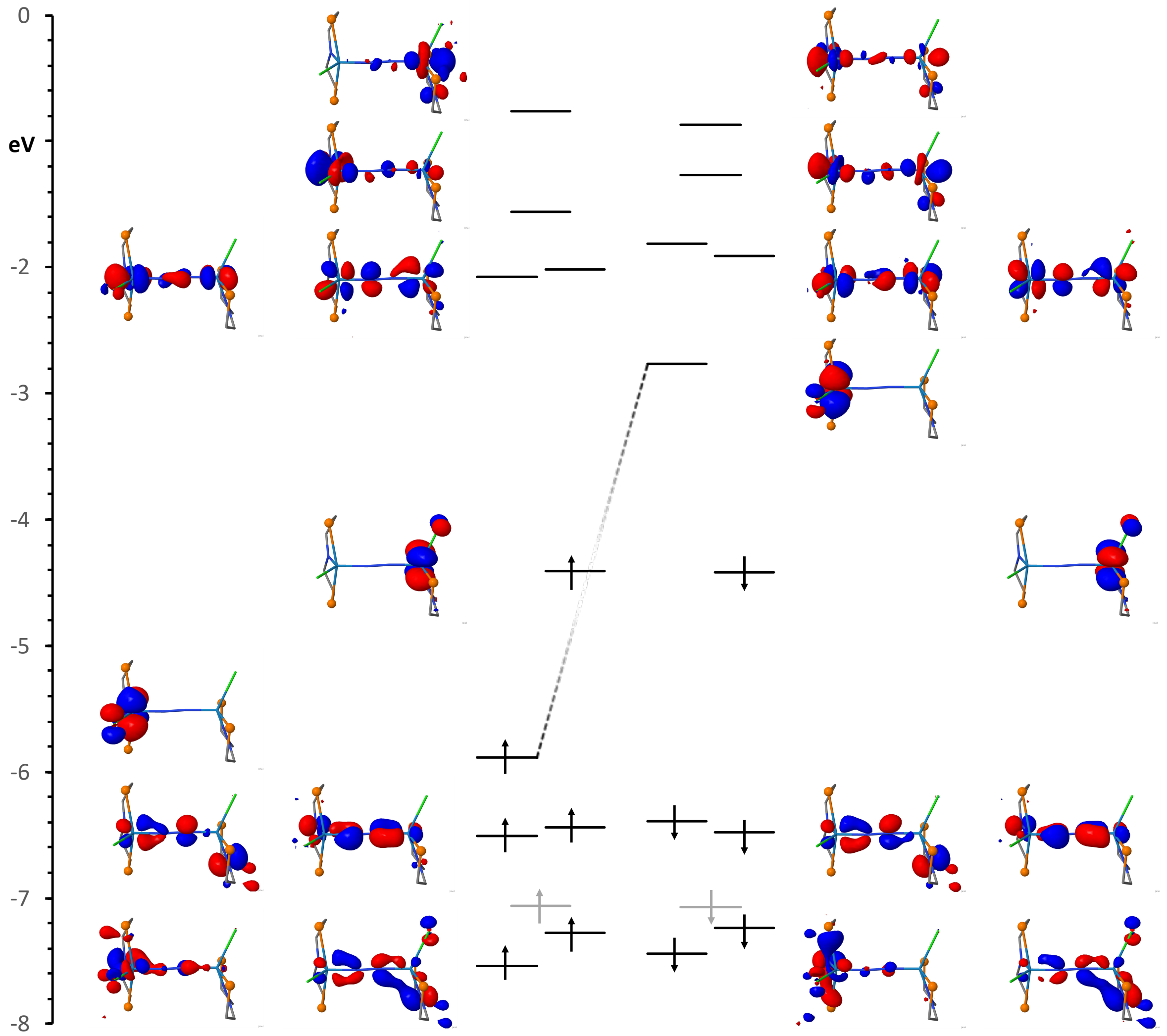

Figure S37 MO Scheme of **7**$^+$: Orbitals in grey are not shown for simplification, α and β spaces are visualized separately. Nearly degenerate orbitals are arranged side by side in two columns. The SOMO is one of the non-bonding metal centred δ-orbitals and exclusively localized at one W centre. The nature of the SOMO is reflected in the spin density (see below) which exhibits only weak spin polarization of the neighbouring atoms.

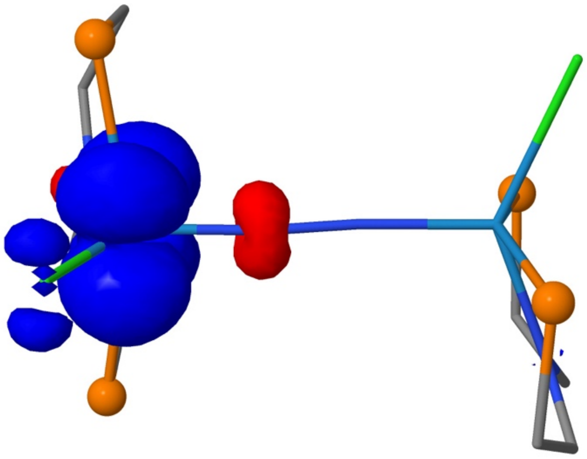

Figure S38 Spin density plot of **7**$^+$.

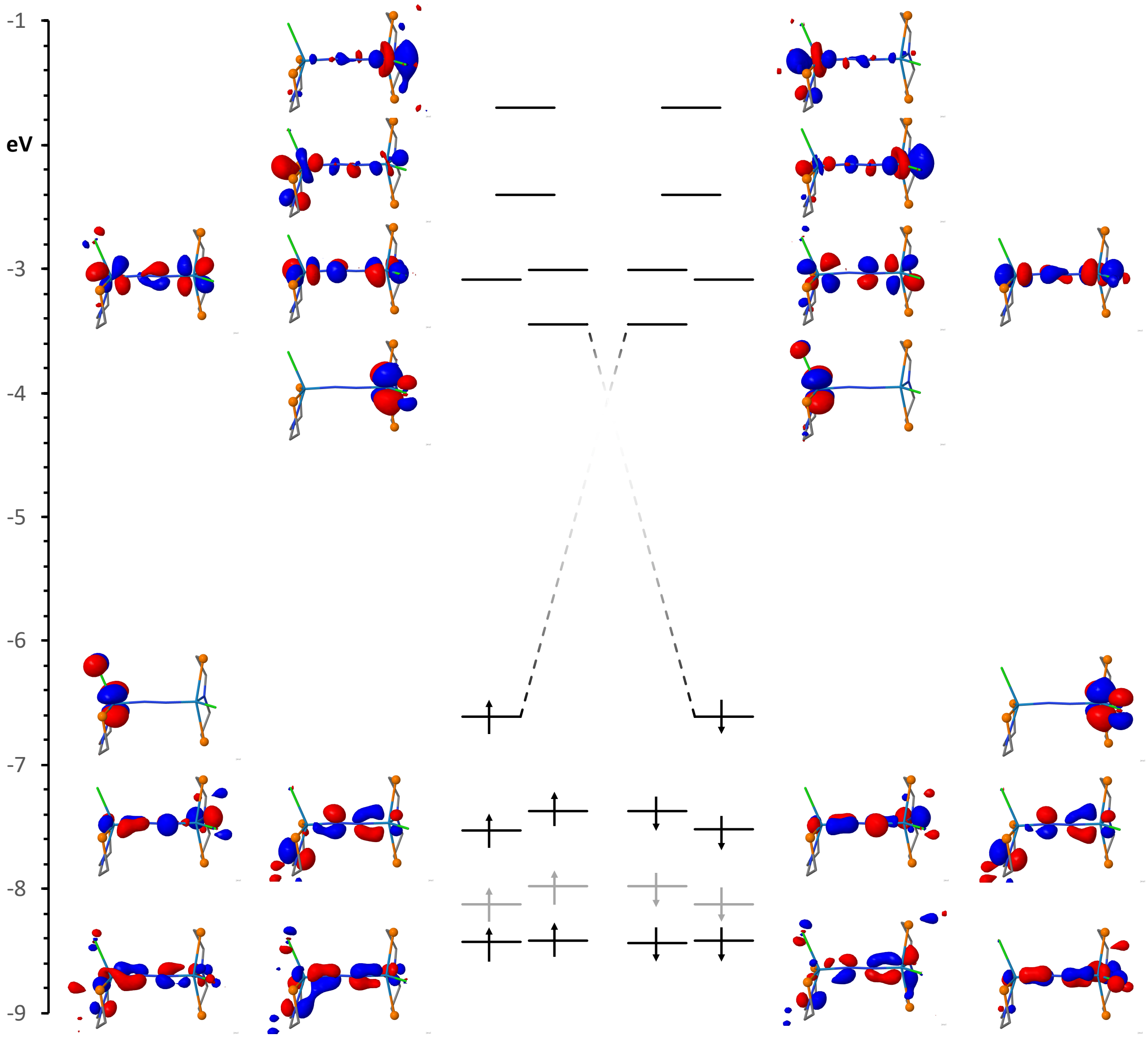

Figure S39 MO Scheme of **8**$^{2+}$: Orbitals in grey are not shown, α and β spaces are visualized separately. Nearly degenerate orbitals are arranged side by side in two columns. The SOMO's are the two non-bonding metal centred δ-orbitals.

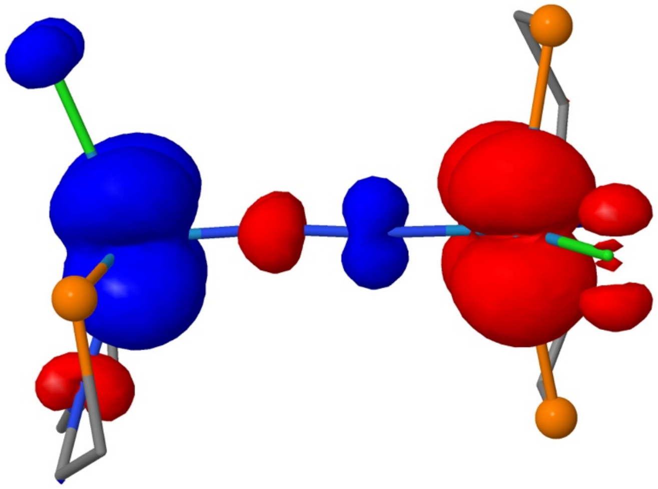

Figure S40 Spin density plot of **8**$^{2+}$.

4.3.3 First protonation step

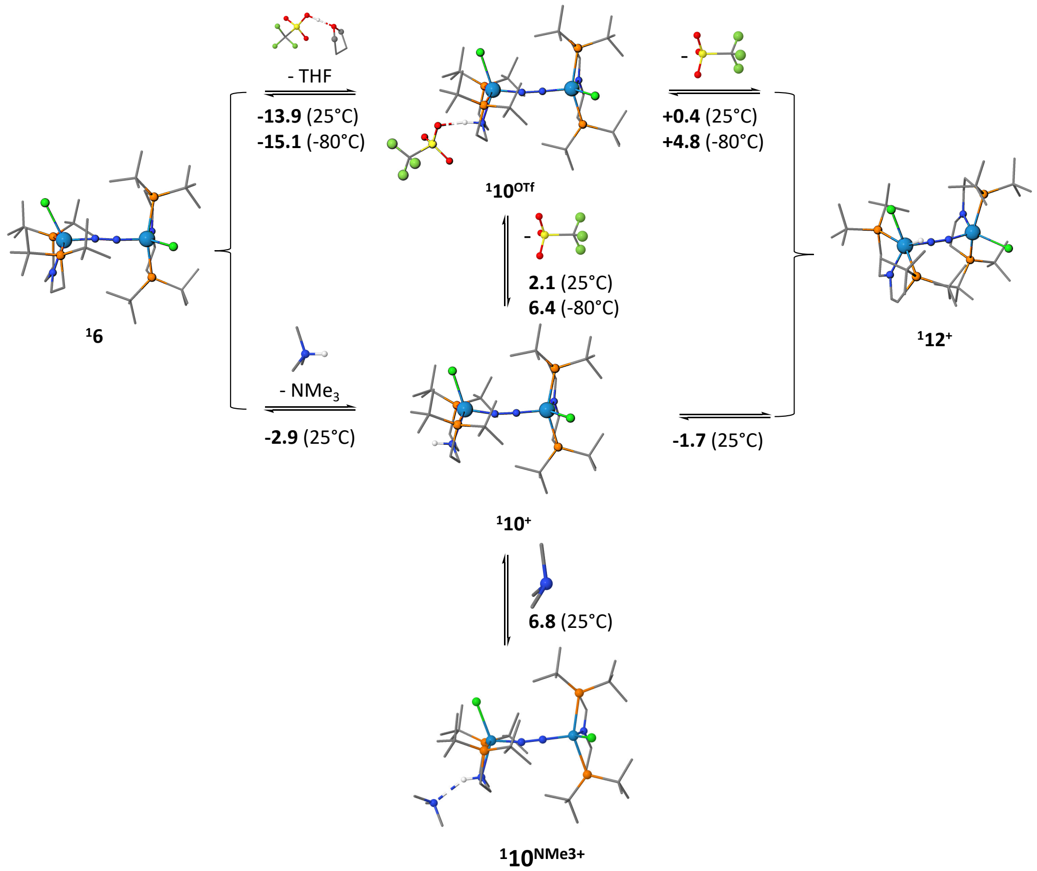

Figure S41 Reaction free energies (in kcal·mol^{-1}) of the first protonation steps with and without hydrogen bonds to the corresponding base (NMe_3 or OTf^-). For all calculated structures, a closed shell ground state is predicted with other spin states at least 10 kcal·mol^{-1} higher in energy. All hydrogen atoms are omitted except the transferred one.

4.3.4 Second protonation step, nitrogen splitting and hydrogen formation

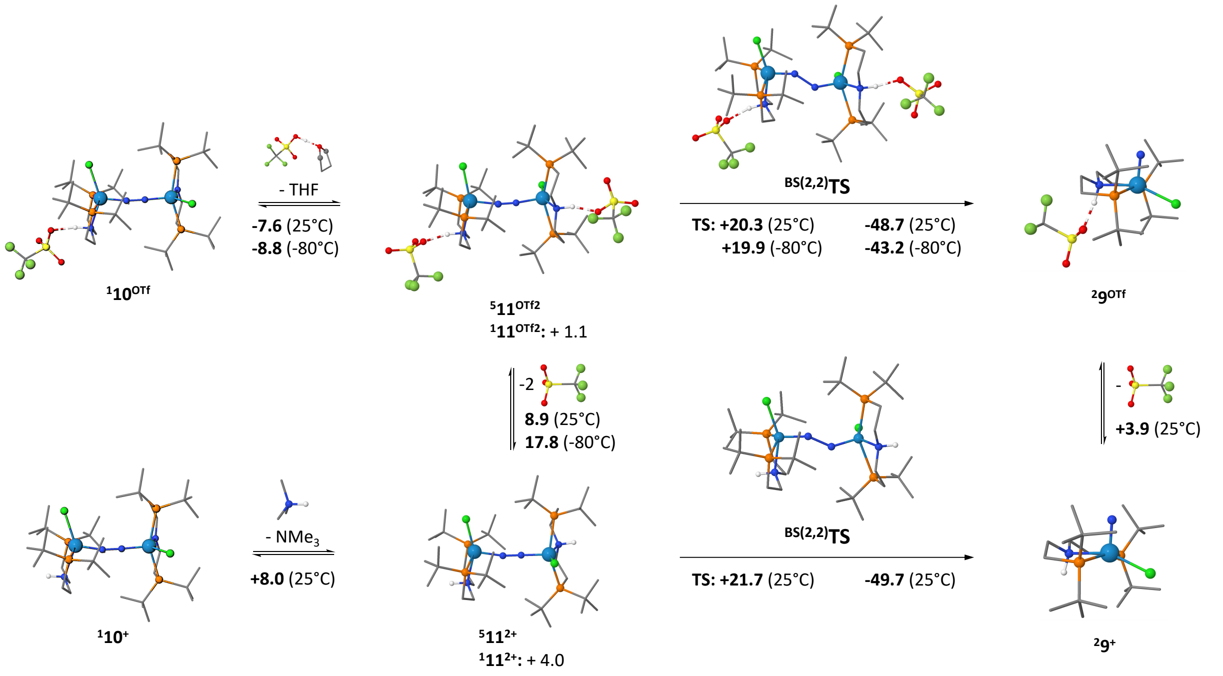

Figure S42 Reaction free energies (in kcal·mol^{-1}) of the second protonation step leading to nitrogen splitting with and without hydrogen bonds to OTf^-. Second protonation of the pincer backbone is accompanied by a spin change to a quintet ground state with only a small ΔG difference to the closed shell singlet. All hydrogen atoms are omitted except the transferred one.

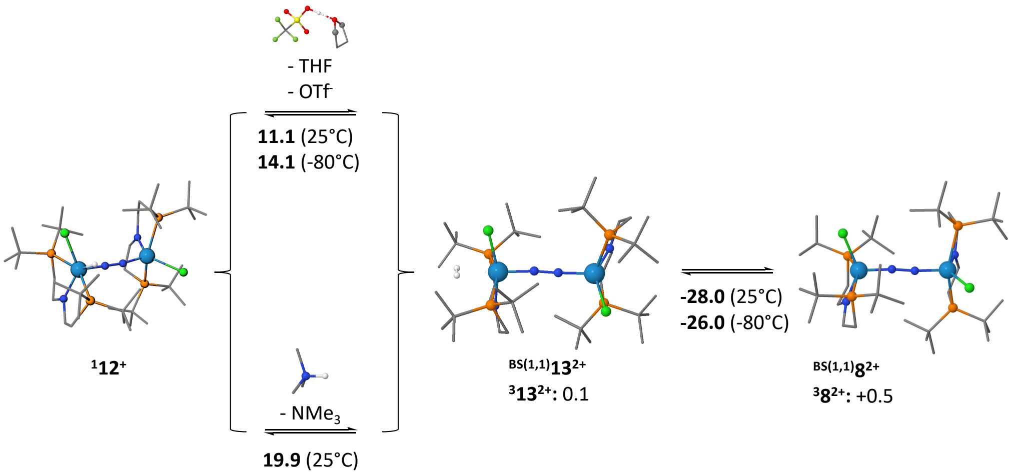

Figure S43 Reaction free energies (in kcal·mol^{-1}) of the second protonation step leading to H_2 evolution via intermediate H_2 complex **13^{2+}**. (For kinetics and a possible transition state see next chapter). The protonation of hydride is accompanied by a spin change to a triplet or BS(1,1) spin state. All hydrogen atoms are omitted except the transferred one.

Table S20 Computed SCF and free energies differences vs. **11^{2+}** and **11^{OTf_2}**, respectively, of the transition states of N_2 splitting (in kcal·mol^{-1}).

			**ΔE | ΔG with correction**	**ΔE | ΔG without correction**
**$^{BS(2,2)}TS^{2+}$**<br>**$11^{2+} \rightarrow 2\ 9^{+}$**	ε_{LS} = -4216.717583 H<br>ε_{HS} = -4216.691239 H<br>E_{ZPE} = 1.2680581H	$<S^2>_{LS}$ = 1.460<br>$<S^2>_{HS}$ = 6.049<br>ΔG = 741.09 kcal·mol^{-1}	15.1 | 16.4<br>J_A = -1260 cm^{-1}	20.4 | 21.7
**$^{BS(2,2)}TS^{OTf_2}$**<br>**$11^{OTf_2} \rightarrow 2\ 9^{OTf}$**	ε_{LS} = -6140.149862H<br>ε_{HS} = -6140.124247H<br>E_{ZPE} = 1.329434 H	$<S^2>_{LS}$ = 1.482<br>$<S^2>_{HS}$ = 6.051<br>ΔG = 769.34 kcal·mol^{-1}	14.0 | 15.1<br>J_A = -1231 cm^{-1}	19.2 | 20.3

4.4 Kinetics of protonation

4.4.1 Hydrogen evaluation reaction

To locate the transition state of hydrogen evolution, the reaction coordinate of the reaction of **12^{+}** with $HNMe_3^+$ was evaluated (Figure S42). The reaction is essentially uphill and the product, dihydrogen complex **13^{2+}**, is unstable with regard to **8^{2+}** and H_2 and should easily release dihydrogen in an exothermic and exergonic process. Although a singlet ground state was computed for **12^{+}**, the scan on the BS(1,1) hypersurface does not transform to a closed shell singlet. Possibly, an electronic or structural reconfiguration of the formed W-H species must occur in a preceding step.

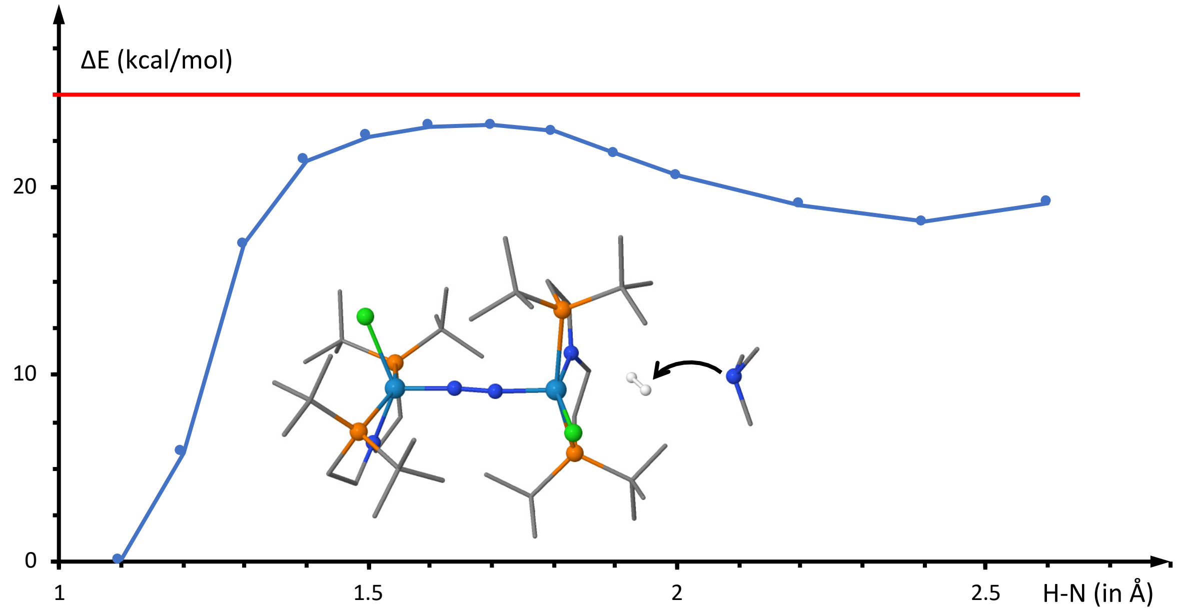

Figure S44 Relaxed Surface Scan of the protonation step of proposed metal hydride intermediate **12^{+}** by $HNMe_3^+$ to form the possible dihydrogen intermediate **13^{2+}**. The reaction coordinate was evaluated in the reverse way starting from **13^{2+}** and NMe_3 as depending on the H-N distance (arrow) on the BS(1,1) surface. The graph shows the single point energies (kcal·mol^{-1}) relative to **12^{+}** and $HNMe_3^+$ on the M06/def2-TZVPP-SMD(THF)//D3BJ-RIJCOSX-PBE0/def2-SVP level of theory. For comparison, the energy of single **13^{2+}** and NMe_3 is demonstated by the red line.

Starting from this scan, a transition state was located on the BS(1,1) and Triplet surface (see Figure S43). However, the calculated barrier (**^{3}TS:** ΔE = 14.0; ΔG = 25.4 kcal·mol^{-1} vs. **12^{+}** and $HNMe_3^+$) is considerable higher than experimentally determined. Entropy contributes strongly to this calculated value (just compare to **13^{2+}:** ΔE

= 20.0; ΔH = 19.9 kcal·mol^{-1} vs. **12$^+$**!). Alternative effects (stabilization of the transition state by additional van-der-Waals attraction with the ethyl groups of NEt_3) and pathways (intermediate protonation of the pincer backbone and/or proton shuttle steps involving the solvent) might play a role and cannot be ruled out on this stage of investigation. A thorough study of the actual, detailed mechanism of H_2 evaluation requires additional computational investigation exceeding the scope of this work. We therefore decided to use the energy of the hydrogen complex as estimate for the kinetic barrier, which is close to the experimental value.

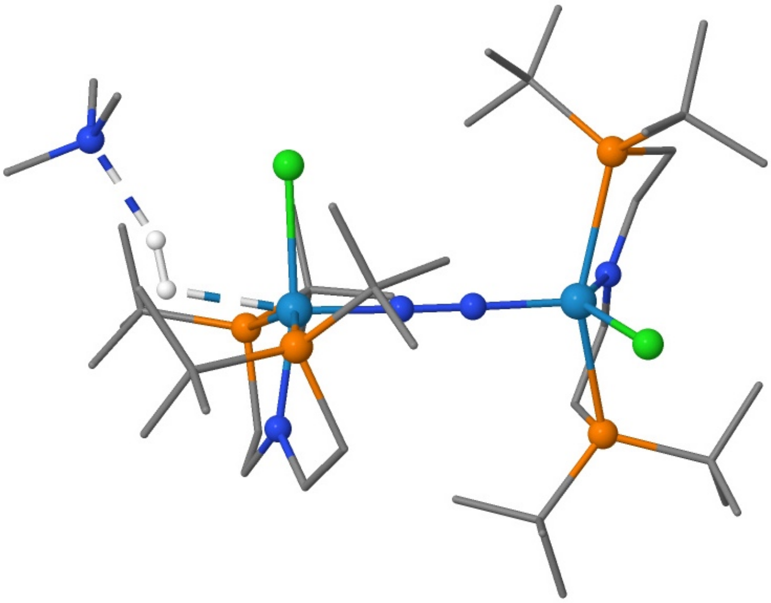

Figure S45 Located transition state of the reaction of **12$^+$** with $HNMe_3^+$.

4.4.2 Formation of **11$^{(OTf)2}$** from **10^{OTf}** and HOTf

The protonation step of **10^{OTf}** with HOTf to **11$^{(OTf)2}$** was investigated by a relaxed surface scan to assure that N_2 splitting (**TS 11$^{(OTf)2}$** $\rightarrow$ 2 **9^{OTf}**) is the rate determining step on the N_2 splitting branch starting from **6**. Movement of the triflate is quite unhindered resulting in a very flat potential energy surface, especially at long bonding distances. For this reason, optimizations did not converge even after 500 and more steps with applying loose convergence criteria. The relative energies of the lowest energy steps of each constrained optimization are plotted for the $m_s = 2$ and $m_s = 0$ (closed shell singlet) surfaces in Figure S44. Despite full convergence, the scan clearly indicates that this step is not connected with any sizable barrier but with a spin change. For **10^{OTf}** and **10$^+$** singlet ground states are computed and quintet ground states for **11$^{(OTf)2}$** and **11^{2+}**, which is in agreement with the NMR and Evans' data for the current system and the analogous molybdenum compounds.[9]

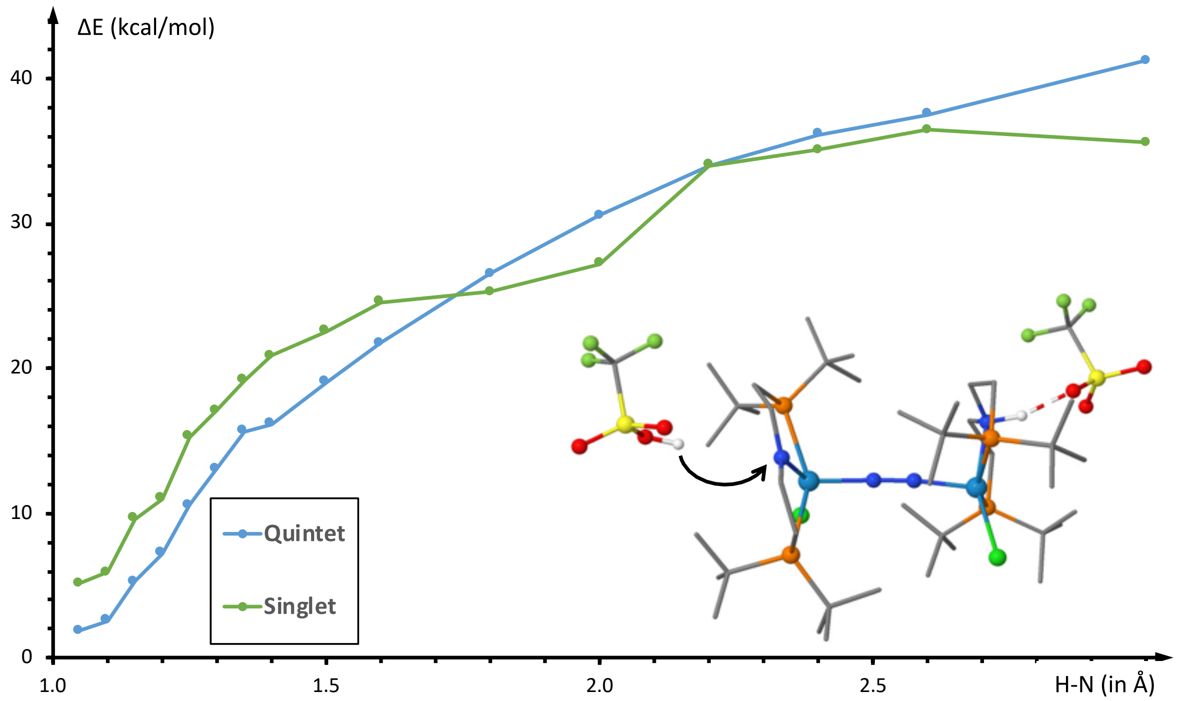

Figure S46 Relaxed Surface Scan of the protonation step of **10^{OTf}** with HOTf leading to **11$^{(OTf)2}$**. Relative energies of the lowest optimization step after 500 cycles at the D3BJ-RIJCOSX-PBE0/def2-SVP level of theory. The reaction coordinate was evaluated by elongation of the N-H bond at one pincer side of **11$^{(OTf)2}$**.

4.5 Structures

Table S21 Computed structures (all hydrogens except protons on the pincer backbone or metal are omitted for clarity) showing H in white, C in grey, N in blue, O in red, F in light green, P in orange, S in yellow, Cl in green and W in light blue.

^{1}**6**

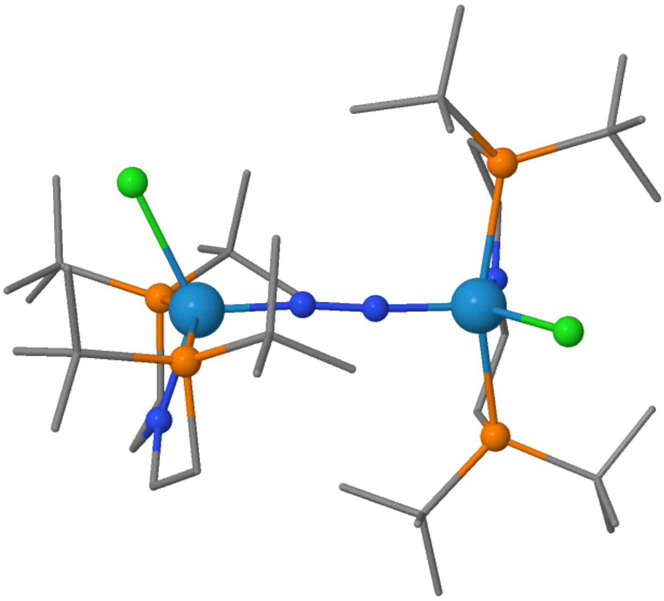

^{2}**7**$^{+}$

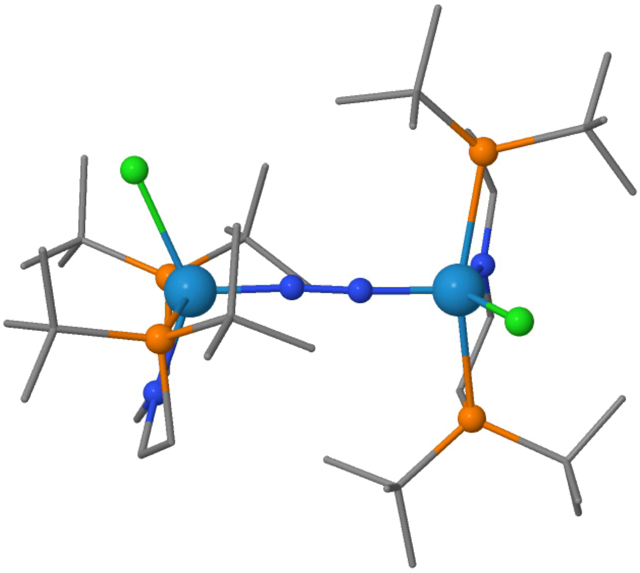

$^{BS(1,1)}\mathbf{8}^{2+}$

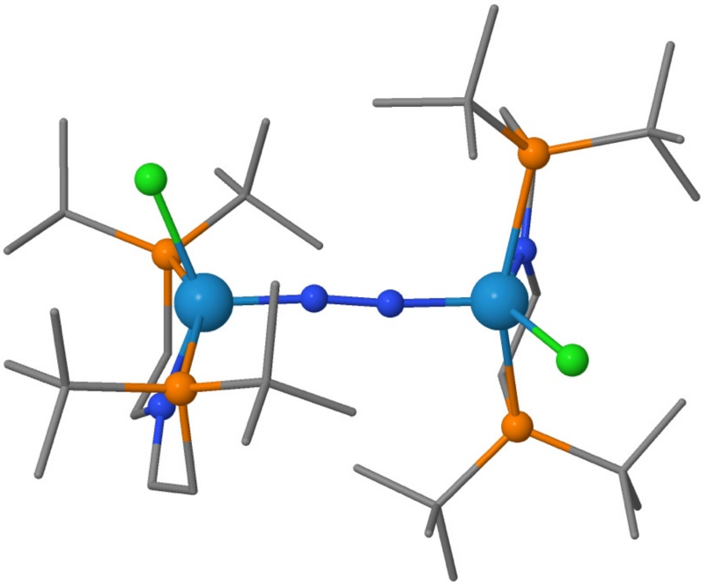

$^{2}\mathbf{9}^{+}$

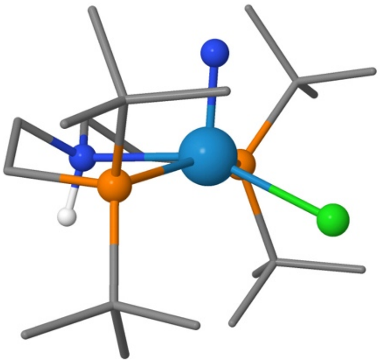

$^{2}\mathbf{9}^{OTf}$

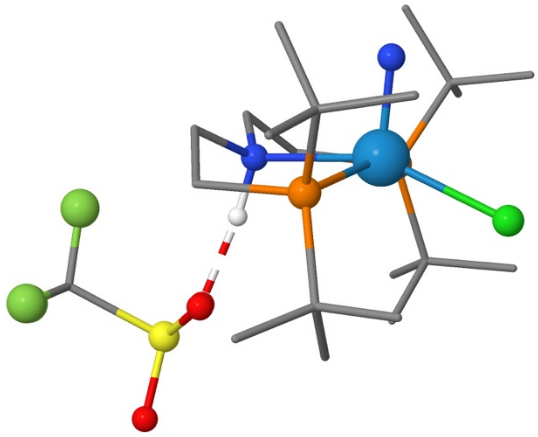

$^{1}\mathbf{10}^{+}$

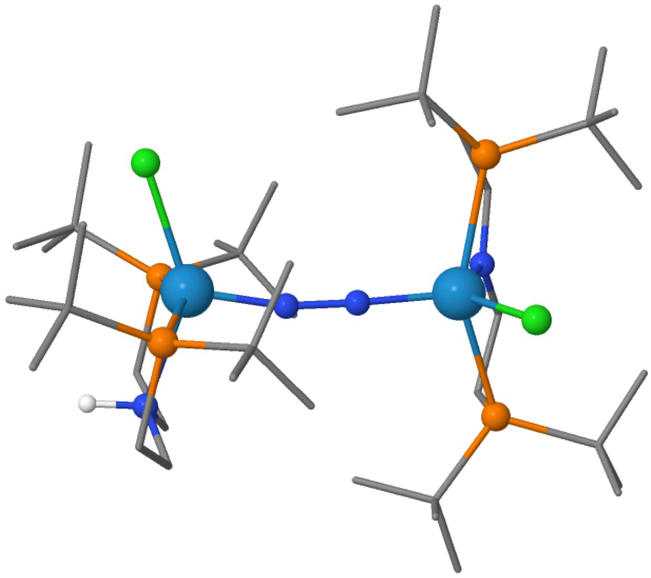

$^{1}\mathbf{10}^{OTf}$

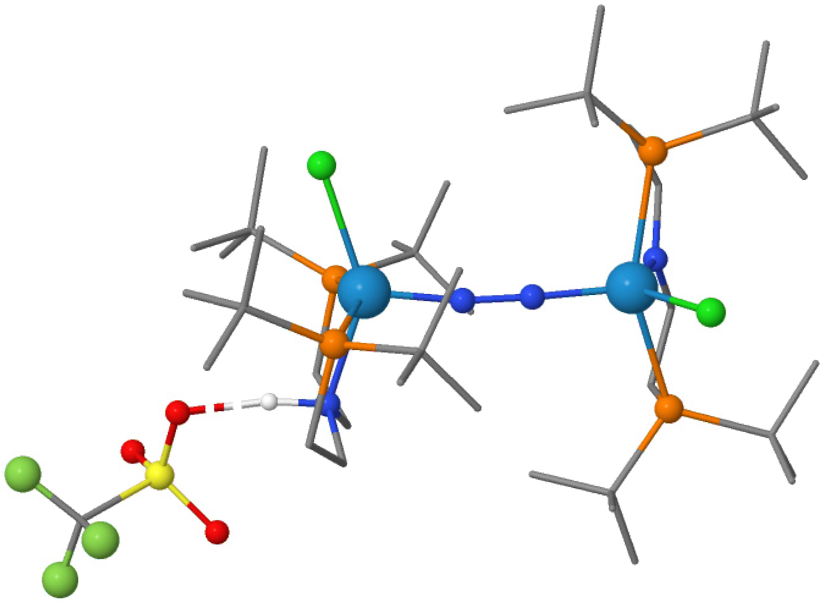

$^{1}\mathbf{10}^{NMe_3+}$

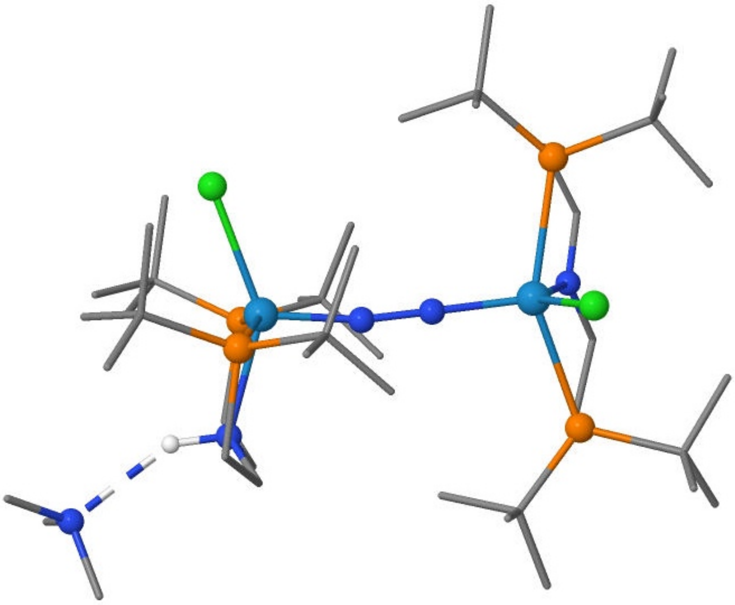

^{5}**11**$^{2+}$

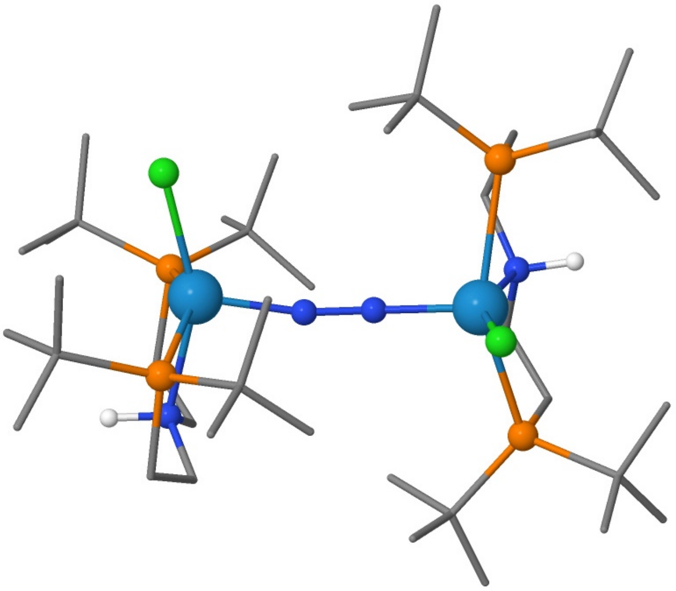

$^{BS(2,2)}$**TS**

11^{2+} $\rightarrow$ 2 **9**$^{+}$

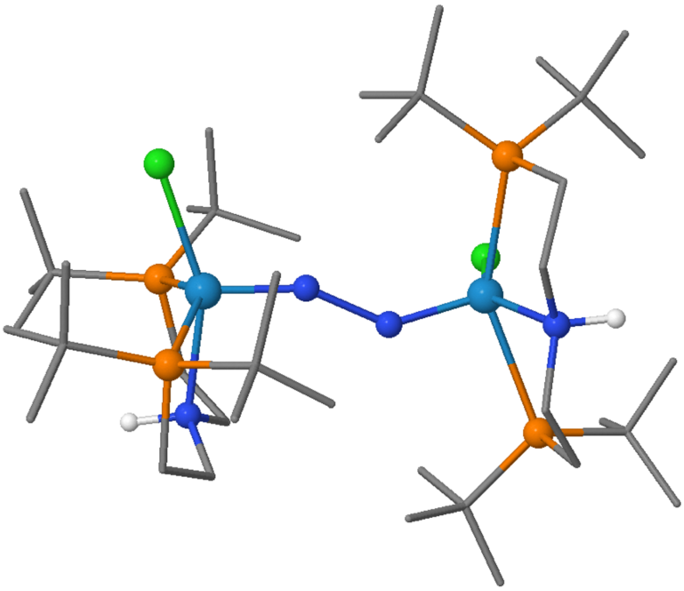

^{5}**11**$^{(OTf)2}$

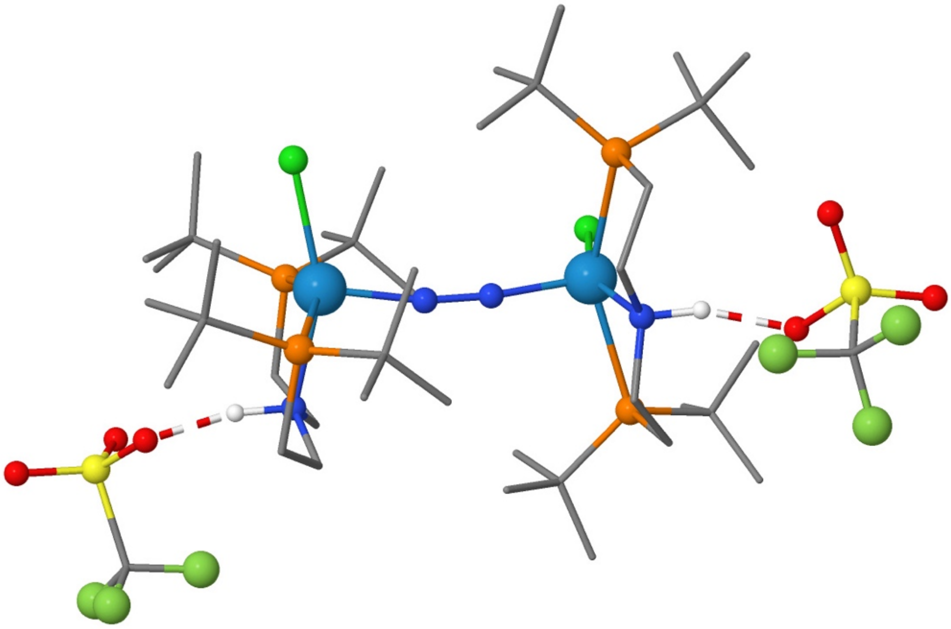

$^{BS(2,2)}$**TS**

$\mathbf{11}^{(\mathbf{OTf})2} \rightarrow 2\ \mathbf{9}^{\mathbf{OTf}}$

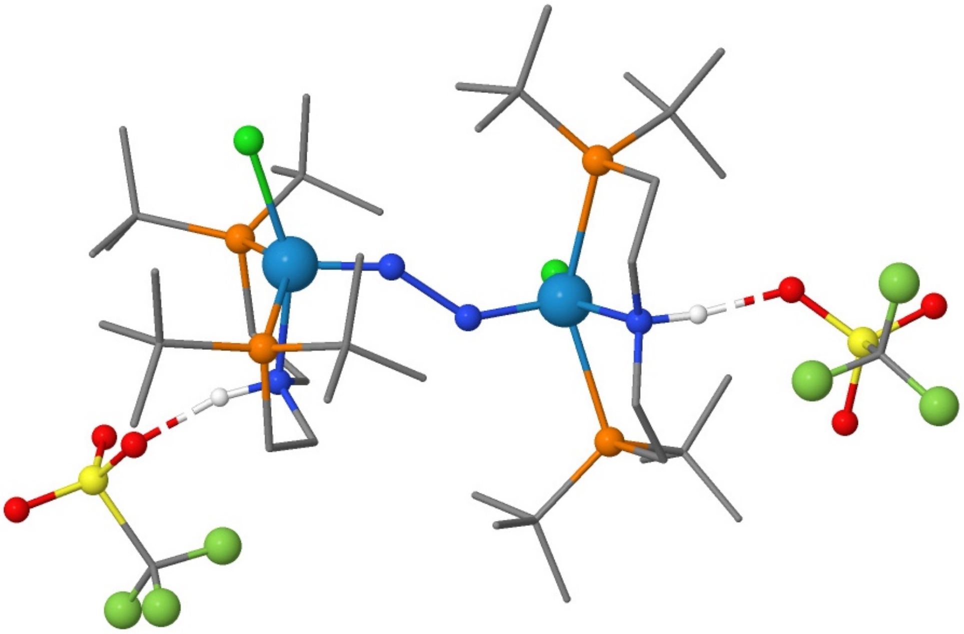

$^{1}\mathbf{12}^{+}$

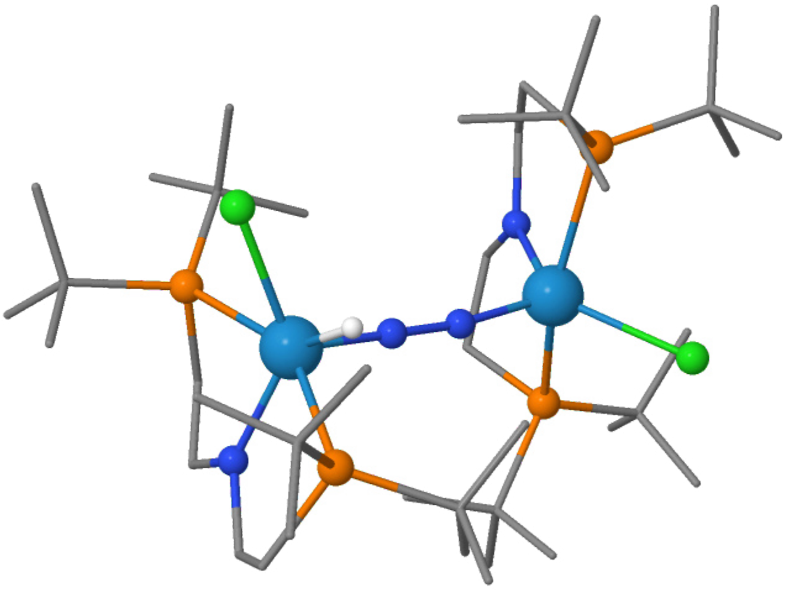

$^{BS(1,1)}\mathbf{13}^{2+}$

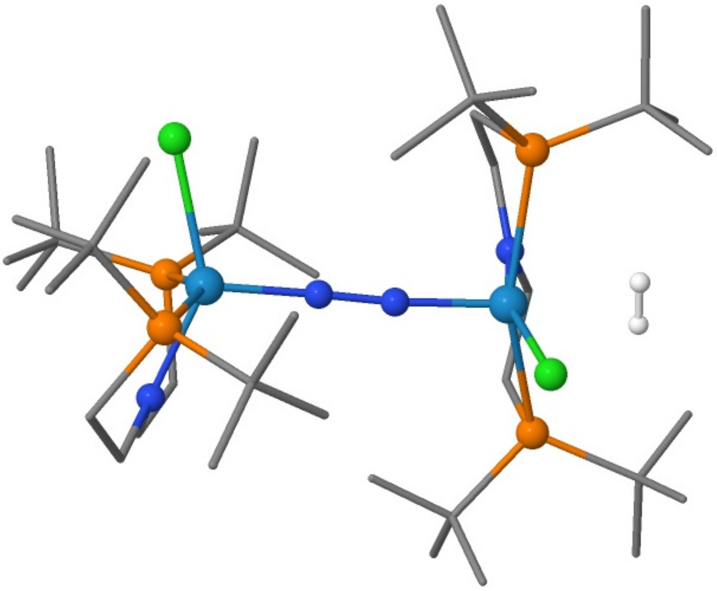

**HOTf**	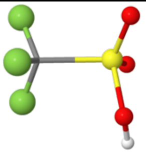
**OTf$^-$**	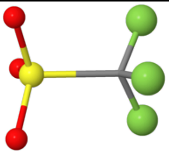
**THF-HOTf**	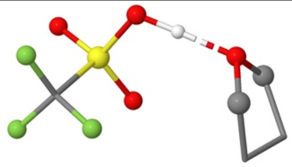
**$(THF)_2H^+$**	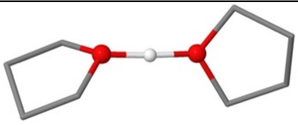

Table S22 Computed structures (xyz coordiantes)

^{1}**6**			
W	0.0308	2.4047	0.0270
C	-2.1820	4.5367	-2.0849
P	-1.9959	2.8058	-1.3177
N	-1.2189	3.0211	1.4739
P	1.6479	2.8264	1.8518
C	2.2639	4.6196	2.0096
Cl	1.5044	3.5230	-1.6081
C	-2.5407	1.4696	-2.5479
C	3.0660	1.6057	2.1359
N	0.1041	0.6100	-0.0310
C	0.4777	2.6091	3.2600
H	0.8387	2.9966	4.2247
H	0.3570	1.5208	3.3571
C	-0.8674	3.2133	2.8818
H	-0.8945	4.2906	3.1510
H	-1.6479	2.7453	3.5148
C	-2.6345	3.3551	1.3051
H	-3.2051	2.9716	2.1738
H	-2.7812	4.4554	1.3317
C	-3.2483	2.7708	0.0407
H	-3.4461	1.7045	0.2143
H	-4.2054	3.2530	-0.2063
C	3.5355	4.8178	1.1819
H	3.7766	5.8938	1.1575
H	3.3855	4.4829	0.1444
H	4.4041	4.2985	1.6088
C	2.4972	5.0343	3.4627
H	2.9010	6.0607	3.4805
H	3.2136	4.3882	3.9859
H	1.5605	5.0461	4.0393
C	1.1875	5.5297	1.4060
H	1.4923	6.5810	1.5424
H	0.2036	5.4046	1.8771
H	1.0880	5.3503	0.3280
C	-1.4284	5.5065	-1.1665
H	-1.5951	6.5382	-1.5196
H	-0.3489	5.3117	-1.2016
H	-1.7636	5.4565	-0.1210
C	-1.5011	4.5828	-3.4544
H	-1.4636	5.6309	-3.7956
H	-2.0454	4.0114	-4.2192
H	-0.4659	4.2142	-3.3916
C	-3.6355	4.9999	-2.1884
H	-3.6599	5.9836	-2.6866
H	-4.0939	5.1311	-1.1973
H	-4.2686	4.3195	-2.7714
C	3.9891	1.9727	3.2962
H	4.7288	1.1658	3.4302
H	3.4368	2.0696	4.2438
H	4.5480	2.9020	3.1224
C	3.8491	1.5055	0.8237
H	4.5938	0.6974	0.9065
H	4.3819	2.4313	0.5757
H	3.1776	1.2744	-0.0150
C	2.4447	0.2373	2.4299
H	3.2339	-0.5312	2.4113
H	1.6971	-0.0561	1.6828
H	1.9819	0.1967	3.4264
C	-2.8136	0.1839	-1.7636
H	-2.9589	-0.6386	-2.4828
H	-3.7302	0.2494	-1.1593
H	-1.9770	-0.1006	-1.1137
C	-3.7990	1.8261	-3.3362
H	-4.0884	0.9617	-3.9574
H	-3.6509	2.6780	-4.0128
H	-4.6482	2.0500	-2.6723
C	-1.3618	1.2042	-3.4868
H	-1.6131	0.3668	-4.1593
H	-0.4651	0.9372	-2.9113
H	-1.1081	2.0710	-4.1090
W	0.0536	-2.4311	-0.0164
C	-1.8367	-4.5896	2.3694
P	-1.7836	-2.8662	1.5685
N	-1.3576	-3.0572	-1.3030
P	1.4282	-2.8094	-2.0374
C	2.0232	-4.5976	-2.3086
Cl	1.7441	-3.5443	1.3967
C	-2.1926	-1.5252	2.8477
C	2.7893	-1.5760	-2.4865
N	0.1175	-0.6361	0.0560
C	0.0857	-2.5754	-3.2782
H	0.3259	-2.9265	-4.2930
H	-0.0655	-1.4877	-3.3237
C	-1.1846	-3.2220	-2.7467
H	-1.2154	-4.2961	-3.0285
H	-2.0520	-2.7694	-3.2678
C	-2.7293	-3.4350	-0.9582
H	-3.4166	-3.0695	-1.7463
H	-2.8444	-4.5392	-0.9704
C	-3.1960	-2.8687	0.3756
H	-3.4459	-1.8092	0.2299
H	-4.1001	-3.3794	0.7387
C	3.4094	-4.8014	-1.6926
H	3.6536	-5.8764	-1.7260
H	3.4218	-4.4861	-0.6384
H	4.2012	-4.2709	-2.2388
C	2.0288	-4.9885	-3.7865
H	2.4325	-6.0104	-3.8848
H	2.6494	-4.3268	-4.4042
H	1.0138	-5.0005	-4.2096
C	1.0597	-5.5237	-1.5569
H	1.3364	-6.5713	-1.7640
H	0.0110	-5.3911	-1.8550
H	1.1390	-5.3656	-0.4736
C	-1.2087	-5.5592	1.3607
H	-1.2784	-6.5849	1.7604
H	-0.1456	-5.3310	1.2124

H	-1.7158	-5.5492	0.3857
C	-0.9635	-4.6035	3.6259
H	-0.8612	-5.6441	3.9768
H	-1.3988	-4.0227	4.4510
H	0.0459	-4.2231	3.4061
C	-3.2518	-5.0748	2.6848
H	-3.1868	-6.0602	3.1763
H	-3.8503	-5.2113	1.7721
H	-3.8021	-4.4069	3.3586
C	3.5382	-1.9141	-3.7741
H	4.2525	-1.1033	-3.9940
H	2.8564	-1.9899	-4.6355
H	4.1147	-2.8460	-3.7030
C	3.7488	-1.4976	-1.2956
H	4.4663	-0.6780	-1.4627
H	4.3202	-2.4221	-1.1488
H	3.2013	-1.2947	-0.3641
C	2.1290	-0.2056	-2.6597
H	2.9102	0.5684	-2.7239
H	1.4849	0.0635	-1.8139
H	1.5386	-0.1428	-3.5852
C	-2.6093	-0.2576	2.0975
H	-2.6988	0.5640	2.8271
H	-3.5903	-0.3632	1.6117
H	-1.8686	0.0533	1.3502
C	-3.3178	-1.9037	3.8084
H	-3.5384	-1.0390	4.4568
H	-3.0531	-2.7431	4.4648
H	-4.2464	-2.1573	3.2744
C	-0.9049	-1.2135	3.6140
H	-1.0891	-0.3779	4.3102
H	-0.1058	-0.9231	2.9189
H	-0.5399	-2.0661	4.1994

^{2}7^{+}

W	-0.0053	2.3668	-0.0034
C	-2.1876	4.7264	1.9157
P	-1.5861	2.9270	1.8376
N	1.2701	3.0445	1.3713
P	1.9734	2.8009	-1.4412
C	2.1038	4.5015	-2.2732
Cl	-1.5121	3.3928	-1.6282
C	-2.9991	1.7194	2.1907
C	2.4682	1.4208	-2.6440
N	-0.0704	0.5481	0.0070
C	3.2606	2.8094	-0.1199
H	4.1993	3.3047	-0.4059
H	3.4865	1.7515	0.0717
C	2.6722	3.4138	1.1446
H	2.7797	4.5168	1.1342
H	3.2770	3.0765	2.0076
C	0.9556	3.3197	2.7779
H	1.7506	2.8805	3.4110
H	1.0030	4.4091	2.9781
C	-0.3843	2.7558	3.2217
H	-0.2786	1.6726	3.3781
H	-0.7159	3.2023	4.1705
C	1.3802	4.4896	-3.6215
H	1.3175	5.5247	-3.9945
H	0.3523	4.1113	-3.5166
H	1.9091	3.9033	-4.3852
C	3.5514	4.9676	-2.4355
H	3.5523	5.9278	-2.9767
H	4.1750	4.2691	-3.0067
H	4.0355	5.1487	-1.4648
C	1.3702	5.4959	-1.3656
H	1.5247	6.5151	-1.7555
H	1.7336	5.4836	-0.3284
H	0.2911	5.2998	-1.3661
C	-1.1190	5.5992	1.2480
H	-1.4146	6.6566	1.3437
H	-1.0432	5.3743	0.1774
H	-0.1260	5.4936	1.7048
C	-3.4784	4.8938	1.1105
H	-3.7132	5.9686	1.0463
H	-4.3398	4.4017	1.5812
H	-3.3596	4.5158	0.0844
C	-2.3837	5.2053	3.3547
H	-2.7830	6.2324	3.3330
H	-1.4343	5.2422	3.9085
H	-3.0915	4.5900	3.9242
C	3.6899	1.7600	-3.4955
H	3.9549	0.8805	-4.1056
H	4.5666	2.0036	-2.8764
H	3.5099	2.5918	-4.1880
C	1.2521	1.1179	-3.5226
H	1.4803	0.2604	-4.1775
H	0.9693	1.9604	-4.1644
H	0.3793	0.8711	-2.9028
C	2.7892	0.1641	-1.8313
H	2.9214	-0.6767	-2.5319
H	1.9808	-0.1038	-1.1401
H	3.7275	0.2601	-1.2659
C	-2.3823	0.3603	2.5326
H	-3.1807	-0.3981	2.5609
H	-1.9018	0.3563	3.5215
H	-1.6516	0.0344	1.7829
C	-3.8936	2.1462	3.3534
H	-4.6388	1.3549	3.5375
H	-4.4470	3.0719	3.1498
H	-3.3218	2.2783	4.2846
C	-3.8102	1.5675	0.9005
H	-4.5670	0.7787	1.0399
H	-3.1618	1.2846	0.0592
H	-4.3339	2.4877	0.6172
W	-0.0579	-2.4666	0.0425
C	-2.1152	-4.6523	-2.1747
P	-1.5350	-2.8580	-1.9791
N	1.3105	-3.1140	-1.2719
P	1.8824	-2.8671	1.6222
C	1.9084	-4.5806	2.4250
Cl	-1.6826	-3.3490	1.5343

C	-2.8938	-1.6146	-2.3722
C	2.2939	-1.4928	2.8494
N	-0.1149	-0.6859	0.1071
C	3.1986	-2.8689	0.3404
H	4.1290	-3.3474	0.6787
H	3.4182	-1.8097	0.1506
C	2.6857	-3.4923	-0.9501
H	2.7831	-4.5948	-0.9178
H	3.3497	-3.1671	-1.7719
C	1.0870	-3.2750	-2.7080
H	1.9408	-2.8336	-3.2557
H	1.0950	-4.3497	-2.9771
C	-0.1897	-2.6130	-3.2026
H	-0.0350	-1.5256	-3.2463
H	-0.4540	-2.9538	-4.2142
C	1.0959	-4.5670	3.7219
H	1.0017	-5.6021	4.0871
H	0.0794	-4.1806	3.5568
H	1.5753	-3.9840	4.5195
C	3.3351	-5.0692	2.6803
H	3.2867	-6.0461	3.1877
H	3.9163	-4.3951	3.3214
H	3.8914	-5.2230	1.7442
C	1.2217	-5.5462	1.4518
H	1.2869	-6.5669	1.8614
H	1.6910	-5.5635	0.4587
H	0.1560	-5.3080	1.3398
C	-1.1362	-5.5497	-1.4091
H	-1.4135	-6.6027	-1.5785
H	-1.1976	-5.3672	-0.3278
H	-0.0928	-5.4292	-1.7290
C	-3.4970	-4.8462	-1.5452
H	-3.7389	-5.9208	-1.5650
H	-4.2932	-4.3247	-2.0922
H	-3.5129	-4.5226	-0.4942
C	-2.1328	-5.0666	-3.6466
H	-2.5276	-6.0926	-3.7218
H	-1.1242	-5.0774	-4.0844
H	-2.7709	-4.4225	-4.2652
C	3.4321	-1.8577	3.8012
H	3.6556	-0.9829	4.4330
H	4.3559	-2.1138	3.2608
H	3.1794	-2.6868	4.4744
C	1.0137	-1.1635	3.6230
H	1.2145	-0.3214	4.3048
H	0.6508	-2.0027	4.2289
H	0.2062	-0.8661	2.9403
C	2.7048	-0.2513	2.0529
H	2.7895	0.5930	2.7553
H	1.9644	0.0372	1.2961
H	3.6872	-0.3683	1.5733
C	-2.2269	-0.2515	-2.5732
H	-3.0064	0.5246	-2.6183
H	-1.6660	-0.1946	-3.5166
H	-1.5585	0.0226	-1.7482
C	-3.6752	-1.9659	-3.6376
H	-4.3952	-1.1570	-3.8409
H	-4.2505	-2.8963	-3.5455
H	-3.0188	-2.0445	-4.5177
C	-3.8188	-1.5286	-1.1542
H	-4.5487	-0.7207	-1.3190
H	-3.2564	-1.2933	-0.2393
H	-4.3797	-2.4539	-0.9776

BS(1,1)**8**$^{2+}$

W	0.0173	2.4008	0.0448
C	-2.1651	4.6087	-2.1167
P	-2.0593	2.8744	-1.3656
N	-1.2212	3.0238	1.4688
P	1.6859	2.8721	1.9129
C	2.2555	4.6739	2.0188
Cl	1.4618	3.3124	-1.5746
C	-2.5661	1.5240	-2.5837
C	3.0956	1.6423	2.1518
N	0.0814	0.6087	-0.0234
C	0.4734	2.6087	3.2634
H	0.8214	2.9916	4.2337
H	0.3654	1.5183	3.3554
C	-0.8675	3.2120	2.8801
H	-0.8976	4.2889	3.1303
H	-1.6526	2.7475	3.5041
C	-2.6216	3.4156	1.2772
H	-3.2060	3.0822	2.1528
H	-2.7051	4.5182	1.2733
C	-3.2503	2.8201	0.0287
H	-3.4506	1.7549	0.2084
H	-4.2083	3.3032	-0.2097
C	3.5321	4.8797	1.1999
H	3.7597	5.9570	1.1812
H	3.4075	4.5520	0.1576
H	4.4040	4.3740	1.6338
C	2.4730	5.0920	3.4744
H	2.8526	6.1260	3.4876
H	3.2081	4.4685	3.9984
H	1.5373	5.0886	4.0521
C	1.1622	5.5515	1.4004
H	1.4583	6.6071	1.5056
H	0.1851	5.4402	1.8884
H	1.0512	5.3548	0.3264
C	-1.3705	5.5514	-1.2062
H	-1.5239	6.5864	-1.5501
H	-0.2939	5.3474	-1.2643
H	-1.6874	5.5120	-0.1548
C	-1.5145	4.6373	-3.5020
H	-1.4649	5.6850	-3.8372
H	-2.0894	4.0863	-4.2577
H	-0.4848	4.2522	-3.4789
C	-3.6157	5.0905	-2.1884
H	-3.6314	6.0715	-2.6889
H	-4.0542	5.2362	-1.1908
H	-4.2692	4.4219	-2.7623
C	4.0228	2.0307	3.3038

H	4.7745	1.2353	3.4299
H	3.4822	2.1206	4.2579
H	4.5693	2.9644	3.1209
C	3.8656	1.5463	0.8310
H	4.6511	0.7810	0.9321
H	4.3544	2.4868	0.5516
H	3.2071	1.2492	0.0025
C	2.4821	0.2746	2.4614
H	3.2819	-0.4818	2.4404
H	1.7319	-0.0300	1.7212
H	2.0327	0.2327	3.4636
C	-2.8718	0.2496	-1.7923
H	-3.0014	-0.5746	-2.5116
H	-3.8085	0.3247	-1.2215
H	-2.0582	-0.0343	-1.1130
C	-3.8094	1.9002	-3.3900
H	-4.0917	1.0435	-4.0228
H	-3.6454	2.7539	-4.0589
H	-4.6697	2.1226	-2.7413
C	-1.3717	1.2474	-3.5007
H	-1.6370	0.4373	-4.1989
H	-0.4916	0.9327	-2.9220
H	-1.0820	2.1165	-4.1030
W	0.0321	-2.4212	-0.0266
C	-1.8378	-4.6653	2.3733
P	-1.8504	-2.9293	1.6216
N	-1.3651	-3.0459	-1.2959
P	1.4602	-2.8547	-2.0922
C	2.0123	-4.6540	-2.3098
Cl	1.6734	-3.3277	1.3953
C	-2.2077	-1.5845	2.9002
C	2.8204	-1.6161	-2.4975
N	0.0921	-0.6292	0.0527
C	0.0857	-2.5735	-3.2726
H	0.3149	-2.9200	-4.2905
H	-0.0512	-1.4835	-3.3148
C	-1.1822	-3.2172	-2.7408
H	-1.2104	-4.2932	-2.9961
H	-2.0499	-2.7727	-3.2612
C	-2.7263	-3.4588	-0.9390
H	-3.4176	-3.1226	-1.7318
H	-2.7959	-4.5622	-0.9406
C	-3.2054	-2.8866	0.3849
H	-3.4470	-1.8244	0.2426
H	-4.1176	-3.3909	0.7341
C	3.4108	-4.8673	-1.7246
H	3.6395	-5.9437	-1.7672
H	3.4649	-4.5622	-0.6694
H	4.1968	-4.3497	-2.2890
C	1.9790	-5.0493	-3.7871
H	2.3600	-6.0784	-3.8828
H	2.6070	-4.4089	-4.4194
H	0.9579	-5.0479	-4.1948
C	1.0452	-5.5454	-1.5242
H	1.3044	-6.5992	-1.7132
H	-0.0060	-5.4142	-1.8135
H	1.1419	-5.3785	-0.4436
C	-1.1846	-5.6015	1.3509
H	-1.2375	-6.6321	1.7354
H	-0.1228	-5.3643	1.2096
H	-1.6888	-5.5969	0.3749
C	-0.9872	-4.6866	3.6455
H	-0.8869	-5.7317	3.9778
H	-1.4429	-4.1285	4.4738
H	0.0266	-4.3008	3.4655
C	-3.2580	-5.1582	2.6583
H	-3.1914	-6.1491	3.1347
H	-3.8452	-5.2882	1.7378
H	-3.8180	-4.5068	3.3402
C	3.5651	-1.9724	-3.7846
H	4.2925	-1.1737	-4.0004
H	2.8880	-2.0364	-4.6498
H	4.1294	-2.9103	-3.7109
C	3.7759	-1.5493	-1.3022
H	4.5317	-0.7724	-1.4968
H	4.3085	-2.4912	-1.1268
H	3.2458	-1.2830	-0.3762
C	2.1682	-0.2436	-2.6783
H	2.9616	0.5171	-2.7399
H	1.5234	0.0331	-1.8355
H	1.5875	-0.1736	-3.6090
C	-2.6436	-0.3174	2.1592
H	-2.6995	0.5034	2.8919
H	-3.6442	-0.4153	1.7145
H	-1.9300	-0.0147	1.3821
C	-3.3163	-1.9802	3.8758
H	-3.5181	-1.1272	4.5433
H	-3.0419	-2.8296	4.5134
H	-4.2577	-2.2191	3.3591
C	-0.9026	-1.2853	3.6439
H	-1.0830	-0.4770	4.3709
H	-0.1161	-0.9581	2.9496
H	-0.5190	-2.1486	4.2007

^{2}9^{+}

W	-1.2670	0.6977	-0.0602
C	-1.8041	-2.6521	1.5398
P	-2.0828	-1.7153	-0.0710
N	0.3655	-0.3077	-1.2492
P	0.5100	2.5201	-0.0653
C	1.4049	2.7324	1.5783
Cl	-2.3520	1.3449	1.9381
C	-3.7943	-1.9512	-0.8171
C	-0.0108	4.1351	-0.8819
N	-2.1535	1.2312	-1.3667
C	1.7724	1.7321	-1.1752
H	2.5465	1.2793	-0.5353
H	2.2871	2.4840	-1.7920
C	1.1408	0.6679	-2.0550
H	1.9178	0.1342	-2.6290
H	0.4394	1.1133	-2.7741
C	-0.1833	-1.3978	-2.0947

H	-0.8974	-0.9319	-2.7882
H	0.6299	-1.8421	-2.6940
C	-0.8574	-2.4638	-1.2491
H	-1.3356	-3.2160	-1.8933
H	-0.1041	-3.0048	-0.6552
C	0.5762	3.6310	2.4988
H	1.0226	3.6143	3.5056
H	-0.4623	3.2796	2.5874
H	0.5716	4.6765	2.1625
C	2.8240	3.2811	1.4256
H	3.2827	3.3489	2.4248
H	2.8450	4.2859	0.9876
H	3.4707	2.6285	0.8208
C	1.4602	1.3268	2.1895
H	2.0305	1.3604	3.1314
H	1.9681	0.5936	1.5406
H	0.4509	0.9626	2.4354
C	-0.4881	-2.0999	2.1019
H	-0.2289	-2.6419	3.0252
H	-0.5839	-1.0358	2.3676
H	0.3628	-2.2305	1.4122
C	-2.9288	-2.3275	2.5255
H	-2.6729	-2.7539	3.5084
H	-3.8875	-2.7687	2.2215
H	-3.0604	-1.2428	2.6535
C	-1.6817	-4.1638	1.3424
H	-1.4741	-4.6300	2.3187
H	-0.8545	-4.4414	0.6730
H	-2.6018	-4.6167	0.9546
C	1.0762	5.2043	-0.7775
H	0.7531	6.0897	-1.3474
H	2.0341	4.8748	-1.2089
H	1.2504	5.5314	0.2564
C	-1.3114	4.6012	-0.2189
H	-1.6488	5.5245	-0.7158
H	-1.1899	4.8242	0.8487
H	-2.1071	3.8498	-0.3249
C	-0.2888	3.8399	-2.3591
H	-0.6848	4.7565	-2.8237
H	-1.0469	3.0531	-2.4854
H	0.6215	3.5709	-2.9160
C	-3.7172	-1.5512	-2.2932
H	-4.7348	-1.5938	-2.7115
H	-3.0994	-2.2413	-2.8875
H	-3.3515	-0.5223	-2.4228
C	-4.2691	-3.3996	-0.7099
H	-5.2284	-3.4933	-1.2431
H	-4.4440	-3.7095	0.3288
H	-3.5691	-4.1092	-1.1773
C	-4.7584	-1.0017	-0.0978
H	-5.7590	-1.1128	-0.5440
H	-4.4507	0.0471	-0.2142
H	-4.8474	-1.2150	0.9749
H	1.0210	-0.7408	-0.5891

^{2}**9**^{OTf}

W	-1.2884	0.7223	-0.0647
C	-1.8552	-2.6218	1.5966
P	-2.0534	-1.7084	-0.0395
N	0.2920	-0.2367	-1.2952
P	0.5065	2.5226	-0.0885
C	1.4444	2.6881	1.5351
Cl	-2.3403	1.3576	1.9833
C	-3.7321	-1.9889	-0.8503
C	-0.0254	4.1661	-0.8381
N	-2.2375	1.2804	-1.3184
C	1.7076	1.7676	-1.2653
H	2.4894	1.2531	-0.6806
H	2.2140	2.5318	-1.8732
C	1.0185	0.7346	-2.1402
H	1.7768	0.1993	-2.7339
H	0.2947	1.1895	-2.8325
C	-0.2002	-1.3834	-2.0848
H	-0.9410	-1.0090	-2.8056
H	0.6421	-1.8153	-2.6506
C	-0.7855	-2.4450	-1.1657
H	-1.1962	-3.2867	-1.7425
H	0.0260	-2.8399	-0.5349
C	0.7045	3.6388	2.4764
H	1.1773	3.5844	3.4701
H	-0.3513	3.3514	2.5941
H	0.7564	4.6858	2.1466
C	2.8969	3.1236	1.3315
H	3.3646	3.2406	2.3225
H	2.9881	4.0844	0.8081
H	3.4794	2.3623	0.7933
C	1.4243	1.2816	2.1449
H	2.0472	1.2742	3.0540
H	1.8411	0.5185	1.4737
H	0.4045	0.9973	2.4449
C	-0.6113	-2.0124	2.2557
H	-0.4010	-2.5596	3.1892
H	-0.7936	-0.9625	2.5304
H	0.2962	-2.0715	1.6348
C	-3.0626	-2.3508	2.4959
H	-2.8502	-2.7569	3.4980
H	-3.9799	-2.8370	2.1355
H	-3.2478	-1.2717	2.6067
C	-1.6448	-4.1245	1.4070
H	-1.5025	-4.5849	2.3981
H	-0.7426	-4.3452	0.8191
H	-2.5002	-4.6224	0.9332
C	1.0905	5.2080	-0.7634
H	0.7638	6.1200	-1.2892
H	2.0138	4.8625	-1.2537
H	1.3339	5.4933	0.2690
C	-1.2859	4.6481	-0.1143
H	-1.6363	5.5794	-0.5888
H	-1.1116	4.8585	0.9484
H	-2.0916	3.9029	-0.1868
C	-0.3755	3.9119	-2.3076
H	-0.7692	4.8469	-2.7378

H	-1.1521	3.1398	-2.4125
H	0.5037	3.6271	-2.9043
C	-3.6133	-1.5989	-2.3261
H	-4.6126	-1.6756	-2.7834
H	-2.9480	-2.2732	-2.8857
H	-3.2701	-0.5609	-2.4456
C	-4.1767	-3.4483	-0.7592
H	-5.1109	-3.5705	-1.3313
H	-4.3812	-3.7630	0.2726
H	-3.4364	-4.1378	-1.1939
C	-4.7528	-1.0602	-0.1851
H	-5.7268	-1.1865	-0.6850
H	-4.4516	-0.0075	-0.2846
H	-4.8931	-1.2758	0.8815
H	1.0023	-0.6595	-0.6425
C	3.8285	-2.3423	-1.3594
S	3.4464	-1.5049	0.2466
O	4.1427	-2.2804	1.2556
O	3.8761	-0.1156	0.0246
O	1.9514	-1.6404	0.2784
F	5.1261	-2.3524	-1.6040
F	3.2188	-1.6992	-2.3693
F	3.3807	-3.5909	-1.3548

^{1}10^{+}

W	-0.1443	2.5076	-0.0427
C	1.7240	4.5923	2.4578
P	1.8009	2.9969	1.4402
N	1.3236	3.2328	-1.5451
P	-1.6577	2.9080	-1.9954
C	-2.6960	4.4889	-1.9531
Cl	-1.7701	3.2484	1.5763
C	2.5137	1.5452	2.4167
C	-2.6127	1.4179	-2.6477
N	0.0515	0.7553	-0.2046
C	-0.4022	3.3094	-3.3223
H	-0.3774	4.4024	-3.4547
H	-0.6942	2.8913	-4.2966
C	0.9745	2.8117	-2.9269
H	1.7399	3.1856	-3.6300
H	1.0159	1.7147	-2.9416
C	2.7233	2.8812	-1.1950
H	2.7837	1.7860	-1.2142
H	3.4065	3.2678	-1.9720
C	3.1059	3.4223	0.1689
H	4.1001	3.0533	0.4614
H	3.1917	4.5192	0.1223
C	-3.9401	4.2444	-1.0963
H	-4.4558	5.2036	-0.9273
H	-3.6772	3.8257	-0.1137
H	-4.6552	3.5738	-1.5928
C	-3.0914	5.0177	-3.3324
H	-3.6369	5.9662	-3.1990
H	-3.7535	4.3356	-3.8772
H	-2.2232	5.2375	-3.9711
C	-1.8145	5.5356	-1.2595
H	-2.3506	6.4976	-1.2249
H	-0.8704	5.7233	-1.7981
H	-1.5949	5.2514	-0.2204
C	0.9852	5.6002	1.5672
H	0.9288	6.5703	2.0865
H	-0.0475	5.2796	1.3698
H	1.5028	5.7874	0.6115
C	0.8887	4.3435	3.7155
H	0.6919	5.3066	4.2138
H	1.4148	3.7056	4.4395
H	-0.0821	3.8877	3.4707
C	3.0883	5.1759	2.8275
H	2.9268	6.1326	3.3504
H	3.7120	5.3962	1.9487
H	3.6618	4.5304	3.5019
C	-3.5822	1.7445	-3.7802
H	-4.0030	0.7999	-4.1613
H	-3.0907	2.2456	-4.6279
H	-4.4257	2.3644	-3.4477
C	-3.3572	0.8062	-1.4612
H	-3.8788	-0.1049	-1.7928
H	-4.1043	1.4870	-1.0323
H	-2.6602	0.5239	-0.6623
C	-1.5770	0.4093	-3.1512
H	-2.0817	-0.5469	-3.3609
H	-0.8078	0.1987	-2.3983
H	-1.0916	0.7338	-4.0837
C	3.0277	0.5183	1.4060
H	3.3415	-0.3825	1.9569
H	3.9046	0.8775	0.8465
H	2.2469	0.2077	0.7011
C	3.6544	1.9343	3.3529
H	4.0775	1.0163	3.7929
H	3.3188	2.5674	4.1848
H	4.4736	2.4497	2.8291
C	1.3612	0.9159	3.2006
H	1.7410	0.0466	3.7614
H	0.5720	0.5715	2.5188
H	0.9079	1.6092	3.9208
W	-0.0221	-2.3037	0.0310
C	1.9789	-4.8901	-1.9012
P	1.8947	-3.0639	-1.3783
N	1.2705	-2.8743	1.4449
P	-1.5772	-2.7330	1.9587
C	-2.1628	-4.5173	2.2597
Cl	-1.5874	-3.4817	-1.4381
C	2.3861	-1.9528	-2.8306
C	-2.9948	-1.5175	2.2304
N	-0.0333	-0.4964	-0.1763
C	-0.3516	-2.4133	3.2906
H	-0.6592	-2.7690	4.2841
H	-0.2620	-1.3201	3.3404
C	0.9846	-3.0049	2.8780
H	1.0439	-4.0696	3.1827
H	1.7881	-2.5009	3.4498
C	2.6665	-3.2749	1.2394

H	3.2932	-2.8144	2.0275
H	2.7755	-4.3678	1.3915
C	3.2224	-2.8732	-0.1162
H	3.4568	-1.8001	-0.0978
H	4.1501	-3.4147	-0.3483
C	-3.5050	-4.7802	1.5743
H	-3.7136	-5.8612	1.6261
H	-3.4765	-4.4955	0.5124
H	-4.3412	-4.2666	2.0669
C	-2.2558	-4.8347	3.7532
H	-2.6722	-5.8486	3.8696
H	-2.9075	-4.1465	4.3062
H	-1.2677	-4.8376	4.2353
C	-1.1379	-5.4554	1.6206
H	-1.3941	-6.4962	1.8775
H	-0.1108	-5.2734	1.9646
H	-1.1663	-5.3634	0.5291
C	1.2088	-5.6957	-0.8534
H	1.3294	-6.7703	-1.0665
H	0.1393	-5.4622	-0.9020
H	1.5661	-5.5209	0.1706
C	1.2782	-5.1033	-3.2447
H	1.1920	-6.1873	-3.4234
H	1.8390	-4.6809	-4.0893
H	0.2609	-4.6851	-3.2358
C	3.4158	-5.4130	-1.9532
H	3.3957	-6.4476	-2.3325
H	3.8758	-5.4492	-0.9552
H	4.0721	-4.8345	-2.6151
C	-3.7629	-1.7616	3.5279
H	-4.5102	-0.9610	3.6528
H	-3.1055	-1.7348	4.4105
H	-4.3068	-2.7152	3.5254
C	-3.9304	-1.5967	1.0187
H	-4.5852	-0.7107	1.0113
H	-4.5738	-2.4830	1.0425
H	-3.3670	-1.6202	0.0754
C	-2.3830	-0.1173	2.2809
H	-3.1878	0.6332	2.2884
H	-1.7574	0.0900	1.4046
H	-1.7809	0.0525	3.1847
C	2.6606	-0.5553	-2.2744
H	2.8289	0.1278	-3.1232
H	3.5639	-0.5184	-1.6483
H	1.8113	-0.1803	-1.6886
C	3.6374	-2.4291	-3.5668
H	3.8974	-1.6927	-4.3451
H	3.4902	-3.3924	-4.0715
H	4.5045	-2.5159	-2.8943
C	1.1911	-1.8563	-3.7842
H	1.3705	-1.0489	-4.5133
H	0.2632	-1.6377	-3.2381
H	1.0289	-2.7799	-4.3504
H	1.2635	4.2581	-1.5269

^{1}**10**$^{NMe3+}$

W	-0.2501	2.5226	0.2122
C	1.5425	4.4979	2.8060
P	1.6729	2.9567	1.7161
N	1.2045	3.3199	-1.2759
P	-1.7700	2.9060	-1.7464
C	-2.8508	4.4536	-1.6730
Cl	-1.9295	3.1313	1.8340
C	2.3964	1.4745	2.6535
C	-2.6998	1.4099	-2.4216
N	-0.0148	0.7784	0.0488
C	-0.5102	3.3329	-3.0575
H	-0.4872	4.4275	-3.1692
H	-0.7919	2.9212	-4.0375
C	0.8639	2.8407	-2.6408
H	1.6284	3.1876	-3.3556
H	0.9006	1.7433	-2.6250
C	2.6090	2.9937	-0.9206
H	2.7132	1.9067	-0.9992
H	3.2874	3.4397	-1.6689
C	2.9787	3.4633	0.4760
H	3.9734	3.0844	0.7546
H	3.0488	4.5610	0.4976
C	-4.1095	4.1323	-0.8650
H	-4.6547	5.0666	-0.6538
H	-3.8553	3.6655	0.0988
H	-4.7946	3.4726	-1.4147
C	-3.2170	5.0349	-3.0402
H	-3.8429	5.9288	-2.8854
H	-3.7920	4.3399	-3.6626
H	-2.3350	5.3600	-3.6121
C	-2.0148	5.4832	-0.9043
H	-2.5428	6.4508	-0.8933
H	-1.0340	5.6576	-1.3694
H	-1.8688	5.1775	0.1417
C	0.7862	5.5284	1.9564
H	0.6943	6.4677	2.5254
H	-0.2343	5.1905	1.7243
H	1.3069	5.7725	1.0163
C	0.6949	4.1454	4.0306
H	0.4431	5.0668	4.5802
H	1.2349	3.4890	4.7270
H	-0.2491	3.6614	3.7362
C	2.8803	5.1074	3.2275
H	2.6782	6.0034	3.8365
H	3.4857	5.4356	2.3697
H	3.4883	4.4318	3.8391
C	-3.6517	1.7423	-3.5672
H	-4.0676	0.8026	-3.9663
H	-3.1414	2.2493	-4.4006
H	-4.4996	2.3628	-3.2469
C	-3.4476	0.7695	-1.2526
H	-3.9188	-0.1650	-1.5961
H	-4.2338	1.4135	-0.8380
H	-2.7540	0.5193	-0.4398
C	-1.6435	0.4217	-2.9174
H	-2.1295	-0.5366	-3.1585

H	-0.8957	0.2043	-2.1449
H	-1.1334	0.7716	-3.8268
C	2.9560	0.4949	1.6215
H	3.3056	-0.4051	2.1535
H	3.8160	0.9051	1.0716
H	2.1906	0.1661	0.9093
C	3.5105	1.8587	3.6234
H	3.9456	0.9380	4.0462
H	3.1457	2.4613	4.4658
H	4.3270	2.4052	3.1274
C	1.2511	0.7789	3.3923
H	1.6542	-0.0943	3.9314
H	0.4922	0.4229	2.6842
H	0.7527	1.4265	4.1247
W	-0.0153	-2.2867	0.1231
C	1.9392	-4.6375	-2.1210
P	1.9116	-2.9014	-1.3548
N	1.2696	-3.0314	1.4753
P	-1.5851	-2.8950	2.0000
C	-2.2755	-4.6669	2.0248
Cl	-1.6109	-3.1860	-1.5023
C	2.4729	-1.6332	-2.6454
C	-2.9351	-1.6575	2.4707
N	-0.0485	-0.4750	0.0499
C	-0.3623	-2.8440	3.3716
H	-0.6907	-3.3759	4.2754
H	-0.2679	-1.7802	3.6295
C	0.9767	-3.3609	2.8756
H	1.0477	-4.4582	3.0264
H	1.7735	-2.9354	3.5157
C	2.6638	-3.4234	1.2384
H	3.2920	-3.0306	2.0609
H	2.7635	-4.5263	1.3102
C	3.2374	-2.9227	-0.0762
H	3.5415	-1.8746	0.0467
H	4.1299	-3.4921	-0.3708
C	-3.6165	-4.7388	1.2907
H	-3.8799	-5.7995	1.1462
H	-3.5606	-4.2685	0.2981
H	-4.4322	-4.2791	1.8643
C	-2.4242	-5.2103	3.4474
H	-2.9110	-6.1983	3.3967
H	-3.0434	-4.5752	4.0924
H	-1.4511	-5.3575	3.9374
C	-1.2875	-5.5530	1.2663
H	-1.6016	-6.6052	1.3648
H	-0.2590	-5.4758	1.6470
H	-1.2900	-5.3043	0.1985
C	1.1602	-5.5557	-1.1785
H	1.2597	-6.5954	-1.5308
H	0.0939	-5.3010	-1.1870
H	1.5255	-5.5189	-0.1421
C	1.2148	-4.6357	-3.4688
H	1.0654	-5.6796	-3.7898
H	1.7959	-4.1368	-4.2559
H	0.2239	-4.1638	-3.3912

C	3.3578	-5.1897	-2.2743
H	3.3025	-6.1547	-2.8044
H	3.8304	-5.3869	-1.3012
H	4.0213	-4.5381	-2.8548
C	-3.6783	-2.0274	3.7537
H	-4.3989	-1.2276	3.9884
H	-2.9969	-2.1136	4.6136
H	-4.2519	-2.9593	3.6620
C	-3.9077	-1.5362	1.2925
H	-4.4821	-0.6013	1.3895
H	-4.6268	-2.3615	1.2571
H	-3.3767	-1.5133	0.3313
C	-2.2544	-0.3043	2.6833
H	-3.0245	0.4708	2.8141
H	-1.6432	-0.0103	1.8219
H	-1.6224	-0.2833	3.5825
C	2.7527	-0.3307	-1.9003
H	2.9754	0.4504	-2.6444
H	3.6263	-0.3978	-1.2368
H	1.8861	-0.0135	-1.3069
C	3.7426	-2.0289	-3.3968
H	4.0375	-1.1974	-4.0576
H	3.6023	-2.9099	-4.0359
H	4.5836	-2.2179	-2.7130
C	1.3210	-1.3825	-3.6214
H	1.5313	-0.4744	-4.2102
H	0.3718	-1.2434	-3.0881
H	1.1821	-2.2051	-4.3305
H	1.1627	4.3570	-1.3471
N	1.7527	6.2283	-2.3677
C	2.2892	5.8326	-3.6587
H	3.0812	5.0794	-3.5320
H	1.4960	5.4005	-4.2871
H	2.7300	6.6890	-4.2105
C	2.8063	6.7636	-1.5253
H	3.5874	6.0065	-1.3622
H	3.2895	7.6573	-1.9727
H	2.3996	7.0563	-0.5457
C	0.6972	7.2092	-2.5435
H	1.0556	8.1222	-3.0628
H	-0.1216	6.7861	-3.1435
H	0.2845	7.5100	-1.5699

$^{1}\mathbf{10}^{\mathbf{OTf}}$

W	-0.1273	2.4283	0.0427
C	1.6452	4.5397	2.6521
P	1.7998	3.0222	1.5356
N	1.2834	3.2554	-1.3953
P	-1.6401	2.9025	-1.8879
C	-2.8605	4.3437	-1.7699
Cl	-1.7901	3.0492	1.6987
C	2.5540	1.5419	2.4356
C	-2.4462	1.3672	-2.6486
N	0.0848	0.6747	-0.1015
C	-0.4162	3.5012	-3.1536
H	-0.3994	4.6011	-3.1055

H	-0.7074	3.2247	-4.1779
C	0.9781	2.9993	-2.8232
H	1.7151	3.5405	-3.4389
H	1.0809	1.9210	-3.0054
C	2.7164	3.0208	-1.0918
H	2.9048	1.9408	-1.1679
H	3.3251	3.5381	-1.8526
C	3.0643	3.5560	0.2861
H	4.0804	3.2522	0.5781
H	3.0707	4.6566	0.2450
C	-4.1083	3.8962	-1.0069
H	-4.7278	4.7817	-0.7890
H	-3.8396	3.4315	-0.0459
H	-4.7299	3.1982	-1.5864
C	-3.2339	4.9373	-3.1297
H	-3.8906	5.8067	-2.9607
H	-3.7766	4.2381	-3.7767
H	-2.3535	5.3058	-3.6757
C	-2.1459	5.4268	-0.9508
H	-2.7973	6.3151	-0.9003
H	-1.1881	5.7486	-1.3852
H	-1.9707	5.0929	0.0810
C	0.8329	5.5670	1.8537
H	0.7384	6.4840	2.4587
H	-0.1856	5.2058	1.6535
H	1.3016	5.8567	0.9027
C	0.8665	4.1653	3.9141
H	0.6381	5.0859	4.4761
H	1.4413	3.5090	4.5833
H	-0.0886	3.6794	3.6639
C	2.9946	5.1678	3.0065
H	2.8075	6.0601	3.6265
H	3.5373	5.5116	2.1141
H	3.6468	4.4995	3.5814
C	-3.4069	1.6842	-3.7922
H	-3.7473	0.7351	-4.2394
H	-2.9275	2.2687	-4.5919
H	-4.3004	2.2249	-3.4538
C	-3.1697	0.6126	-1.5331
H	-3.6360	-0.2936	-1.9543
H	-3.9577	1.2107	-1.0579
H	-2.4595	0.3050	-0.7545
C	-1.3289	0.4727	-3.1853
H	-1.7765	-0.4717	-3.5351
H	-0.6014	0.2221	-2.4033
H	-0.8042	0.9250	-4.0397
C	3.1121	0.6027	1.3646
H	3.4063	-0.3467	1.8399
H	4.0032	1.0178	0.8710
H	2.3615	0.3621	0.6024
C	3.6814	1.9111	3.3964
H	4.1254	0.9809	3.7885
H	3.3282	2.4939	4.2575
H	4.4867	2.4743	2.9008
C	1.4285	0.8230	3.1806
H	1.8281	-0.0895	3.6517
H	0.6289	0.5252	2.4905
H	0.9795	1.4446	3.9663
W	0.0260	-2.3839	-0.0633
C	-2.1158	-4.6826	2.0383
P	-1.9091	-2.8897	1.4356
N	-1.3314	-3.0138	-1.4006
P	1.5101	-3.0131	-1.9634
C	2.1024	-4.8188	-2.0730
Cl	1.5334	-3.5302	1.5116
C	-2.2871	-1.6819	2.8360
C	2.9286	-1.8422	-2.4064
N	0.1324	-0.5825	-0.0151
C	0.2699	-2.8555	-3.3163
H	0.5503	-3.3565	-4.2538
H	0.2055	-1.7781	-3.5191
C	-1.0787	-3.3391	-2.8067
H	-1.1811	-4.4322	-2.9725
H	-1.8724	-2.8825	-3.4306
C	-2.7585	-3.2307	-1.1482
H	-3.3431	-2.7546	-1.9602
H	-3.0010	-4.3119	-1.2198
C	-3.2378	-2.6716	0.1817
H	-3.3600	-1.5845	0.0880
H	-4.2110	-3.0944	0.4696
C	3.4352	-4.9967	-1.3432
H	3.6530	-6.0751	-1.2705
H	3.3841	-4.5956	-0.3201
H	4.2759	-4.5309	-1.8749
C	2.2110	-5.3111	-3.5172
H	2.6284	-6.3317	-3.5089
H	2.8661	-4.6926	-4.1433
H	1.2266	-5.3720	-4.0037
C	1.0689	-5.6806	-1.3449
H	1.3236	-6.7443	-1.4849
H	0.0463	-5.5316	-1.7176
H	1.0874	-5.4689	-0.2698
C	-1.4180	-5.5939	1.0271
H	-1.6180	-6.6459	1.2909
H	-0.3336	-5.4387	1.0620
H	-1.7635	-5.4372	-0.0037
C	-1.4202	-4.8871	3.3855
H	-1.4098	-5.9661	3.6127
H	-1.9412	-4.3861	4.2124
H	-0.3767	-4.5411	3.3505
C	-3.5873	-5.0906	2.1293
H	-3.6458	-6.1033	2.5617
H	-4.0613	-5.1368	1.1382
H	-4.1848	-4.4249	2.7650
C	3.7074	-2.2581	-3.6528
H	4.4567	-1.4823	-3.8814
H	3.0547	-2.3528	-4.5345
H	4.2502	-3.2028	-3.5171
C	3.8548	-1.7397	-1.1904
H	4.5345	-0.8819	-1.3216
H	4.4730	-2.6343	-1.0553
H	3.2802	-1.5904	-0.2662

C	2.3199	-0.4621	-2.6588
H	3.1394	0.2639	-2.7819
H	1.6926	-0.1301	-1.8212
H	1.7214	-0.4294	-3.5806
C	-2.4481	-0.2998	2.2027
H	-2.4911	0.4646	2.9928
H	-3.3727	-0.2059	1.6153
H	-1.6023	-0.0384	1.5572
C	-3.5609	-2.0118	3.6111
H	-3.7454	-1.2099	4.3449
H	-3.4884	-2.9544	4.1701
H	-4.4425	-2.0603	2.9531
C	-1.0711	-1.6469	3.7683
H	-1.1438	-0.7635	4.4226
H	-0.1346	-1.5765	3.1980
H	-1.0043	-2.5334	4.4094
H	1.1513	4.2975	-1.3007
S	2.2926	6.4481	-1.9686
O	2.7359	5.6782	-3.1375
O	0.9932	5.9501	-1.4077
C	1.7903	8.0861	-2.6587
O	3.2949	6.7368	-0.9421
F	0.7879	7.9203	-3.5165
F	2.8059	8.6502	-3.2955
F	1.3867	8.9017	-1.6956

^{5}**11**$^{2+}$

W	-0.4343	2.2129	1.4089
C	1.8799	4.4207	3.3503
P	1.8915	2.8828	2.2518
N	0.5037	3.1057	-0.4570
P	-2.4896	2.7017	-0.0547
C	-3.4504	4.2387	0.4777
Cl	-1.5833	2.3032	3.4604
C	3.0840	1.5652	2.8807
C	-3.6260	1.2832	-0.5356
N	-0.0858	0.4305	0.9022
C	-1.6459	3.2358	-1.6222
H	-1.6389	4.3370	-1.6460
H	-2.2198	2.9177	-2.5046
C	-0.2197	2.7221	-1.6875
H	0.2975	3.1402	-2.5693
H	-0.1875	1.6273	-1.7650
C	1.9621	2.8948	-0.5419
H	2.1355	1.8139	-0.6041
H	2.3518	3.3514	-1.4689
C	2.6466	3.4960	0.6694
H	3.7283	3.2988	0.6453
H	2.5394	4.5920	0.6498
C	-4.4483	3.8637	1.5757
H	-4.8685	4.7880	2.0027
H	-3.9703	3.3054	2.3943
H	-5.2908	3.2756	1.1882
C	-4.1684	4.9313	-0.6823
H	-4.6883	5.8193	-0.2891
H	-4.9245	4.2976	-1.1586
H	-3.4783	5.2888	-1.4600
C	-2.4120	5.1934	1.0703
H	-2.8965	6.1459	1.3377
H	-1.5990	5.4413	0.3673
H	-1.9822	4.7823	1.9940
C	0.7246	5.2795	2.8282
H	0.6968	6.2308	3.3825
H	-0.2429	4.7853	2.9932
H	0.8239	5.5395	1.7613
C	1.5728	4.0183	4.7941
H	1.3593	4.9267	5.3788
H	2.4267	3.5222	5.2751
H	0.6895	3.3650	4.8617
C	3.1732	5.2356	3.2860
H	3.0509	6.1284	3.9197
H	3.4026	5.5969	2.2729
H	4.0461	4.6908	3.6625
C	-4.8490	1.7045	-1.3456
H	-5.3838	0.7962	-1.6663
H	-4.5809	2.2601	-2.2571
H	-5.5576	2.3069	-0.7627
C	-4.0350	0.5706	0.7530
H	-4.5405	-0.3748	0.5029
H	-4.7181	1.1681	1.3696
H	-3.1531	0.3322	1.3621
C	-2.7729	0.3360	-1.3757
H	-3.3359	-0.5903	-1.5616
H	-1.8527	0.0477	-0.8498
H	-2.5146	0.7632	-2.3551
C	3.2987	0.5961	1.7151
H	3.8898	-0.2606	2.0762
H	3.8675	1.0456	0.8879
H	2.3425	0.2199	1.3243
C	4.4337	2.1167	3.3333
H	5.1049	1.2724	3.5597
H	4.3533	2.7171	4.2488
H	4.9275	2.7229	2.5589
C	2.3976	0.8170	4.0239
H	3.0109	-0.0528	4.3108
H	1.4032	0.4610	3.7244
H	2.2733	1.4379	4.9193
W	-0.3107	-2.6062	0.4853
C	0.4958	-4.4744	-2.6773
P	0.9934	-2.9312	-1.7021
N	1.7331	-3.2114	1.2657
P	-0.8169	-3.3493	2.8938
C	-1.5311	-5.0957	2.9928
Cl	-2.4706	-3.0044	-0.3575
C	1.3362	-1.4667	-2.8427
C	-1.6754	-2.1551	4.0615
N	-0.0214	-0.7629	0.7611
C	0.9114	-3.5571	3.5463
H	1.1448	-4.6331	3.5594
H	0.9779	-3.2222	4.5912
C	1.9223	-2.8287	2.6808
H	2.9500	-3.0776	3.0002

H	1.8024	-1.7397	2.7503
C	2.8485	-2.8038	0.3899
H	2.8710	-1.7074	0.3732
H	3.8050	-3.1528	0.8183
C	2.6548	-3.3781	-1.0004
H	3.4650	-3.0598	-1.6727
H	2.7139	-4.4773	-0.9552
C	-3.0422	-5.0438	2.7585
H	-3.4196	-6.0717	2.6410
H	-3.3021	-4.4845	1.8475
H	-3.5758	-4.6019	3.6106
C	-1.2251	-5.8071	4.3121
H	-1.6386	-6.8268	4.2598
H	-1.6826	-5.3170	5.1786
H	-0.1477	-5.9130	4.5050
C	-0.8787	-5.8710	1.8455
H	-1.2305	-6.9149	1.8579
H	0.2208	-5.9122	1.9241
H	-1.1572	-5.4545	0.8669
C	0.0205	-5.4884	-1.6340
H	-0.1850	-6.4509	-2.1285
H	-0.9168	-5.1614	-1.1623
H	0.7718	-5.6899	-0.8523
C	-0.6776	-4.1485	-3.6038
H	-1.0823	-5.0908	-4.0055
H	-0.3722	-3.5391	-4.4651
H	-1.4951	-3.6388	-3.0718
C	1.6502	-5.0856	-3.4739
H	1.2765	-5.9842	-3.9899
H	2.4830	-5.4131	-2.8352
H	2.0492	-4.4146	-4.2425
C	-1.9679	-2.7319	5.4442
H	-2.3560	-1.9229	6.0835
H	-1.0688	-3.1305	5.9384
H	-2.7330	-3.5190	5.4194
C	-2.9631	-1.6878	3.3817
H	-3.3783	-0.8325	3.9372
H	-3.7303	-2.4716	3.3463
H	-2.7711	-1.3609	2.3512
C	-0.7221	-0.9706	4.2034
H	-1.2210	-0.1667	4.7641
H	-0.4441	-0.5515	3.2271
H	0.1896	-1.2356	4.7570
C	2.1039	-0.4421	-2.0053
H	2.2120	0.4807	-2.5974
H	3.1210	-0.7755	-1.7524
H	1.5654	-0.2002	-1.0779
C	2.1592	-1.8188	-4.0802
H	2.4158	-0.8878	-4.6113
H	1.6046	-2.4483	-4.7877
H	3.1071	-2.3207	-3.8348
C	-0.0061	-0.8577	-3.2452
H	0.1712	0.0813	-3.7944
H	-0.6187	-0.6364	-2.3621
H	-0.5905	-1.5138	-3.9008
H	0.3680	4.1167	-0.3512

H	1.7169	-4.2368	1.2467

$^{BS(2,2)}$**TS 11^{2+}** $\rightarrow$ 2 **9^{+}**

W	2.1504	0.2291	-0.9215
C	1.0776	-3.3100	-1.8835
P	2.5909	-2.2474	-1.5415
N	3.4547	-0.4856	0.7608
P	3.0270	2.4510	-0.0037
C	1.8183	3.6879	0.7345
Cl	1.9835	0.9309	-3.1592
C	3.9830	-2.5042	-2.7891
C	4.3226	3.2952	-1.0942
N	0.5172	-0.0046	-0.2976
C	4.0270	1.8107	1.4273
H	4.0369	2.5348	2.2549
H	5.0732	1.7130	1.0955
C	3.5139	0.4645	1.8978
H	2.4993	0.5420	2.3123
H	4.1673	0.0504	2.6848
C	3.0887	-1.8614	1.1823
H	3.6821	-2.1411	2.0694
H	2.0314	-1.8374	1.4785
C	3.3275	-2.8506	0.0552
H	4.4086	-2.9897	-0.0980
H	2.9335	-3.8408	0.3248
C	0.7249	3.9300	-0.3056
H	-0.0439	4.5827	0.1355
H	0.2413	2.9852	-0.5864
H	1.0988	4.4156	-1.2158
C	2.4395	5.0075	1.1835
H	1.6589	5.6002	1.6871
H	2.8100	5.6113	0.3448
H	3.2568	4.8683	1.9072
C	1.1959	2.9875	1.9417
H	0.3407	3.5840	2.2952
H	1.8979	2.8877	2.7827
H	0.7994	2.0010	1.6638
C	0.2693	-3.3306	-0.5841
H	-0.7029	-3.8093	-0.7826
H	0.0923	-2.3088	-0.2181
H	0.7569	-3.9197	0.2068
C	0.2611	-2.6071	-2.9732
H	-0.6928	-3.1420	-3.1077
H	0.7738	-2.6024	-3.9428
H	0.0469	-1.5625	-2.7046
C	1.4169	-4.7397	-2.2980
H	0.4832	-5.3209	-2.3642
H	2.0654	-5.2484	-1.5687
H	1.8956	-4.7871	-3.2848
C	5.2969	4.1585	-0.2883
H	6.0169	4.6113	-0.9885
H	5.8854	3.5749	0.4349
H	4.8081	4.9789	0.2477
C	3.6132	4.1349	-2.1586
H	4.3607	4.4975	-2.8817
H	3.1221	5.0191	-1.7308

H	2.8698	3.5460	-2.7160
C	5.1125	2.1860	-1.7925
H	5.9360	2.6393	-2.3666
H	4.4841	1.6350	-2.5051
H	5.5765	1.4770	-1.0867
C	5.0099	-1.4159	-2.4631
H	5.8551	-1.4857	-3.1659
H	5.4400	-1.5194	-1.4526
H	4.5823	-0.4095	-2.5778
C	4.6539	-3.8760	-2.6912
H	5.4955	-3.9013	-3.4015
H	3.9809	-4.6986	-2.9579
H	5.0742	-4.0833	-1.6962
C	3.4529	-2.2786	-4.2067
H	4.3048	-2.2563	-4.9044
H	2.9168	-1.3234	-4.3022
H	2.7924	-3.0941	-4.5319
H	4.4041	-0.5357	0.3732
W	-2.3568	1.0632	0.7802
C	-1.0785	-0.3789	4.0088
P	-2.6802	-0.1128	3.0392
N	-3.0942	-0.9727	0.1376
P	-3.5536	1.4715	-1.4635
C	-2.5921	2.5447	-2.6709
Cl	-2.5754	3.2437	1.6614
C	-4.0996	0.4664	4.1447
C	-5.3932	1.8963	-1.3795
N	-0.6877	0.8203	0.3516
C	-3.5336	-0.2510	-2.1644
H	-3.1225	-0.2404	-3.1830
H	-4.5730	-0.5999	-2.2560
C	-2.7367	-1.1967	-1.2807
H	-1.6558	-1.0237	-1.3781
H	-2.9420	-2.2453	-1.5592
C	-2.6803	-2.0599	1.0422
H	-3.0710	-3.0237	0.6704
H	-1.5857	-2.1121	1.0115
C	-3.1838	-1.7970	2.4511
H	-4.2850	-1.8384	2.4701
H	-2.8357	-2.5801	3.1411
C	-2.3385	3.8997	-2.0062
H	-1.6472	4.4807	-2.6365
H	-1.8878	3.7922	-1.0119
H	-3.2562	4.4899	-1.8935
C	-3.3021	2.7360	-4.0098
H	-2.5968	3.2111	-4.7100
H	-4.1715	3.4010	-3.9284
H	-3.6239	1.7880	-4.4670
C	-1.2598	1.8304	-2.9055
H	-0.5863	2.5033	-3.4565
H	-1.3674	0.9233	-3.5183
H	-0.7545	1.5540	-1.9710
C	-0.1263	-1.1755	3.1117
H	0.8474	-1.2394	3.6240
H	0.0289	-0.6912	2.1359
H	-0.4666	-2.2098	2.9527
C	-0.4518	0.9850	4.3029
H	0.5529	0.8320	4.7292
H	-1.0286	1.5664	5.0309
H	-0.3512	1.5883	3.3926
C	-1.2984	-1.1545	5.3071
H	-0.3169	-1.3796	5.7542
H	-1.8093	-2.1164	5.1496
H	-1.8631	-0.5761	6.0492
C	-6.1333	1.6971	-2.7035
H	-7.2125	1.8163	-2.5170
H	-5.9931	0.6923	-3.1297
H	-5.8545	2.4331	-3.4644
C	-5.5496	3.3379	-0.8905
H	-6.6107	3.5265	-0.6642
H	-5.2511	4.0645	-1.6580
H	-4.9654	3.5371	0.0211
C	-5.9943	0.9461	-0.3414
H	-7.0535	1.2029	-0.1842
H	-5.4895	1.0323	0.6302
H	-5.9770	-0.1063	-0.6703
C	-5.2224	0.8877	3.1957
H	-6.0942	1.2035	3.7901
H	-5.5615	0.0628	2.5502
H	-4.9219	1.7415	2.5701
C	-4.6196	-0.6348	5.0707
H	-5.4686	-0.2291	5.6436
H	-3.8738	-0.9763	5.7967
H	-4.9960	-1.5104	4.5224
C	-3.6629	1.6883	4.9554
H	-4.5540	2.1301	5.4281
H	-3.2056	2.4627	4.3219
H	-2.9682	1.4254	5.7641
H	-4.1165	-0.9285	0.1842

^{5}**11**$^{(OTf)2}$

W	-2.2907	-0.0239	-1.0178
C	-0.8994	3.5571	-1.3940
P	-2.4691	2.5180	-1.2609
N	-3.2438	0.5515	0.9205
P	-3.2545	-2.2440	-0.2226
C	-2.1034	-3.7076	0.0519
Cl	-2.2093	-0.5873	-3.3341
C	-3.7778	3.0922	-2.4986
C	-4.8078	-2.7360	-1.1718
N	-0.5594	-0.1854	-0.2942
C	-3.8703	-1.7505	1.4475
H	-3.7743	-2.5780	2.1649
H	-4.9465	-1.5200	1.3747
C	-3.1488	-0.5083	1.9421
H	-2.0870	-0.7023	2.1456
H	-3.6219	-0.1589	2.8753
C	-2.8439	1.8780	1.4222
H	-3.3863	2.0875	2.3599
H	-1.7703	1.8603	1.6445
C	-3.1869	2.9412	0.3919
H	-4.2819	2.9730	0.2844

H	-2.8577	3.9373	0.7234
C	-1.2574	-3.8823	-1.2084
H	-0.4405	-4.5939	-1.0091
H	-0.8061	-2.9257	-1.5044
H	-1.8400	-4.2538	-2.0612
C	-2.8201	-5.0033	0.4213
H	-2.0621	-5.7712	0.6476
H	-3.4460	-5.3898	-0.3937
H	-3.4484	-4.8890	1.3180
C	-1.1872	-3.3039	1.2037
H	-0.3914	-4.0542	1.3230
H	-1.7282	-3.2324	2.1574
H	-0.6867	-2.3488	0.9991
C	-0.1040	3.2792	-0.1185
H	0.8848	3.7569	-0.2040
H	0.0462	2.2006	0.0272
H	-0.5865	3.6938	0.7785
C	-0.0879	3.0560	-2.5882
H	0.9030	3.5388	-2.5874
H	-0.5656	3.2816	-3.5490
H	0.0563	1.9698	-2.5348
C	-1.1527	5.0575	-1.5090
H	-0.1884	5.5886	-1.4481
H	-1.7887	5.4409	-0.6968
H	-1.6134	5.3296	-2.4679
C	-5.8040	-3.5134	-0.3090
H	-6.6506	-3.8157	-0.9472
H	-6.2207	-2.8892	0.4941
H	-5.3784	-4.4272	0.1253
C	-4.4287	-3.5239	-2.4265
H	-5.3312	-3.6530	-3.0458
H	-4.0467	-4.5280	-2.1960
H	-3.6820	-2.9883	-3.0323
C	-5.4533	-1.4159	-1.5996
H	-6.4298	-1.6211	-2.0679
H	-4.8378	-0.9023	-2.3522
H	-5.6479	-0.7423	-0.7542
C	-4.8636	2.0119	-2.4834
H	-5.6900	2.3280	-3.1411
H	-5.2961	1.8326	-1.4868
H	-4.4769	1.0672	-2.8918
C	-4.4108	4.4334	-2.1248
H	-5.1860	4.6715	-2.8712
H	-3.6944	5.2641	-2.1180
H	-4.9134	4.3960	-1.1481
C	-3.1741	3.1528	-3.9023
H	-3.9895	3.2916	-4.6305
H	-2.6557	2.2166	-4.1607
H	-2.4789	3.9951	-4.0270
H	-4.2664	0.6707	0.7233
W	2.3240	-1.0262	0.3686
C	0.9522	-0.1581	3.8987
P	2.5003	-0.3933	2.8421
N	3.2597	0.9997	0.2490
P	3.3191	-1.0246	-1.9916
C	2.2079	-1.2929	-3.4855
Cl	2.2456	-3.3983	0.6074
C	3.8294	-1.3606	3.7748
C	4.9089	-2.0363	-2.1115
N	0.5902	-0.3956	-0.0086
C	3.8881	0.7298	-2.0935
H	3.7782	1.1194	-3.1158
H	4.9644	0.7688	-1.8547
C	3.1482	1.6001	-1.0930
H	2.0837	1.7043	-1.3418
H	3.6015	2.6057	-1.0813
C	2.8586	1.9162	1.3296
H	3.3942	2.8731	1.2069
H	1.7835	2.1114	1.2430
C	3.2136	1.3069	2.6747
H	4.3092	1.2204	2.7311
H	2.8904	1.9558	3.5019
C	1.4474	-2.6058	-3.2980
H	0.6177	-2.6556	-4.0209
H	1.0192	-2.6718	-2.2892
H	2.0854	-3.4856	-3.4490
C	2.9549	-1.2804	-4.8170
H	2.2176	-1.3308	-5.6352
H	3.6309	-2.1387	-4.9299
H	3.5386	-0.3578	-4.9598
C	1.2116	-0.1373	-3.4632
H	0.4538	-0.2870	-4.2465
H	1.6981	0.8298	-3.6510
H	0.6711	-0.0884	-2.5091
C	0.1354	0.9333	3.2045
H	-0.8357	1.0292	3.7155
H	-0.0526	0.6798	2.1520
H	0.6226	1.9182	3.2479
C	0.1387	-1.4513	3.8826
H	-0.8418	-1.2733	4.3543
H	0.6259	-2.2656	4.4319
H	-0.0309	-1.7953	2.8554
C	1.2413	0.2682	5.3359
H	0.2889	0.5228	5.8299
H	1.8865	1.1580	5.3931
H	1.7052	-0.5364	5.9222
C	5.9104	-1.4600	-3.1152
H	6.7775	-2.1395	-3.1616
H	6.2938	-0.4805	-2.7958
H	5.5043	-1.3785	-4.1311
C	4.5806	-3.4924	-2.4410
H	5.4933	-4.0962	-2.3114
H	4.2498	-3.6222	-3.4809
H	3.8114	-3.9007	-1.7678
C	5.5252	-1.9791	-0.7134
H	6.5177	-2.4583	-0.7292
H	4.9103	-2.5371	0.0065
H	5.6794	-0.9522	-0.3557
C	4.8996	-1.6876	2.7301
H	5.7432	-2.1938	3.2275
H	5.3105	-0.7984	2.2266
H	4.5055	-2.3903	1.9824

C	4.4746	-0.5655	4.9105
H	5.2605	-1.1883	5.3681
H	3.7680	-0.2927	5.7032
H	4.9658	0.3487	4.5489
C	3.2463	-2.6741	4.2978
H	4.0739	-3.3120	4.6482
H	2.7153	-3.2254	3.5062
H	2.5681	-2.5245	5.1498
H	4.2853	0.8712	0.4186
C	-6.8153	1.5062	2.9405
S	-7.0021	0.7347	1.2684
O	-5.8208	1.3194	0.5526
O	-6.8375	-0.7027	1.5355
O	-8.2896	1.1933	0.7810
F	-5.5899	1.2512	3.4295
F	-7.7040	1.0258	3.7928
F	-6.9526	2.8228	2.8718
C	6.8032	3.3265	0.4450
S	7.0140	1.4935	0.2955
O	6.8605	1.2491	-1.1466
O	8.3034	1.2098	0.8980
O	5.8366	1.0042	1.0857
F	7.7188	3.9722	-0.2552
F	6.8837	3.7075	1.7126
F	5.5959	3.6944	-0.0140

$^{BS(2,2)}$**TS 11**$^{(OTf)2}$ $\rightarrow$ 2 **9**^{OTf}

W	2.1136	0.1520	-0.7823
C	1.1903	-3.5890	-1.2118
P	2.6142	-2.3548	-1.2619
N	3.5033	-0.3980	0.8580
P	2.9760	2.4438	-0.0066
C	1.8018	3.7121	0.7443
Cl	1.7638	0.7199	-3.0602
C	3.7592	-2.6402	-2.7314
C	4.1984	3.2461	-1.2085
N	0.4865	-0.0176	-0.1163
C	4.0373	1.9151	1.4158
H	4.0557	2.6861	2.1993
H	5.0670	1.7875	1.0496
C	3.5655	0.5811	1.9626
H	2.5741	0.6501	2.4333
H	4.2803	0.2152	2.7186
C	3.3485	-1.7854	1.3447
H	4.0603	-1.9547	2.1684
H	2.3318	-1.9039	1.7442
C	3.6471	-2.7520	0.2137
H	4.7113	-2.6527	-0.0611
H	3.5053	-3.7954	0.5299
C	0.6784	3.9771	-0.2528
H	-0.0704	4.6307	0.2212
H	0.1764	3.0403	-0.5227
H	1.0268	4.4680	-1.1699
C	2.4667	5.0236	1.1539
H	1.7234	5.6351	1.6917
H	2.8055	5.6105	0.2895
H	3.3202	4.8747	1.8327
C	1.1984	3.0459	1.9826
H	0.3621	3.6639	2.3460
H	1.9228	2.9458	2.8041
H	0.7779	2.0622	1.7337
C	0.6902	-3.6048	0.2340
H	-0.2593	-4.1611	0.2823
H	0.5098	-2.5866	0.6021
H	1.3970	-4.1057	0.9106
C	0.0831	-3.0635	-2.1250
H	-0.8080	-3.7066	-2.0341
H	0.3833	-3.0539	-3.1809
H	-0.1935	-2.0395	-1.8439
C	1.5916	-5.0079	-1.6064
H	0.7241	-5.6727	-1.4602
H	2.4114	-5.3975	-0.9844
H	1.8899	-5.0847	-2.6601
C	5.2181	4.1418	-0.5014
H	5.9042	4.5505	-1.2611
H	5.8369	3.5807	0.2132
H	4.7635	4.9909	0.0215
C	3.4277	4.0349	-2.2682
H	4.1312	4.3474	-3.0565
H	2.9666	4.9460	-1.8617
H	2.6484	3.4190	-2.7424
C	4.9536	2.1050	-1.8963
H	5.7150	2.5419	-2.5634
H	4.2799	1.5087	-2.5258
H	5.4832	1.4423	-1.1949
C	4.6720	-1.4115	-2.7786
H	5.4439	-1.5668	-3.5494
H	5.2082	-1.2471	-1.8347
H	4.1097	-0.5099	-3.0536
C	4.6316	-3.8874	-2.5666
H	5.2463	-3.9951	-3.4754
H	4.0523	-4.8118	-2.4524
H	5.3307	-3.7860	-1.7247
C	2.9424	-2.6984	-4.0231
H	3.6370	-2.6862	-4.8787
H	2.2783	-1.8264	-4.1220
H	2.3429	-3.6163	-4.1042
H	4.4775	-0.3587	0.4541
W	-2.3843	1.1502	0.7249
C	-1.0918	-0.4373	3.8753
P	-2.6388	-0.3141	2.7903
N	-2.9249	-0.8002	-0.1732
P	-3.5806	1.8020	-1.4910
C	-2.5560	2.8826	-2.6447
Cl	-2.7320	3.2425	1.8351
C	-4.1856	-0.0992	3.8530
C	-5.3737	2.3820	-1.4048
N	-0.6924	0.9523	0.3451
C	-3.6418	0.1438	-2.2962
H	-3.4373	0.2262	-3.3732
H	-4.6603	-0.2679	-2.1964

C	-2.6639	-0.8036	-1.6246
H	-1.6178	-0.5098	-1.7920
H	-2.8084	-1.8200	-2.0227
C	-2.2791	-1.9042	0.5516
H	-2.4911	-2.8567	0.0364
H	-1.1965	-1.7321	0.5265
C	-2.8214	-1.9620	1.9720
H	-3.8952	-2.1994	1.9236
H	-2.3287	-2.7537	2.5555
C	-2.2413	4.2059	-1.9412
H	-1.4226	4.7126	-2.4765
H	-1.9222	4.0498	-0.9025
H	-3.1006	4.8868	-1.9269
C	-3.2485	3.1434	-3.9818
H	-2.5490	3.6859	-4.6387
H	-4.1489	3.7629	-3.8766
H	-3.5256	2.2135	-4.5013
C	-1.2549	2.1221	-2.9030
H	-0.5577	2.7711	-3.4548
H	-1.4065	1.2226	-3.5162
H	-0.7520	1.8165	-1.9764
C	0.0168	-1.0548	3.0231
H	0.9500	-1.0412	3.6099
H	0.1906	-0.5014	2.0902
H	-0.1887	-2.1056	2.7732
C	-0.6657	0.9741	4.2848
H	0.3187	0.9247	4.7795
H	-1.3668	1.4410	4.9866
H	-0.5775	1.6340	3.4120
C	-1.2930	-1.3191	5.1075
H	-0.3246	-1.4291	5.6228
H	-1.6377	-2.3311	4.8454
H	-1.9983	-0.8885	5.8297
C	-6.1728	1.9589	-2.6402
H	-7.1837	2.3906	-2.5566
H	-6.2971	0.8680	-2.6919
H	-5.7396	2.3198	-3.5818
C	-5.4342	3.8954	-1.1988
H	-6.4736	4.1706	-0.9570
H	-5.1518	4.4586	-2.0987
H	-4.7988	4.2196	-0.3606
C	-5.9704	1.7060	-0.1724
H	-7.0524	1.9132	-0.1342
H	-5.5192	2.1109	0.7432
H	-5.8516	0.6141	-0.1826
C	-5.2956	0.3373	2.8979
H	-6.2403	0.4081	3.4618
H	-5.4605	-0.3636	2.0656
H	-5.0784	1.3348	2.4936
C	-4.6054	-1.3994	4.5412
H	-5.5231	-1.2031	5.1189
H	-3.8533	-1.7863	5.2390
H	-4.8497	-2.1873	3.8153
C	-3.9689	1.0182	4.8747
H	-4.9380	1.2516	5.3442
H	-3.6026	1.9385	4.3942
H	-3.2772	0.7354	5.6798
H	-3.9493	-0.9790	-0.0609
C	-5.5820	-3.7953	-1.1535
S	-6.4128	-2.2028	-0.6940
O	-7.6984	-2.5960	-0.1467
O	-5.4442	-1.6640	0.3167
O	-6.4035	-1.4331	-1.9467
F	-5.2915	-4.5072	-0.0683
F	-4.4261	-3.5468	-1.7897
F	-6.3416	-4.5249	-1.9500
C	7.5377	-1.3478	1.9846
S	7.2018	-1.0849	0.1840
O	6.0847	-0.0849	0.2483
O	8.4381	-0.5621	-0.3659
O	6.7460	-2.4056	-0.2765
F	8.4280	-2.3068	2.1669
F	6.4069	-1.7035	2.6148
F	7.9800	-0.2340	2.5496

^{1}12^{+}

W	-0.3682	2.4585	0.3157
C	1.6562	4.3685	2.8460
P	1.4780	2.6278	2.1003
N	1.1279	3.3723	-0.7054
P	-1.5106	3.2851	-1.7422
C	-2.9657	4.4411	-1.3992
Cl	-1.6874	2.9734	2.3104
C	1.7482	1.2709	3.3816
C	-1.8988	1.9501	-3.0086
N	-0.2042	0.7146	0.0002
C	-0.2617	4.3991	-2.4962
H	-0.4850	5.4089	-2.1236
H	-0.3435	4.4305	-3.5926
C	1.1128	3.9706	-2.0391
H	1.7832	4.8506	-2.0445
H	1.5562	3.2559	-2.7590
C	2.5403	3.2982	-0.3228
H	3.1243	2.9050	-1.1763
H	2.9192	4.3268	-0.1609
C	2.8400	2.4439	0.8926
H	2.8283	1.3835	0.6045
H	3.8344	2.6728	1.3031
C	-4.2052	3.6420	-0.9993
H	-4.9872	4.3435	-0.6689
H	-4.0022	2.9577	-0.1610
H	-4.6171	3.0642	-1.8384
C	-3.2817	5.3366	-2.6003
H	-4.0834	6.0332	-2.3082
H	-3.6373	4.7781	-3.4729
H	-2.4237	5.9509	-2.9083
C	-2.5481	5.3324	-0.2222
H	-3.3251	6.0974	-0.0653
H	-1.6037	5.8690	-0.4038
H	-2.4557	4.7634	0.7128
C	0.9613	5.3697	1.9130
H	1.0872	6.3807	2.3321

H	-0.1186	5.1740	1.8485
H	1.3779	5.3736	0.8979
C	0.9724	4.4584	4.2132
H	1.0041	5.5085	4.5446
H	1.4798	3.8624	4.9831
H	-0.0817	4.1545	4.1594
C	3.1320	4.7529	2.9766
H	3.1966	5.7411	3.4596
H	3.6282	4.8405	1.9995
H	3.7058	4.0496	3.5941
C	-2.6804	2.4901	-4.2060
H	-2.7327	1.6987	-4.9708
H	-2.1982	3.3627	-4.6718
H	-3.7133	2.7568	-3.9452
C	-2.6659	0.8036	-2.3507
H	-2.8408	0.0151	-3.0994
H	-3.6421	1.1131	-1.9563
H	-2.0899	0.3536	-1.5348
C	-0.5472	1.4208	-3.4910
H	-0.7278	0.5599	-4.1518
H	0.0780	1.0729	-2.6582
H	0.0147	2.1658	-4.0726
C	1.9304	-0.0420	2.6185
H	1.9627	-0.8631	3.3518
H	2.8758	-0.0773	2.0596
H	1.1085	-0.2554	1.9241
C	3.0011	1.5228	4.2214
H	3.1583	0.6573	4.8854
H	2.9168	2.4107	4.8610
H	3.9035	1.6241	3.5993
C	0.5129	1.1311	4.2743
H	0.6714	0.2807	4.9571
H	-0.3919	0.9380	3.6830
H	0.3248	2.0189	4.8887
W	0.0644	-2.3174	-0.3234
C	2.5874	-4.3666	-2.2256
P	2.2196	-2.6340	-1.5359
N	1.2177	-3.0252	1.1401
P	-1.6909	-3.0907	1.2826
C	-2.3307	-4.8682	1.1072
Cl	-1.1194	-3.3275	-2.1976
C	2.7517	-1.2953	-2.7604
C	-3.1174	-1.8921	1.6246
N	-0.0914	-0.4967	-0.2251
C	-0.6464	-3.0940	2.7999
H	-1.0583	-3.6871	3.6290
H	-0.6067	-2.0454	3.1268
C	0.7573	-3.5424	2.4352
H	0.8327	-4.6474	2.4527
H	1.4523	-3.1979	3.2240
C	2.6761	-3.1833	1.0952
H	3.1065	-2.7945	2.0370
H	2.9461	-4.2582	1.0797
C	3.3224	-2.4616	-0.0733
H	3.3494	-1.3838	0.1430
H	4.3559	-2.7992	-0.2358
C	-3.4946	-4.9066	0.1141
H	-3.7473	-5.9595	-0.0917
H	-3.2170	-4.4362	-0.8409
H	-4.4017	-4.4258	0.5038
C	-2.7392	-5.4775	2.4499
H	-3.1489	-6.4842	2.2668
H	-3.5101	-4.9016	2.9755
H	-1.8792	-5.6017	3.1243
C	-1.1932	-5.7097	0.5210
H	-1.5192	-6.7612	0.4700
H	-0.2795	-5.6799	1.1306
H	-0.9547	-5.3877	-0.4993
C	1.7645	-5.3714	-1.4139
H	2.0397	-6.3916	-1.7274
H	0.6940	-5.2401	-1.6119
H	1.9350	-5.3023	-0.3310
C	2.1284	-4.4824	-3.6811
H	2.2037	-5.5392	-3.9843
H	2.7543	-3.9038	-4.3732
H	1.0782	-4.1763	-3.7964
C	4.0694	-4.7236	-2.1012
H	4.2359	-5.7031	-2.5781
H	4.3833	-4.8208	-1.0519
H	4.7329	-4.0020	-2.5945
C	-4.1661	-2.4309	2.5963
H	-4.9006	-1.6337	2.7971
H	-3.7265	-2.7177	3.5633
H	-4.7211	-3.2890	2.1958
C	-3.7594	-1.5400	0.2802
H	-4.5134	-0.7511	0.4356
H	-4.2584	-2.3966	-0.1887
H	-3.0085	-1.1674	-0.4286
C	-2.5197	-0.6189	2.2297
H	-3.2826	0.1750	2.2280
H	-1.6630	-0.2426	1.6581
H	-2.2102	-0.7625	3.2748
C	2.7748	0.0390	-2.0083
H	2.9021	0.8516	-2.7419
H	3.6193	0.1011	-1.3061
H	1.8441	0.2270	-1.4566
C	4.1384	-1.5305	-3.3580
H	4.4005	-0.6740	-4.0010
H	4.1818	-2.4301	-3.9854
H	4.9158	-1.6079	-2.5824
C	1.6884	-1.2219	-3.8602
H	1.8412	-0.3064	-4.4545
H	0.6749	-1.2009	-3.4361
H	1.7395	-2.0736	-4.5474
H	-2.0359	2.3241	-0.0138

^{3}TS 12$^+$ + HNMe$_3$$^+$ → 13^{2+} + NMe_3

W	-9.7708	-6.9799	-2.9421
C	-12.7644	-6.0538	-4.9301
P	-10.8727	-6.1352	-5.0581
N	-10.3567	-8.6734	-3.8095

P	-9.6445	-8.5903	-0.9124
C	-10.4529	-8.0914	0.7261
Cl	-10.8237	-5.2382	-1.7671
C	-10.1401	-4.6385	-5.9388
C	-7.9986	-9.5013	-0.6570
N	-8.0537	-6.4805	-3.2211
C	-10.7897	-9.8251	-1.6505
H	-11.8028	-9.4266	-1.4963
H	-10.7415	-10.7976	-1.1409
C	-10.5443	-9.9631	-3.1386
H	-11.4161	-10.4700	-3.5891
H	-9.6808	-10.6230	-3.3437
C	-10.6582	-8.8558	-5.2313
H	-10.0273	-9.6693	-5.6347
H	-11.6992	-9.2171	-5.3287
C	-10.4669	-7.6070	-6.0729
H	-9.4090	-7.4980	-6.3443
H	-11.0449	-7.6636	-7.0063
C	-9.7628	-6.8336	1.2628
H	-10.2612	-6.5317	2.1976
H	-9.8470	-5.9973	0.5557
H	-8.7033	-6.9976	1.4938
C	-10.3954	-9.2168	1.7610
H	-10.9631	-8.8985	2.6498
H	-9.3745	-9.4419	2.0956
H	-10.8613	-10.1452	1.3983
C	-11.9273	-7.7678	0.4516
H	-12.3533	-7.3141	1.3601
H	-12.5232	-8.6659	0.2381
H	-12.0611	-7.0407	-0.3611
C	-13.2258	-7.0519	-3.8594
H	-14.3251	-7.0142	-3.8059
H	-12.8467	-6.7910	-2.8607
H	-12.9459	-8.0884	-4.0866
C	-13.2161	-4.6571	-4.4935
H	-14.3050	-4.6833	-4.3314
H	-13.0245	-3.8872	-5.2517
H	-12.7488	-4.3516	-3.5467
C	-13.4097	-6.4344	-6.2653
H	-14.5017	-6.3268	-6.1702
H	-13.2159	-7.4826	-6.5357
H	-13.0906	-5.7957	-7.0984
C	-8.2281	-10.9452	-0.2016
H	-7.2481	-11.3985	0.0160
H	-8.6933	-11.5569	-0.9879
H	-8.8349	-11.0282	0.7080
C	-7.1285	-8.7517	0.3530
H	-6.1268	-9.2079	0.3600
H	-7.5223	-8.8054	1.3761
H	-6.9960	-7.6968	0.0757
C	-7.2560	-9.5246	-1.9951
H	-6.3629	-10.1601	-1.8887
H	-6.9043	-8.5256	-2.2755
H	-7.8592	-9.9383	-2.8145
C	-8.7175	-5.0030	-6.3688
H	-8.2467	-4.0998	-6.7882
H	-8.7037	-5.7620	-7.1642
H	-8.0950	-5.3447	-5.5296
C	-10.9298	-4.2384	-7.1862
H	-10.4048	-3.4064	-7.6826
H	-11.9441	-3.8877	-6.9598
H	-10.9983	-5.0608	-7.9140
C	-10.0628	-3.4841	-4.9354
H	-9.5860	-2.6176	-5.4213
H	-9.4654	-3.7624	-4.0550
H	-11.0475	-3.1596	-4.5784
W	-5.1489	-5.7071	-3.6082
C	-2.6718	-7.2044	-6.1540
P	-4.5056	-7.0388	-5.7318
N	-5.3995	-4.3043	-5.0165
P	-5.2487	-3.5644	-2.1881
C	-3.7316	-2.4275	-2.1170
Cl	-4.4383	-7.2700	-1.9096
C	-5.4425	-8.6507	-6.0576
C	-6.1498	-3.6564	-0.5283
N	-6.8804	-6.1885	-3.4209
C	-6.4172	-2.7397	-3.3371
H	-6.4926	-1.6590	-3.1462
H	-7.4017	-3.1781	-3.1122
C	-6.0364	-3.0101	-4.7845
H	-5.3869	-2.2057	-5.1674
H	-6.9519	-2.9599	-5.4025
C	-5.0823	-4.4212	-6.4378
H	-5.7859	-3.7972	-7.0153
H	-4.0843	-3.9935	-6.6366
C	-5.1779	-5.8468	-6.9554
H	-6.2358	-6.1129	-7.0927
H	-4.6851	-5.9555	-7.9331
C	-2.5918	-3.1102	-1.3693
H	-1.7184	-2.4385	-1.3681
H	-2.2955	-4.0424	-1.8603
H	-2.8370	-3.3311	-0.3252
C	-4.0622	-1.0958	-1.4411
H	-3.2020	-0.4196	-1.5700
H	-4.2260	-1.2044	-0.3613
H	-4.9355	-0.5940	-1.8829
C	-3.2574	-2.1477	-3.5479
H	-2.2738	-1.6557	-3.4949
H	-3.9272	-1.4606	-4.0803
H	-3.1386	-3.0631	-4.1457
C	-2.0932	-5.7905	-6.2900
H	-0.9979	-5.8702	-6.3723
H	-2.3055	-5.1494	-5.4230
H	-2.4464	-5.2846	-7.1993
C	-1.9709	-7.9265	-5.0084
H	-0.8851	-7.9223	-5.1953
H	-2.2817	-8.9735	-4.9111
H	-2.1541	-7.4345	-4.0475
C	-2.4212	-7.9439	-7.4682
H	-1.3478	-7.8682	-7.7043
H	-2.9687	-7.5086	-8.3171
H	-2.6585	-9.0134	-7.4025

C	-6.8833	-2.3554	-0.1928
H	-7.4202	-2.4999	0.7582
H	-7.6396	-2.0988	-0.9485
H	-6.2162	-1.4969	-0.0603
C	-5.1663	-4.0328	0.5799
H	-5.7403	-4.2758	1.4879
H	-4.4877	-3.2102	0.8412
H	-4.5724	-4.9211	0.3155
C	-7.1865	-4.7720	-0.6493
H	-7.7329	-4.8394	0.3035
H	-6.7161	-5.7450	-0.8439
H	-7.9345	-4.5773	-1.4294
C	-6.8539	-8.4677	-5.5028
H	-7.4652	-9.3315	-5.8091
H	-7.3429	-7.5596	-5.8821
H	-6.8571	-8.4237	-4.4101
C	-5.5527	-8.9585	-7.5539
H	-6.1399	-9.8832	-7.6726
H	-4.5869	-9.1253	-8.0404
H	-6.0846	-8.1679	-8.1033
C	-4.7744	-9.8026	-5.3043
H	-5.4390	-10.6805	-5.3381
H	-4.6020	-9.5518	-4.2462
H	-3.8207	-10.1053	-5.7566
H	-3.0017	-5.2898	-3.4825
H	-2.6266	-5.7738	-2.9148
N	-1.1679	-6.4895	-1.7631
C	-0.0104	-5.7588	-2.2547
H	0.1654	-6.0032	-3.3130
H	-0.1799	-4.6753	-2.1760
H	0.9113	-6.0005	-1.6895
C	-1.4449	-6.1693	-0.3691
H	-1.5308	-5.0877	-0.2243
H	-2.3878	-6.6428	-0.0623
H	-0.6368	-6.5343	0.2949
C	-0.9437	-7.9271	-1.8575
H	-1.8749	-8.4661	-1.6364
H	-0.6039	-8.2050	-2.8613
H	-0.1660	-8.2558	-1.1404

BS(1,1) **13**$^{2+}$

W	0.2304	1.7462	1.7638
C	2.8715	1.8947	4.3606
P	2.4571	0.9962	2.7381
N	1.4480	3.1560	1.0670
P	-1.5248	3.6287	1.2520
C	-2.6139	4.1897	2.6876
Cl	-0.7778	0.9412	3.7502
C	2.9534	-0.8201	2.7846
C	-2.4660	3.6070	-0.3968
N	-0.0353	0.4653	0.5154
C	-0.2761	4.9645	1.0691
H	-0.0840	5.3240	2.0892
H	-0.6609	5.8192	0.4986
C	1.0273	4.4437	0.4944
H	1.8111	5.1929	0.7098
H	0.9727	4.3576	-0.6064
C	2.9044	3.0634	0.9720
H	3.2117	3.2641	-0.0708
H	3.3549	3.8761	1.5729
C	3.4704	1.7227	1.3961
H	3.3804	1.0029	0.5705
H	4.5383	1.7971	1.6436
C	-3.6302	3.0921	3.0277
H	-4.0914	3.3361	3.9973
H	-3.1515	2.1076	3.1313
H	-4.4370	3.0187	2.2901
C	-3.3164	5.5060	2.3479
H	-3.8895	5.8349	3.2299
H	-4.0323	5.4010	1.5213
H	-2.6053	6.3086	2.1028
C	-1.7132	4.3955	3.9129
H	-2.3452	4.7485	4.7424
H	-0.9301	5.1539	3.7710
H	-1.2590	3.4505	4.2385
C	2.0584	3.1940	4.3700
H	2.3201	3.7626	5.2765
H	0.9789	2.9968	4.4220
H	2.2635	3.8409	3.5047
C	2.4738	1.0832	5.5966
H	2.6722	1.7079	6.4824
H	3.0632	0.1653	5.7138
H	1.4040	0.8333	5.5990
C	4.3593	2.2368	4.4219
H	4.5565	2.7592	5.3716
H	4.6558	2.9268	3.6175
H	5.0157	1.3585	4.3900
C	-2.5142	5.0033	-1.0317
H	-3.1370	4.9480	-1.9382
H	-1.5213	5.3579	-1.3383
H	-2.9648	5.7552	-0.3701
C	-3.8944	3.0890	-0.2238
H	-4.3347	2.9823	-1.2272
H	-4.5334	3.7866	0.3322
H	-3.9276	2.1062	0.2638
C	-1.6990	2.6576	-1.3170
H	-2.1540	2.6624	-2.3199
H	-1.7259	1.6212	-0.9619
H	-0.6422	2.9451	-1.4240
C	2.9638	-1.3550	1.3525
H	3.0789	-2.4472	1.4080
H	3.8188	-0.9745	0.7763
H	2.0280	-1.1450	0.8140
C	4.3499	-1.0123	3.3770
H	4.6347	-2.0687	3.2537
H	4.3880	-0.7912	4.4501
H	5.1107	-0.4082	2.8603
C	1.9079	-1.5958	3.5817
H	2.1736	-2.6642	3.5903
H	0.9065	-1.4910	3.1388
H	1.8453	-1.2708	4.6271
W	-0.5106	-1.7275	-1.5315

C	1.5154	-2.5472	-4.8317
P	1.5227	-1.6321	-3.1524
N	0.6592	-3.1026	-0.6853
P	-2.2494	-2.9160	-0.0768
C	-2.9776	-4.5464	-0.7226
Cl	-1.9898	-0.5870	-3.0145
C	2.2721	0.1035	-3.3270
C	-3.5655	-1.9202	0.8869
N	-0.2231	-0.3723	-0.3533
C	-0.9845	-3.3396	1.1904
H	-1.3470	-4.0910	1.9093
H	-0.8430	-2.4008	1.7494
C	0.3326	-3.7830	0.5671
H	0.3301	-4.8739	0.4107
H	1.1427	-3.5881	1.2961
C	1.9265	-3.6004	-1.2161
H	2.5786	-3.8882	-0.3715
H	1.7643	-4.5306	-1.7908
C	2.6708	-2.5706	-2.0597
H	3.1146	-1.8173	-1.3928
H	3.4986	-3.0239	-2.6238
C	-3.7886	-4.3127	-2.0021
H	-4.1501	-5.2882	-2.3623
H	-3.1894	-3.8725	-2.8128
H	-4.6620	-3.6684	-1.8463
C	-3.8420	-5.1987	0.3536
H	-4.0928	-6.2206	0.0284
H	-4.7882	-4.6790	0.5378
H	-3.2972	-5.2981	1.3037
C	-1.8366	-5.5296	-1.0177
H	-2.2625	-6.4054	-1.5322
H	-1.3795	-5.9011	-0.0916
H	-1.0430	-5.1176	-1.6584
C	1.4961	-4.0504	-4.5304
H	1.3760	-4.5941	-5.4793
H	0.6660	-4.3546	-3.8748
H	2.4393	-4.3936	-4.0824
C	0.2641	-2.1709	-5.6320
H	0.2476	-2.7714	-6.5542
H	0.2551	-1.1151	-5.9271
H	-0.6699	-2.3708	-5.0866
C	2.7775	-2.2736	-5.6602
H	2.7621	-2.9427	-6.5346
H	3.7009	-2.4976	-5.1052
H	2.8341	-1.2468	-6.0392
C	-3.7004	-2.4858	2.3068
H	-4.5201	-1.9526	2.8136
H	-2.7952	-2.3230	2.9078
H	-3.9454	-3.5524	2.3309
C	-4.9221	-1.9290	0.1761
H	-5.5991	-1.2565	0.7247
H	-5.3937	-2.9180	0.1645
H	-4.8518	-1.5595	-0.8575
C	-3.0883	-0.4727	0.9805
H	-3.8209	0.0997	1.5718
H	-3.0078	-0.0099	-0.0117
H	-2.1264	-0.3871	1.5000
C	1.9439	0.8985	-2.0654
H	2.4857	1.8561	-2.1070
H	2.2433	0.3873	-1.1396
H	0.8723	1.1186	-2.0001
C	3.7957	0.0250	-3.4651
H	4.1807	1.0532	-3.5491
H	4.1267	-0.5225	-4.3537
H	4.2712	-0.4199	-2.5786
C	1.6159	0.8103	-4.5174
H	1.9620	1.8560	-4.5404
H	0.5197	0.8265	-4.4280
H	1.8809	0.3674	-5.4843
H	-1.3351	-3.3096	-2.6542
H	-0.7135	-3.1371	-3.1061

$HNMe_3^+$

N	-1.5846	2.0385	-0.0072
C	-1.1434	2.7498	-1.2383
H	-1.5344	2.2247	-2.1184
H	-1.5225	3.7788	-1.2158
H	-0.0463	2.7575	-1.2641
C	-1.1515	2.7484	1.2276
H	-1.5301	3.7775	1.2035
H	-1.5486	2.2225	2.1045
H	-0.0546	2.7556	1.2608
C	-1.1549	0.6131	-0.0066
H	-1.5470	0.1184	0.8903
H	-1.5410	0.1195	-0.9067
H	-0.0580	0.5780	-0.0029
H	-2.6119	2.0425	-0.0106

NMe_3

N	-1.5460	2.0329	-0.0001
C	-1.1459	2.7243	-1.1962
H	-1.5527	2.2151	-2.0840
H	-1.5407	3.7522	-1.1905
H	-0.0393	2.7895	-1.3213
C	-1.1440	2.7229	1.1961
H	-1.5388	3.7508	1.1922
H	-1.5493	2.2126	2.0840
H	-0.0372	2.7880	1.3194
C	-1.1453	0.6515	-0.0013
H	-1.5447	0.1391	0.8878
H	-1.5461	0.1401	-0.8903
H	-0.0385	0.5111	-0.0022

HOTf

S	-0.8245	0.6261	0.3640
O	-0.5704	1.2880	1.6126
O	-0.3671	1.1050	-0.9200
C	-2.6580	0.3758	0.2323
O	-0.3846	-0.9133	0.5322
H	-0.1530	-1.2629	-0.3436
F	-3.2387	1.5478	0.0827

F	-3.1104	-0.2109	1.3195
F	-2.9128	-0.3838	-0.8209

OTf^-

S	-0.8247	0.4380	0.2376
O	-0.5303	1.1726	1.4763
O	-0.5203	1.1518	-1.0108
C	-2.6844	0.4044	0.2298
O	-0.4914	-0.9937	0.2510
F	-3.2075	1.6375	0.2289
F	-3.1775	-0.2273	1.3026
F	-3.1675	-0.2247	-0.8490

thf-HOTf

S	0.8752	-7.1310	-3.2106
O	0.4466	-8.3580	-2.5905
O	-0.0554	-6.0646	-3.5430
C	1.6710	-7.6170	-4.8101
O	2.1160	-6.5602	-2.4468
H	2.1951	-5.5260	-2.5353
F	0.7868	-8.2384	-5.5648
F	2.7021	-8.4081	-4.5913
F	2.0930	-6.5276	-5.4490
C	1.1315	-3.3418	-2.3351
O	2.3119	-4.0862	-2.6939
C	2.7778	-3.6822	-3.9854
C	1.5478	-3.1239	-4.6717
C	0.8573	-2.4116	-3.5121
H	0.3164	-4.0648	-2.1860
H	1.3271	-2.8088	-1.3929
H	3.5664	-2.9193	-3.8612
H	3.2138	-4.5614	-4.4787
H	1.7900	-2.4611	-5.5141
H	0.9239	-3.9534	-5.0381
H	1.3143	-1.4234	-3.3435
H	-0.2185	-2.2611	-3.6756

thf

C	-4.2173	0.8525	0.1171
O	-2.8159	0.8734	-0.0577
C	-2.3529	2.2012	-0.1897
C	-3.5138	3.0979	0.2239
C	-4.7072	2.2569	-0.2151
H	-4.6554	0.0778	-0.5333
H	-4.4641	0.5866	1.1642
H	-1.4563	2.3390	0.4370
H	-2.0589	2.3976	-1.2397
H	-3.5248	3.2330	1.3178
H	-3.4766	4.0944	-0.2390
H	-5.6466	2.5180	0.2933
H	-4.8685	2.3613	-1.3006

$(thf)_2H^+$

C	-0.5303	-7.0663	-2.5073
O	-1.6779	-6.2241	-2.8177
C	-2.9106	-6.9973	-2.8655
C	-2.5336	-8.3089	-2.2097
C	-1.0707	-8.4780	-2.6136
H	0.2717	-6.8303	-3.2202
H	-0.2010	-6.8067	-1.4905
H	-3.6832	-6.4249	-2.3358
H	-3.1989	-7.1178	-3.9218
H	-2.6314	-8.2340	-1.1158
H	-3.1730	-9.1352	-2.5481
H	-0.5225	-9.1774	-1.9685
H	-0.9936	-8.8444	-3.6492
H	-1.5777	-5.3129	-3.5671
C	-0.7878	-4.4226	-5.5450
O	-1.5451	-4.3746	-4.3068
C	-1.5809	-3.0281	-3.7504
C	-0.6054	-2.2490	-4.6103
C	-0.6889	-2.9685	-5.9546
H	0.1984	-4.8672	-5.3354
H	-1.3376	-5.0601	-6.2502
H	-2.6157	-2.6665	-3.8385
H	-1.3058	-3.0923	-2.6885
H	-0.8735	-1.1860	-4.6744
H	0.4130	-2.3141	-4.1971
H	-1.5916	-2.6647	-6.5067
H	0.1792	-2.7810	-6.6006

H_2

H	-0.3804	0.0000	0.0000
H	0.3804	0.0000	0.0000

References

[1] K. Abdur-Rashid, T.P. Fong, B. Greaves, D.G. Gusev, J.G. Hinman, S.E. Landau, A.J. Lough, R.H. Morris, *J. Am. Chem. Soc.* **2000**, *122*, 9155–9171.

[2] I. Krossing, A. Reisinger, *Eur. J. Inorg. Chem.* **2005**, 1979–1989.

[3] R.F. Jordan, S.F. Echols, *Inorg. Chem*. **1987**, *26*, 383–386.

[4] J. Meiners, A. Friedrich, E. Herdtweck, S. Schneider, *Organometallics* **2009**, *28*, 6331.

[5] Belford, R. L.; Nilges, M. J. In *EPR Symposium, 21st Rocky Mountain Conference* Denver, Colorado, August,**1979**.

[6] D.F. Evans, *J. Chem. Soc.* **1959**, 2003. (b) S. K. Sur, *J. Magn. Res.* **1989**, *82*, 169.

[7] a) W. Haberditzel, *Angew. Chem. Int. Ed. Engl.* **1966**, 5, 288. b) G. A. Bain, J. F. Berry, *J. Chem. Educ.* **2008**, 85, 532.

[8] E. Bill, *julX, Program for Simulation of Molecular Magnetic Data*, Max-Planck Institute for Chemical Energy Conversion, Mülheim/Ruhr, **2008**.

[9] G. A. Silantyev, M. Förster, B. Schluschaß, J. Abbenseth, C. Würtele, C. Volkmann, M. C. Holthausen, S. Schneider, *Angew. Chemie Int. Ed.* **2017**, *56*, 5872–5876.

[10] J. Polster, H. Lachmann, Spectrometric Titrations-Analysis of Chemical Equilibria; VCH-Verlagsgesellschaft: Weinheim, Germany, **1989**.

[11] APEX2 v2014.9-0/APEX3 v2016.9-0 (SAINT/SADABS/SHELXT/SHELXL), Bruker AXS Inc., Madison, WI, USA, **2014/2016**. b) G. M. Sheldrick, *Acta Cryst.* **2015**, A71, 3-8. c) G. M. Sheldrick, *Acta Cryst*. **2015**, C71, 3-8. d) G. M. Sheldrick, *Acta Cryst*. **2008**, A64, 112-122.

[12] (a) F. Neese, *Wiley Interdiscip. Rev. Comput. Mol. Sci.* **2012**, *2*, 73. (b) F. Neese, *Wiley Interdiscip. Rev. Comput. Mol. Sci.* **2017**, e1327.

[13] C. Adamo, V. Barone*, J. Chem. Phys*. **1999**, *110*, 6158–6170.

[14] (a) S. Grimme, J. Antony, S. Ehrlich, H. Krieg, *J. Chem. Phys.* **2010**, *132*, 154104. (b) S. Grimme, S. Ehrlich, L. Goerigk, *J. Comput. Chem*. **2011**, *32*, 1456.

[15] F. Neese, F. Wennmohs, A. Hansen, U. Becker, *Chem. Phys.* **2009**, *356*, 98.

[16] J. Abbenseth, M. Finger, C. Würtele, M. Kasanmascheff, S. Schneider, *Inorg. Chem. Front.* **2016**, *3*, 469–477.

[17] (a) K. Eichkorn, F. Weigend, O. Treutler, R. Ahlrichs, *Theor. Chem. Acc.* **1997**, *97*, 119. (b) D. Andrae, U. Haeussermann, M. Dolg, H. Stoll, H. Preuss, *Theor. Chim. Acta* **1990**, *77*, 123. (c) F. Weigend, R. Ahlrichs, *Phys. Chem. Chem. Phys.* **2005**, *7*, 3297. (d) F. Weigend, M. Häser, H. Patzelt, R. Ahlrichs, *Chem. Phys. Lett.* **1998**, *294*, 143.

[18] (a) T. Soda, Y. Kitagawa, T. Onishi, Y. Takano, Y. Shigeta, H. Nagao, Y. Yoshioka, K. Yamaguchi, *Chem. Phys. Lett.* **2000**, *319*, 223. (b) S. Yamanaka, T. Kawakami, H. Nagao, K. Yamaguchi, *Chem. Phys. Lett.* **1994**, *231*, 25. (c) Q. Knijnenburg, D. Hetterscheid, T. M. Kooistra, P. H. M. Budzelaar, *Eur. J. Inorg. Chem.* **2004**, 1204.

[19] S. Grimme, *Chem. Eur. J.* **2012**, *18*, 9955.

[20] Y. Zhao, D. G. Truhlar, *Theor. Chem. Acc.* **2007**, *120*, 215.

[21] A. V. Marenich, C. J. Cramer, D. G. Truhlar, *J. Phys. Chem. B* **2009**, *113*, 6378.

[22] S. J. Konezny, M. D. Doherty, O. R. Luca, R. H. Crabtree, G. L. Soloveichik, V. S. Batista, *J. Phys. Chem. C* **2012**, *116*, 6349.

[23] B. M. Lindley, R. S. van Alten, M. Finger, F. Schendzielorz, C. Würtele, A. J. M. Miller, I. Siewert, S. Schneider, J*. Am. Chem. Soc.* **2018**, *140*, 7922–7935.
